# Supplementary material for: Stereodivergent 1,3-difunctionalization of alkenes by charge relocation
Source: Nature. 2024 Jan 31;626(7997):92–7. doi: 10.1038/s41586-023-06938-0 (PMC10830407; doi:10.1038/s41586-023-06938-0)
Supplement: Supplementary file 1 — Supplementary Information [file 41586_2023_6938_MOESM1_ESM.pdf]

nature portfolio

https://doi.org/10.1038/s41586-023-06938-0

Supplementary information

Stereodivergent 1,3-difunctionalization of alkenes by charge relocation

In the format provided by the authors and unedited

Stereodivergent 1,3-difunctionalisation of alkenes by charge relocation

Bogdan R. Brutiu,[1,†] Giulia Iannelli,[1,†] Margaux Riomet,[1] Daniel Kaiser[1] and Nuno Maulide[1,2,*]

[1]Institute of Organic Chemistry, University of Vienna, Währinger Straße 38, 1090 Vienna, Austria

[2]Research Platform NeGeMac, Währinger Straße 38, 1090 Vienna, Austria

[†]Equal contribution

*Corresponding author; nuno.maulide@univie.ac.at

Table of Contents

1. General Information 2

2. Optimisation 3

2.1. Scope Overview 5

3. Experimental Procedures 9

3.1. Synthesis of acyl chlorides 9

3.2. Synthesis of alkene substrates 10

3.3. Synthesis of *syn*-alcohols 11

3.4. Synthesis of *anti*-alcohols 68

3.5. Synthesis using different nucleophiles 79

3.6. Synthesis of 1,4-dicarbonyls 86

3.7. Ipomeanol derivatives and trisubstituted alkene 93

3.8. Comparison with metal-catalysed 1,3-functionalisations (Pd and Ni) 98

3.9. Mechanistic study: Demonstration of charge relocation 103

3.10. NOESY experiment to demonstrate the relative stereochemistry 107

4. NMR Spectra 108

5. X-rays 215

6. References 242

1. General Information

Unless otherwise stated, all glassware was flame-dried before use and all reactions were performed under an atmosphere of argon. All solvents were distilled from appropriate drying agents prior to use or, if purchased in anhydrous form, used as received. All reagents were used as received from commercial suppliers, unless otherwise stated. Reaction progress was monitored by thin layer chromatography (TLC) performed on aluminium plates coated with silica gel F_{254} with 0.2 mm thickness. Chromatograms were visualised by fluorescence quenching with UV light at 254 nm, or by staining using either potassium permanganate or phosphomolybdic acid. Flash column chromatography was performed using silica gel 60 (230-400 mesh, Merck and co.), or pre-packed columns. Neat infrared spectra were recorded using a Perkin-Elmer Spectrum 100 FT-IR spectrometer. Wavenumbers (ν_{max}) are reported in cm^{-1}. HR-ESI-MS spectra (*m/z* 50-1900) were obtained in a maXis UHR ESI-Qq-TOF mass spectrometer (Bruker Daltonics, Bremen, Germany) in the positive and/or negative ion mode by direct infusion. The sum formulas of the detected ions were determined using Bruker Compass DataAnalysis 4.1 based on the mass accuracy ($\Delta m/z \leq 5$ ppm) and isotopic pattern matching (SmartFormula algorithm). All ^{1}H NMR, ^{13}C DEPTQ-135 NMR, ^{13}C CPD NMR and ^{19}F NMR spectra were recorded using a Bruker AV-400, AV-500, AV-600 or AV-700 spectrometer at 300 K. Chemical shifts are given in parts per million (ppm, δ), referenced to the solvent peak of $CDCl_3$, defined at δ = 7.26 ppm (^{1}H NMR) and δ = 77.16 (^{13}C NMR).[1] Coupling constants are quoted in Hz (*J*). ^{1}H NMR splitting patterns are designated as singlet (s), doublet (d), triplet (t), quartet (q) or heptet (hept) as they appeared in the spectrum. If the appearance of a signal differs from the expected splitting pattern, the observed pattern is designated as apparent (app). Splitting patterns that could not be interpreted or easily visualised are designated as multiplet (m) or broad (br). All melting points are reported as an average of two measurements.

2. Optimisation

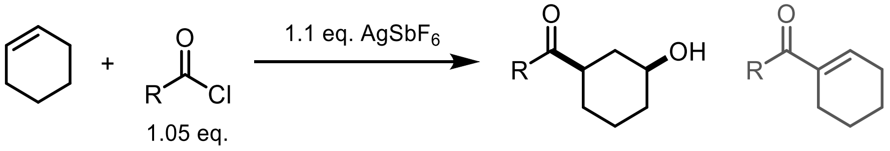

Entry	Acyl chloride	Silver salt or L.A.	Time	Temperature	Solvent	A	B
1	Nonanoyl chloride	$AgSbF_6$	**10 min**	23 °C	CH_2Cl_2	60%[(a)]	25%
2	Nonanoyl chloride	$AgSbF_6$	30 min	23 °C	CH_2Cl_2	40%[(a)]	45%
3	Nonanoyl chloride	$AgSbF_6$	60 min	23 °C	CH_2Cl_2	35%[(a)]	35%
4	Lauroyl chloride	$AgSbF_6$	10 min	0 °C	CH_2Cl_2	52%[(a)]	46%
5	Lauroyl chloride	$AgSbF_6$	10 min	23 °C	CH_2Cl_2	67%[(a)]	31%
6	**Lauroyl chloride**	**$AgSbF_6$**	**10 min**	**35 °C**	**CH_2Cl_2**	**70%[(a)]**	**30%**
7	Lauroyl chloride	$AgSbF_6$	10 min	35 °C	$CHCl_3$	22%[(a)]	62%
8	Lauroyl chloride	$AgSbF_6$	10 min	35 °C	1,2-DCE	23%[(a)]	57%
9	Lauroyl chloride	$AgSbF_6$	10 min	35 °C	1,2-Difluorobenzene	12%[(a)]	58%
10	Lauroyl chloride	$AgSbF_6$	10 min	35 °C	Cyclohexane	30%[(a)]	32%
11	Lauroyl chloride	$AgSbF_6$	10 min	35 °C	THF	n.d.	n.d.
12	Lauroyl chloride	AgOTf	10 min	35 °C	CH_2Cl_2	**24%**	**49%**
13	Lauroyl chloride	$AgBF_4$	10 min	35 °C	CH_2Cl_2	**11%**	**<5%**
14	Lauroyl chloride	$AgPF_6$	10 min	35 °C	CH_2Cl_2	**13%**	**5%**
15	Lauroyl chloride	$AgCO_3$	10 min	35 °C	CH_2Cl_2	no conversion	

16	Lauroyl chloride	$AgNO_3$	10 min	35 °C	CH_2Cl_2	no conversion	
**17**	Lauroyl chloride	$SnCl_4$	10 min	35 °C	CH_2Cl_2	**n.d.**	**1%**
18	Lauroyl chloride	$FeCl_3$	10 min	35 °C	CH_2Cl_2	**n.d.**	**14%**
19	Lauroyl chloride	BF_3 Et_2O	10 min	35 °C	CH_2Cl_2	no conversion	
20	Lauroyl chloride	AlCl3	10 min	35 °C	CH_2Cl_2	**n.d.**	**1%**
21	Lauroyl chloride	no Ag	10 min	35 °C	CH_2Cl_2	no conversion	
22	Lauroyl chloride	$InCl_3$	10 min	35 °C	CH_2Cl_2	**n.d.**	**6%**

[a] isolated yield; 1,2-DCE – 1,2-dichloroethane; THF – tetrahydrofuran; n.d. – not detected

2.1. Scope Overview

Below, a full representation of the scope of this reaction is depicted. Therein, the yields of the respective products are shown. In cases where regioisomeric mixtures were obtained, these have been denoted as r.r. In some cases, enone formation was observed by crude NMR and the corresponding NMR yields of the enone by-products (which were easily separated from the desired products) are reported in parentheses. It should be noted that enone formation was exclusively observed for products derived from acyl chlorides that are either comparatively electron rich or aliphatic. Thus, it can be inferred that the basicity of the carbonyl (being greater for electron-rich carbonyls) leads to increased levels of deprotonation. However, examples such as **20** and **21**, where no enone formation was observed, do not entirely agree with this empirical trend.

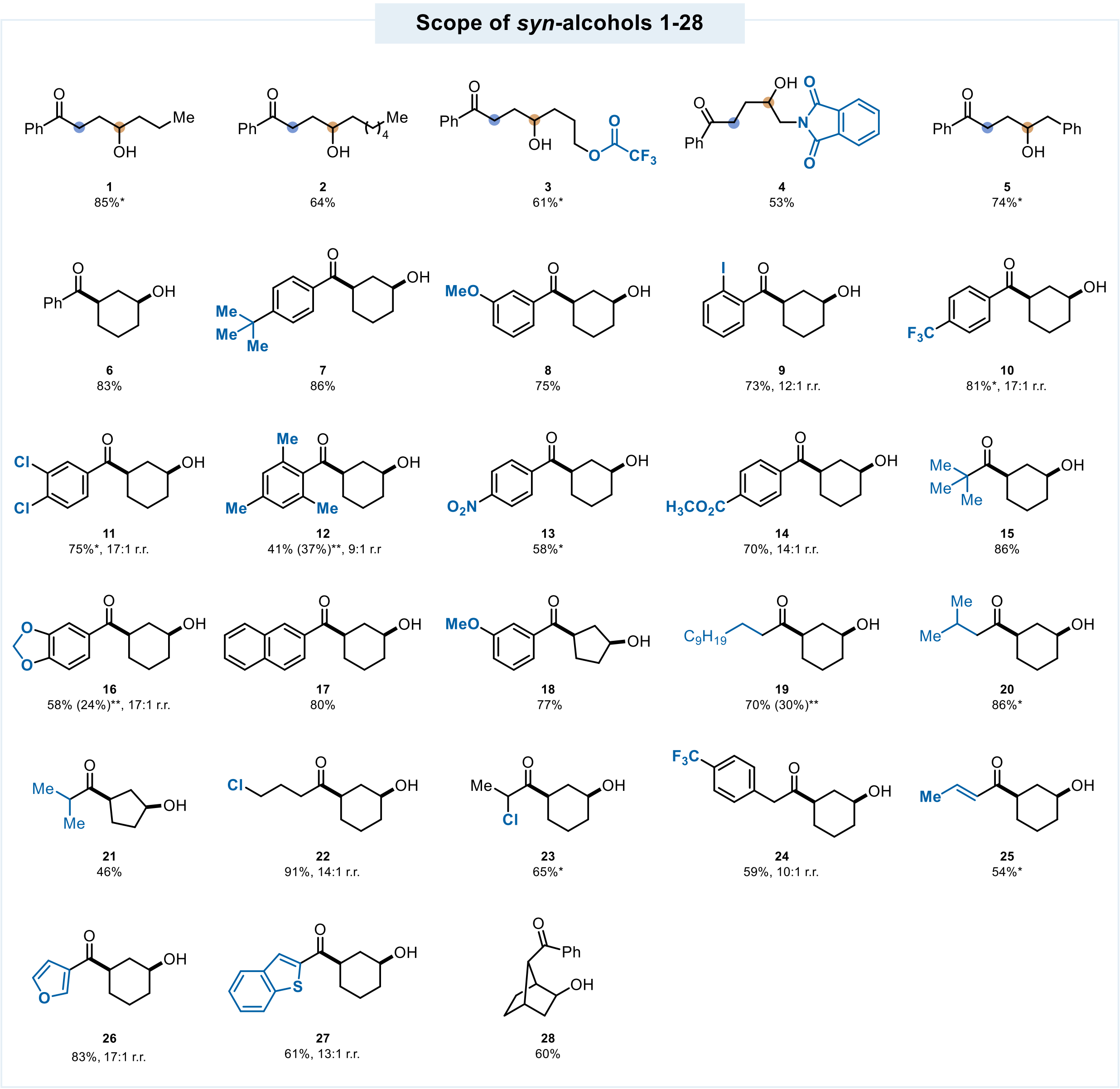

All products obtained with >20:1 diastereomeric (d.r.) and regioisomeric ratio (r.r.), unless otherwise mentioned. All yields correspond to isolated material. *The reported yields correspond to averages over three runs. **Amount of enone by-products observed in the crude mixtures.

Scope of *syn*-alcohols 55-82

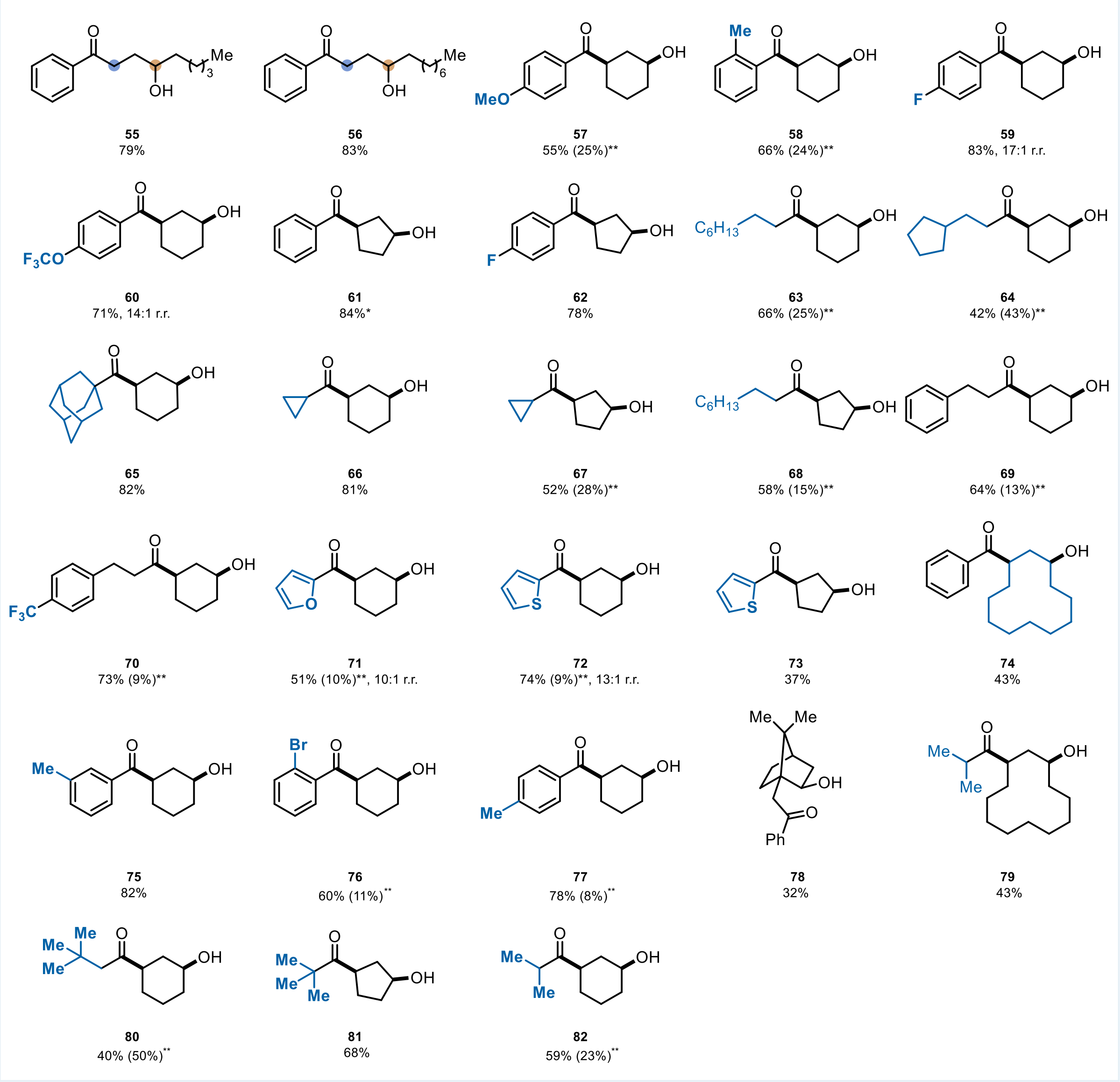

All products obtained with >20:1 diastereomeric (d.r.) and regioisomeric ratio (r.r.), unless otherwise mentioned. All yields correspond to isolated material. *The reported yields correspond to averages over three runs. **Amount of enone by-products observed in the crude mixtures.

Scope of *anti*-alcohols 29-37,83

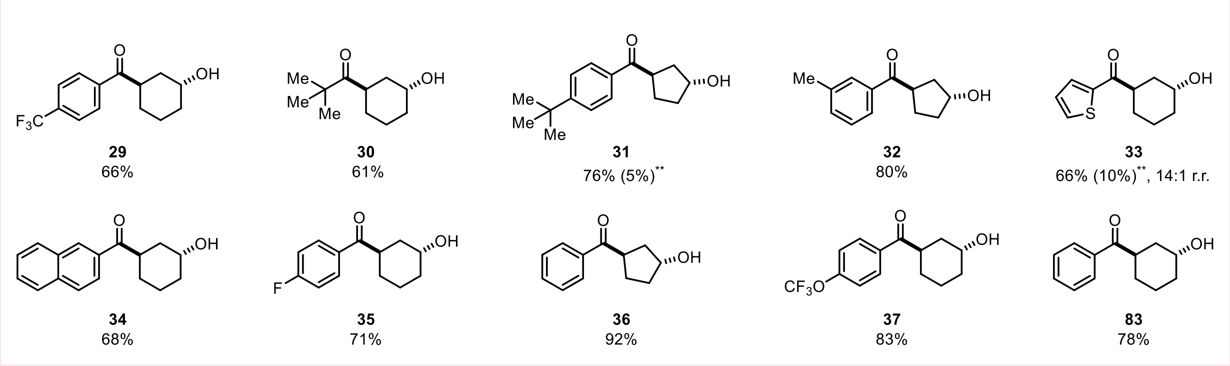

29 66%; **30** 61%; **31** 76% (5%)**; **32** 80%; **33** 66% (10%)**, 14:1 r.r.; **34** 68%; **35** 71%; **36** 92%; **37** 83%; **83** 78%

Scope of other nucleophiles 38-43

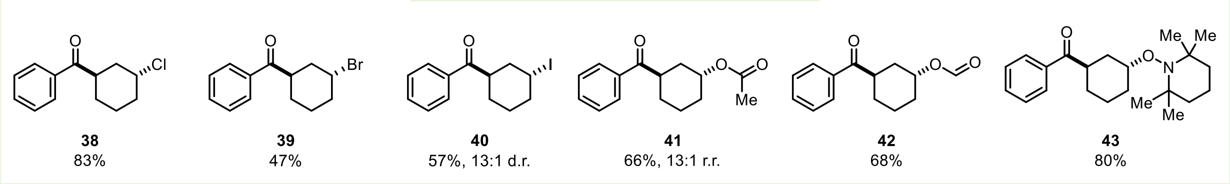

38 83%; **39** 47%; **40** 57%, 13:1 d.r.; **41** 66%, 13:1 r.r.; **42** 68%; **43** 80%

Scope of 1,4-dicarbonyls 44-49

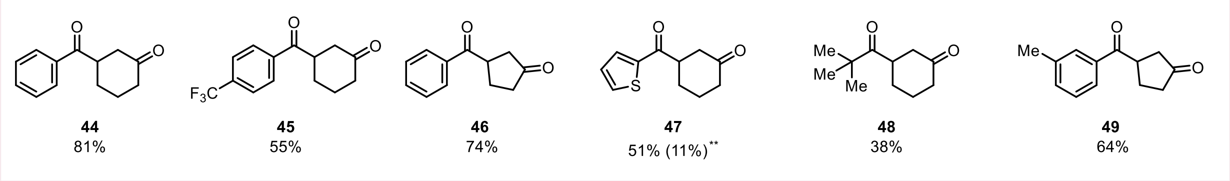

44 81%; **45** 55%; **46** 74%; **47** 51% (11%)**; **48** 38%; **49** 64%

Additonal scope entries 50-54

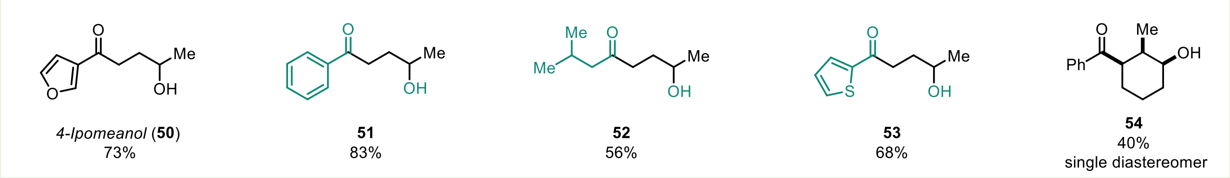

4-Ipomeanol (**50**) 73%; **51** 83%; **52** 56%; **53** 68%; **54** 40% single diastereomer

All products obtained with >20:1 diastereomeric (d.r.) and regioisomeric ratio (r.r.), unless otherwise mentioned. All yields correspond to isolated material. ** Amount of enone by-products observed in the crude mixtures.

3. Experimental Procedures

3.1. Synthesis of acyl chlorides

General Procedure 1 for the preparation of acyl chlorides

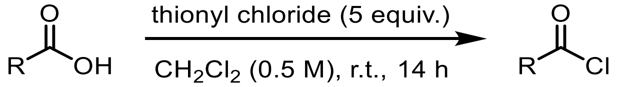

To a stirred solution of the carboxylic acid (1.0 equiv.) in anhydrous dichloromethane (0.1 M), thionyl chloride (5.0 or 10.0 equiv.) was added and the resulting mixture was stirred at ambient temperature for 14 h. Subsequently, removal of excess thionyl chloride under reduced pressure afforded the corresponding acyl chloride. The compound was used in the next step without further purification.

S1: adamantane-1-carbonyl chloride

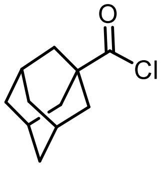

$C_{11}H_{15}ClO$
MW: 198.69

Synthesised following general procedure 1, using 1-adamantanecarboxylic acid (250 mg, 1.36 mmol, 1.00 equiv.), thionyl chloride (560 µL, 6.8 mmol, 5.0 equiv.) and dichloromethane (14 mL). No analytical data were obtained for this compound, which was used without further purification.

S2: 2-(4-(trifluoromethyl)phenyl)acetyl chloride

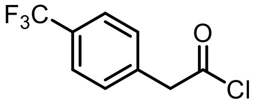

$C_9H_6ClF_3O$
MW: 222.59

Synthesised following general procedure 1, using 4-trifluoromethylphenylacetic acid (210 mg, 1.00 mmol, 1.00 equiv.), thionyl chloride (601 µL, 500 mmol, 5.0 equiv.) and dichloromethane (10 mL). No analytical data were obtained for this compound, which was used without further purification.

S3: 3-(4-(trifluoromethyl)phenyl)propanoyl chloride

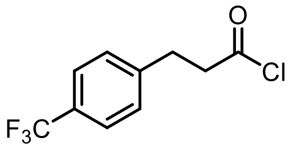

$C_{10}H_8ClF_3O$
MW: 236.62

Synthesised following general procedure 1, using 4-(trifluoromethyl)hydrocinnamic acid (166 mg, 0.75 mmol, 1.00 equiv.), thionyl chloride (549 µL, 7.46 mmol, 9.95 equiv.) and dichloromethane (10 mL). No analytical data were obtained for this compound, which was used without further purification.

3.2. Synthesis of alkene substrates

3.2.1. Synthesis of compound S4:

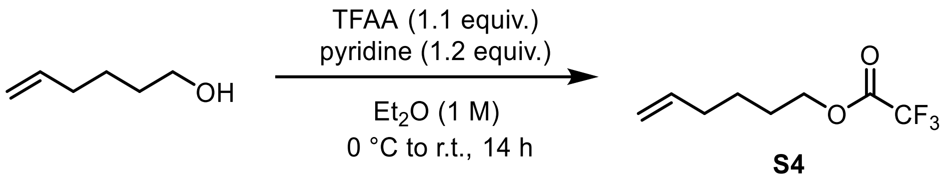

S4: hex-5-en-1-yl 2,2,2-trifluoroacetate

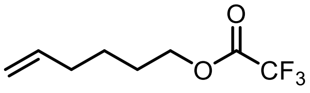

$C_8H_{11}F_3O_2$
MW: 196.17

Compound **S4** was prepared using a previously reported procedure,[2] using 5-hexen-1-ol (344 µL, 3.00 mmol, 1.00 equiv.), pyridine (291 µL, 3.59 mmol, 1.20 equiv.), trifluoroacetic anhydride (458 µL, 3.29 mmol, 1.10 equiv.) and diethyl ether (3 mL). The resulting crude material was purified by flash column chromatography on silica gel (pentane, Rf = 0.80 in heptane/ethyl acetate 90:10) to afford the desired compound as a colourless oil (423 mg, 2.16 mmol, 72%).

^{1}H NMR (400 MHz, CDCl$_3$) δ 5.79 (ddt, *J* = 16.9, 10.2, 6.7 Hz, 1H), 5.09 – 4.94 (m, 2H), 4.36 (t, *J* = 6.6 Hz, 2H), 2.17 – 1.99 (m, 2H), 1.82 – 1.68 (m, 2H), 1.55 – 1.44 (m, 2H).

^{13}C NMR (101 MHz, CDCl$_3$) δ 157.7 (q, *J* = 42.6 Hz), 137.9, 118.9, 114.8 (q, *J* = 285.7 Hz), 68.2, 33.2, 27.6, 24.9.

^{19}F NMR (377 MHz, CDCl$_3$) δ -75.12.

HRMS: We were unable to obtain HRMS data for this compound, despite several attempts.

IR (neat) ν_{max}: 3106, 3001, 2954, 2821, 1799, 1613, 1421, 1356, 1211, 1109, 903, 723, 665 cm^{-1}.

3.3. Synthesis of *syn*-alcohols

3.3.1. General Procedure 2 for the 1,3-hydroxyacylation of alkenes with acylium ions

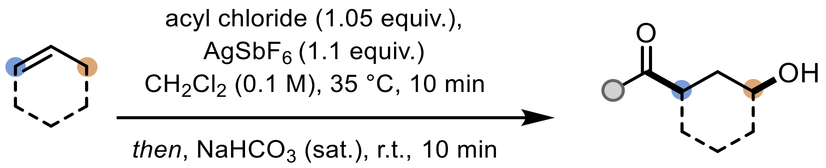

1–28, 55–82

All reactions were performed on a 0.2 mmol scale.

In a flame-dried Schlenk tube or oven-dried vial charged with a magnetic stir bar at ambient temperature, a solution of alkene (1.00 equiv.) and acyl chloride (1.05 equiv.) in dichloromethane (0.1 M) was treated with silver hexafluoroantimonate (1.10 equiv.) under argon atmosphere and the resulting suspension was immediately placed in a pre-heated oil (or sand) bath at 35 °C. After vigorously stirring the reaction mixture at the same temperature for 10 min (30 minutes for linear alkenes), the reaction vessel was removed from the oil (or sand) bath and a saturated aqueous solution of sodium bicarbonate (equal volume to that of dichloromethane) was immediately added, followed by vigorous stirring for 10 min. After this time, the phases were separated, the aqueous phase was extracted with dichloromethane (3 x 5 mL). The combined organic phases were dried over anhydrous magnesium sulfate, filtered and the filtrate was concentrated under reduced pressure. The resulting crude material was purified by flash column chromatography on silica gel (heptane/ethyl acetate) to give the title compounds **1–28, 55–82**. All *syn*-alcohols were obtained as single diastereomers. Where the products were formed as regioisomeric mixtures (providing the inseparable δ-hydroxyl product, as judged by ^{1}H NMR analysis), this has been indicated.

1: 4-hydroxy-1-phenylheptan-1-one

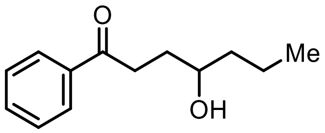

$C_{13}H_{18}O_2$
MW: 206.29
Yield: 85%, >20:1 r.r. (δ)

Synthesised following the general procedure, using 1-hexene (24.8 µL, 0.20 mmol, 1.00 equiv.), benzoyl chloride (27.9 µL, 0.24 mmol, 1.20 equiv.), silver hexafluoroantimonate (89.3 mg, 0.26 mmol, 1.30 equiv.) and dichloromethane (2 mL). Purification by flash column chromatography (heptane/ethyl acetate 90:10 to 20:80, Rf = 0.41 in heptane/ethyl acetate 66:33) gave the title compound (>20:1 r.r., 35.1 mg, 85%) as a colourless oil.

^{1}H NMR (400 MHz, $CDCl_3$): *δ* 7.99 – 7.87 (m, 2H), 7.56 (ddd, *J* = 6.7, 3.9, 1.3 Hz, 1H), 7.48 – 7.44 (m, 2H), 3.70 (app s, 1H), 3.22 – 3.09 (m, 2H), 1.99 (dtd, *J* = 14.4, 7.3, 3.4 Hz, 1H), 1.85 – 1.76 (m, 2H), 1.51 – 1.35 (m, 4H), 0.96 – 0.92 (m, 3H).

^{13}C NMR (101 MHz, $CDCl_3$): *δ* 201.0, 137.1, 133.2, 128.7 (2C), 128.3 (2C), 71.4, 40.2, 35.1, 31.5, 19.0, 14.2.

HRMS (ESI$^+$): exact mass calculated for $[M+Na]^+$ ($C_{13}H_{18}O_2Na$) requires *m/z* 229.1199, found *m/z* 229.1194.

IR (neat) ν_{max}: 2961, 2936, 1717, 1688, 1450, 1315, 1274, 1112, 1095, 1003, 762, 713, 701 cm^{-1}.

2: 4-hydroxy-1-phenyldecan-1-one

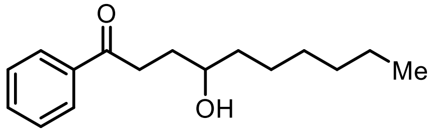

$C_{16}H_{24}O_2$
MW: 248.37
Yield: 64%, >20:1 r.r. (δ)

Synthesised following the general procedure, using 1-nonene (34.6 µL, 0.20 mmol, 1.00 equiv.), benzoyl chloride (24.4 µL, 0.21 mmol, 1.05 equiv.), silver hexafluoroantimonate (75.6 mg, 0.22 mmol, 1.10 equiv.) and dichloromethane (2 mL). Purification by flash column chromatography (heptane/ethyl acetate 90:10 to 20:80, Rf = 0.42 in heptane/ethyl acetate 75:25) gave the title compound (>20:1 r.r., 32.1 mg, 64%) as a colourless oil.

^{1}H NMR (600 MHz, $CDCl_3$) δ 7.99 – 7.98 (m, 2H), 7.57 – 7.55 (m, 1H), 7.45 (dd, *J* = 16.7, 9.0 Hz, 2H), 3.68 – 3.67 (m, 1H), 3.18 – 3.11 (m, 2H), 1.98 – 1.97 (m, 1H), 1,81 – 1.80 (m, 1H), 1.74 (brs, 1H), 1.50 – 1.51 (m, 2H), 1.51– 1.49 (m, 1H), 1.35 – 1.29 (m, 7H), 0.88 (t, *J* = 6.7 Hz, 3H).

^{13}C NMR (151 MHz, $CDCl_3$) δ 201.0, 137.1, 133.2, 128.7 (2C) , 128.3 (2C) , 71.7, 38.0, 35.1, 31.9, 31.5, 29.5, 25.8, 22.7, 14.2.

HRMS (ESI$^+$): exact mass calculated for $[M+Na]^+$ ($C_{16}H_{24}O_2Na$) requires *m/z* 271.1669, found *m/z* 271.1662.

IR (neat) ν_{max}: 2961, 2931, 1718, 1688, 1468, 1315, 1271, 1112, 1069, 992, 774, 744, 668 cm^{-1}.

3: 4-hydroxy-7-oxo-7-phenylheptyl 2,2,2-trifluoroacetate

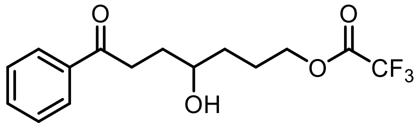

$C_{15}H_{17}F_3O_4$
MW: 318.29
Yield: 61%, >20:1 r.r. (δ)

Synthesised following the general procedure, using hex-5-en-1-yl 2,2,2-trifluoroacetate (39.2 mg, 0.20 mmol, 1.00 equiv.), benzoyl chloride (24.2 µL, 0.21 mmol, 1.05 equiv.), silver hexafluoroantimonate (75.6 mg, 0.22 mmol, 1.10 equiv.) and dichloromethane (2 mL). Purification by flash column chromatography (heptane/ethyl acetate 90:10 to 20:80, Rf = 0.39 in heptane/ethyl acetate 66:33) gave the title compound (>20:1 r.r., 39.1 mg, 61%) as a pale-orange oil.

^{1}H NMR (400 MHz, $CDCl_3$) δ 7.99 – 7.94 (m, 2H), 7.57 (dd, *J* = 13.0, 5.7 Hz, 1H), 7.47 (t, *J* = 7.7 Hz, 2H), 4.48 – 4.32 (m, 2H), 3.72 (dq, *J* = 12.2, 4.0 Hz, 1H), 3.17 (t, *J* = 6.8 Hz, 2H), 2.08 – 1.91 (m, 2H), 1.91 – 1.77 (m, 2H), 1.69 – 1.47 (m, 2H).

^{13}C NMR (101 MHz, $CDCl_3$): δ 201.0, 157.7 (q, *J* = 43.6 Hz), 136.9, 133.5, 128.8 (2C), 128.3 (2C), 114.5 (q, *J* = 285.5 Hz), 71.0, 68.3, 35.1, 33.8, 31.5, 24.8.

As a long acquisition time in chloroform induces a partial decomposition of the compound, both ^{13}C CPD (using a shorter acquisition time) and ^{13}C DEPTQ-135 NMR are attached.

^{19}F NMR (377 MHz, $CDCl_3$) *δ* -75.09.

HRMS (ESI$^+$): exact mass calculated for $[M+Na]^+$ ($C_{15}H_{17}F_3O_4Na$) requires *m/z* 341.0971, found *m/z* 341.0951.

IR (neat) ν_{max}: 3327, 2944, 1715, 1662, 1601, 1450, 1315, 1276, 1177, 1112, 1023, 753, 713, 682 cm^{-1}.

4: 2-(2-hydroxy-5-oxo-5-phenylpentyl)isoindoline-1,3-dione

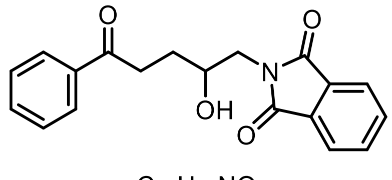

$C_{19}H_{17}NO_4$
MW: 323.35
Yield: 53%, >20:1 r.r. (δ)

Synthesised following the general procedure, using *N*-(3-buten-1-yl)phthalimide (41.1 mg, 0.20 mmol, 1.00 equiv.), benzoyl chloride (24.2 µL, 0.21 mmol, 1.05 equiv.), silver hexafluoroantimonate (75.6 mg, 0.22 mmol, 1.10 equiv.) and dichloromethane (2 mL). Purification by flash column chromatography (heptane/ethyl acetate 90:10 to 20:80, Rf = 0.32 in heptane/ethyl acetate 66:33) gave the title compound (>20:1 r.r., 34.2 mg, 53%) as a pale-yellow solid.

^{1}H NMR (400 MHz, CDCl$_3$): δ 7.94 (d, *J* = 7.7 Hz, 2H), 7.85 (dd, *J* = 5.4, 3.1 Hz, 2H), 7.72 (dd, *J* = 5.4, 3.1 Hz, 2H), 7.59 – 7.56 (m, 1H), 7.46 (t, *J* = 7.6 Hz, 2H), 4.26 (dt, *J* = 12.4, 4.2 Hz, 1H), 3.92 (t, *J* = 6.6 Hz, 2H), 3.49 (d, *J* = 3.7 Hz, 1H), 3.23 – 3.08 (m, 2H), 1.95 – 1.88 (m, 2H).

^{13}C NMR (101 MHz, CDCl$_3$): δ 200.1, 168.8 (2C), 137.0, 134.2 (2C), 133.7, 132.3 (2C), 128.9 (2C), 128.3 (2C), 123.5 (2C), 65.4, 45.0, 35.4, 34.9.

HRMS (ESI$^+$): exact mass calculated for $[M+Na]^+$ ($C_{19}H_{17}NO_4Na$) requires *m/z* 346.1050, found *m/z* 346.1039.

IR (neat) ν_{max}: 3505, 3061, 2926, 1769, 1704, 1596, 1579, 1466, 1477, 1396, 1372, 1276, 1211, 1187, 1127, 1088, 1059, 1031, 1001, 959, 875, 794, 754 cm^{-1}.

Melting point: 112-113 °C.

5: 4-hydroxy-1,5-diphenylpentan-1-one

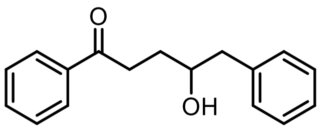

$C_{17}H_{18}O_2$
MW: 254.33
Yield: 74%, >20:1 r.r. (δ)

Synthesised following the general procedure, using 4-phenyl-1-butene (30.0 µL, 0.20 mmol, 1.00 equiv.), benzoyl chloride (24.4 µL, 0.21 mmol, 1.05 equiv.), silver hexafluoroantimonate (75.6 mg, 0.22 mmol, 1.10 equiv.) and dichloromethane (2 mL). Purification by flash column chromatography (heptane/ethyl acetate 90:10 to 20:80) gave the title compound (>20:1 r.r., 37.6 mg, 74%, Rf = 0.28 in heptane/ethyl acetate 75:25) as a pale-yellow oil.

^{1}H NMR (400 MHz, $CDCl_3$): *δ* 7.99 – 7.97 (m, 2H), 7.58 – 7.54 (m, 1H), 7.47 (dd, *J* = 10.4, 4.7 Hz, 2H), 7.36 – 7.30 (m, 2H), 7.25 – 7.22 (m, 3H), 3.94 (dt, *J* = 12.5, 4.1 Hz, 1H), 3.25 – 3.11 (m, 2H), 2.88 (dd, *J* = 13.5, 4.6 Hz, 1H), 2.75 (dd, *J* = 13.5, 8.2 Hz, 1H), 2.09 (dtd, *J* = 10.5, 7.3, 3.3 Hz, 1H), 1.93 – 1.84 (m, 2H).

^{13}C NMR (101 MHz, $CDCl_3$): *δ* 200.8, 138.4, 137.1, 133.2, 129.6 (2C), 128.8 (2C), 128.7 (2C), 128.2 (2C), 126.7, 72.3, 44.6, 35.1, 30.9.

HRMS (ESI$^+$): exact mass calculated for $[M+Na]^+$ ($C_{17}H_{18}O_2Na$) requires *m/z* 277.1199, found *m/z* 277.1193.

IR (neat) ν_{max}: 3062, 3029, 2925, 1715, 1602, 1584, 1494, 1451, 1315, 1271, 1176, 1111, 1069, 1026, 748 cm^{-1}.

6: *syn*-(3-hydroxycyclohexyl)(phenyl)methanone

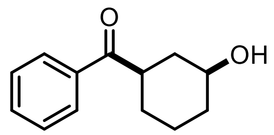

$C_{13}H_{16}O_2$
MW: 204.27
Yield: 83%, >20:1 r.r. (δ)

Synthesised following the general procedure, using cyclohexene (20.3 µL, 0.20 mmol, 1.00 equiv.), benzoyl chloride (24.4 µL, 0.21 mmol, 1.05 equiv.), silver hexafluoroantimonate (75.6 mg, 0.22 mmol, 1.10 equiv.) and dichloromethane (2 mL). Purification by flash column chromatography (heptane/ethyl acetate 90:10 to 20:80, Rf = 0.35 in heptane/ethyl acetate 75:25) gave the title compound (>20:1 r.r., 34.1 mg, 83%) as a colourless solid.

^{1}H NMR (600 MHz, CDCl$_3$): δ 7.93 – 7.92 (m, 2H), 7.59 – 7.52 (m, 1H), 7.47 – 7.46 (m, 2H), 3.77 (td, J = 10.5, 4.2 Hz, 1H), 3.37 (tt, J = 11.2, 3.4 Hz, 1H), 2.15 – 2.13 (m, 1H), 2.02 (app d, J = 12.4 Hz, 2H), 1.90 – 1.84 (m, 2H), 1.58 – 1.49 (m, 1H), 1.47 – 1.39 (m, 2H), 1.33 – 1.22 (m, 1H).

^{13}C NMR (151 MHz, CDCl$_3$): δ 202.6, 136.1, 133.2, 128.8 (2C), 128.4 (2C), 70.1, 44.1, 37.8, 35.3, 28.6, 23.5.

HRMS (ESI$^+$): exact mass calculated for $[M+Na]^+$ ($C_{13}H_{16}O_2Na$) requires *m/z* 227.1043, found *m/z* 227.1043.

IR (neat)ν_{max}: 3390, 2935, 2858, 1676, 1596, 1580, 1447, 1361, 1261, 1234, 1208, 1180, 1135, 1113, 1059, 1012, 948, 899, 852, 805, 750, 699 cm^{-1}.

Melting point: 88-89 °C.

7: *syn*-(4-(*tert*-butyl)phenyl)(3-hydroxycyclohexyl)methanone

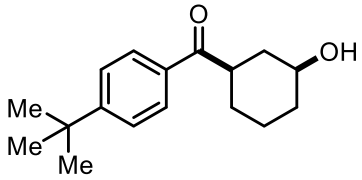

$C_{17}H_{24}O_2$
MW: 260.38
Yield: 86%, >20:1 r.r. (δ)

Synthesised following the general procedure, using cyclohexene (20.3 µL, 0.20 mmol, 1.00 equiv.), 4-*tert*-butylbenzoyl chloride (41.3 µL, 0.21 mmol, 1.05 equiv.), silver hexafluoroantimonate (75.6 mg, 0.22 mmol, 1.10 equiv.) and dichloromethane (2 mL). Purification by flash column chromatography (heptane/ethyl acetate 90:10 to 20:80, Rf = 0.41 in heptane/ethyl acetate 75:25) gave the title compound (>20:1 r.r., 44.6 mg, 86%) as a pale-yellow oil.

^{1}H NMR (600 MHz, $CDCl_3$) δ 7.89 – 7.87 (m, 2H), 7.48 – 7.42 (m, 2H), 3.78 (tt, *J* = 10.4, 4.2 Hz, 1H), 3.37 (tt, *J* = 11.0, 3.5 Hz, 1H), 2.15 – 2.11 (m, 1H), 2.04 – 1.99 (m, 1H), 1.91 – 1.84 (m, 2H), 1.60 (brs, 1H), 1.57 – 1.51 (m, 1H), 1.47 – 1.42 (m, 2H), 1.34 (s, 9H), 1.32 – 1.29 (m, 1H).

^{13}C NMR (151 MHz, $CDCl_3$): δ 202.2, 156.9, 133.5, 128.5 (2C), 125.8 (2C), 70.1, 44.0, 37.8, 35.4, 32.3, 31.2 (3C), 28.7, 23.5.

HRMS (ESI$^+$): exact mass calculated for $[M+Na]^+$ ($C_{17}H_{24}O_2Na$) requires *m/z* 283.1669, found *m/z* 283.1658.

IR (neat) ν_{max}: 3400, 2935, 2861, 1674, 1604, 1563, 1463, 1450, 1408, 1363, 1320, 1297, 1267, 1234, 1190, 1109, 1061, 1009, 955, 946, 882, 844, 804, 763, 715, 699 cm^{-1}.

8: *syn*-(3-hydroxycyclohexyl)(3-methoxyphenyl)methanone

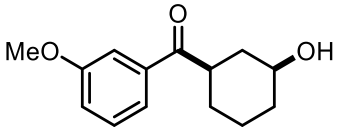

$C_{14}H_{18}O_3$
MW: 234.29
Yield: 75%, >20:1 r.r. (δ)

Synthesised following the general procedure, using cyclohexene (20.3 µL, 0.20 mmol, 1.00 equiv.), 3-methoxybenzoyl chloride (30.1 µL, 0.21 mmol, 1.05 equiv.), silver hexafluoroantimonate (75.6 mg, 0.22 mmol, 1.10 equiv.) and dichloromethane (2 mL). Purification by flash column chromatography (heptane/ethyl acetate 90:10 to 20:80, Rf = 0.39 in heptane/ethyl acetate 75:25) gave the title compound (>20:1 r.r., 35.5 mg, 75%) as a pale-yellow oil.

^{1}H NMR (700 MHz, $CDCl_3$): δ 7.51 (d, *J* = 7.6 Hz, 1H), 7.47 (s, 1H), 7.37 (t, *J* = 7.9 Hz, 1H), 7.11 (dd, *J* = 8.1, 2.4 Hz, 1H), 3.86 (s, 3H), 3.80 – 3.72 (m, 1H), 3.38 – 3.31 (m, 1H), 2.18 – 2.11 (m, 1H), 2.07 – 1.99 (m, 1H), 1.93 – 1.82 (m, 2H), 1.76 – 1.68 (brs, 1H), 1.55 – 1.50 (m, 1H), 1.47 – 1.41 (m, 2H), 1.34 – 1.27 (m, 1H).

^{13}C NMR (176 MHz, $CDCl_3$): δ 202.4, 160.1, 137.6, 129.8, 121.0, 119.5, 113.0, 70.2, 55.6, 44.2, 37.9, 35.2, 28.7, 23.8.

HRMS (ESI$^+$): exact mass calculated for $[M+Na]^+$ ($C_{14}H_{18}O_3Na$) requires *m/z* 257.1154, found *m/z* 257.1150.

IR (neat) ν_{max}: 3386, 2935, 2858, 1676, 1595, 1580, 1486, 1449, 1429, 1360, 1317, 1287, 1266, 1198, 1170, 1132, 1048, 1020, 994, 958, 872, 797, 763, 681 cm^{-1}.

9: *syn*-3-hydroxycyclohexyl(2-iodophenyl)methanone

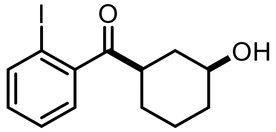

$C_{13}H_{15}IO_2$
MW: 330.16
Yield: 73%, 12:1 r.r. (δ)

Synthesised following the general procedure, using cyclohexene (20.3 µL, 0.20 mmol, 1.00 equiv.), 2-iodobenzoyl chloride (29.0 µL, 0.21 mmol, 1.05 equiv.), silver hexafluoroantimonate (75.6 mg, 0.22 mmol, 1.10 equiv.) and dichloromethane (2 mL). Purification by flash column chromatography (heptane/ethyl acetate 90:10 to 40:60, Rf = 0.35 in heptane/ethyl acetate 66:33) gave the title compound (12:1 r.r., 48.0 mg, 73%) as a colourless solid.

^{1}H NMR (700 MHz, $CDCl_3$): δ 7.88 (d, *J* = 7.9 Hz, 1H), 7.39 (t, *J* = 7.5 Hz, 1H), 7.24 (dd, *J* = 7.6, 1.7 Hz, 1H), 7.12 (td, *J* = 7.7, 1.8 Hz, 1H), 3.67 (tt, *J* = 10.7, 4.2 Hz, 1H), 3.13 – 3.08 (m, 1H), 2.24 – 2.18 (m, 1H), 2.00 (dt, *J* = 6.5, 4.3 Hz, 1H), 1.94 – 1.84 (m, 2H), 1.47 (dd, *J* = 23.0, 12.0 Hz, 1H), 1.43 – 1.32 (m, 2H), 1.29 – 1.22 (m, 1H). *The OH proton was not observed.*

^{13}C NMR (176 MHz, $CDCl_3$): δ 207.2, 145.3, 140.3, 131.4, 128.0, 127.7, 91.3, 70.2, 48.1, 37.1, 35.2, 27.6, 23.5.

HRMS (ESI$^+$): exact mass calculated for $[M+Na]^+$ ($C_{13}H_{15}IO_2Na$) requires *m/z* 353.0015, found *m/z* 353.0011.

IR (neat) ν_{max}: 3373, 2933, 2857, 1695, 1580, 1559, 1461, 1448, 1426, 1360, 1277, 1208, 1135, 1060, 1018, 1003, 953, 942, 881, 806, 763, 734 cm^{-1}.

Melting point: 68-69 °C.

10: *syn*-(3-hydroxycyclohexyl)(4-(trifluoromethyl)phenyl)methanone

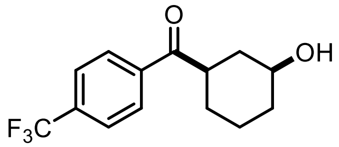

$C_{14}H_{15}F_3O_2$
MW: 272.27
Yield: 81%, 17:1 r.r. (δ)

Synthesised following the general procedure, using cyclohexene (20.3 µL, 0.20 mmol, 1.00 equiv.), 4-(trifluoromethyl)benzoyl chloride (32.3 µL, 0.21 mmol, 1.05 equiv.), silver hexafluoroantimonate (75.6 mg, 0.22 mmol, 1.10 equiv.) and dichloromethane (2 mL). Purification by flash column chromatography (heptane/ethyl acetate 50:50, Rf = 0.35 in heptane/ethyl acetate 66:33) gave the title compound (17:1 r.r., 44.1 mg, 81%) as a colourless solid, which was analysed by X-ray diffraction (see section 5).

^{1}H NMR (600 MHz, $CDCl_3$): δ 8.02 (d, *J* = 8.1 Hz, 2H), 7.73 (d, *J* = 8.2 Hz, 2H), 3.78 (tt, *J* = 10.5, 4.2 Hz, 1H), 3.35 (tt, *J* = 11.2, 3.3 Hz, 1H), 2.18 – 2.12 (m, 1H), 2.04 (d, *J* = 12.6 Hz, 1H), 1.95 – 1.84 (m, 2H), 1.80 (brs, 1H), 1.56 – 1.30 (m, 4H).

^{13}C NMR (151 MHz, $CDCl_3$): δ 201.5, 139.0, 134.5 (q, *J* = 32.8 Hz), 128.8 (2C), 125.9 (q, *J* = 3.4 Hz, 2C), 123.7 (q, *J* = 272.5 Hz), 70.1, 44.5, 37.7, 35.3, 28.5, 23.5.

^{19}F NMR (377 MHz, $CDCl_3$): δ -63.1.

HRMS (ESI$^+$): exact mass calculated for $[M+H]^+$ ($C_{14}H_{16}F_3O_2$) requires *m/z* 273.1097, found *m/z* 273.1100.

IR (neat) ν_{max}: 3297, 2939, 2861, 1684, 1451, 1409, 1374, 1326, 1265, 1233, 1213, 1169, 1129, 1112, 1068, 1015, 984, 946, 910, 856, 767, 743 cm^{-1}.

Melting point: 48-50 °C.

11: *syn*-(3,4-dichlorophenyl)(-3-hydroxycyclohexyl)methanone

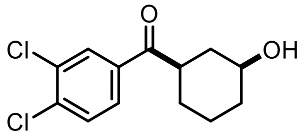

$C_{13}H_{14}Cl_2O_2$
MW: 273.15
Yield: 75%, 17:1 r.r. (δ)

Synthesised following the general procedure, using cyclohexene (20.3 µL, 0.20 mmol, 1.00 equiv.), 3,4-dichlorobenzoyl chloride (44.9 mg, 0.21 mmol, 1.05 equiv.), silver hexafluoroantimonate (75.6 mg, 0.22 mmol, 1.10 equiv.) and dichloromethane (2 mL). Purification by flash column chromatography (heptane/ethyl acetate 90:10 to 40:60, Rf = 0.33 in heptane/ethyl acetate 66:33) gave the title compound (17:1 r.r., 40.9 mg, 75%) as a colourless solid.

^{1}H NMR (400 MHz, $CDCl_3$): δ 7.99 (s, 1H), 7.75 (d, *J* = 8.3 Hz, 1H), 7.55 (d, *J* = 8.3 Hz, 1H), 3.77 – 3.75 (m, 1H), 3.28 – 3.23 (m, 1H), 2.17 – 2.08 (m, 1H), 2.08 – 1.98 (m, 1H), 1.96 – 1.80 (m, 2H), 1.68 (brs, 1H), 1.57 – 1.37 (m, 3H), 1.37 – 1.26 (m, 1H).

^{13}C NMR (101 MHz, $CDCl_3$): δ 200.2, 137.7, 135.7, 133.5, 130.9, 130.4, 127.5, 70.0, 44.2, 37.7, 35.2, 28.5, 23.6.

HRMS (ESI$^+$): exact mass calculated for $[M+Na]^+$ ($C_{13}H_{14}{}^{35}Cl_2O_2Na$) requires *m/z* 295.0269, found *m/z* 295.0249.

IR (neat) ν_{max}: 3366, 2936, 2859, 1682, 1582, 1556, 1465, 1450, 1390, 1264, 1204, 1141, 1062, 1030, 958, 888, 859, 832, 748, 674 cm^{-1}.

Melting point: 105-107 °C.

12: *syn*-(3-hydroxycyclohexyl)(mesityl)methanone

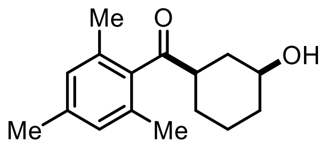

$C_{16}H_{22}O_2$
MW: 246.35
Yield: 41%, 9:1 r.r. (δ)

Synthesised following the general procedure, using cyclohexene (20.3 µL, 0.20 mmol, 1.00 equiv.), 2,4,6-trimethylbenzoyl chloride (35.0 µL, 0.21 mmol, 1.05 equiv.), silver hexafluoroantimonate (75.6 mg, 0.22 mmol, 1.10 equiv.) and dichloromethane (2 mL). Purification by flash column chromatography (heptane/ethyl acetate 90:10 to 40:60, Rf = 0.41 in heptane/ethyl acetate 66:33) gave the title compound (9:1 r.r., 20.5 mg, 41%) as a colourless oil.

^{1}H NMR (500 MHz, $CDCl_3$): δ 6.84 (s, 2H), 3.65 – 3.59 (m, 1H), 2.76 (app ddd, *J* = 11.9, 8.8, 3.1 Hz, 1H), 2.28 (s, 3H), 2.20 – 2.16 (m, 7H), 1.99 (dd, *J* = 10.6, 3.3 Hz, 1H), 1.91 – 1.80 (m, 2H), 1.66 – 1.58 (br s, 1H), 1.50 – 1.32 (m, 4H).

^{13}C NMR (126 MHz, $CDCl_3$): δ 212.2, 138.9, 138.7, 133.4 (2C), 128.8 (2C), 70.4, 50.8, 37.0, 35.3, 27.3, 23.6, 21.2, 19.8 (2C).

HRMS (ESI$^+$): exact mass calculated for $[M+Na]^+$ ($C_{16}H_{22}O_2Na$) requires *m/z* 269.1518, found *m/z* 269.1513.

IR (neat) ν_{max}: 3422, 2930, 2857, 1689, 1610, 1572, 1449, 1378, 1359, 1299, 1257, 1157, 1113, 1063, 1035, 958, 939, 879, 852, 798, 749 cm^{-1}.

13: *syn*-3-hydroxycyclohexyl(4-nitrophenyl)methanone

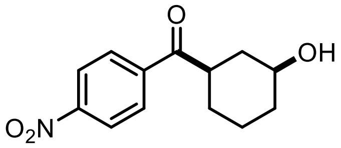

$C_{13}H_{15}NO_4$
MW: 249.27
Yield: 58%, >20:1 r.r. (δ)

Synthesised following the general procedure, using cyclohexene (20.3 µL, 0.20 mmol, 1.00 equiv.), 4-nitrobenzoyl chloride (39.0 mg, 0.21 mmol, 1.05 equiv.), silver hexafluoroantimonate (75.6 mg, 0.22 mmol, 1.10 equiv.) and dichloromethane (2 mL). Purification by flash column chromatography (heptane/ethyl acetate 90:10 to 40:60, Rf = 0.22 in heptane/ethyl acetate 66:33) gave the title compound (>20:1 r.r., 28.9 mg, 58%) as a pale-yellow oil.

^{1}H NMR (700 MHz, $CDCl_3$) δ 8.33 – 8.29 (m, 2H), 8.08 – 8.04 (m, 2H), 3.81 – 3.75 (m, 1H), 3.38 – 3.31 (m, 1H), 2.15 (app d, *J* = 12.5 Hz, 1H), 2.05 (app d, *J* = 12.5 Hz, 1H), 1.95 – 1.91 (m, 1H), 1.89 – 1.81 (m, 1H), 1.82 – 1.74 (m, 1H), 1.56 – 1.38 (m, 3H), 1.33 – 1.27 (m, 1H).

^{13}C NMR (176 MHz, $CDCl_3$) δ 200.9, 150.4, 140.8, 129.4 (2C), 124.0 (2C), 70.0, 44.8, 37.6, 35.2, 28.4, 23.5.

HRMS (ESI$^+$): exact mass calculated for $[M+Na]^+$ ($C_{13}H_{15}NO_4Na$) requires *m/z* 272.0899, found *m/z* 272.0893.

IR (neat) ν_{max}: 3359, 3109, 2935, 2859, 1685, 1603, 1524, 1450, 1406, 1346, 1318, 1260, 1205, 1105, 1061, 1011, 956, 946, 883, 860, 801, 752, 711 cm^{-1}.

14: *syn*-methyl 4-(3-hydroxycyclohexane-1-carbonyl)benzoate

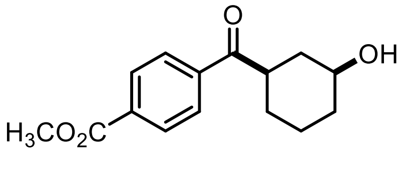

$C_{15}H_{18}O_4$
MW: 262.30
Yield: 70%,14:1 r.r. (δ)

Synthesised following the general procedure, using cyclohexene (20.3 µL, 0.20 mmol, 1.00 equiv.), methyl 4-chlorocarbonylbenzoate (43.9 mg, 0.21 mmol, 1.05 equiv.), silver hexafluoroantimonate (75.6 mg, 0.22 mmol, 1.10 equiv.) and dichloromethane (2 mL). Purification by flash column chromatography (heptane/ethyl acetate 90:10 to 40:60, Rf = 0.25 in heptane/ethyl acetate 66:33) gave the title compound (14:1 r.r., 36.8 mg, 70%) as a colourless solid.

^{1}H NMR (700 MHz, $CDCl_3$): δ 8.11 (d, *J* = 8.3 Hz, 2H), 7.96 (d, *J* = 8.3 Hz, 2H), 3.94 (s, 3H), 3.82 – 3.70 (m, 1H), 3.36 (tt, *J* = 11.3, 3.2 Hz, 1H), 2.21 – 2.09 (m, 1H), 2.08 – 1.98 (m, 1H), 1.95 – 1.81 (m, 2H), 1.81 – 1.65 (brs, 1H), 1.51 (app dd, *J* = 23.4, 11.6 Hz, 1H), 1.48 – 1.41 (m, 1H), 1.40 – 1.37 (m, 1H), 1.37 – 1.28 (m, 1H).

^{13}C NMR (176 MHz, $CDCl_3$): δ 202.0, 166.4, 139.5, 133.9, 130.1 (2C), 128.3 (2C), 70.1, 52.6, 44.5, 37.7, 35.3, 28.5, 23.5.

HRMS (ESI$^+$): exact mass calculated for $[M+Na]^+$ ($C_{15}H_{18}O_4Na$) requires *m/z* 285.1103, found *m/z* 285.1102.

IR (neat) ν_{max}: 3411, 2937, 2859, 1970, 1724, 1682, 1570, 1503, 1437, 1406, 1360, 1280, 1194, 1108, 1061, 1014, 956, 866, 831, 800, 772, 718, 695 cm^{-1}.

Melting point: 110-112 °C.

15: *syn*-(3-hydroxycyclohexyl)-2,2-dimethylpropan-1-one

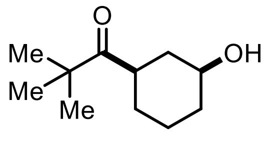

$C_{11}H_{20}O_2$

MW: 184.28

Yield: 86%, >20:1 r.r. (δ)

Synthesised following the general procedure, using cyclohexene (20.3 µL, 0.20 mmol, 1.00 equiv.), pivaloyl chloride (25.9 µL, 0.21 mmol, 1.05 equiv.), silver hexafluoroantimonate (75.6 mg, 0.22 mmol, 1.10 equiv.) and dichloromethane (2 mL). Purification by flash column chromatography (heptane/ethyl acetate 80:20 to 50:50, Rf = 0.33 in heptane/ethyl acetate 66:33) gave the title compound (>20:1 r.r., 31.6 mg, 86%) as a colourless solid.

^{1}H NMR (500 MHz, $CDCl_3$): δ 3.62 (app ddd, *J* = 14.3, 10.2, 3.9 Hz, 1H), 2.98 – 2.84 (m, 1H), 2.02 – 1.93 (m, 1H), 1.92 – 1.79 (m, 2H), 1.63 – 1.53 (m, 2H, overlaps with water peak), 1.46 – 1.23 (m, 4H), 1.14 (s, 9H).

^{13}C NMR (126 MHz, $CDCl_3$): δ 217.5, 70.2, 44.9, 43.1, 38.6, 35.3, 29.0, 26.1 (3C), 23.8.

HRMS (ESI$^+$): exact mass calculated for $[M+Na]^+$ ($C_{11}H_{20}O_2Na$) requires *m/z* 207.1361, found *m/z* 207.1355.

IR (neat) ν_{max}: 3373, 2934, 2860, 1698, 1477, 1464, 1451, 1394, 1364, 1322, 1276, 1261, 1224, 1192, 1140, 1010, 1068, 1004, 955, 884, 859, 834, 789, 750 cm^{-1}.

Melting point: 80-82 °C.

16: *syn*-benzo[*d*][1,3]dioxol-5-yl-(3-hydroxycyclohexyl)methanone

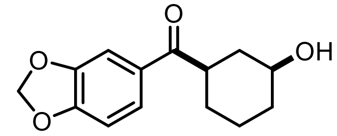

$C_{14}H_{16}O_4$
MW: 248.29
Yield: 58%, 17:1 r.r. (δ)

Synthesised following the general procedure, using cyclohexene (20.3 µL, 0.20 mmol, 1.00 equiv.), piperonyloyl chloride (38.8 mg, 0.21 mmol, 1.05 equiv.), silver hexafluoroantimonate (75.6 mg, 0.22 mmol, 1.10 equiv.) and dichloromethane (2 mL). Purification by flash column chromatography (heptane/ethyl acetate 90:10 to 50:50, Rf = 0.32 in heptane/ethyl acetate 66:33) gave the title compound (17:1 r.r., 28.9 mg, 58%) as a colourless solid.

^{1}H NMR (600 MHz, $CDCl_3$): δ 7.54 (d, *J* = 8.2 Hz, 1H), 7.41 (s, 1H), 6.85 (d, *J* = 8.1 Hz, 1H), 6.04 (s, 2H), 3.81 – 3.63 (m, 1H), 3.34 – 3.26 (m, 1H), 2.10 (d, J = 12.5 Hz, 1H), 2.02 (s, 1H), 1.92 – 1.80 (m, 3H), 1.52 (dd, *J* = 23.0, 11.5 Hz, 1H), 1.43 (t, *J* = 10.3 Hz, 2H), 1.29 (dd, *J* = 15.7, 6.9 Hz, 1H).

^{13}C NMR (151 MHz, $CDCl_3$): δ 200.6, 151.9, 148.5, 130.9, 124.5, 108.4, 108.1, 102.0, 70.1, 43.9, 38.0, 35.4, 28.8, 23.5.

HRMS (ESI$^+$): exact mass calculated for $[M+Na]^+$ ($C_{14}H_{16}O_4Na$) requires *m/z* 271.0947, found *m/z* 271.0947.

IR (neat) ν_{max}: 3411, 2934, 2858, 1667, 1603, 1503, 1488, 1441, 1351, 1256, 1145, 1103, 1037, 957, 932, 879, 814, 781, 746, 649 cm^{-1}.

Melting point: 103-105 °C.

17: *syn*-(3-hydroxycyclohexyl)(naphthalen-2-yl)methanone

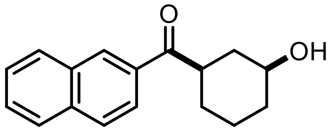

$C_{17}H_{18}O_2$
MW: 254.33
Yield: 80%, >20:1 r.r. (δ)

Synthesised following the general procedure, using cyclohexene (20.3 µL, 0.20 mmol, 1.00 equiv.), 2-naphthoyl chloride (19.1 µL, 0.21 mmol, 1.05 equiv.), silver hexafluoroantimonate (75.6 mg, 0.22 mmol, 1.10 equiv.) and dichloromethane (2 mL). Purification by flash column chromatography (heptane/ethyl acetate 90:10 to 20:80, Rf = 0.29 in heptane/ethyl acetate 75:25) gave the title compound (>20:1 r.r., 41.1 mg, 80%) as a pale-yellow oil.

^{1}H NMR (700 MHz, $CDCl_3$): δ 8.44 (s, 1H), 8.00 (d, *J* = 8.6 Hz, 1H), 7.96 (d, *J* = 8.1 Hz, 1H), 7.88 (dd, *J* = 17.0, 8.3 Hz, 2H), 7.60 (t, *J* = 7.4 Hz, 1H), 7.55 (t, *J* = 7.4 Hz, 1H), 3.86 – 3.80 (m, 1H), 3.54 (m, 1H), 2.21 (app d, *J* = 12.6 Hz, 1H), 2.06 (app d, *J* = 12.2 Hz, 1H), 1.92 (app d, *J* = 8.4 Hz, 2H), 1.60 (m, 1H), 1.54 – 1.44 (m, 2H), 1.33–1.31 (m, 1H). *The OH proton was not observed.*

^{13}C NMR (176 MHz, $CDCl_3$): δ 202.5, 135.7, 133.4, 132.7, 129.9, 129.7, 128.7, 128.6, 127.9, 126.9, 124.4, 70.2, 44.2, 38.0, 35.4, 28.8, 23.6.

HRMS (ESI$^+$): exact mass calculated for $[M+Na]^+$ ($C_{17}H_{18}O_2Na$) requires *m/z* 277.1205, found *m/z* 277.1200.

IR (neat) ν_{max}: 3406, 3058, 2929, 2856, 1672, 1626, 1595, 1576, 1506, 1465, 1449, 1360, 1275, 1213, 1178, 1121, 1060, 962, 938, 898, 862, 823, 763 cm^{-1}.

18: *syn*-(3-hydroxycyclopentyl)(3-methoxyphenyl)methanone

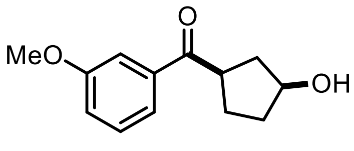

$C_{13}H_{16}O_3$
MW: 220.29
Yield: 77%

Synthesised following the general procedure, using cyclopentene (17.7 µL, 0.20 mmol, 1.00 equiv.), 3-methoxybenzoyl chloride (30.1 µL, 0.21 mmol, 1.05 equiv.), silver hexafluoroantimonate (75.6 mg, 0.22 mmol, 1.10 equiv.) and dichloromethane (2 mL). Purification by flash column chromatography (heptane/ethyl acetate 80:20 to 50:50, Rf = 0.14 in heptane/ethyl acetate 75:25) gave the title compound (34.1 mg, 77%) as a pale-yellow oil.

^{1}H NMR (400 MHz, $CDCl_3$): δ 7.55 (d, *J* = 7.7 Hz, 1H), 7.53 – 7.48 (m, 1H), 7.38 (t, *J* = 7.9 Hz, 1H), 7.12 (dd, *J* = 8.0, 2.3 Hz, 1H), 4.43 – 4.32 (m, 1H), 3.94 – 3.87 (m, 1H), 3.85 (s, 3H), 3.09 (s, 1H), 2.18 – 2.04 (m, 3H), 2.03 – 1.92 (m, 1H), 1.88 – 1.78 (m, 2H).

^{13}C NMR (101 MHz, $CDCl_3$): δ 204.7, 160.0, 137.6, 129.8, 121.4, 119.9, 113.1, 73.9, 55.6, 44.6, 38.3, 36.4, 29.1.

HRMS (ESI$^+$): exact mass calculated for $[M+Na]^+$ ($C_{13}H_{16}O_3Na$) requires *m/z* 243.0992, found *m/z* 243.0988.

IR (neat) ν_{max}: 3404, 2942, 2837, 1677, 1595, 1581, 1486, 1464, 1450, 1430, 1349, 1316, 1286, 1257, 1200, 1170, 1082, 1045, 994, 960, 878, 797 cm^{-1}.

19: *syn*-(3-hydroxycyclohexyl)undecan-1-one

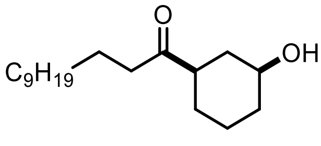

$C_{18}H_{34}O_2$
MW: 282.47
Yield: 70%, >20:1 r.r. (δ)

Synthesised following the general procedure, using cyclohexene (20.3 µL, 0.20 mmol, 1.00 equiv.), lauroyl chloride (50.6 µL, 0.21 mmol, 1.05 equiv.), silver hexafluoroantimonate (75.6 mg, 0.22 mmol, 1.10 equiv.) and dichloromethane (2 mL). Purification by flash column chromatography (heptane/ethyl acetate 80:20 to 50:50, Rf = 0.32 in heptane/ethyl acetate 66:33) gave the title compound (>20:1 r.r., 39.7 mg, 70%) as a colourless solid.

^{1}H NMR (600 MHz, $CDCl_3$): δ 3.64 (tt, *J* = 10.4, 4.2 Hz, 1H), 2.46 – 2.41 (m, 3H), 2.14 – 2.03 (m, 1H), 2.00 – 1.90 (m, 1H), 1.87 – 1.76 (m, 2H), 1.59 – 1.50 (m, 1H, overlaps with water peak), 1.40 – 1.16 (m, 22H), 0.88 (t, *J* = 7.0 Hz, 3H).

^{13}C NMR (151 MHz, $CDCl_3$): δ 213.2, 70.1, 49.3, 40.9, 37.1, 35.3, 32.1, 29.8 (2C), 29.7, 29.6, 29.5, 29.4, 27.6, 23.9, 23.4, 22.9, 14.3.

HRMS (ESI$^+$): exact mass calculated for $[M+Na]^+$ ($C_{18}H_{34}O_2Na$) requires *m/z* 305.2457, found *m/z* 305.2449.

IR (neat) ν_{max}: 3432, 2923, 2852, 1730, 1449, 1378, 1311, 1275, 1246, 1169, 1132, 1035, 982, 908, 892, 746, 722, 694 cm^{-1}.

Melting point: 52-54 °C.

20: *syn*-1-(3-hydroxycyclohexyl)-3-methylbutan-1-one

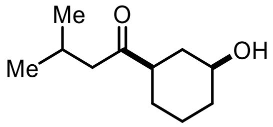

$C_{11}H_{20}O_2$
MW: 184.28
Yield: 86%, >20:1 r.r. (δ)

Synthesised following the general procedure, using cyclohexene (24.8 µL, 0.20 mmol, 1.00 equiv.), isovaleryl chloride (26.9 µL, 0.21 mmol, 1.05 equiv.), silver hexafluoroantimonate (75.6 mg, 0.22 mmol, 1.10 equiv.) and dichloromethane (2 mL). Purification by flash column chromatography (heptane/ethyl acetate 80:20 to 50:50, Rf = 0.35 in heptane/ethyl acetate 66:33) gave the title compound (>20:1 r.r., 31.7 mg, 86%) as a colourless oil.

^{1}H NMR (400 MHz, $CDCl_3$): δ 3.63 (td, *J* = 10.1, 5.0 Hz, 1H), 2.39 (tt, *J* = 11.3, 3.5 Hz, 1H), 2.31 (d, *J* = 6.9 Hz, 2H), 2.14 (td, *J* = 13.4, 6.7 Hz, 1H), 2.06 (dd, *J* = 11.0, 4.9 Hz, 1H), 2.00 – 1.90 (m, 2H), 1.87 – 1.73 (m, 2H), 1.38 – 1.17 (m, 4H), 0.89 (d, *J* = 6.7 Hz, 6H).

^{13}C NMR (151 MHz, $CDCl_3$): δ 212.8, 70.0, 49.9, 49.6, 36.9, 35.2, 27.4, 24.5, 23.3, 22.7, 22.7.

HRMS (ESI$^+$): exact mass calculated for $[M+Na]^+$ ($C_{11}H_{20}O_2Na$) requires *m/z* 207.1356, found *m/z* 207.1372.

IR (neat) ν_{max}: 2961, 2936, 1717, 1688, 1450, 1315, 1274, 1112, 1095, 1003, 762, 713, 701 cm^{-1}.

21: *syn*-1-(3-hydroxycyclopentyl)-2-methylpropan-1-one

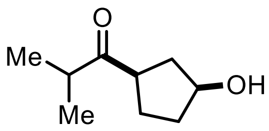

$C_9H_{16}O_2$
MW: 156.23
Yield: 46%

Synthesised following the general procedure, using cyclopentene (17.7 µL, 0.20 mmol, 1.00 equiv.), isobutyryl chloride (22.0 µL, 0.21 mmol, 1.05 equiv.), silver hexafluoroantimonate (75.6 mg, 0.22 mmol, 1.10 equiv.) and dichloromethane (2 mL). Purification by flash column chromatography (heptane/ethyl acetate 80:20 to 50:50, Rf = 0.34 in heptane/ethyl acetate 75:25) gave the title compound (14.3 mg, 46%) as a pale-yellow oil.

^{1}H NMR (600 MHz, $CDCl_3$): δ 4.28 (d, J = 2.6 Hz, 1H), 3.25 (tdd, J = 9.4, 5.8, 3.6 Hz, 1H), 3.11 (d, J = 7.8 Hz, 1H), 2.74 (hept, J = 6.9 Hz, 1H), 2.04 – 1.88 (m, 2H), 1.87 – 1.78 (m, 3H), 1.78 – 1.65 (m, 1H), 1.12 (dd, J = 6.9, 3.5 Hz, 6H).

^{13}C NMR (176 MHz, $CDCl_3$): δ 220.3, 73.8, 46.9, 40.8, 38.2, 36.5, 28.4, 18.4, 18.2.

HRMS (ESI$^+$): exact mass calculated for $[M+Na]^+$ ($C_9H_{16}O_2Na$) requires *m/z* 179.1043, found *m/z* 179.1037.

IR (neat) ν_{max}: 3404, 2966, 2936, 2874, 1701, 1467, 1383, 1364, 1291, 1207, 1179, 1075, 1051, 960, 739 cm^{-1}.

22: ***syn*-4-chloro-1-(3-hydroxycyclohexyl)butan-1-one**

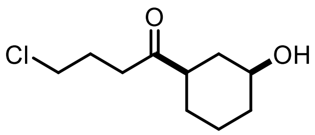

$C_{10}H_{17}ClO_2$
MW: 204.69
Yield: 91%, 14:1 r.r. (δ)

Synthesised following the general procedure, using cyclohexene (20.3 µL, 0.20 mmol, 1.00 equiv.), 4-chlorobutyryl chloride (23.7 µL, 0.21 mmol, 1.05 equiv.), silver hexafluoroantimonate (75.6 mg, 0.22 mmol, 1.10 equiv.) and dichloromethane (2 mL). Purification by flash column chromatography (heptane/ethyl acetate 80:20 to 50:50, Rf = 0.14 in heptane/ethyl acetate 66:33) gave the title compound (14:1 r.r., 37.3 mg, 91%) as a pale-yellow oil.

^{1}H NMR (400 MHz, CDCl$_3$): *δ* 3.68 – 3.60 (m, 1H), 3.56 (t, *J* = 6.2 Hz, 2H), 2.70 – 2.60 (m, 2H), 2.44 (tt, *J* = 11.4, 3.5 Hz, 1H), 2.12 – 2.07 (m, 1H), 2.07 – 1.99 (m, 2H), 1.96 (dd, *J* = 9.0, 3.6 Hz, 1H), 1.89 – 1.78 (m, 3H), 1.41 – 1.15 (m, 4H).

^{13}C NMR (101 MHz, CDCl$_3$): *δ* 211.7, 70.0, 49.5, 44.6, 37.3, 37.0, 35.1, 27.6, 26.3, 23.4.

HRMS (ESI$^+$): exact mass calculated for [M+Na]$^+$ ($C_{10}H_{17}{}^{35}ClO_2Na$) requires *m/z* 227.0809, found *m/z* 227.0806.

IR (neat) ν$_{max}$: 3398, 2934, 2858, 1703, 1466, 1449, 1408, 1378, 1361, 1319, 1293, 1270, 1251, 1230, .1199, 1142, 1063, 1046, 956, 929, 906, 889, 872, 847, 830 cm^{-1}.

23: ***syn*-2-chloro-1-(3-hydroxycyclohexyl)propan-1-one**

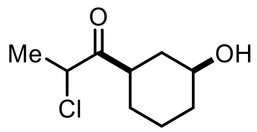

$C_9H_{15}ClO_2$
MW: 190.67
Yield: 65%, >20:1 r.r. (δ)

Synthesised following the general procedure, using cyclohexene (20.3 µL, 0.20 mmol, 1.00 equiv.), 2-chloropropionyl chloride (20.5 µL, 0.21 mmol, 1.05 equiv.), silver hexafluoroantimonate (75.6 mg, 0.22 mmol, 1.10 equiv.) and dichloromethane (2 mL). Purification by flash column chromatography (heptane/ethyl acetate 80:20 to 50:50, Rf = 0.25 in heptane/ethyl acetate 66:33) gave the title compound (>20:1 r.r., 24.8 mg, 65%) as a pale-yellow oil.

^{1}H NMR (400 MHz, CDCl$_3$): *δ* 4.47 (q, *J* = 6.8 Hz, 1H), 4.00 (dd, *J* = 6.0, 3.8 Hz, 1H), 2.89 (tt, *J* = 10.2, 3.6 Hz, 1H), 2.01 – 1.87 (m, 1H), 1.87 – 1.71 (m, 3H), 1.71 – 1.61 (m, 4H), 1.61 – 1.58 (m, 4H).

^{13}C NMR (101 MHz, CDCl$_3$): *δ* 207.4, 66.1, 56.6, 45.7, 32.1, 31.9, 23.5, 22.9, 20.1.

HRMS (ESI$^+$): exact mass calculated for $[M+Na]^+$ ($C_9H_{15}O_2ClNa$) requires *m/z* 213.0653, found *m/z* 213.0655.

IR (neat) ν_{max}: 3401, 2931, 2866, 1663, 1636, 1559, 1521, 1497, 1465, 1396, 1198, 978, 871, 705 cm^{-1}.

24: *syn*-1-(3-hydroxycyclohexyl)-2-(4-(trifluoromethyl)phenyl)ethan-1-one

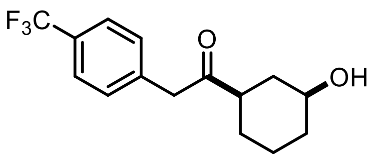

$C_{15}H_{17}F_3O_2$
MW: 286.29
Yield: 59%, 10:1 r.r. (δ)

Synthesised following the general procedure, using cyclohexene (20.3 µL, 0.20 mmol, 1.00 equiv.), **S2** (46.7 mg, 0.21 mmol, 1.05 equiv.), silver hexafluoroantimonate (75.6 mg, 0.22 mmol, 1.10 equiv.) and dichloromethane (2 mL). Purification by flash column chromatography (heptane/ethyl acetate 60:40 to 50:50, Rf = 0.26 in heptane/ethyl acetate 50:50) gave the title compound (10:1 r.r., 33.9 mg, 59%) as a colourless solid.

^{1}H NMR (400 MHz, CDCl$_3$): *δ* 7.58 (d, *J* = 8.1 Hz, 2H), 7.29 (d, *J* = 8.0 Hz, 2H), 3.82 (s, 2H), 3.71 – 3.54 (m, 1H), 2.54 (tt, *J* = 11.4, 3.6 Hz, 1H), 2.17 – 2.05 (m, 1H), 2.01 – 1.92 (m, 1H), 1.90 – 1.60 (m, 3H), 1.44 – 1.16 (m, 4H).

^{13}C NMR (101 MHz, CDCl$_3$): *δ* 208.9, 138.2, 130.0 (2C, $^{2}J_{C\text{–}F}$ coupling not visible), 129.5 (q, *J* = 32.3 Hz, 2C), 125.7 (q, *J* = 3.8 Hz), 122.9, 69.9, 49.2, 47.3, 37.0, 35.1, 27.6, 23.3.

^{19}F NMR (377 MHz, CDCl$_3$): *δ* -62.53.

HRMS (ESI$^+$): exact mass calculated for $[M+H]^+$ ($C_{15}H_{18}F_3O_2$) requires *m/z* 287.1253, found *m/z* 287.1245.

IR (neat) ν_{max}: 3324, 2904, 2859, 1711, 1667, 1617, 1467, 1451, 1419, 1372, 1359, 1322, 1269, 1252, 1232, 1190, 1123, 1107, 1065, 1049,998, 963, 916, 852, 833, 815, 786 cm^{-1}.

Melting point: 58-60 °C.

25: *syn*-((*E*)-3-hydroxycyclohexyl)but-2-en-1-one

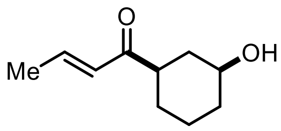

$C_{10}H_{16}O_2$
MW: 168.24
Yield: 54%, >20:1 r.r. (δ)

Synthesised following the general procedure, using cyclohexene (20.3 µL, 0.20 mmol, 1.00 equiv.), (*E*)-2-butenoyl chloride (20.1 µL, 0.21 mmol, 1.05 equiv.), silver hexafluoroantimonate (75.6 mg, 0.22 mmol, 1.10 equiv.) and dichloromethane (2 mL). Purification by flash column chromatography (heptane/ethyl acetate 80:20 to 50:50, Rf = 0.20 in heptane/ethyl acetate 75:25) gave the title compound (>20:1 r.r., 18.2 mg, 54%) as a pale-yellow oil.

^{1}H NMR (400 MHz, $CDCl_3$): *δ* 6.91 (dq, *J* = 13.7, 6.9 Hz, 1H), 6.19 (dd, *J* = 15.6, 1.6 Hz, 1H), 3.67 (ddd, *J* = 14.2, 10.0, 4.1 Hz, 1H), 2.65 (tt, *J* = 11.0, 3.5 Hz, 1H), 2.10 – 2.01 (m, 1H), 2.00 – 1.93 (m, 1H), 1.90 (dd, *J* = 6.9, 1.5 Hz, 3H), 1.88 – 1.81 (m, 2H), 1.81 – 1.72 (m, 1H), 1.45 – 1.19 (m, 4H).

^{13}C NMR (126 MHz, $CDCl_3$): *δ* 202.0, 143.2, 130.1, 70.0, 47.0, 37.1, 35.2, 27.8, 23.2, 18.4.

HRMS (ESI$^+$): exact mass calculated for $[M+H]^+$ ($C_{10}H_{17}O_2$) requires *m/z* 169.1223, found *m/z* 169.1219.

IR (neat) ν_{max}: 3408, 2934, 2858, 2360, 1688, 1662, 1626, 1444, 1361, 1294, 1199, 1150, 1058, 969, 934, 889, 869, 844, 787 cm^{-1}.

26: *syn*-furan-3-yl-(3-hydroxycyclohexyl)methanone

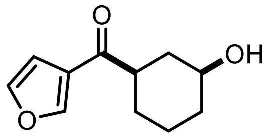

$C_{11}H_{14}O_3$
MW: 194.23
Yield: 83%,17:1 r.r. (δ)

Synthesised following the general procedure, using cyclohexene (20.3 µL, 0.20 mmol, 1.00 equiv.), 3-furoyl chloride (20.7 µL, 0.21 mmol, 1.05 equiv.), silver hexafluoroantimonate (75.6 mg, 0.22 mmol, 1.10 equiv.) and dichloromethane (2 mL). Purification by flash column chromatography (heptane/ethyl acetate 80:20 to 50:50, Rf = 0.31 in heptane/ethyl acetate 50:50) gave the title compound (17:1 r.r., 31.7 mg, 83%) as a colourless solid.

^{1}H NMR (400 MHz, CDCl$_3$): *δ* 8.11 – 7.93 (m, 1H), 7.49 – 7.36 (m, 1H), 6.75 (dd, *J* = 1.9, 0.8 Hz, 1H), 3.71 (tt, *J* = 10.3, 4.2 Hz, 1H), 2.92 (ddd, *J* = 14.6, 7.3, 3.6 Hz, 1H), 2.19 – 2.05 (m, 2H), 2.05 – 1.93 (m, 1H), 1.92 – 1.79 (m, 2H), 1.53 (ddd, *J* = 12.6, 11.4, 10.7 Hz, 1H), 1.47 – 1.35 (m, 2H), 1.35 – 1.22 (m, 1H).

^{13}C NMR (151 MHz, CDCl$_3$): *δ* 197.5, 147.3, 144.4, 126.7, 109.1, 69.8, 47.0, 37.5, 35.2, 28.6, 23.3.

HRMS (ESI$^+$): exact mass calculated for $[M+H]^+$ ($C_{11}H_{15}O_3$) requires *m/z* 195.1016, found *m/z* 195.1012.

IR (neat) ν_{max}: 3410, 3136, 2936, 2860, 1715, 1668, 1561, 1511, 1450, 1396, 1364, 1326, 1284, 1158, 1066, 926, 873, 772 cm^{-1}.

Melting point: 85-87 °C.

27: *syn*-benzo[*b*]thiophen-2-yl-(3-hydroxycyclohexyl)methanone

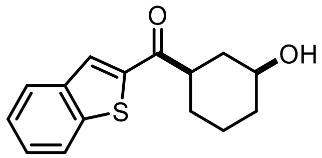

$C_{15}H_{16}O_2S$
MW: 260.35
Yield: 61%, 13:1 r.r. (δ)

Synthesised following the general procedure, using cyclohexene (20.3 µL, 0.20 mmol, 1.00 equiv.), benzo[*b*]thiophene-2-carbonyl chloride (42.1 mg, 0.21 mmol, 1.05 equiv.), silver hexafluoroantimonate (75.6 mg, 0.22 mmol, 1.10 equiv.) and dichloromethane (2 mL). Purification by flash column chromatography (heptane/ethyl acetate 80:20 to 50:50, Rf = 0.32 in heptane/ethyl acetate 50:50) gave the title compound (13:1 r.r., 31.9 mg, 61%) as a colourless solid.

^{1}H NMR (600 MHz, $CDCl_3$): *δ* 7.97 (s, 1H), 7.91 – 7.83 (m, 2H), 7.49 – 7.44 (m, 1H), 7.44 – 7.39 (m, 1H), 3.84 – 3.68 (m, 1H), 3.35 (tt, *J* = 11.3, 3.2 Hz, 1H), 2.26 – 2.16 (m, 1H), 2.10 – 2.00 (m, 1H), 1.94 (m, 2H), 1.77 (app brs, 1H), 1.67 – 1.56 (m, 1H, overlaps with water peak), 1.55 – 1.44 (m, 2H), 1.37 – 1.27 (m, 1H).

^{13}C NMR (151 MHz, $CDCl_3$): *δ* 196.8, 143.0, 142.8, 139.3, 129.0, 127.7, 126.1, 125.3, 123.2, 70.1, 45.8, 38.1, 35.3, 29.0, 23.6.

HRMS (ESI$^+$): exact mass calculated for $[M+Na]^+$ ($C_{15}H_{16}O_2SNa$) requires *m/z* 283.0769, found *m/z* 283.0765.

IR (neat) ν_{max}: 3394, 3057, 2935, 2858, 1656, 1593, 1557, 1514, 1450, 1428, 1359, 1333, 1274, 1260, 1214, 1170, 1130, 1060, 980, 942, 879, 842, 751, 722 cm^{-1}.

Melting point: 101-103 °C.

28: ((1*S,2*S**,4*S**,7*R**)-2-hydroxybicyclo[2.2.1]heptan-7-yl)(phenyl)methanone**

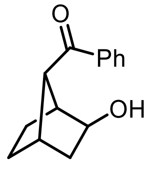

$C_{14}H_{16}O_2$
MW: 216.28
Yield: 60%
>20:1 d.r.

Synthesised following the general procedure, using norbornene (18.8 mg, 0.20 mmol, 1.00 equiv.), benzoyl chloride (24.4 µl, 0.21 mmol, 1.05 equiv.), silver hexafluoroantimonate (75.6 mg, 0.22 mmol, 1.10 equiv.) and dichloromethane (2 ml). Purification by flash column chromatography (heptane/ethyl acetate 80:20 to 50:50, Rf = 0.36 in heptane/ethyl acetate 66:33) gave the title compound (>20:1 d.r., 26.0 mg, 60%) as a colourless solid.

^{1}H NMR (400 MHz, $CDCl_3$): δ 8.00 (d, *J* = 7.5 Hz, 2H), 7.58 (t, *J* = 7.4 Hz, 1H), 7.47 (t, *J* = 7.7 Hz, 2H), 4.50 (d, *J* = 10.3 Hz, 1H), 3.82 – 3.71 (m, 1H), 3.40 (s, 1H), 2.69 (t, *J* = 4.0 Hz, 1H), 2.62 (d, *J* = 4.4 Hz, 1H), 1.90 (dd, *J* = 13.6, 7.5 Hz, 1H), 1.86 – 1.76 (m, 1H), 1.74 – 1.64 (m, 1H), 1.55 (ddd, *J* = 13.5, 7.2, 3.6 Hz, 1H), 1.37 – 1.21 (m, 2H).

^{13}C NMR (101 MHz, $CDCl_3$): δ 204.0, 137.0, 133.5, 128.8 (2C), 128.7 (2C), 75.3, 57.6, 47.3, 42.8, 41.9, 28.8, 26.4.

HRMS (ESI$^+$): exact mass calculated for $[M+Na]^+$ ($C_{14}H_{16}O_2Na$) requires *m/z* 239.1043, found *m/z* 239.1041.

IR (neat) ν_{max}: 3427, 2969, 2959, 2873, 1752, 1722, 1680, 1660, 1598, 1579, 1447, 1382, 1328, 1294, 1267, 1230, 1180, 1093, 1014, 1000, 769, 747, 693, 669 cm^{-1}.

Melting point: 100-102 °C.

Additional scope entries:

55: 4-hydroxy-1-phenylnonan-1-one

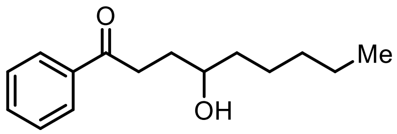

$C_{15}H_{22}O_2$
MW: 234.34
Yield: 79%, >20:1 r.r. (δ)

Synthesised following the general procedure, using 1-octene (31.4 µL, 0.20 mmol, 1.00 equiv.), benzoyl chloride (24.4 µL, 0.21 mmol, 1.05 equiv.), silver hexafluoroantimonate (75.6 mg, 0.22 mmol, 1.10 equiv.) and dichloromethane (2 mL). Purification by flash column chromatography (heptane/ethyl acetate 90:10 to 20:80, Rf = 0.59 in heptane/ethyl acetate 66:33) gave the title compound (>20:1 r.r., 36.8 mg, 79%) as a colourless solid.

^{1}H NMR (600 MHz, CDCl$_3$) δ 8.00 – 7.97 (m, 2H), 7.58 – 7.54 (m, 1H), 7.46(dd, *J* = 16.7, 9.0 Hz, 2H), 3.69 – 3.66 (m, 1H), 3.23 – 3.09 (m, 2H), 2.02 – 1.96 (m, 1H), 1.85 – 1.78 (m, 1H), 1.74 (brs, 1H), 1.51 – 1.45 (m, 3H), 1.36 – 1.29 (m, 5H), 0.89 (t, *J* = 6.7 Hz, 3H).

^{13}C NMR (101 MHz, CDCl$_3$) δ 201.0, 137.1, 133.2, 128.7 (2C), 128.3 (2C), 71.7, 38.0, 35.1, 32.0, 31.5, 25.5, 22.8, 14.2.

HRMS (ESI$^+$): exact mass calculated for $[M+Na]^+$ ($C_{15}H_{22}O_2Na$) requires *m/z* 257.1512, found *m/z* 257.1506.

IR (neat) ν_{max}: 2960, 2929, 1716, 1689, 1457, 1312, 1270, 1111, 1071, 991, 798, 774 cm^{-1}.

Melting point: 48-51 °C.

56: 4-hydroxy-1-phenyldodecan-1-one

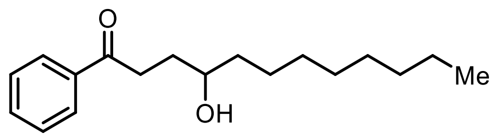

$C_{18}H_{28}O_2$
MW: 276.42
Yield: 83%, >20:1 r.r. (δ)

Synthesised following the general procedure, using 1-undecene (41.1 µL, 0.20 mmol, 1.00 equiv.), benzoyl chloride (24.4 µL, 0.21 mmol, 1.05 equiv.), silver hexafluoroantimonate (75.6 mg, 0.22 mmol, 1.10 equiv.) and dichloromethane (2 mL). Purification by flash column chromatography (heptane/ethyl acetate 90:10 to 20:80, Rf = 0.45 in heptane/ethyl acetate 75:25) gave the title compound (>20:1 r.r., 45.6 mg, 83%) as a pale-yellow oil.

^{1}H NMR (600 MHz, $CDCl_3$) δ 7.99 (dd, *J* = 8.2, 1.0 Hz, 2H), 7.58 – 7.54 (m, 1H), 7.46 (dd, *J* = 17.0, 9.3 Hz, 2H), 3.67 (app s, 1H), 3.22 – 3.09 (m, 2H), 1.99 (dtd, *J* = 10.8, 7.3, 3.4 Hz, 1H), 1.85 – 1.76 (m, 1H), 1.74 (brs, 1H), 1.51 – 1.43 (m, 3H), 1.37 – 1.27 (m, 11H), 0.88 (t, *J* = 7.0 Hz, 3H).

^{13}C NMR (151 MHz, $CDCl_3$) δ 201.0, 137.1, 133.2, 128.7 (2C), 128.3 (2C), 71.7, 38.1, 35.1, 32.0, 31.5, 29.8, 29.7, 29.4, 25.8, 22.8, 14.3.

HRMS (ESI$^+$): exact mass calculated for $[M+Na]^+$ ($C_{18}H_{28}O_2Na$) requires *m/z* 299.1982, found *m/z* 299.1975.

IR (neat) ν_{max}: 2960, 2931, 1713, 1688, 1469, 1315, 1214, 1112, 1069, 931, 745, 667, 554 cm^{-1}.

57: *syn*-(3-hydroxycyclohexyl)(4-methoxyphenyl)methanone

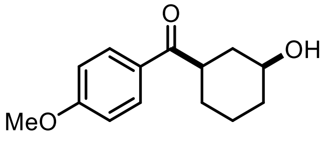

$C_{14}H_{18}O_3$
MW: 234.29
Yield: 55%, >20:1 r.r. (δ)

Synthesised following the general procedure, using cyclohexene (20.3 µL, 0.20 mmol, 1.00 equiv.), 4-methoxybenzoyl chloride (28.4 µL, 0.21 mmol, 1.05 equiv.), silver hexafluoroantimonate (75.6 mg, 0.22 mmol, 1.10 equiv.) and dichloromethane (2 mL). Purification by flash column chromatography (heptane/ethyl acetate 90:10 to 20:80, Rf = 0.37 in heptane/ethyl acetate 75:25) gave the title compound (>20:1 r.r., 26.1 mg, 55%) as a colourless solid.

^{1}H NMR (700 MHz, $CDCl_3$): δ 7.92 (d, *J* = 8.0 Hz, 2H), 6.93 (d, *J* = 8.0 Hz, 2H), 3.87 (s, 3H), 3.81 – 3.69 (m, 1H), 3.37 – 3.32 (m, 1H), 2.13 – 2.09 (m, 1H), 2.03 – 1.96 (m, 1H), 1.90 – 1.76 (m, 2H), 1.57 – 1.51 (m, 1H), 1.46 – 1.41 (m, 2H), 1.34 – 1.27 (m, 1H), 1.26 – 1.23 (m, 1H).

^{13}C NMR (176 MHz, $CDCl_3$): δ 201.1, 163.6, 130.8 (2C), 129.0, 114.0 (2C), 70.1, 55.6, 43.7, 37.9, 35.4, 28.8, 23.5.

HRMS (ESI$^+$): exact mass calculated for $[M+Na]^+$ ($C_{14}H_{18}O_3Na$) requires *m/z* 257.1154, found *m/z* 257.1147.

IR (neat) ν_{max}: 3410, 2935, 2858, 1667, 1600, 1574, 1510, 1451, 1419, 1372, 1256, 1171, 1139, 1116, 1061, 1029, 955, 882, 840, 815, 770, 753 cm^{-1}.

Melting point: 79-81 °C.

58: *syn*-(3-hydroxycyclohexyl)(*m*-tolyl)methanone

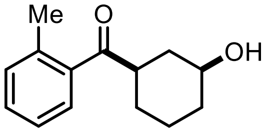

$C_{14}H_{18}O_2$
MW: 218.29
Yield: 66%, >20:1 r.r. (δ)

Synthesised following the general procedure, using cyclohexene (20.3 µL, 0.20 mmol, 1.00 equiv.), *o*-toluoyl chloride (27.4 µL, 0.21 mmol, 1.05 equiv.), silver hexafluoroantimonate (75.6 mg, 0.22 mmol, 1.10 equiv.) and dichloromethane (2 mL). Purification by flash column chromatography (heptane/ethyl acetate 90:10 to 20:80, Rf = 0.29 in heptane/ethyl acetate 66:33) gave the title compound (>20:1 r.r., 28.9 mg, 66%) as a colourless solid.

^{1}H NMR (400 MHz, $CDCl_3$) δ 7.50 – 7.48 (m, 1H), 7.39 – 7.32 (m, *J* = 7.2 Hz, 1H), 7.29 – 7.24 (m, 2H), 3.71 (app s, 1H), 3.21 – 3.09 (m, 1H), 2.42 (s, 3H), 2.13 (app d, *J* = 12.4 Hz, 1H), 2.00 (app d, *J* = 12.0 Hz, 1H), 1.91 – 1.75 (m, 3H), 1.51 – 1.22 (m, 4H).

^{13}C NMR (101 MHz, $CDCl_3$) δ 207.3, 138.5, 137.6, 131.8, 130.9, 127.4, 125.7, 70.2, 47.5, 37.4, 35.3, 27.9, 23.5, 20.8.

HRMS (ESI$^+$): exact mass calculated for $[M+Na]^+$ ($C_{14}H_{18}O_2Na$) requires *m/z* 241.1205, found *m/z* 241.1198.

IR (neat) ν_{max}: 3366, 2933, 2858, 1682, 1599, 1571,1485, 1451, 1360, 1259, 1212, 1128, 1060, 1009,953, 882, 852, 819, 760, 655 cm^{-1}.

Melting point: 64-65 °C.

59: *syn*-fluorophenyl-(3-hydroxycyclohexyl)methanone

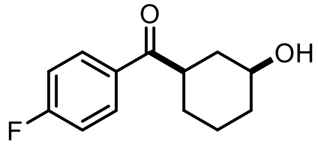

$C_{13}H_{15}FO_2$
MW: 222.26
Yield: 83%, 17:1 r.r. (δ)

Synthesised following the general procedure, using cyclohexene (20.3 µL, 0.20 mmol, 1.00 equiv.), 4-fluorobenzoyl chloride (24.8 µL, 0.21 mmol, 1.05 equiv.), silver hexafluoroantimonate (75.6 mg, 0.22 mmol, 1.10 equiv.) and dichloromethane (2 mL). Purification by flash column chromatography (heptane/ethyl acetate 90:10 to 40:60, Rf = 0.32 in heptane/ethyl acetate 50:50) gave the title compound (17:1 r.r., 37.1 mg, 83%) as a colourless solid.

^{1}H NMR (600 MHz, $CDCl_3$): δ 7.98 – 7.95 (m, 2H), 7.15 – 7.12 (m, 2H), 3.77 (app s, 1H), 3.34 –3.31 (m, 1H), 2.17 – 2.06 (m, 1H), 2.08 – 1.97 (m, 1H), 1.93 – 1.80 (m, 2H), 1.77 (brs, 1H), 1.57 – 1.46 (m, 1H), 1.49 – 1.39 (m, 2H), 1.37 – 1.19 (m, 1H).

^{13}C NMR (151 MHz, $CDCl_3$): δ 200.9, 165.9 (d, *J* = 253.5 Hz), 132.5(d, *J* = 3.0 Hz), 131.1 (d, *J* = 9.0 Hz, 2C), 115.9 (d, *J* = 22.5 Hz, 2C), 70.1, 44.1, 37.8, 35.4, 28.7, 23.5.

^{19}F (372 MHz, $CDCl_3$): δ -105.3.

HRMS (ESI$^+$): exact mass calculated for $[M+Na]^+$ ($C_{13}H_{15}FO_2Na$) requires *m/z* 245.0954, found *m/z* 245.0949.

IR (neat) ν_{max}: 3353, 2935, 2859, 1676, 1596, 1505, 1450, 1410, 1361, 1298, 1262, 1230, 1156, 1105, 1060, 1012, 955, 882, 844, 823, 777, 749 cm^{-1}.

Melting point: 103-104 °C

60: *syn*-(3-hydroxycyclohexyl)(4-(trifluoromethoxy)phenyl)methanone

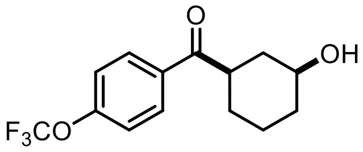

$C_{14}H_{15}F_3O_3$
MW: 288.27
Yield: 71%, 14:1 r.r. (δ)

Synthesised following the general procedure, using cyclohexene (20.3 µL, 0.20 mmol, 1.00 equiv.), 4-(trifluoromethoxy)benzoyl chloride (33.2 µL, 0.21 mmol, 1.05 equiv.), silver hexafluoroantimonate (75.6 mg, 0.22 mmol, 1.10 equiv.) and dichloromethane (2 mL). Purification by flash column chromatography (heptane/ethyl acetate 90:10 to 40:60, Rf = 0.20 in heptane/ethyl acetate 75:25) gave the title compound (14:1 r.r., 40.9 mg, 71%) as a pale-yellow oil.

^{1}H NMR (400 MHz, CDCl$_3$): *δ* 7.98 (d, *J* = 8.8 Hz, 2H), 7.28 (d, *J* = 8.4 Hz, 2H), 3.81 – 3.72 (m, 1H), 3.32 (tt, *J* = 11.2, 3.4 Hz, 1H), 2.14 – 2.08 (m, 1H), 2.06 – 1.95 (m, 2H), 1.93 – 1.76 (m, 2H), 1.58 – 1.36 (m, 3H), 1.29 (ddd, *J* = 22.7, 12.3, 3.6 Hz, 1H).

^{13}C NMR (101 MHz, CDCl$_3$): *δ* 201.0, 152.7, 134.3, 130.5 (2C), 120.9 (2C), 120.5 (q, *J* = 258.8 Hz), 70.1, 44.2, 37.7, 35.3, 28.6, 23.5.

^{19}F NMR (377 MHz, CDCl$_3$) *δ* -57.63.

HRMS (ESI$^+$): exact mass calculated for $[M+H]^+$ ($C_{14}H_{16}O_3F_3$) requires *m/z* 289.1046, found *m/z* 289.1040.

IR (neat) ν_{max}: 3361, 2937, 2862, 2361, 1681, 1602, 1505, 1451, 1413, 1362, 1254, 1207, 1165, 1113, 1062, 1014, 956, 927, 883, 859, 816, 796 cm^{-1}.

61: *syn*-(3-hydroxycyclopentyl)(phenyl)methanone

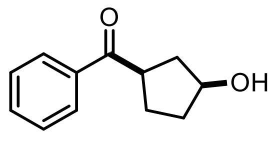

$C_{12}H_{14}O_2$
MW: 190.24
Yield: 84%

Synthesised following the general procedure, using cyclopentene (17.7 µL, 0.20 mmol, 1.00 equiv.), benzoyl chloride (24.4 µL, 0.21 mmol, 1.05 equiv.), silver hexafluoroantimonate (75.6 mg, 0.22 mmol, 1.10 equiv.) and dichloromethane (2 mL). Purification by flash column chromatography (heptane/ethyl acetate 80:20 to 50:50, Rf = 0.36 in heptane/ethyl acetate 66:33) gave the title compound (32.0 mg, 84%) as a colourless oil.

^{1}H NMR (400 MHz, $CDCl_3$): δ 8.02 – 7.96 (m, 2H), 7.58 (dd, *J* = 10.5, 4.2 Hz, 1H), 7.48 (t, *J* = 7.6 Hz, 2H), 4.42 – 4.33 (m, 1H), 3.94 (ddd, *J* = 13.3, 9.4, 6.2 Hz, 1H), 3.09 (d, *J* = 7.1 Hz, 1H), 2.18 – 2.04 (m, 3H), 2.04 – 1.93 (m, 1H), 1.88 – 1.80 (m, 2H).

^{13}C NMR (151 MHz, $CDCl_3$): δ 205.0, 136.2, 133.5, 128.9 (2C), 128.8 (2C), 73.9, 44.5, 38.2, 36.4, 29.1.

HRMS (ESI$^+$): exact mass calculated for $[M+Na]^+$ ($C_{12}H_{14}O_2Na$) requires *m/z* 213.0886, found *m/z* 213.0879.

IR (neat) ν_{max}: 3394, 2955, 2870, 1677, 1596, 1579, 1448, 1361, 1281, 1225, 1180, 1160, 1079, 1052, 1002, 993, 958, 932, 882, 842, 790, 766, 701.

62: *syn*-(4-fluorophenyl)(3-hydroxycyclopentyl)methanone

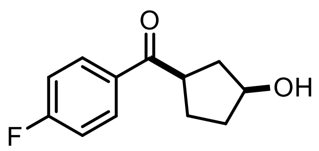

$C_{12}H_{13}FO_2$
MW: 208.23
Yield: 78%

Synthesised following the general procedure, using cyclopentene (17.7 µL, 0.20 mmol, 1.00 equiv.), 4-fluorobenzoyl chloride (24.8 µL, 0.21 mmol, 1.05 equiv.), silver hexafluoroantimonate (75.6 mg, 0.22 mmol, 1.10 equiv.) and dichloromethane (2 mL). Purification by flash column chromatography (heptane/ethyl acetate 80:20 to 50:50, Rf = 0.30 in heptane/ethyl acetate 75:25) gave the title compound (32.6 mg, 78%) as a colourless solid.

^{1}H NMR (600 MHz, $CDCl_3$): δ 8.04 – 7.99 (m, 2H), 7.17 – 7.13 (m, 2H), 4.43 – 4.26 (m, 1H), 3.88 (app ddd, J = 13.1, 9.5, 6.3 Hz, 1H), 2.99 (brs, 1H), 2.15 – 2.02 (m, 3H), 2.03 – 1.91 (m, 1H), 1.87 – 1.78 (m, 2H).

^{13}C NMR (151 MHz, $CDCl_3$): δ 203.2, 166.1 (d, J = 255.4 Hz), 132.6 (d, J = 3.0 Hz), 131.52 (d, J = 9.4 Hz, 2C), 116.0 (d, J = 21.8 Hz, 2C), 73.9, 44.5, 38.2, 36.4, 29.0.

^{19}F NMR (565 MHz, $CDCl_3$) δ -105.2.

HRMS (ESI$^+$): exact mass calculated for $[M+Na]^+$ ($C_{12}H_{13}FO_2Na$) requires *m/z* 231.0798, found *m/z* 231.0791.

IR (neat) ν_{max}: 3409, 2942, 1677, 1594, 1505, 1410, 1358, 1298, 1221, 1155, 1082, 1051, 994, 958, 846, 757, 682 cm^{-1}.

Melting point: 43-45 °C.

63: *syn*-(3-hydroxycyclohexyl)nonan-1-one

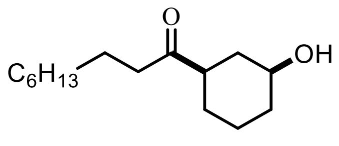

$C_{15}H_{28}O_2$
MW: 240.38
Yield: 66 %, >20:1 (δ)

Synthesised following the general procedure, using cyclohexene (20.3 µL, 0.20 mmol, 1.00 equiv.), nonanoyl chloride (37.9 µL, 0.21 mmol, 1.05 equiv.), silver hexafluoroantimonate (75.6 mg, 0.22 mmol, 1.10 equiv.) and dichloromethane (2 mL). Purification by flash column chromatography (heptane/ethyl acetate 90:10 to 50:50, Rf = 0.33 in heptane/ethyl acetate 66:33) gave the title compound (>20:1 r.r., 32.1 mg, 66%) as a colourless solid.

^{1}H NMR (600 MHz, $CDCl_3$): *δ* 3.64 (tt, *J* = 10.4, 4.2 Hz, 1H), 2.48 – 2.38 (m, 3H), 2.11 – 2.04 (m, 1H), 1.98 – 1.90 (m, 1H), 1.87 – 1.74 (m, 2H), 1.57 – 1.52 (m, 2H), 1.38 – 1.20 (m, 15H), 0.88 – 0.86 (m, 3H).

^{13}C NMR (151 MHz, $CDCl_3$): *δ* 213.2, 70.1, 49.3, 40.9, 37.0, 35.2, 32.0, 29.5, 29.4, 29.3, 27.6, 23.9, 23.3, 22.8, 14.2.

HRMS (ESI$^+$): exact mass calculated for $[M+Na]^+$ ($C_{15}H_{28}O_2Na$) requires *m/z* 263.1987, found *m/z* 263.1974.

IR (neat) ν_{max}: 3399, 2927, 2855, 1707, 1452, 1407, 1362, 1273, 1197, 1146, 1060, 963, 749 cm^{-1}.

Melting point: 139-141 °C.

64: *syn*-3-cyclopentyl-1-(-3-hydroxycyclohexyl)propan-1-one

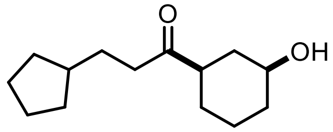

$C_{14}H_{24}O_2$
MW: 224.34
Yield: 42%, >20:1 r.r. (δ)

Synthesised following the general procedure, using cyclohexene (20.3 µL, 0.20 mmol, 1.00 equiv.), cyclopentanepropionyl chloride (32.2 µL, 0.21 mmol, 1.05 equiv.), silver hexafluoroantimonate (75.6 mg, 0.22 mmol, 1.10 equiv.) and dichloromethane (2 mL). Purification by flash column chromatography (heptane/ethyl acetate 80:20 to 50:50, Rf = 0.32 in heptane/ethyl acetate 66:33) gave the title compound (>20:1 r.r., 19.1 mg, 42%) as a pale-yellow oil.

^{1}H NMR (400 MHz, $CDCl_3$): δ 3.64 (app dq, *J* = 10.2, 4.9, 4.1 Hz, 1H), 2.60 – 2.33 (m, 3H), 2.09 (app dd, *J* = 11.6, 4.6 Hz, 1H), 2.03 – 1.89 (app dd, *J* = 12.1, 4.7 Hz, 1H), 1.89 – 1.78 (m, 2H), 1.72 – 1.68 (m, 3H), 1.65 – 1.44 (m, 6H), 1.42 – 1.17 (m, 5H), 1.14 – 0.98 (m, 2H).

^{13}C NMR (151 MHz, $CDCl_3$): *δ* 213.3, 70.1, 49.3, 40.2, 39.8, 37.1, 35.2, 32.7, 32.6, 30.1, 27.7, 25.3 (2C), 23.4.

HRMS (ESI$^+$): exact mass calculated for $[M+Na]^+$ ($C_{14}H_{24}O_2Na$) requires *m/z* 247.1674, found *m/z* 247.1666.

IR (neat) ν_{max}: 3399, 2933, 2858, 1703, 1449, 1407, 1360, 1273, 1195, 1143, 1055, 961, 749, 714 cm^{-1}.

65: *syn*-adamantan-1-yl-(3-hydroxycyclohexyl)methanone

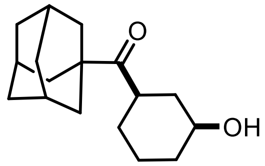

$C_{17}H_{26}O_2$
MW: 262.39
Yield: 82 %, >20:1 r.r. (δ)

Synthesised following the general procedure, using cyclohexene (20.3 µL, 0.20 mmol, 1.00 equiv.), adamantane-1-carbonyl chloride (**S1**) (41.7 mg, 0.21 mmol, 1.05 equiv.), silver hexafluoroantimonate (75.6 mg, 0.22 mmol, 1.10 equiv.) and dichloromethane (2 mL). Purification by flash column chromatography (heptane/ethyl acetate 90:10 to 20:80, Rf = 0.29 in heptane/ethyl acetate 75:25) gave the title compound (>20:1 r.r., 43.1 mg, 82%) as a pale-yellow oil.

^{1}H NMR (400 MHz, $CDCl_3$): *δ* 3.75 – 3.36 (m, 1H), 2.95 (tt, *J* = 11.3, 3.5 Hz, 1H), 2.05 (m, 3H), 2.00 – 1.92 (m, 1H), 1.91 – 1.62 (m, 15H), 1.54 (app dd, *J* = 9.9, 4.3 Hz, 1H), 1.46 – 1.18 (m, 4H).

^{13}C NMR (101 MHz, $CDCl_3$): *δ* 216.9, 70.2, 47.1, 42.1, 38.4, 37.9 (3C), 36.7 (3C), 35.4, 28.8, 28.0 (3C), 23.7.

HRMS (ESI$^+$): exact mass calculated for $[M+Na]^+$ ($C_{17}H_{22}O_2Na$) requires *m/z* 285.1831, found *m/z* 285.1822.

IR (neat) ν_{max}: 3374, 2904, 2850, 1694, 1451, 1354, 1304, 1275, 1256, 1201, 1162, 1063, 1017, 953, 935, 843, 751, 669 cm^{-1}.

66: *syn*-cyclopropyl(3-hydroxycyclohexyl)methanone

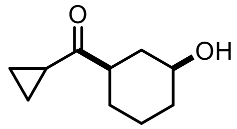

$C_{10}H_{16}O_2$
MW: 168.24
Yield: 81%, >20:1 r.r. (δ)

Synthesised following the general procedure, using cyclohexene (20.3 μL, 0.20 mmol, 1.00 equiv.), cyclopropanecarbonyl chloride (19.1 μL, 0.21 mmol, 1.05 equiv.), silver hexafluoroantimonate (75.6 mg, 0.22 mmol, 1.10 equiv.) and dichloromethane (2 mL). Purification by flash column chromatography (heptane/ethyl acetate 70:30 to 50:50, Rf = 0.31 in heptane/ethyl acetate 75:25) gave the title compound (>20:1 r.r., 27.6 mg, 81%) as a pale-yellow oil.

^{1}H NMR (600 MHz, $CDCl_3$): δ 3.67 (tt, *J* = 10.1, 4.2 Hz, 1H), 2.62 (tt, *J* = 11.0, 3.7 Hz, 1H), 2.19 – 2.10 (m, 1H), 2.04 (app brs, 1H), 2.01 – 1.91 (m, 2H), 1.91 – 1.85 (m, 2H), 1.44 – 1.30 (m, 3H), 1.25 (tdd, *J* = 13.8, 11.0, 3.7 Hz, 1H), 1.03 – 0.95 (m, 2H), 0.90 – 0.81 (m, 2H).

^{13}C NMR (151 MHz, $CDCl_3$): δ 213.0, 69.9, 50.0, 36.9, 35.2, 27.6, 23.1, 19.1, 11.1, 11.0.

HRMS (ESI$^+$): exact mass calculated for $[M+Na]^+$ ($C_{10}H_{16}O_2Na$) requires *m/z* 191.1042, found *m/z* 191.1043.

IR (neat) ν_{max}: 3397, 3008, 2934, 2858, 1691, 1449, 1391, 1363, 1274, 1197, 1147, 1060, 962, 912, 878, 849, 818, 749 cm^{-1}.

67: *syn*-cyclopropyl(3-hydroxycyclopentyl)methanone

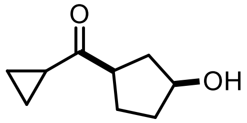

$C_9H_{14}O_2$
MW: 154.21
Yield: 52%

Synthesised following the general procedure, using cyclopentene (17.7 µL, 0.20 mmol, 1.00 equiv.), cyclopropanecarbonyl chloride (19.1 µL, 0.21 mmol, 1.05 equiv.), silver hexafluoroantimonate (75.6 mg, 0.22 mmol, 1.10 equiv.) and dichloromethane (2 mL). Purification by flash column chromatography (heptane/ethyl acetate 90:10 to 50:50, Rf = 0.32 in heptane/ethyl acetate 75:25) gave the title compound (16.0 mg, 52%) as a colourless oil.

^{1}H NMR (400 MHz, $CDCl_3$): δ 4.33 – 4.25 (m, 1H), 3.22 (dt, *J* = 13.7, 6.4 Hz, 1H), 2.81 (brs, 1H), 2.07 – 1.89 (m, 5H), 1.83 – 1.70 (m, 2H), 1.05 (dd, *J* = 5.9, 3.2 Hz, 2H), 0.98 – 0.87 (m, 2H). *The OH proton was not observed.*

^{13}C NMR (101 MHz, $CDCl_3$): δ 215.7, 73.7, 49.9, 37.6, 36.0, 27.7, 20.1, 11.6, 11.4.

HRMS (ESI$^+$): exact mass calculated for $[M+Na]^+$ ($C_9H_{14}O_2Na$) requires *m/z* 177.0886, found *m/z* 177.0888.

IR (neat) ν_{max}: 2959, 2925, 2853, 1748, 1723, 1462, 1377, 1327, 1268, 1203, 1117, 1101, 989, 919, 745, 732, 705 cm^{-1}.

68: *syn*-(3-hydroxycyclopentyl)nonan-1-one

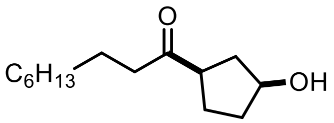

$C_{14}H_{26}O_2$
MW: 226.36
Yield: 58%

Synthesised following the general procedure, using cyclopentene (17.7 µL, 0.20 mmol, 1.00 equiv.), nonanoyl chloride (37.9 µL, 0.21 mmol, 1.05 equiv.), silver hexafluoroantimonate (75.6 mg, 0.22 mmol, 1.10 equiv.) and dichloromethane (2 mL). Purification by flash column chromatography (heptane/ethyl acetate 90:10 to 20:80, Rf = 0.36 in heptane/ethyl acetate 75:25) gave the title compound (26.3 mg, 58%) as a colourless oil.

^{1}H NMR (500 MHz, $CDCl_3$): δ 4.29 (t, *J* = 7.3 Hz, 1H), 3.19 – 2.99 (m, 1H), 2.57 – 2.45 (m, 2H), 1.99 – 1.91 (m, 1H), 1.91 – 1.82 (m, 2H), 1.79 – 1.69 (m, 2H), 1.57 (app p, *J* = 7.3 Hz, 2H), 1.33 – 1.20 (m, 12H), 0.87 (t, *J* = 7.0 Hz, 3H).

^{13}C NMR (126 MHz, $CDCl_3$): δ 216.1, 73.7, 49.1, 42.5, 37.6, 36.1, 32.0, 29.5, 29.4, 29.3, 27.7, 23.8, 22.8, 14.2.

HRMS (ESI$^+$): exact mass calculated for $[M+Na]^+$ ($C_{14}H_{26}O_2Na$) requires *m/z* 249.1831, found *m/z* 249.1824.

IR (neat) ν_{max}: 3408, 2923, 2853, 1705, 1462, 1407, 1375, 1283, 1203, 1130, 1078, 1008, 959, 841, 747, 722 cm^{-1}.

69: *syn*-1-(3-hydroxycyclohexyl)-3-phenylpropan-1-one

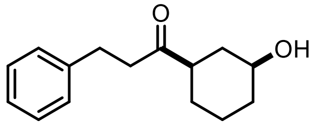

$C_{15}H_{20}O_2$
MW: 232.32
Yield: 64%, >20:1 r.r. (δ)

Synthesised following the general procedure, using cyclohexene (20.3 µL, 0.20 mmol, 1.00 equiv.), hydrocinnamoyl chloride (31.8 µL, 0.21 mmol, 1.05 equiv.), silver hexafluoroantimonate (75.6 mg, 0.22 mmol, 1.10 equiv.) and dichloromethane (2 mL). Purification by flash column chromatography (heptane/ethyl acetate 60:40 to 40:60, Rf = 0.26 in heptane/ethyl acetate 66:33) gave the title compound (>20:1 r.r., 29.7 mg, 64%) as a pale-yellow oil.

^{1}H NMR (400 MHz, $CDCl_3$): *δ* 7.31 – 7.24 (m, 2H), 7.22 – 7.14 (m, 3H), 3.67 – 3.53 (m, 1H), 2.94 – 2.83 (m, 2H), 2.82 – 2.74 (m, 2H), 2.39 (tt, *J* = 11.3, 3.5 Hz, 1H), 2.09 – 2.03 (m, 1H), 1.98 – 1.90 (m, 1H), 1.84 – 1.80 (m, 1H), 1.80 – 1.69 (m, 2H), 1.37 – 1.27 (m, 2H), 1.27 – 1.14 (m, 2H).

^{13}C NMR (101 MHz, $CDCl_3$): *δ* 211.9, 141.3, 128.6 (2C), 128.4 (2C), 126.2, 70.0, 49.5, 42.4, 36.9, 35.1, 29.8, 27.5, 23.3.

HRMS (ESI$^+$): exact mass calculated for $[M+H]^+$ ($C_{15}H_{21}O_2$) requires *m/z* 233.1536, found *m/z* 233.1531.

IR (neat) ν_{max}: 3464, 3086, 3027, 2934, 2857, 1740, 1704, 1604, 1495, 1451, 1405, 1364, 1327, 1301, 1269, 1229, 1220, 1200, 1142, 1130, 1111, 1069, 1055, 1031, 966, 952, 928, 906, 850, 818, 748 cm^{-1}.

70: *syn*-(3-hydroxycyclohexyl)-3-(4-(trifluoromethyl)phenyl)propan-1-one

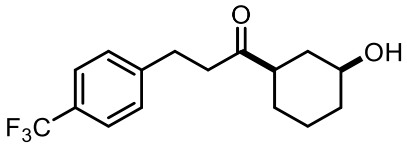

$C_{16}H_{19}F_3O_2$
MW: 300.13
Yield: 73%, >20:1 r.r. (δ)

Synthesised following the general procedure, using cyclohexene (20.3 µL, 0.20 mmol, 1.00 equiv.), **S3** (49.7 mg, 0.21 mmol, 1.05 equiv.), silver hexafluoroantimonate (75.6 mg, 0.22 mmol, 1.10 equiv.) and dichloromethane (2 mL). Purification by flash column chromatography (heptane/ethyl acetate 80:20 to 50:50, Rf = 0.30 in heptane/ethyl acetate 50:50) gave the title compound (>20:1 r.r., 43.7 mg, 73%) as a colourless solid.

^{1}H NMR (400 MHz, CDCl$_3$): *δ* 7.52 (d, *J* = 8.1 Hz, 2H), 7.28 (d, *J* = 8.0 Hz, 2H), 3.66 – 3.57 (m, 1H), 2.94 (t, *J* = 7.4 Hz, 2H), 2.79 (dd, *J* = 11.1, 4.2 Hz, 2H), 2.39 (ddd, *J* = 11.4, 7.4, 3.5 Hz, 1H), 2.13 – 2.02 (m, 1H), 2.00 – 1.91 (m, 1H), 1.89 – 1.71 (m, 3H), 1.42 – 1.10 (m, 4H).

^{13}C NMR (101 MHz, CDCl$_3$): *δ* 211.2, 145.5, 129.1 ($^2J_{C–F}$ coupling not visible), 128.8 (2C), 127.1 (q, *J* = 273.2 Hz), 125.5 (q, *J* = 3.7 Hz, 2C), 69.9, 49.4, 41.9, 36.9, 35.1, 29.5, 27.5, 23.3.

^{19}F NMR (377 MHz, CDCl$_3$): δ -62.37.

HRMS (ESI$^+$): exact mass calculated for $[M+Na]^+$ ($C_{16}H_{19}F_3O_2Na$) requires *m/z* 323.1229, found *m/z* 323.1223.

IR (neat) ν_{max}: 3341, 2934, 2858, 1710, 1667, 1618, 1467, 1451, 1418, 1364, 1323, 1269,

1230, 1191, 1162, 1122, 1109, 1067, 1018, 967, 952, 853, 830, 737 cm^{-1}.

Melting point: 65-67 °C.

71: *syn*-furan-2-yl-(-3-hydroxycyclohexyl)methanone

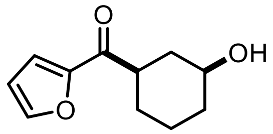

$C_{11}H_{14}O_3$
MW: 194.23
Yield: 51%, 10:1 r.r. (δ)

Synthesised following the general procedure, using cyclohexene (20.3 µL, 0.20 mmol, 1.00 equiv.), 2-furoyl chloride (22.7 µL, 0.21 mmol, 1.05 equiv.), silver hexafluoroantimonate (75.6 mg, 0.22 mmol, 1.10 equiv.) and dichloromethane (2 mL). Purification by flash column chromatography (heptane/ethyl acetate 80:20 to 50:50, Rf = 0.29 in heptane/ethyl acetate 50:50) gave the title compound (10:1 r.r., 20.0 mg, 51%) as a colourless oil.

^{1}H NMR (600 MHz, $CDCl_3$): δ 7.58 – 7.57 (m, 1H), 7.20 – 7.19 (m, 1H), 6.57 – 6.42 (m, 1H), 3.74 (tt, *J* = 10.4, 4.2 Hz, 1H), 3.18 (tt, *J* = 11.2, 3.6 Hz, 1H), 2.18 – 2.07 (m, 1H), 2.06 – 1.97 (m, 1H), 1.95 – 1.78 (m, 3H), 1.57 – 1.48 (m, 1H), 1.50 – 1.36 (m, 2H), 1.34 – 1.16 (m, 1H).

^{13}C NMR (151 MHz, $CDCl_3$): δ 191.5, 152.3, 146.5, 117.5, 112.5, 70.0, 44.8, 37.3, 35.2, 28.1, 23.5.

HRMS (ESI$^+$): exact mass calculated for $[M+Na]^+$ ($C_{11}H_{14}O_3Na$) requires *m/z* 217.0841, found *m/z* 217.0837.

IR (neat) ν_{max}: 3408, 3128, 2935, 2859, 1666, 1565, 1465, 1397, 1361, 1275, 1193, 1163, 1137, 1062, 1032, 1008, 956, 915, 882, 846, 765 cm^{-1}.

72: *syn*-(3-hydroxycyclohexyl)(thiophen-2-yl)methanone

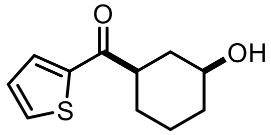

$C_{11}H_{14}O_2S$
MW: 210.29
Yield: 74%, 13:1 r.r. (δ)

Synthesised following the general procedure, using cyclohexene (20.3 µL, 0.20 mmol, 1.00 equiv.), thiophene-2-carbonyl chloride (22.7 µL, 0.21 mmol, 1.05 equiv.), silver hexafluoroantimonate (75.6 mg, 0.22 mmol, 1.10 equiv.) and dichloromethane (2 mL). Purification by flash column chromatography (heptane/ethyl acetate 80:20 to 50:50, Rf = 0.29 in heptane/ethyl acetate 50:50) gave the title compound (13:1 r.r., 31.2 mg, 74%) as a pale-yellow oil.

^{1}H NMR (400 MHz, $CDCl_3$): δ 7.78 – 7.66 (m, 1H), 7.66 – 7.60 (m, 1H), 7.17 – 7.01 (m, 1H), 3.76 (app d, *J* = 10.2 Hz, 1H), 3.23 – 3.18 (m, 1H), 2.21 – 2.06 (m, 1H), 2.08 – 1.96 (m, 1H), 1.94 – 1.77 (m, 3H), 1.64 – 1.38 (m, 3H), 1.37 – 1.21 (m, 1H).

^{13}C NMR (101 MHz, $CDCl_3$): δ 195.4, 143.5, 133.9, 131.9, 128.3, 69.9, 45.8, 37.9, 35.2, 28.9, 23.4.

HRMS (ESI$^+$): exact mass calculated for $[M+Na]^+$ ($C_{11}H_{14}O_2Na$) requires *m/z* 233.0613, found *m/z* 233.0610.

IR (neat) ν_{max}: 3389, 3088, 2934, 2858, 1652, 1517, 1449, 1413, 1353, 1261, 1211, 1185, 1134, 1059, 983, 940, 879, 861, 764, 724 cm^{-1}.

73: *syn*-(3-hydroxycyclopentyl)(thiophen-2-yl)methanone

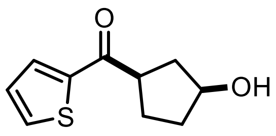

$C_{10}H_{12}O_2S$
MW: 196.26
Yield: 37%

Synthesised following the general procedure, using cyclopentene (17.7 µL, 0.20 mmol, 1.00 equiv.), thiophene-2-carbonyl chloride (22.7 µL, 0.21 mmol, 1.05 equiv.), silver hexafluoroantimonate (75.6 mg, 0.22 mmol, 1.10 equiv.) and dichloromethane (2 mL). Purification by flash column chromatography (heptane/ethyl acetate 80:20 to 50:50, Rf = 0.40 in heptane/ethyl acetate 50:50) gave the title compound (14.5 mg, 37%) as a pale-yellow oil.

^{1}H NMR (400 MHz, $CDCl_3$): *δ* 7.77 (d, *J* = 3.7 Hz, 1H), 7.69 (d, *J* = 4.9 Hz, 1H), 7.16 (t, *J* = 4.3 Hz, 1H), 4.36 (br s, 1H), 3.80 (app dq, *J* = 9.3, 6.2 Hz, 1H), 3.22 (br s, 1H), 2.24 – 1.98 (m, 4H), 1.84 (ddd, *J* = 13.2, 8.7, 4.4 Hz, 2H).

^{13}C NMR (101 MHz, $CDCl_3$): *δ* 198.1, 143.6, 134.7, 132.9, 128.5, 73.8, 45.9, 38.5, 36.6, 29.6.

HRMS (ESI$^+$): exact mass calculated for $[M+Na]^+$ ($C_{10}H_{12}O_2SNa$) requires *m/z* 219.0450, found *m/z* 219.0446.

IR (neat) ν_{max}: 3399, 3093, 2955, 2362, 1737, 1653, 1518, 1414, 1353, 1239, 1080, 1059, 986, 844, 810, 728 cm^{-1}.

74: *syn*-(3-hydroxycyclododecyl)(phenyl)methanone

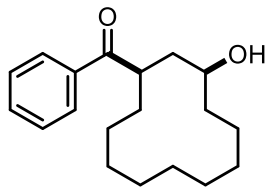

$C_{19}H_{28}O_2$
MW: 288.43
Yield: 43%, >20:1 r.r. (δ)

Synthesised following the general procedure, using cyclododecene (193 µL, 1.00 mmol, 1.00 equiv.), benzoyl chloride (122 µL, 1.05 mmol, 1.05 equiv.), silver hexafluoroantimonate (378 mg, 1.10 mmol, 1.10 equiv.) and dichloromethane (10 mL). Purification by flash column chromatography (heptane/ethyl acetate 80:20 to 40:60, Rf = 0.34 in heptane/ethyl acetate 80:20) gave the title compound (>20:1 r.r., 124 mg, 43%) as a colourless solid.

^{1}H NMR (600 MHz, CDCl$_3$): *δ* 7.95 (dd, *J* = 8.2, 1.1 Hz, 2H), 7.59 – 7.53 (m, 1H), 7.46 (dd, *J* = 10.7, 4.7 Hz, 2H), 3.97 (dd, *J* = 9.0, 4.3 Hz, 1H), 3.62 (ddt, *J* = 10.2, 7.0, 3.5 Hz, 1H), 2.27 (ddd, *J* = 14.9, 6.7, 5.2 Hz, 1H), 2.21 (s, 1H), 1.77 (dt, *J* = 15.0, 3.6 Hz, 1H), 1.70 (ddd, *J* = 13.8, 9.0, 4.3 Hz, 2H), 1.54 (ddd, *J* = 15.5, 9.8, 5.2 Hz, 3H), 1.49 – 1.33 (m, 13H).

^{13}C NMR (151 MHz, CDCl$_3$): *δ* 205.3, 136.7, 133.0, 128.8 (2C), 128.5 (2C), 68.0, 40.1, 33.4, 32.6, 28.9, 24.8, 24.3, 23.9, 23.6, 23.2, 22.9, 21.9.

HRMS (ESI$^+$): exact mass calculated for $[M+Na]^+$ ($C_{19}H_{28}O_2Na$) requires *m/z* 311.1982, found *m/z* 311.1976.

IR (neat) ν$_{max}$: 3429, 2933, 2861, 2361, 1733, 1681, 1596, 1580, 1469, 1446, 1332, 1235, 1210, 1157, 1078, 1046, 1027, 989, 962, 784 cm^{-1}.

Melting point: 120-122 °C.

75: *syn*-(3-hydroxycyclohexyl)(m-tolyl)methanone

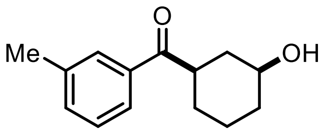

$C_{14}H_{18}O_2$
MW: 218.29
Yield: 82%, >20:1 r.r. (δ)

Synthesised following the general procedure, using cyclohexene (20.3 µL, 0.20 mmol, 1.00 equiv.), *m*-toluoyl chloride (27.7 µL, 0.21 mmol, 1.05 equiv.), silver hexafluoroantimonate (75.6 mg, 0.22 mmol, 1.10 equiv.) and dichloromethane (2 mL). Purification by flash column chromatography (heptane/ethyl acetate 90:10 to 20:80, Rf = 0.35 in heptane/ethyl acetate 2:1) gave the title compound (>20:1 r.r., 35.8 mg, 82%) as a pale-yellow oil.

^{1}H NMR (500 MHz, $CDCl_3$): δ 7.77 – 7.62 (m, 2H), 7.40 – 7.30 (m, 2H), 3.87 – 3.62 (m, 1H), 3.36 (tt, *J* = 11.1, 3.4 Hz, 1H), 2.41 (s, 3H), 2.20 – 2.08 (m, 1H), 2.05 – 1.95 (m, 1H), 1.95 – 1.80 (m, 3H), 1.56 – 1.37 (m, 3H), 1.36 – 1.17 (m, 1H).

^{13}C NMR (126 MHz, $CDCl_3$) δ 202.8, 138.6, 136.2, 133.9, 129.0, 128.7, 125.6, 70.1, 44.1, 37.9, 35.3, 28.6, 23.5, 21.5.

HRMS (ESI$^+$): exact mass calculated for $[M+Na]^+$ ($C_{14}H_{18}O_2Na$) requires *m/z* 241.1205, found *m/z* 241.1199.

IR (neat) ν_{max}: 3398, 2934, 2858, 1676, 1601, 1585, 1449, 1361, 1265, 1164, 1133, 1117, 1062, 957, 901, 868, 802, 763, 749, 681 cm^{-1}.

76: *syn*-3-hydroxycyclohexyl(2-bromophenyl)methanone

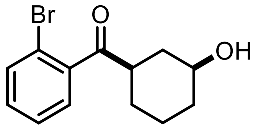

$C_{13}H_{15}BrO_2$
MW: 283.16
Yield: 60%, 11:1 r.r. (δ)

Synthesised following the general procedure, using cyclohexene (20.3 µL, 0.20 mmol, 1.00 equiv.), 2-bromobenzoyl chloride (27.4 µL, 0.21 mmol, 1.05 equiv.), silver hexafluoroantimonate (75.6 mg, 0.22 mmol, 1.10 equiv.) and dichloromethane (2 mL). Purification by flash column chromatography (heptane/ethyl acetate 90:10 to 40:60, Rf = 0.28 in heptane/ethyl acetate 3:1) gave the title compound (11:1 r.r., 34.2 mg, 60%) as a colourless solid.

^{1}H NMR (600 MHz, $CDCl_3$): δ 7.59 (dd, *J* = 7.9, 0.7 Hz, 1H), 7.35 (td, *J* = 7.5, 1.1 Hz, 1H), 7.30 – 7.24 (m, 2H), 3.66 (tt, *J* = 10.6, 4.2 Hz, 1H), 3.18 – 3.06 (m, 1H), 2.24 – 2.17 (m, 1H), 2.04 – 1.99 (m, 1H), 1.92 – 1.84 (m, 2H), 1.44 (dd, *J* = 23.0, 12.1 Hz, 1H), 1.39 – 1.20 (m, 4H).

^{13}C NMR (151 MHz, $CDCl_3$): δ 206.6, 141.9, 133.5, 131.4, 128.3, 127.4, 118.7, 70.2, 48.6, 37.1, 35.2, 27.5, 23.5.

HRMS (ESI$^+$): exact mass calculated for $[M+Na]^+$ ($C_{13}H_{15}BrO_2Na$) requires *m/z* 305.0148, found *m/z* 305.0150.

IR (neat) ν_{max}: 3366, 2934, 2857, 1698, 1587, 1563, 1464, 1448, 1428, 1360, 1276, 1209, 1136, 1054, 1028, 1008, 954, 881, 806, 764, 743 cm^{-1}.

Melting point: 63-68 °C

77: *syn*-(3-hydroxycyclohexyl)(p-tolyl)methanone

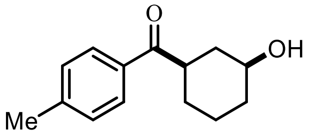

$C_{14}H_{18}O_2$
MW: 218.29
Yield: 78%, >20:1 r.r. (δ)

Synthesised following the general procedure, using cyclohexene (20.3 µL, 0.20 mmol, 1.00 equiv.), *p*-toluoyl chloride (27.8 µL, 0.21 mmol, 1.05 equiv.), silver hexafluoroantimonate (75.6 mg, 0.22 mmol, 1.10 equiv.) and dichloromethane (2 mL). Purification by flash column chromatography (heptane/ethyl acetate 90:10 to 20:80, Rf = 0.30 in heptane/ethyl acetate 3:1) gave the title compound (>20:1 r.r., 34.2 mg, 78%) as a colourless solid.

^{1}H NMR (400 MHz, $CDCl_3$) δ 7.88 – 7.78 (m, 2H), 7.28 – 7.22 (m, 2H), 3.83 – 3.71 (m, 1H), 3.36 (app ddt, *J* = 14.5, 7.2, 3.5 Hz, 1H), 2.41 (s, 3H), 2.16 – 2.10 (m, 1H), 2.05 – 1.97 (m, 1H), 1.92 – 1.77 (m, 3H), 1.56 – 1.50 (m, 1H), 1.48 – 1.39 (m, 2H), 1.34 – 1.27 (m, 1H).

^{13}C NMR (101 MHz, $CDCl_3$): δ 202.2, 144.0, 133.6, 129.5 (2C), 128.6 (2C), 70.2, 44.0, 37.9, 35.4, 28.7, 23.5, 21.8.

HRMS (ESI$^+$): exact mass calculated for $[M+Na]^+$ ($C_{14}H_{18}O_2Na$) requires *m/z* 241.1205, found *m/z* 241.1197.

IR (neat) ν_{max}: 3386, 2934, 2858, 1674, 1606, 1571, 1449, 1408, 1360, 1263, 1235, 1206, 1181, 1135, 1117, 1061, 1014, 955, 945, 882, 853, 827, 766, 747, 685 cm^{-1}.

Melting point: 98-114 °C

78: 2-((1*R,2*R**,4*R**)-2-hydroxy-7,7-dimethylbicyclo[2.2.1]heptan-1-yl)-1-phenylethan-1-one**

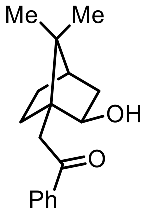

$C_{17}H_{22}O_2$
MW: 258.36
Yield: 32%
>20:1 d.r.

Synthesised following the general procedure, using (±)-camphene (64.2 µL, 0.40 mmol, 1.00 equiv.), benzoyl chloride (48.4 µL, 0.42 mmol, 1.05 equiv.), silver hexafluoroantimonate (151.2 mg, 0.44 mmol, 1.10 equiv.) and dichloromethane (4 mL), with a reaction time of 30 minutes. Purification by flash column chromatography (heptane/ethyl acetate 80:20 to 50:50, Rf = 0.71 in heptane/ethyl acetate 2:1) gave the title compound (>20:1 d.r., 33.0 mg, 32%) as a pale-yellow solid.

^{1}H NMR (400 MHz, $CDCl_3$): δ 8.00 (d, *J* = 7.4 Hz, 2H), 7.62 – 7.53 (m, 1H), 7.48 (t, *J* = 7.7 Hz, 2H), 3.89 – 3.74 (m, 1H), 3.43 (d, *J* = 2.6 Hz, 1H), 3.22 (d, *J* = 13.5 Hz, 1H), 2.83 (d, *J* = 13.5 Hz, 1H), 1.87 – 1.76 (m, 1H), 1.77 – 1.63 (m, 3H), 1.52 – 1.42 (m, 1H), 1.15 (s, 3H), 1.05 – 0.96 (m, 2H), 0.94 (s, 3H).

^{13}C NMR (101 MHz, $CDCl_3$): δ 203.7, 137.8, 133.6, 128.8 (2C), 128.7 (2C), 77.2, 52.7, 47.7, 44.8, 38.9, 34.9, 31.1, 27.4, 20.9, 20.2.

HRMS (ESI$^+$): exact mass calculated for $[M+Na]^+$ ($C_{17}H_{22}O_2Na$) requires *m/z* 281.1512, found *m/z* 281.1515.

IR (neat) ν_{max}: 3468, 2943, 2121, 1877, 1665, 1596, 1580, 1450, 1361, 1296, 1222, 1208, 1075, 1027, 933, 704, 641 cm^{-1}.

Melting point: 76-80 °C

79: *syn*-(3-hydroxycyclododecyl)-2-methylpropan-1-one

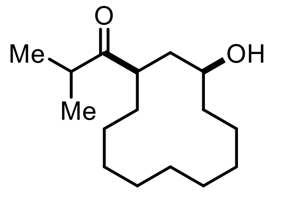

$C_{16}H_{30}O_2$
MW: 254.41
Yield: 43%, >20:1 r.r. (δ)

Synthesised following the general procedure, using cyclododecene (38.5 µL, 0.20 mmol, 1.00 equiv.), isobutyryl chloride (22.0 µL, 0.21 mmol, 1.05 equiv.), silver hexafluoroantimonate (75.6 mg, 0.22 mmol, 1.10 equiv.) and dichloromethane (2 mL). Purification by flash column chromatography (heptane/ethyl acetate 70:30, Rf = 0.30 in heptane/ethyl acetate 3:1) gave the title compound (>20:1 r.r., 21.8 mg, 43%) as a colourless solid.

^{1}H NMR (400 MHz, $CDCl_3$): *δ* 3.88 (dd, *J* = 8.7, 4.1 Hz, 1H), 2.94 – 2.76 (m, 2H), 2.18 (app s, 1H), 2.03 (ddd, *J* = 14.8, 6.5, 5.2 Hz, 1H), 1.70 – 1.51 (m, 3H), 1.48 – 1.16 (m, 16H), 1.09 (t, *J* = 6.6 Hz, 6H).

^{13}C NMR (176 MHz, $CDCl_3$): *δ* 219.6, 67.9, 43.7, 39.9, 33.5, 32.5, 27.8, 24.7, 24.1, 23.9, 23.4, 23.1, 23.1, 21.9, 19.2, 18.7.

HRMS (ESI$^+$): exact mass calculated for $[M+Na]^+$ ($C_{16}H_{30}O_2Na$) requires *m/z* 277.2138, found *m/z* 277.2126.

IR (neat) ν_{max}: 3444, 2934, 2864, 2361, 1704, 1469, 1446, 1383, 1345, 1267, 1189, 1158, 1080, 1026, 1004, 741 cm^{-1}.

Melting point: 76-80 °C

80: *syn*-(3-hydroxycyclohexyl)-3,3-dimethylbutan-1-one

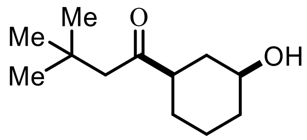

$C_{12}H_{22}O_2$
MW: 198.30
Yield: 40%, >20:1 r.r. (δ)

Synthesised following the general procedure, using cyclohexene (20.3 µL, 0.20 mmol, 1.00 equiv.), 3,3-dimethylbutyryl chloride (29.4 µL, 0.21 mmol, 1.05 equiv.), silver hexafluoroantimonate (75.6 mg, 0.22 mmol, 1.10 equiv.) and dichloromethane (2 mL). Purification by flash column chromatography (heptane/ethyl acetate 80:20 to 50:50, Rf = 0.34 in heptane/ethyl acetate 2:1) gave the title compound (>20:1 r.r., 16.0 mg, 40%) as a pale-yellow oil.

^{1}H NMR (400 MHz, $CDCl_3$) δ 3.65 – 3.60 (m, 1H), 2.47 – 2.34 (m, 1H), 2.34 (s, 2H), 2.05 (ddt, *J* = 12.4, 4.0, 2.1 Hz, 1H), 1.99 – 1.87 (m, 1H), 1.89 – 1.71 (m, 3H), 1.33 – 1.27 (m, 4H), 1.00 (s, 9H).

^{13}C NMR (101 MHz, $CDCl_3$): δ 212.8, 70.1, 53.1, 50.8, 36.8, 35.3, 31.2, 29.9 (3C), 27.4, 23.3.

HRMS (ESI$^+$): exact mass calculated for $[M+Na]^+$ ($C_{12}H_{22}O_2Na$) requires *m/z* 221.1518, found *m/z* 221.1505.

IR (neat) ν_{max}: 3371, 2936, 2862, 2364, 2220, 2024, 1706, 1434, 1364, 1275, 1260, 1141, 1060, 968, 919, 763, 749 cm^{-1}.

81: *syn*-1-(3-hydroxycyclopentyl)-2,2-dimethylpropan-1-one

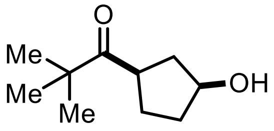

$C_{10}H_{18}O_2$
MW: 170.25
Yield: 68%

Synthesised following the general procedure, using cyclopentene (17.7 µL, 0.20 mmol, 1.00 equiv.), pivaloyl chloride (25.9 µl, 0.21 mmol, 1.05 equiv.), silver hexafluoroantimonate (75.6 mg, 0.22 mmol, 1.10 equiv.) and dichloromethane (2 mL). Purification by flash column chromatography (heptane/ethyl acetate 80:20 to 50:50, Rf = 0.33 in heptane/ethyl acetate 3:1) gave the title compound (23.1 mg, 68%) as a colourless oil.

^{1}H NMR (700 MHz, $CDCl_3$): δ 4.27 (brs, 1H), 3.68 (brs, 1H), 3.49 (app qd, *J* = 9.4, 2.7 Hz, 1H), 1.98 (ddd, *J* = 16.2, 11.4, 6.2 Hz, 1H), 1.93 – 1.85 (m, 2H), 1.76 – 1.65 (m, 3H), 1.16 (s, 9H).

^{13}C NMR (176 MHz, $CDCl_3$): δ 223.2, 74.0, 44.4, 42.7, 39.5, 37.2, 30.1, 26.1 (3C).

As the carbonyl was not sufficiently visible in the ^{13}C DEPTQ-135 NMR spectrum, an additional ^{13}C CPD NMR spectrum is attached.

HRMS (ESI$^+$): exact mass calculated for $[M+Na]^+$ ($C_{10}H_{18}O_2Na$) requires *m/z* 193.1199, found *m/z* 193.1209.

IR (neat) ν_{max}: 2960, 2925, 2854, 1748, 1724, 1463, 1378, 1327, 1268, 1202, 1118, 1102, 995, 911, 745, 732 cm^{-1}.

82: *syn*-(3-hydroxycyclohexyl)-2-methylpropan-1-one

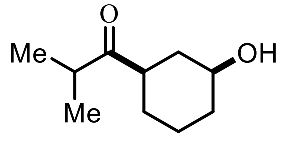

$C_{10}H_{18}O_2$
MW: 170.25
Yield: 59%, >20:1 r.r. (δ)

Synthesised following the general procedure, using cyclohexene (20.3 µL, 0.20 mmol, 1.00 equiv.), isobutyryl chloride (22.0 µL, 0.21 mmol, 1.05 equiv.), silver hexafluoroantimonate (75.6 mg, 0.22 mmol, 1.10 equiv.) and dichloromethane (2 mL). Purification by flash column chromatography (heptane/ethyl acetate 80:20 to 50:50, Rf = 0.34 in heptane/ethyl acetate 2:1) gave the title compound (>20:1 r.r., 20.0 mg, 59%) as a pale-yellow oil.

^{1}H NMR (600 MHz, $CDCl_3$): *δ* 3.62 (tt, *J* = 10.4, 4.2 Hz, 1H), 2.78 – 2.70 (m, 1H), 2.58 (tt, *J* = 11.3, 3.6 Hz, 1H), 2.05 – 1.97 (m, 1H), 1.97 – 1.90 (m, 1H), 1.85 – 1.79 (m, 1H), 1.75 – 1.67 (m, 1H), 1.63 (brs, 1H) 1.37 – 1.16 (m, 4H), 1.09 – 0.97 (m, 6H).

^{13}C NMR (151 MHz, $CDCl_3$): *δ* 216.7, 70.1, 47.5, 39.2, 37.2, 35.3, 27.8, 23.4, 18.7, 18.5.

HRMS (ESI$^+$): exact mass calculated for $[M+Na]^+$ ($C_{10}H_{18}O_2Na$) requires *m/z* 193.1187, found *m/z* 193.1192.

IR (neat) ν_{max}: 3499, 2967, 2934, 2859, 1704, 1465, 1450, 1383, 1364, 1275, 1197, 1142, 1058, 958, 875, 849, 806, 749, 647 cm^{-1}.

3.4. Synthesis of *anti*-alcohols

3.4.1. General Procedure 3 for the 1,3-hydroxyacylation of alkenes with acylium ions

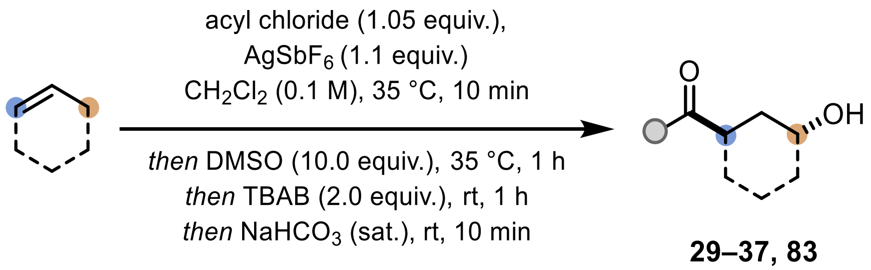

29–37, 83

All reactions were performed on a 0.2 mmol scale.

In a flame-dried Schlenk tube or oven-dried vial charged with a magnetic stir bar at ambient temperature, a solution of alkene (1.00 equiv.) and acyl chloride (1.05 equiv.) in dichloromethane (0.1 M) was treated with silver hexafluoroantimonate (1.10 equiv.) under argon atmosphere and the resulting suspension was immediately placed in a pre-heated oil (or sand) bath at 35 °C. After vigorously stirring the reaction mixture at the same temperature for 10 min, dimethyl sulfoxide (10.0 equiv.) was added and the reaction mixture was vigorously stirred for 1 h at 35 °C. Then, the reaction vessel was removed from the oil (or sand) bath and a solution of tetrabutylammonium bromide (2.0 equiv.) in DCM (10 M) was immediately added. The reaction mixture was vigorously stirred for 1 h at room temperature, after which time a saturated aqueous solution of sodium bicarbonate (equal volume to that of dichloromethane) was added, followed by vigorous stirring for 10 min. After this time, the phases were separated, the aqueous phase was extracted with dichloromethane (3 x 5 mL) and the combined organic phases were dried over anhydrous magnesium sulfate, filtered and the filtrate was concentrated under reduced pressure. The resulting crude material was purified by flash column chromatography on silica gel (heptane/ethyl acetate) to give the title compounds **29–37, 83**. All *anti*-alcohols were obtained as single diastereomers (unless reported otherwise). Where the products were formed as regioisomeric mixtures (providing the inseparable δ-hydroxyl product, as judged by ^{1}H NMR analysis), this has been indicated.

29: *anti*-(3-hydroxycyclohexyl)(4-(trifluoromethyl)phenyl)methanone

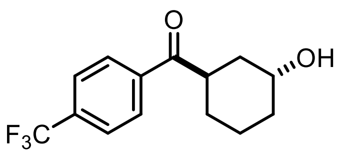

$C_{14}H_{15}F_3O_2$
MW: 272.27
Yield: 66%, >20:1 r.r. (δ)
>20:1 d.r.

Synthesised following the general procedure, using cyclohexene (20.3 µL, 0.20 mmol, 1.00 equiv.), 4-(trifluoromethyl)benzoyl chloride (32.2 µL, 0.21 mmol, 1.05 equiv.), silver hexafluoroantimonate (75.6 mg, 0.22 mmol, 1.10 equiv.), DMSO (142 µL, 2.00 mmol, 10.0 equiv.), tetrabutylammonium bromide (129 mg, 0.40 mmol, 2.00 equiv.) and dichloromethane (2 mL). Purification by flash column chromatography (heptane/ethyl acetate 80:20 to 50:50, Rf = 0.22 in heptane/ethyl acetate 75:25) gave the title compound (>20:1 r.r., >20:1 d.r., 36.1 mg, 66%) as a pale-yellow oil.

^{1}H NMR (400 MHz, $CDCl_3$): *δ* 8.05 (d, *J* = 8.1 Hz, 2H), 7.71 (d, *J* = 8.2 Hz, 2H), 4.25 (d, *J* = 2.6 Hz, 1H), 3.79 (tt, *J* = 11.0, 3.5 Hz, 1H), 1.97 – 1.85 (m, 2H), 1.85 – 1.71 (m, 2H), 1.69 – 1.43 (m, 4H). *The OH proton was not observed.*

^{13}C NMR (101 MHz, $CDCl_3$): *δ* 202.9, 139.0, 134.3 (q, *J* = 32.7 Hz), 128.8 (2C), 125.8 (q, *J* = 3.7 Hz, 2C), 123.8 (q, *J* = 272.5 Hz), 66.2, 40.2, 35.4, 32.8, 28.8, 19.8.

^{19}F NMR (377 MHz, $CDCl_3$): *δ* -63.11.

HRMS (ESI$^+$): exact mass calculated for $[M+H]^+$ ($C_{14}H_{16}F_3O_2$) requires *m/z* 273.1097, found *m/z* 273.1096.

IR (neat) ν_{max}: 3422, 2935, 2863, 1682, 1580, 1511, 1450, 1409, 1322, 1235, 1166, 1123, 1065, 1016, 967, 908, 890, 846, 807, 783 cm^{-1}.

30: *anti*-(3-hydroxycyclohexyl)-2,2-dimethylpropan-1-one

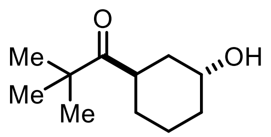

$C_{11}H_{20}O_2$
MW: 184.28
Yield: 61%, >20:1 r.r. (δ)
>20:1 d.r.

Synthesised following the general procedure, using cyclohexene (20.3 µL, 0.20 mmol, 1.00 equiv.), pivaloyl chloride (25.9 µL, 0.21 mmol, 1.05 equiv.), silver hexafluoroantimonate (75.6 mg, 0.22 mmol, 1.10 equiv.), DMSO (142 µL, 2.00 mmol, 10.0 equiv.), tetrabutylammonium bromide (129 mg, 0.40 mmol, 2.00 equiv.) and dichloromethane (2 mL). Purification by flash column chromatography (heptane/ethyl acetate 80:20 to 50:50, Rf = 0.25 in heptane/ethyl acetate 75:25) gave the title compound (>20:1 r.r., >20:1 d.r., 22.4 mg, 61%) as a pale-yellow solid.

^{1}H NMR (700 MHz, $CDCl_3$): *δ* 4.18 (dd, *J* = 5.9, 2.9 Hz, 1H), 3.42 – 3.27 (m, 1H), 1.75 – 1.68 (m, 2H), 1.67 – 1.61 (m, 3H), 1.59 – 1.51 (m, 2H), 1.50 – 1.42 (m, 1H), 1.43 – 1.35 (m, 1H), 1.14 (s, 9H).

^{13}C NMR (176 MHz, $CDCl_3$): *δ* 219.0, 66.2, 45.0, 38.5, 36.0, 32.4, 29.5, 26.1 (3C), 19.5.

HRMS (ESI$^+$): exact mass calculated for $[M+H]^+$ ($C_{11}H_{21}O_2$) requires *m/z* 185.1536, found *m/z* 185.1536.

IR (neat) ν_{max}: 3424, 2930, 2865, 1689, 1478, 1450, 1394, 1366, 1314, 1269, 1225, 1180, 1129, 1072, 1014, 981, 887, 857, 822, 789 cm^{-1}.

Melting point: 41-43 °C.

31: *anti*-(4-(*tert*-butyl)phenyl)(3-hydroxycyclopentyl)methanone

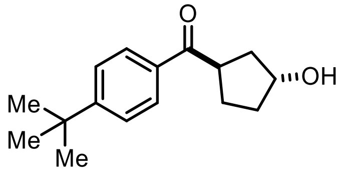

$C_{16}H_{22}O_2$
MW: 246.35
Yield: 76%
>20:1 d.r.

Synthesised following the general procedure, using cyclopentene (20.3 µL, 0.20 mmol, 1.00 equiv.), 4-*tert*-butylbenzoyl chloride (41.3 µL, 0.21 mmol, 1.05 equiv.), silver hexafluoroantimonate (75.6 mg, 0.22 mmol, 1.10 equiv.), DMSO (142 µL, 2.00 mmol, 10.0 equiv.), tetrabutylammonium bromide (129 mg, 0.40 mmol, 2.00 equiv.) and dichloromethane (2 mL). Purification by flash column chromatography (heptane/ethyl acetate 80:20 to 50:50, Rf = 0.22 in heptane/ethyl acetate 66:33) gave the title compound (>20:1 d.r., 37.4 mg, 76%) as a colourless oil.

^{1}H NMR (400 MHz, $CDCl_3$): δ 8.11 – 7.89 (m, 2H), 7.58 – 7.39 (m, 2H), 4.52 (dt, *J* = 7.1, 2.3 Hz, 1H), 4.04 (app qd, *J* = 8.6, 6.0 Hz, 1H), 2.28 – 2.13 (m, 2H), 2.03 – 1.83 (m, 3H), 1.82 – 1.68 (m, 1H), 1.55 (brs, 1H), 1.34 (d, *J* = 2.4 Hz, 9H).

^{13}C NMR (101 MHz, $CDCl_3$): δ 202.0, 156.8, 134.1, 128.6 (2C), 125.7 (2C), 74.2, 44.2, 39.0, 35.3, 35.2, 31.2 (3C), 27.8.

HRMS (ESI$^+$): exact mass calculated for $[M+H]^+$ ($C_{16}H_{23}O_2$) requires *m/z* 247.1693, found *m/z* 247.1690.

IR (neat) ν_{max}: 3420, 2963, 2870, 2248, 1673, 1604, 1565, 1462, 1408, 1363, 1299, 1268, 1226, 1190, 1109, 1009, 974, 909, 847, 779 cm^{-1}.

32: *anti*-(3-hydroxycyclopentyl)(m-tolyl)methanone

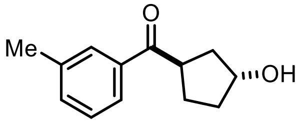

$C_{13}H_{16}O_2$
MW: 204.27
Yield: 80%
>20:1 d.r.

Synthesised following the general procedure, using cyclopentene (17.7 µL, 0.20 mmol, 1.00 equiv.), *m*-toluoyl chloride (27.7 µL, 0.21 mmol, 1.05 equiv.), silver hexafluoroantimonate (75.6 mg, 0.22 mmol, 1.10 equiv.), DMSO (142 µL, 2.00 mmol, 10.0 equiv.), tetrabutylammonium bromide (129 mg, 0.40 mmol, 2.00 equiv.) and dichloromethane (2 mL). Purification by flash column chromatography (heptane/ethyl acetate 80:20 to 50:50, Rf = 0.23 in heptane/ethyl acetate 66:33) gave the title compound (>20:1 d.r., 32.7 mg, 80%) as a colourless oil.

^{1}H NMR (400 MHz, $CDCl_3$): δ 7.81 – 7.73 (m, 2H), 7.40 – 7.32 (m, 2H), 4.51 (td, J = 4.8, 2.4 Hz, 1H), 4.12 – 3.93 (m, 1H), 2.40 (s, 3H), 2.26 – 2.13 (m, 2H), 1.99 – 1.87 (m, 3H), 1.83 (s, 1H), 1.77 – 1.67 (m, 1H).

^{13}C NMR (101 MHz, $CDCl_3$): δ 202.6, 138.5, 136.8, 133.8, 129.2, 128.6, 125.9, 74.1, 44.3, 39.0, 35.2, 27.8, 21.5.

HRMS (ESI$^+$): exact mass calculated for $[M+H]^+$ ($C_{13}H_{17}O_2$) requires *m/z* 205.1223, found *m/z* 205.1219.

IR (neat) ν_{max}: 3408, 3047, 2938, 2868, 1675, 1602, 1584, 1485, 1432, 1354, 1306, 1249, 1165, 1017, 976, 938, 914, 842, 796 cm^{-1}.

33: *anti*-(3-hydroxycyclohexyl)(thiophen-2-yl)methanone

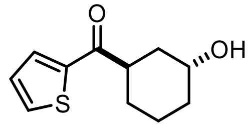

$C_{11}H_{14}O_2S$
MW: 210.29
Yield: 66%, 14:1 r.r. (δ)
>20:1 d.r.

Synthesised following the general procedure, using cyclohexene (20.3 µL, 0.20 mmol, 1.00 equiv.), thiophene-2-carbonyl chloride (22.7 µL, 0.21 mmol, 1.05 equiv.), silver hexafluoroantimonate (75.6 mg, 0.22 mmol, 1.10 equiv.), DMSO (142 µL, 2.00 mmol, 10.0 equiv.), tetrabutylammonium bromide (129 mg, 0.40 mmol, 2.00 equiv.) and dichloromethane (2 mL). Purification by flash column chromatography (heptane/ethyl acetate 80:20 to 50:50, Rf = 0.19 in heptane/ethyl acetate 66:33) gave the title compound (14:1 r.r, >20:1 d.r., 27.7 mg, 66%) as a colourless oil.

^{1}H NMR (400 MHz, $CDCl_3$) δ 7.76 (s, 1H), 7.61 (dd, *J* = 3.1, 1.6 Hz, 1H), 7.11 (s, 1H), 4.25 (app s, 1H), 3.64 (t, *J* = 9.7 Hz, 1H), 1.98 – 1.89 (m, 2H), 1.89 – 1.78 (m, 2H), 1.78 – 1.70 (m, 1H). 1.69 – 1.52 (m, 4H).

^{13}C NMR (151 MHz, $CDCl_3$) δ 196.8, 143.8, 133.7, 132.0, 128.2, 66.3, 41.5, 35.8, 32.7, 29.4, 19.8.

HRMS (ESI$^+$): exact mass calculated for $[M+Na]^+$ ($C_{11}H_{14}O_2SNa^+$) requires 233.0607, found 233.0606.

IR (neat) ν_{max}: 3415, 2931, 1647, 1516, 1448, 1413, 1268, 1121, 979, 942, 764.

34: *anti*-(3-hydroxycyclohexyl)(naphthalen-2-yl)methanone

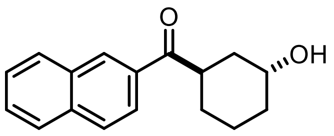

$C_{17}H_{18}O_2$
MW: 254.33
Yield: 68%, >20:1 r.r. (δ)
>20:1 d.r.

Synthesised following the general procedure, using cyclohexene (20.3 µL, 0.20 mmol, 1.00 equiv.), 2-naphthoyl chloride (40.0 mg, 0.21 mmol, 1.05 equiv.), silver hexafluoroantimonate (75.6 mg, 0.22 mmol, 1.10 equiv.), DMSO (142 µL, 2.00 mmol, 10.0 equiv.), tetrabutylammonium bromide (129 mg, 0.40 mmol, 2.00 equiv.) and dichloromethane (2 mL). Purification by flash column chromatography (heptane/ethyl acetate 80:20 to 50:50, Rf = 0.19 in heptane/ethyl acetate 75:25) gave the title compound (>20:1 r.r., >20:1 d.r., 34.6 mg, 68%) as a colourless oil.

^{1}H NMR (400 MHz, $CDCl_3$): *δ* 8.49 (s, 1H), 8.02 (dd, *J* = 8.6, 1.6 Hz, 1H), 7.96 (d, *J* = 8.0 Hz, 1H), 7.92 – 7.81 (m, 2H), 7.64 – 7.45 (m, 2H), 4.28 (d, *J* = 2.6 Hz, 1H), 3.97 (tt, *J* = 10.9, 3.5 Hz, 1H), 2.07 – 1.82 (m, 4H), 1.76 (ddd, *J* = 26.4, 16.0, 8.7 Hz, 1H), 1.69 – 1.54 (m, 4H).

^{13}C NMR (101 MHz, $CDCl_3$): *δ* 203.9, 135.6, 133.5, 132.7, 130.0, 129.7, 128.6, 128.5, 127.8, 126.8, 124.4, 66.4, 39.8, 35.7, 32.8, 29.2, 19.9.

HRMS (ESI$^+$): exact mass calculated for $[M+H]^+$ ($C_{17}H_{19}O_2$) requires *m/z* 255.1380, found *m/z* 255.1379.

IR (neat) ν_{max}: 3421, 3058, 2930, 2859, 2247, 1667, 1626, 1595, 1577, 1507, 1467, 1449, 1434, 1378, 1350, 1279, 1251, 1228, 1173, 1117, 1054, 974, 942, 907, 862, 821, 790 cm^{-1}.

35: *anti*-(4-fluorophenyl)(3-hydroxycyclohexyl)methanone

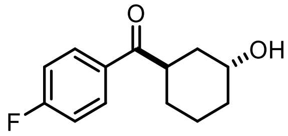

$C_{13}H_{15}FO_2$
MW: 222.26
Yield: 71%, >20:1 r.r. (δ)
>20:1 d.r.

Synthesised following the general procedure, using cyclohexene (20.3 µL, 0.20 mmol, 1.00 equiv.), 4-fluorobenzoyl chloride (24.8 µL, 0.21 mmol, 1.05 equiv.), silver hexafluoroantimonate (75.6 mg, 0.22 mmol, 1.10 equiv.), DMSO (142 µL, 2.00 mmol, 10.0 equiv.), tetrabutylammonium bromide (129 mg, 0.40 mmol, 2.00 equiv.) and dichloromethane (2 mL). Purification by flash column chromatography (heptane/ethyl acetate 80:20 to 50:50, Rf = 0.27 in heptane/ethyl acetate 66:33) gave the title compound (>20:1 r.r., >20:1 d.r., 31.6 mg, 71%) as a colourless oil.

^{1}H NMR (400 MHz, $CDCl_3$): *δ* 7.99 (dd, *J* = 8.5, 5.6 Hz, 2H), 7.11 (t, *J* = 8.6 Hz, 2H), 4.25 (brs, 1H), 3.76 (ddd, *J* = 10.9, 7.0, 3.7 Hz, 1H), 1.95 – 1.68 (m, 5H), 1.67 – 1.44 (m, 4H).

^{13}C NMR (151 MHz, $CDCl_3$): *δ* 200.1, 166.7 (d, *J* = 254.6 Hz), 132.5 (d, *J* = 3.0 Hz), 131.1 (d, *J* = 9.0 Hz, 2C), 115.9 (d, *J* = 21.8 Hz, 2C), 70.1, 44.1, 37.8, 35.4, 28.7, 23.5.

^{19}F NMR (377 MHz, $CDCl_3$): *δ* -105.69.

HRMS (ESI$^+$): exact mass calculated for $[M+Na]^+$ ($C_{13}H_{15}O_2FNa$) requires *m/z* 245.0948, found *m/z* 245.0948.

IR (neat) ν_{max}: 3489, 2930, 2861, 1713, 1668, 1595, 1505, 1433, 1409, 1376, 1315, 1298, 1285, 1268, 1229, 1156, 1121, 1056, 1029, 1012, 984, 926, 842, 817 cm^{-1}.

36: *anti*-(3-hydroxycyclopentyl)(phenyl)methanone

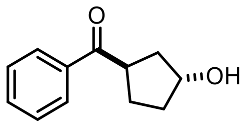

$C_{12}H_{14}O_2$
MW: 190.24
Yield: 92%
>20:1 d.r.

Synthesised following the general procedure, using cyclopentene (17.7 µL, 0.20 mmol, 1.00 equiv.), benzoyl chloride (24.4 µL, 0.21 mmol, 1.05 equiv.), silver hexafluoroantimonate (75.6 mg, 0.22 mmol, 1.10 equiv.), DMSO (142 µL, 2.00 mmol, 10.0 equiv.), tetrabutylammonium bromide (129 mg, 0.40 mmol, 2.00 equiv.) and dichloromethane (2 mL). Purification by flash column chromatography (heptane/ethyl acetate 80:20 to 50:50, Rf = 0.23 in heptane/ethyl acetate 66:33) gave the title compound (>20:1 d.r., 35.0 mg, 92%) as a pale-yellow oil.

^{1}H NMR (400 MHz, $CDCl_3$) δ 8.06 – 7.93 (m, 2H), 7.55 (t, J = 7.3 Hz, 1H), 7.46 (t, J = 7.6 Hz, 2H), 4.55 – 4.48 (m, 1H), 4.09 – 3.90 (m, 1H), 2.31 – 2.14 (m, 2H), 2.03 – 1.86 (m, 3H), 1.81 – 1.66 (m, 2H).

^{13}C NMR (101 MHz, $CDCl_3$) δ 202.4, 136.7, 133.1, 128.7 (2C), 128.6 (2C), 74.1, 44.3, 38.9, 35.2, 27.8.

HRMS (ESI$^+$): exact mass calculated for $[M+Na]^+$ ($C_{12}H_{14}O_2Na$) requires *m/z* 213.0886, found *m/z* 213.0880.

IR (neat) ν_{max}: 3405, 2939, 1675, 1596, 1579, 1447, 1358, 1312, 1219, 1179, 1072, 1010, 972, 938, 849, 754.

37: *anti*-(3-hydroxycyclohexyl)(4-(trifluoromethoxy)phenyl)methanone

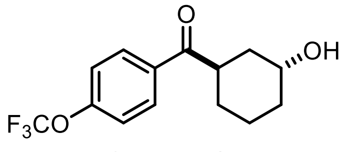

Synthesised following the general procedure, using cyclohexene (20.3 µL, 0.20 mmol, 1.00 equiv.), 4-(trifluoromethoxy)benzoyl chloride (33.2 µL, 0.21 mmol, 1.05 equiv.), silver hexafluoroantimonate (75.6 mg, 0.22 mmol, 1.10 equiv.), DMSO (142 µL, 2.00 mmol, 10.0 equiv.), tetrabutylammonium bromide (129 mg, 0.40 mmol, 2.00 equiv.) and dichloromethane (2 mL). Purification by flash column chromatography (heptane/ethyl acetate 80:20 to 50:50, Rf = 0.39 in heptane/ethyl acetate 50:50) gave the title compound (>20:1 r.r., >20:1 d.r., 47.9 mg, 83%) as a colourless oil.

^{1}H NMR (400 MHz, $CDCl_3$): *δ* 8.01 (d, *J* = 8.6 Hz, 2H), 7.27 (d, *J* = 8.7 Hz, 2H), 4.25 (app s, 1H), 3.76 (tt, *J* = 11.1, 3.3 Hz, 1H), 2.00 – 1.70 (m, 5H), 1.68 – 1.57 (m, 3H), 1.51 (ddd, *J* = 24.6, 12.8, 3.6 Hz, 1H).

^{13}C NMR (101 MHz, $CDCl_3$): *δ* 202.4, 152.6, 134.5, 130.5 (2C), 120.6 (2C), 120.5 (q, *J* = 258.9 Hz), 66.2, 39.9, 35.5, 32.8, 28.9, 19.8.

^{19}F NMR (377 MHz, $CDCl_3$): *δ* -57.63.

HRMS (ESI$^+$): exact mass calculated for $[M+Na]^+$ ($C_{14}H_{15}O_3F_3Na$) requires *m/z* 311.0866, found *m/z* 311.0865.

IR (neat) ν_{max}: 3427, 2935, 2863, 1713, 1682, 1602, 1587, 1505, 1451, 1434, 1413, 1377, 1312, 1302, 1254, 1204, 1154, 1120, 1107, 1056, 1032, 1016, 967, 925, 907, 890, 861, 844, 813, 795 cm^{-1}.

83: *anti*-(3-hydroxycyclohexyl)(phenyl)methanone

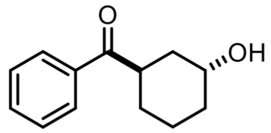

$C_{13}H_{16}O_2$
MW: 204.27
Yield: 78%, >20:1 r.r. (δ)
>20:1 d.r.

Synthesised following the general procedure, using cyclohexene (20.3 µL, 0.20 mmol, 1.00 equiv.), benzoyl chloride (24.4 µL, 0.21 mmol, 1.05 equiv.), silver hexafluoroantimonate (75.6 mg, 0.22 mmol, 1.10 equiv.), DMSO (142 µL, 2.00 mmol, 10.0 equiv.), tetrabutylammonium bromide (129 mg, 0.40 mmol, 2.00 equiv.) and dichloromethane (2 mL). Purification by flash column chromatography (heptane/ethyl acetate 80:20 to 50:50, Rf = 0.26 in heptane/ethyl acetate 66:33) gave the title compound (>20:1 r.r., >20:1 d.r., 31.8 mg, 78%) as a pale-yellow oil.

^{1}H NMR (400 MHz, $CDCl_3$): *δ* 7.97 – 7.85 (m, 2H), 7.62 – 7.51 (m, 1H), 7.44 (dd, *J* = 10.4, 4.7 Hz, 2H), 4.38 – 4.17 (m, 1H), 3.80 (tt, *J* = 10.9, 3.5 Hz, 1H), 1.97 – 1.84 (m, 2H), 1.84 – 1.70 (m, 3H), 1.68 – 1.58 (m, 2H), 1.58 – 1.46 (m, 2H).

^{13}C NMR (101 MHz, $CDCl_3$): *δ* 204.0, 136.2, 133.0, 128.8 (2C), 128.5 (2C), 66.3, 39.8, 35.6, 32.8, 29.0, 19.8.

HRMS (ESI$^+$): exact mass calculated for $[M+H]^+$ ($C_{13}H_{17}O_2$) requires *m/z* 205.1223, found *m/z* 205.1214.

IR (neat)ν_{max}: 3391, 2934, 2858, 1677, 1593, 1445, 1360, 1263, 1234, 1201, 1182, 1133 cm^{-1}.

3.5. Synthesis using different nucleophiles

3.5.1. General Procedure 4 for the synthesis using different nucleophiles

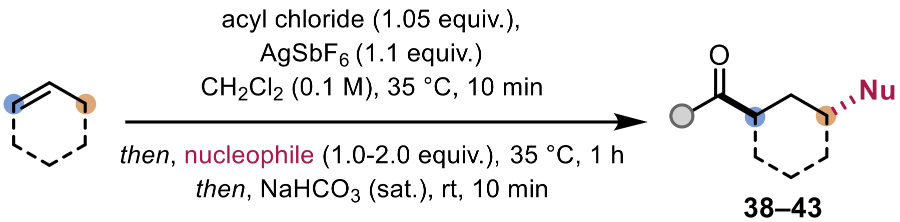

38–43

All reactions were performed on a 0.2 mmol scale.

In a flame-dried Schlenk tube or oven-dried vial charged with a magnetic stir bar, a solution of alkene (1.00 equiv.) and acyl chloride (1.05 equiv.) in dichloromethane (0.1 M) was treated with silver hexafluoroantimonate (1.10 equiv.) under argon atmosphere and immediately placed in a pre-heated oil (or sand) bath at 35 °C. After vigorously stirring the reaction mixture at the same temperature for 10 min, the stirring was stopped and the supernatant was transferred using a syringe into an oven-dried vial or flame-dried Schlenk tube. Then the corresponding nucleophile (1.00–2.00 equiv.) was added, and the reaction mixture was vigorously stirred at 35 °C for 1 h. Then, the reaction vessel was removed from the oil (or sand) bath and a saturated aqueous solution of sodium bicarbonate (equal volume to that of dichloromethane) was immediately added, followed by vigorous stirring for 10 min. After this time, the phases were separated, the aqueous phase was extracted with dichloromethane (3 x 5 mL) and the combined organic phases were dried over anhydrous magnesium sulfate, filtered and the filtrate was concentrated under reduced pressure. The resulting crude material was purified by flash column chromatography on silica gel (heptane/ethyl acetate) to give the title compounds **38–43**.

38: *anti*-(3-chlorocyclohexyl)(phenyl)methanone

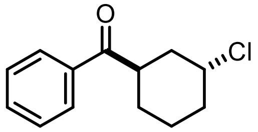

$C_{13}H_{15}ClO$
MW: 222.71
Yield: 83%, >20:1 r.r. (δ)
>20:1 d.r.

Synthesised following the general procedure, using cyclohexene (20.3 µL, 0.20 mmol, 1.00 equiv.), benzoyl chloride (24.4 µL, 0.21 mmol, 1.05 equiv.), silver hexafluoroantimonate (75.6 mg, 0.22 mmol, 1.10 equiv.), dichloromethane (2 mL), and tetrabutylammonium chloride (61.1 mg, 0.22 mmol, 1.10 equiv.) as the nucleophile. Purification by flash column chromatography (heptane/ethyl acetate 95:5 to 50:50, Rf = 0.35 in heptane/ethyl acetate 95:5) gave the title compound (>20:1 r.r., >20:1 d.r., 37.9 mg, 83%) as a colourless oil.

^{1}H NMR (600 MHz, $CDCl_3$): *δ* 8.02 – 7.93 (m, 2H), 7.61 – 7.53 (m, 1H), 7.47 (t, *J* = 7.7 Hz, 2H), 4.65 (dd, *J* = 6.8, 3.4 Hz, 1H), 3.89 (tt, *J* = 11.1, 3.3 Hz, 1H), 2.21 – 2.13 (m, 1H), 2.08 – 1.92 (m, 4H), 1.86 – 1.78 (m, 1H), 1.74 – 1.66 (m, 1H), 1.58 – 1.47 (m, 1H).

^{13}C NMR (151 MHz, $CDCl_3$): *δ* 203.0, 136.0, 133.2, 128.9 (2C), 128.5 (2C), 59.4, 39.8, 36.8, 33.7, 28.8, 20.0.

HRMS (ESI$^+$): exact mass calculated for $[M+H]^+$ ($C_{13}H_{16}{}^{35}ClO$) requires *m/z* 223.0884, found *m/z* 223.0884.

IR (neat) ν_{max}: 3060, 2940, 2862, 1677, 1596, 1579, 1447, 1430, 1375, 1339, 1322, 1268, 1224, 1201, 1175, 1138, 1098, 1076, 1020, 1001, 968, 910, 862, 818, 791 cm^{-1}.

39: *anti*–(3-bromocyclohexyl)(phenyl)methanone

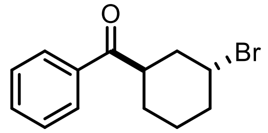

$C_{13}H_{15}BrO$
MW: 267.17
Yield: 47%, >20:1 r.r. (δ)
>20:1 d.r.

Synthesised following the general procedure, using cyclohexene (20.3 μL, 0.20 mmol, 1.00 equiv.), benzoyl chloride (24.4 μL, 0.21 mmol, 1.05 equiv.), silver hexafluoroantimonate (75.6 mg, 0.22 mmol, 1.10 equiv.), dichloromethane (2 mL), and tetrabutylammonium bromide (70.9 mg, 0.22 mmol, 1.10 equiv.) as the nucleophile. Purification by flash column chromatography (heptane/ethyl acetate 95:5 to 50:50, Rf = 0.36 in heptane/ethyl acetate 95:5) gave the title compound (>20:1 r.r., >20:1 d.r., 24.9 mg, 47%) as a colourless oil.

^{1}H NMR (600 MHz, $CDCl_3$) δ 7.98 (d, *J* = 7.4 Hz, 2H), 7.57 (t, *J* = 7.4 Hz, 1H), 7.48 (t, *J* = 7.7 Hz, 2H), 4.90 – 4.73 (m, 1H), 3.93 (tt, *J* = 11.0, 3.3 Hz, 1H), 2.32 – 2.21 (m, *J* = 14.5 Hz, 1H), 2.13 – 2.04 (m, 2H), 2.02 – 1.93 (m, 1H), 1.88 – 1.80 (m, 1H), 1.76 – 1.69 (m, 1H), 1.61 – 1.52 (m, 2H, overlaps with water peak).

^{13}C NMR (151 MHz, $CDCl_3$) δ 202.9, 136.0, 133.3, 128.9 (2C), 128.6 (2C), 53.9, 40.7, 37.5, 34.6, 28.8, 20.9.

HRMS (ESI$^+$): exact mass calculated for $[M+Na]^+$ ($C_{13}H_{15}{}^{79}BrONa^+$) requires 289.0198, found 289.0196.

IR (neat) ν_{max}: 3061, 2924, 1679, 1596, 1579, 1375, 1320, 1279, 1215, 967, 697 cm^{-1}.

40: *anti*-(3-iodocyclohexyl)(phenyl)methanone

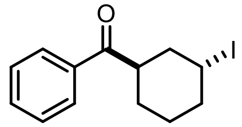

$C_{13}H_{15}IO$
MW: 314.17
Yield: 57%, >20:1 r.r. (δ)
13:1 d.r.

Synthesised following the general procedure, using cyclohexene (20.3 µL, 0.20 mmol, 1.00 equiv.), benzoyl chloride (24.4 µL, 0.21 mmol, 1.05 equiv.), silver hexafluoroantimonate (75.6 mg, 0.22 mmol, 1.10 equiv.), dichloromethane (2 mL), and tetrabutylammonium iodide (81.3 mg, 0.22 mmol, 1.1 equiv.) as the nucleophile. Purification by flash column chromatography (heptane/ethyl acetate 95:5 to 50:50, Rf = 0.39 in heptane/ethyl acetate 95:5) gave the title compound (>20:1 r.r., 13:1 d.r., 35.5 mg, 57%) as a colourless oil.

Major diastereomer:

^{1}H NMR (600 MHz, $CDCl_3$) δ 8.02–7.95 (m, 2H), 7.59–7.53 (m, *J* = 7.4 Hz, 1H), 7.50–7.46 (m, *J* = 7.6 Hz, 2H), 5.04–4.93 (m, 1H), 3.88 (tt, *J* = 11.0, 3.2 Hz, 1H), 2.28 (d, *J* = 14.7 Hz, 1H), 2.14–2.07 (m, 1H), 1.98–1.89 (m, 2H), 1.83 (ddd, *J* = 14.4, 10.9, 3.2 Hz, 1H), 1.77–1.72 (m, 1H), 1.65–1.57 (m, 2H).

^{13}C NMR (151 MHz, $CDCl_3$) δ 202.8, 135.9, 133.2, 128.9 (2C), 128.5 (2C), 42.3, 39.1, 36.2, 34.8, 28.8, 22.4.

Minor diastereomer:

^{1}H NMR (600 MHz, $CDCl_3$) δ 7.93–7.89 (m, *J* = 8.3 Hz, 2H), 7.57 (t, *J* = 7.4 Hz, 1H), 7.48 (t, *J* = 7.7 Hz, 2H), 4.24 (tt, *J* = 12.4, 3.9 Hz, 1H), 3.36 (tt, *J* = 11.8, 3.2 Hz, 1H), 2.60 (d, *J* = 13.2 Hz, 1H), 2.49 (d, *J* = 13.1 Hz, 1H), 2.20 (d, *J* = 12.6 Hz, 1H), 2.05–1.99 (m, 2H), 1.97 (d, *J* = 1.7 Hz, 1H), 1.79–1.77 (m, 1H), 1.56–1.48 (m, 1H).

^{13}C NMR (151 MHz, $CDCl_3$) δ 200.9, 135.8, 133.3, 128.9 (2C), 128.4 (2C), 47.7, 42.5, 40.0, 28.1, 28.0, 27.0.

HRMS (ESI$^+$): exact mass calculated for $[M+Na]^+$ ($C_{13}H_{15}IONa^+$) requires *m/z* 337.0060, found *m/z* 337.0060.

IR (neat) ν_{max}: 2935, 1677, 1446, 1275, 1261, 1206, 1006, 964 cm^{-1}.

41: *anti*-3-benzoylcyclohexyl acetate

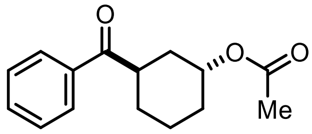

$C_{15}H_{18}O_3$
MW: 246.31
Yield: 66%, 13:1 r.r. (δ)
>20:1 d.r.

Synthesised following the general procedure, using cyclohexene (20.3 µL, 0.20 mmol, 1.00 equiv.), benzoyl chloride (24.4 µL, 0.21 mmol, 1.05 equiv.), silver hexafluoroantimonate (75.6 mg, 0.22 mmol, 1.10 equiv.), dichloromethane (2 mL), and *N*,*N*-dimethylacetamide (186 µL, 2.00 mmol, 10.0 equiv.) as the nucleophile. Purification by flash column chromatography (heptane/ethyl acetate 95:5 to 50:50, Rf = 0.28 in heptane/ethyl acetate 90:10) gave the title compound (13:1 r.r., >20:1 d.r., 32.4 mg, 66%) as a colourless oil.

^{1}H NMR (600 MHz, $CDCl_3$): *δ* 7.96 – 7.92 (m, 2H), 7.58 – 7.53 (m, 1H), 7.50 – 7.43 (m, 2H), 5.28 – 5.22 (m, 1H), 3.67 (tt, *J* = 11.5, 3.5 Hz, 1H), 2.12 (s, 3H), 2.01 (dddd, *J* = 14.3, 5.7, 3.8, 2.0 Hz, 1H), 1.97 – 1.93 (m, 1H), 1.93 – 1.88 (m, 1H), 1.83 (ddd, *J* = 14.3, 11.6, 2.8 Hz, 1H), 1.78 – 1.65 (m, 2H), 1.57 – 1.45 (m, 2H).

^{13}C NMR (151 MHz, $CDCl_3$): *δ* 203.1, 170.5, 136.2, 133.1, 128.8 (2C), 128.4 (2C), 69.6, 40.3, 32.6, 29.6, 29.1, 21.6, 20.5.

HRMS (ESI$^+$): exact mass calculated for $[M+Na]^+$ ($C_{15}H_{18}O_3Na$) requires *m/z* 269.1148, found *m/z* 269.1146.

IR (neat) ν_{max}: 2939, 2862, 1733, 1680, 1597, 1580, 1448, 1375, 1236, 1207, 1180, 1146, 1113, 1018, 977, 960, 887, 830, 792 cm^{-1}.

42: *anti*-3-benzoylcyclohexyl formate

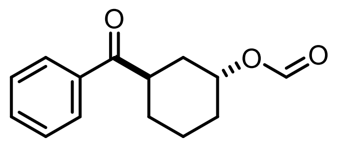

$C_{14}H_{16}O_3$
MW: 232.28
Yield: 68%, >20:1 r.r. (δ)
>20:1 d.r.

Synthesised following the general procedure, using cyclohexene (20.3 µL, 0.20 mmol, 1.00 equiv.), benzoyl chloride (24.4 µL, 0.21 mmol, 1.05 equiv.), silver hexafluoroantimonate (75.6 mg, 0.22 mmol, 1.10 equiv.), dichloromethane (2 mL), and dimethyl formamide (155 µL, 2.00 mmol, 10.0 equiv.) as the nucleophile. Purification by flash column chromatography (heptane/ethyl acetate 95:5 to 50:50, Rf = 0.46 in heptane/ethyl acetate 83:17) gave the title compound (>20:1 r.r., >20:1 d.r., 31.6 mg, 68%) as a colourless oil.

^{1}H NMR (700 MHz, $CDCl_3$): *δ* 8.14 (s, 1H), 7.94 (d, *J* = 7.4 Hz, 2H), 7.56 (t, *J* = 7.4 Hz, 1H), 7.46 (t, *J* = 7.7 Hz, 2H), 5.37 (app s, 1H), 3.70 (tt, *J* = 11.5, 3.4 Hz, 1H), 2.03 (d, *J* = 14.4 Hz, 1H), 1.95 (dd, *J* = 21.4, 11.2 Hz, 2H), 1.90 – 1.83 (m, 1H), 1.79 – 1.67 (m, 2H), 1.62 – 1.55 (m, 1H), 1.51 (ddd, *J* = 25.0, 12.6, 3.9 Hz, 1H).

^{13}C NMR (176 MHz, $CDCl_3$): *δ* 202.9, 160.6, 136.0, 133.2, 128.8 (2C), 128.4 (2C), 69.8, 40.1, 32.5, 29.6, 29.0, 20.3.

HRMS (ESI$^+$): exact mass calculated for $[M+Na]^+$ ($C_{14}H_{16}O_3Na$) requires *m/z* 255.0992, found *m/z* 255.0980.

IR (neat) ν_{max}: 2939, 2863, 1716, 1679, 1597, 1579, 1448, 1378, 1283, 1242, 1175, 1110, 1026, 1002, 975, 937, 885, 822, 792 cm^{-1}.

43: *anti*-phenyl(3-((2,2,6,6-tetramethylpiperidin-1-yl)oxy)cyclohexyl)methanone

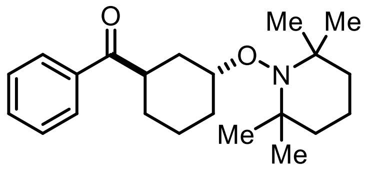

$C_{22}H_{33}NO_2$
MW: 343.51
Yield: 80%, >20:1 r.r. (δ)
>20:1 d.r.

Synthesised following the general procedure, using cyclohexene (20.3 µL, 0.20 mmol, 1.00 equiv.), benzoyl chloride (24.4 µL, 0.21 mmol, 1.05 equiv.), silver hexafluoroantimonate (75.6 mg, 0.22 mmol, 1.10 equiv.), dichloromethane (2 mL), and TEMPO (63.8 mg, 0.40 mmol, 2.00 equiv.) as the nucleophile. Purification by flash column chromatography (heptane/ethyl acetate 95:5 to 50:50, Rf = 0.24 in heptane/ethyl acetate 95:5) gave the title compound (>20:1 r.r., >20: d.r., 54.7 mg, 80%) as an orange oil. In contrast to other examples, the reaction with TEMPO reaction was conducted at 35 °C for 14 h.

^{1}H NMR (400 MHz, CDCl$_3$): *δ* 8.01 – 7.91 (m, 2H), 7.55 (t, *J* = 7.3 Hz, 1H), 7.46 (t, *J* = 7.5 Hz, 2H), 4.13 – 4.00 (m, 1H), 3.72 (tt, *J* = 10.9, 3.4 Hz, 1H), 2.16 (d, *J* = 13.5 Hz, 1H), 2.12 – 2.03 (m, 1H), 1.93 – 1.81 (m, 1H), 1.80 – 1.51 (m, 5H), 1.47 (app s, 4H), 1.43 – 1.34 (m, 2H), 1.20 (s, 6H), 1.15 (s, 6H).

^{13}C NMR (176 MHz, CDCl$_3$): *δ* 204.2, 136.5, 132.9, 128.7 (2C), 128.5 (2C), 78.1, 59.4 (2C, seen only in HMBC), 40.9, 40.5 (2C), 34.6 (2C, seen only in HSQC), 34.1, 30.3, 29.1, 20.9, 20.1 (2C, seen only in HSQC), 17.3.

HRMS (ESI$^+$): exact mass calculated for $[M+H]^+$ ($C_{22}H_{34}NO_2$) requires *m/z* 344.2584, found *m/z* 344.2572.

IR (neat) ν_{max}: 3058, 3000, 2970, 2930, 2867, 2359, 2339, 1717, 1681, 1597, 1580, 1541, 1447, 1374, 1360, 1337, 1299, 1279, 1258, 1237, 1209, 1180, 1158, 1132, 1117, 1082, 1044, 1028, 1001, 908, 879, 820, 792 cm^{-1}.

3.6. Synthesis of 1,4-dicarbonyls

3.6.1. General Procedure 5 for the synthesis of 1,4-dicarbonyls

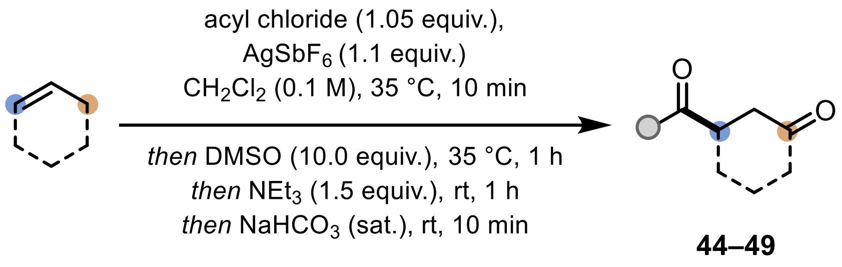

All reactions were performed on a 0.2 mmol scale.

In a flame-dried Schlenk tube or oven-dried vial charged with a magnetic stir bar, a solution of alkene (1.00 equiv.) and acyl chloride (1.05 equiv.) in dichloromethane (0.1 M) was treated with silver hexafluoroantimonate (1.10 equiv.) under argon atmosphere and the resulting suspension was immediately placed in a pre-heated oil (or sand) bath at 35 °C. After vigorously stirring the reaction mixture at the same temperature for 10 min, dimethyl sulfoxide (10.0 equiv.) was added and the reaction mixture was vigorously stirred for 1 h at 35 °C. Then, the reaction vessel was removed from the oil (or sand) bath and triethyl amine (1.50 equiv.) was immediately added. The reaction mixture was vigorously stirred for 1 h at room temperature, followed by the addition of a saturated aqueous solution of sodium bicarbonate (equal volume to that of dichloromethane), followed by vigorous stirring for 10 min. After this time, the phases were separated, the aqueous phase was extracted with dichloromethane (3 x 5 mL) and the combined organic phases were dried over anhydrous magnesium sulfate, filtered and the filtrate was concentrated under reduced pressure. The resulting crude material was purified by flash column chromatography on silica gel (heptane/ethyl acetate) to give the title compounds **44–49**. Where the products were formed as regioisomeric mixtures, this has been indicated.

44: 3-benzoylcyclohexan-1-one

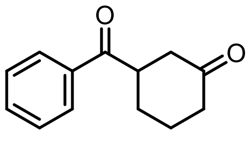

$C_{13}H_{14}O_2$
MW: 202.25
Yield: 81%, >20:1 r.r. (δ)

Synthesised following the general procedure, using cyclohexene (20.3 µL, 0.20 mmol, 1.00 equiv.), benzoyl chloride (24.4 µL, 0.21 mmol, 1.05 equiv.), silver hexafluoroantimonate (75.6 mg, 0.22 mmol, 1.10 equiv.), DMSO (142 µL, 2.00 mmol, 10.0 equiv.), triethylamine (41.8 µL, 0.30 mmol, 1.50 equiv.) and dichloromethane (2 mL). Purification by flash column chromatography (heptane/ethyl acetate 95:5 to 50:50, Rf = 0.28 in heptane/ethyl acetate 90:10) gave the title compound (>20:1 r.r., 32.7 mg, 81%) as a pale-yellow oil.

^{1}H NMR (400 MHz, $CDCl_3$): *δ* 8.03 – 7.86 (m, 2H), 7.59 (t, *J* = 7.4 Hz, 1H), 7.48 (t, *J* = 7.7 Hz, 2H), 3.81 (ddd, *J* = 14.2, 6.9, 3.6 Hz, 1H), 2.69 (app s, 1H), 2.48 (dd, *J* = 14.7, 4.3 Hz, 1H), 2.45 – 2.34 (m, 2H), 2.18 – 1.98 (m, 2H), 1.93 – 1.73 (m, 2H).

^{13}C NMR (101 MHz, $CDCl_3$): *δ* 210.4, 200.5, 135.5, 133.6, 129.0 (2C), 128.5 (2C), 45.3, 43.3, 41.1, 28.5, 25.0.

HRMS (ESI$^+$): exact mass calculated for $[M+H]^+$ ($C_{13}H_{15}O_2$) requires *m/z* 203.1067, found *m/z* 203.1066.

IR (neat) ν_{max}: 2944, 2868, 1709, 1676, 1596, 1580, 1448, 1420, 1375, 1346, 1315, 1262, 1220, 1178, 1106, 1011, 987, 959, 907, 883, 852, 754 cm^{-1}.

45: 3-(4-(trifluoromethyl)benzoyl)cyclohexan-1-one

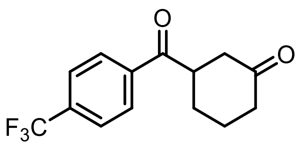

$C_{14}H_{13}F_3O_2$
MW: 270.25
Yield: 55%, >20:1 r.r. (δ)

Synthesised following the general procedure, using cyclohexene (20.3 μL, 0.20 mmol, 1.00 equiv.), benzoyl chloride (24.4 μL, 0.21 mmol, 1.05 equiv.), silver hexafluoroantimonate (75.6 mg, 0.22 mmol, 1.10 equiv.), DMSO (142 μL, 2.00 mmol, 10.0 equiv.), triethylamine (41.8 μL, 0.30 mmol, 1.50 equiv.) and dichloromethane (2 mL). Purification by flash column chromatography (heptane/ethyl acetate 95:5 to 50:50, Rf = 0.22 in heptane/ethyl acetate 75:25) gave the title compound (>20:1 r.r., 29.7 mg, 55%) as a pale-yellow oil.

^{1}H NMR (400 MHz, CDCl$_3$): *δ* 8.04 (d, *J* = 8.2 Hz, 2H), 7.75 (d, *J* = 8.2 Hz, 2H), 3.90 – 3.75 (m, 1H), 2.71 (dd, *J* = 14.6, 10.6 Hz, 1H), 2.54 – 2.35 (m, 3H), 2.18 – 2.02 (m, 2H), 1.95 – 1.75 (m, 2H).

^{13}C NMR (101 MHz, CDCl$_3$): *δ* 209.7, 199.7, 138.2, 134.9 (q, *J* = 32.8 Hz), 128.9 (2C), 126.1 (q, *J* = 3.7 Hz, 2C), 123.6 (q, *J* = 272.7 Hz), 45.6, 43.0, 41.1, 28.3, 24.8.

^{19}F NMR (377 MHz, CDCl$_3$): *δ* -63.20.

HRMS (ESI$^+$): exact mass calculated for $[M+H]^+$ ($C_{14}H_{14}F_3O_2$) requires *m/z* 271.0940, found *m/z* 271.0940.

IR (neat) ν$_{max}$: 3071, 2948, 2872, 2363, 1713, 1686, 1580, 1511, 1451, 1410, 1323, 1264, 1222, 1167, 1126, 1113, 1066, 1036, 1014, 989, 961, 908, 846, 794 cm^{-1}.

46: 3-benzoylcyclopentan-1-one

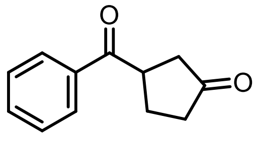

$C_{12}H_{12}O_2$
MW: 188.23
Yield: 74%

Synthesised following the general procedure, using cyclopentene (17.7 µL, 0.20 mmol, 1.00 equiv.), benzoyl chloride (24.4 µL, 0.21 mmol, 1.05 equiv.), silver hexafluoroantimonate (75.6 mg, 0.22 mmol, 1.10 equiv.), DMSO (142 µl, 2.00 mmol, 10.0 equiv.), triethylamine (41.8 µL, 0.30 mmol, 1.50 equiv.) and dichloromethane (2 mL). Purification by flash column chromatography (heptane/ethyl acetate 95:5 to 50:50, Rf = 0.22 in heptane/ethyl acetate 75:25) gave the title compound (27.7 mg, 74%) as a pale-yellow oil.

^{1}H NMR (400 MHz, CDCl$_3$): δ 8.00 (d, *J* = 7.3 Hz, 2H), 7.61 (t, *J* = 7.4 Hz, 1H), 7.50 (t, *J* = 7.6 Hz, 2H), 4.13 (app p, *J* = 7.5 Hz, 1H), 2.71 (dd, *J* = 18.4, 7.9 Hz, 1H), 2.50 – 2.24 (m, 4H), 2.23 – 2.06 (m, 1H).

^{13}C NMR (101 MHz, CDCl$_3$): δ 217.0, 200.4, 135.7, 133.7, 129.0 (2C), 128.6 (2C), 43.2, 41.1, 37.5, 27.1.

HRMS (ESI$^+$): exact mass calculated for $[M+H]^+$ ($C_{12}H_{13}O_2$) requires *m/z* 189.0910, found *m/z* 189.0908.

IR (neat) ν_{max}: 3061, 2969, 2363, 1737, 1673, 1596, 1579, 1448, 1403, 1365, 1264, 1221, 1158, 1133, 1074, 1005, 980, 950, 909, 883, 807, 777 cm^{-1}.

47: 3-(thiophene-2-carbonyl)cyclohexan-1-one

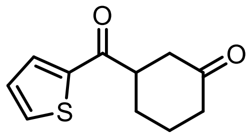

$C_{11}H_{12}O_2S$
MW: 208.28
Yield: 51%, >20:1 r.r. (δ)

Synthesised following the general procedure, using cyclohexene (20.3 µL, 0.20 mmol, 1.00 equiv.), thiophene-2-carbonyl chloride (22.7 µL, 0.21 mmol, 1.05 equiv.), silver hexafluoroantimonate (75.6 mg, 0.22 mmol, 1.10 equiv.), DMSO (142 µL, 2.00 mmol, 10.0 equiv.), triethylamine (41.8 µL, 0.30 mmol, 1.50 equiv.) and dichloromethane (2 mL). Purification by flash column chromatography (heptane/ethyl acetate 95:5 to 50:50, Rf = 0.18 in heptane/ethyl acetate 75:25) gave the title compound (>20:1 r.r., 21.3 mg, 51%) as a colourless solid.

^{1}H NMR (400 MHz, $CDCl_3$): *δ* 7.73 (d, *J* = 3.6 Hz, 1H), 7.68 (d, *J* = 4.9 Hz, 1H), 7.17 – 7.12 (m, 1H), 3.63 (tt, *J* = 10.7, 3.9 Hz, 1H), 2.72 (dd, *J* = 14.5, 11.0 Hz, 1H), 2.48 (dd, *J* = 14.7, 4.3 Hz, 1H), 2.44 – 2.32 (m, 2H), 2.21 – 2.05 (m, 2H), 2.02 – 1.76 (m, 2H).

^{13}C NMR (126 MHz, $CDCl_3$): *δ* 210.1, 193.3, 142.8, 134.7, 132.3, 128.5, 47.0, 43.3, 41.1, 28.9, 25.0.

HRMS (ESI$^+$): exact mass calculated for $[M+H]^+$ ($C_{11}H_{13}O_2S$) requires *m/z* 209.0631, found *m/z* 209.0630.

IR (neat) ν_{max}: 3090, 2946, 2869, 2361, 1710, 1656, 1518, 1449, 1414, 1375, 1352, 1316, 1265, 1239, 1223, 1201, 1180, 1146, 1106, 1081, 1059, 1040, 942, 896, 859, 842, 826, 757 cm^{-1}.

Melting point: 65-66 °C.

48: 3-pivaloylcyclohexan-1-one

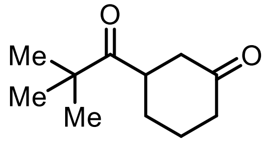

$C_{11}H_{18}O_2$
MW: 182.26
Yield: 38%, >20:1 r.r. (δ)

Synthesised following the general procedure, using cyclohexene (20.3 µL, 0.20 mmol, 1.00 equiv.), pivaloyl chloride (25.9 µL, 0.21 mmol, 1.05 equiv.), silver hexafluoroantimonate (75.6 mg, 0.22 mmol, 1.10 equiv.), DMSO (142 µL, 2.00 mmol, 10.0 equiv.), triethylamine (41.8 µL, 0.30 mmol, 1.50 equiv.) and dichloromethane (2 mL). Purification by flash column chromatography (heptane/ethyl acetate 95:5 to 50:50, Rf = 0.30 in heptane/ethyl acetate 83:17) gave the title compound (>20:1 r.r., 13.7 mg, 38%) as a colourless solid.

^{1}H NMR (400 MHz, $CDCl_3$): *δ* 3.32 (dtd, *J* = 10.3, 8.1, 4.0 Hz, 1H), 2.65 – 2.49 (m, 1H), 2.45 – 2.30 (m, 2H), 2.24 (ddd, *J* = 12.8, 3.9, 1.9 Hz, 1H), 2.11 (ddd, *J* = 13.9, 7.7, 4.2 Hz, 1H), 1.92 – 1.61 (m, 3H), 1.14 (s, 9H).

^{13}C NMR (101 MHz, $CDCl_3$): *δ* 215.8, 210.6, 44.9, 44.5, 44.4, 41.1, 28.8, 26.0 (3C), 25.4.

HRMS (ESI^{+}): exact mass calculated for $[M+H]^+$ ($C_{11}H_{19}O_2$) requires *m/z* 183.1380, found *m/z* 183.1379.

IR (neat) ν_{max}: 2956, 2871, 1703, 1479, 1367, 1225, 1064, 971, 739 cm^{-1}.

Melting point: 47-49 °C.

49: 3-(3-methylbenzoyl)cyclopentan-1-one

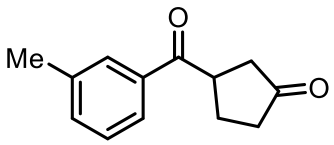

$C_{13}H_{14}O_2$
MW: 202.25
Yield: 64%

Synthesised following the general procedure, using cyclopentene (17.7 µL, 0.20 mmol, 1.00 equiv.), *m*-toluoyl chloride (27.7 µL, 0.21 mmol, 1.05 equiv.), silver hexafluoroantimonate (75.6 mg, 0.22 mmol, 1.10 equiv.), DMSO (142 µL, 2.00 mmol, 10.0 equiv.), triethylamine (41.8 µL, 0.30 mmol, 1.50 equiv.) and dichloromethane (2 mL). Purification by flash column chromatography (heptane/ethyl acetate 95:5 to 50:50, Rf = 0.29 in heptane/ethyl acetate 75:25) gave the title compound (25.9 mg, 64%) as a pale-yellow oil.

^{1}H NMR (400 MHz, $CDCl_3$): δ 7.78 (d, J = 9.2 Hz, 2H), 7.45 – 7.32 (m, 2H), 4.12 (dd, J = 15.1, 7.6 Hz, 1H), 2.70 (dd, J = 18.4, 7.9 Hz, 1H), 2.47 (d, J = 8.0 Hz, 1H), 2.43 (s, 3H), 2.42 – 2.23 (m, 3H), 2.21 – 2.10 (m, 1H).

^{13}C NMR (101 MHz, $CDCl_3$): δ 217.1, 200.6, 138.9, 135.8, 134.4, 129.1, 128.8, 125.8, 43.2, 41.2, 37.5, 27.2, 21.5.

HRMS (ESI$^+$): exact mass calculated for $[M+H]^+$ ($C_{13}H_{15}O_2$) requires *m/z* 203.1067, found *m/z* 203.1065.

IR (neat) ν_{max}: 3049, 2961, 2922, 1738, 1674, 1602, 1585, 1485, 1458, 1426, 1404, 1363, 1264, 1249, 1177, 1154, 1088, 1033, 1015, 981, 954, 912, 845, 805, 777 cm^{-1}.

3.7. Ipomeanol derivatives and trisubstituted alkene

50: 4-Ipomeanol

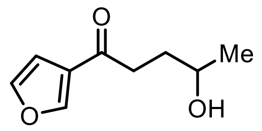

$C_9H_{12}O_3$
MW: 168.19
Yield: 73%, >20:1 r.r. (δ)

Synthesised following the general procedure, using 1-butene (74.0 µL, 9% in hexane, 0.20 mmol, 1.00 equiv.), 3-furoyl chloride (20.7 µL, 0.21 mmol, 1.05 equiv.), silver hexafluoroantimonate (75.6 mg, 0.22 mmol, 1.10 equiv.) and dichloromethane (2 mL). Purification by flash column chromatography (heptane/ethyl acetate 80:20 to 50:50, Rf = 0.36 in heptane/ethyl acetate 50:50) gave the title compound (>20:1 r.r., 24.1 mg, 73%) as a pale-yellow oil.

^{1}H NMR (400 MHz, CDCl$_3$): *δ* 8.15 – 7.96 (m, 1H), 7.48 – 7.39 (m, 1H), 6.77 (dd, *J* = 1.9, 0.8 Hz, 1H), 3.87 (dqd, *J* = 12.3, 6.2, 3.9 Hz, 1H), 2.91 (t, *J* = 7.1 Hz, 2H), 1.98 – 1.74 (m, 3H), 1.24 (d, *J* = 6.2 Hz, 3H).

^{13}C NMR (101 MHz, CDCl$_3$): *δ* 195.6, 147.4, 144.4, 127.8, 108.8, 67.6, 36.8, 33.1, 24.0.

HRMS (ESI$^+$): exact mass calculated for $[M+H]^+$ ($C_9H_{13}O_3$) requires *m/z* 169.0859, found *m/z* 169.0854.

IR (neat) ν_{max}: 3425, 3135, 2967, 2928, 1718, 1671, 1562, 1510, 1455, 1394, 1376, 1349, 1306, 1243, 1156, 1132, 1046, 1019, 995, 955, 932, 903, 873, 821, 795 cm^{-1}.

51: 4-hydroxy-1-phenylpentan-1-one

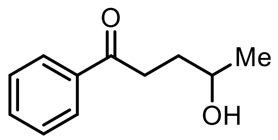

$C_{11}H_{14}O_2$
MW: 178.23
Yield: 83%, >20:1 r.r. (δ)

Synthesised following the general procedure, using 1-butene (148 µL, 9% in hexane, 0.40 mmol, 2.00 equiv.), benzoyl chloride (23.2 µL, 0.20 mmol, 1.00 equiv.), silver hexafluoroantimonate (75.6 mg, 0.22 mmol, 1.10 equiv.) and dichloromethane (2 mL). Purification by flash column chromatography (heptane/ethyl acetate 80:20 to 50:50, Rf = 0.27 in heptane/ethyl acetate 66:33) gave the title compound (>20:1 r.r., 29.7 mg, 83%) as a pale-yellow oil.

^{1}H NMR (400 MHz, $CDCl_3$): *δ* 8.01 – 7.91 (m, 2H), 7.55 (t, *J* = 7.4 Hz, 1H), 7.45 (t, *J* = 7.6 Hz, 2H), 4.00 – 3.74 (m, 1H), 3.22 – 2.97 (m, 2H), 1.95 (dtd, *J* = 11.1, 7.3, 4.1 Hz, 2H), 1.92 – 1.70 (m, 1H), 1.25 (d, *J* = 6.2 Hz, 3H).

^{13}C NMR (101 MHz, $CDCl_3$): *δ* 200.9, 137.0, 133.2, 128.7 (2C), 128.2 (2C), 67.7, 35.1, 33.2, 24.0.

HRMS (ESI$^+$): exact mass calculated for $[M+H]^+$ ($C_{11}H_{15}O_2$) requires *m/z* 179.1067, found *m/z* 179.1051.

IR (neat) ν_{max}: 3422, 3059, 2969, 2929, 1716, 1683, 1598, 1581, 1492, 1448, 1377, 1314, 1273, 1207, 1177, 1111, 1068, 1025, 911, 848, 759 cm^{-1}.

52: 7-hydroxy-2-methyloctan-4-one

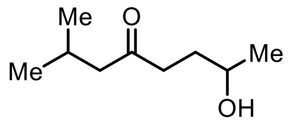

$C_9H_{18}O_2$
MW: 158.24
Yield: 56%, >20:1 r.r. (δ)

Synthesised following the general procedure, using 1-butene (74 µL, 9% in hexane, 0.20 mmol, 1.00 equiv.), isovaleryl chloride (25.3 µL, 0.21 mmol, 1.05 equiv.), silver hexafluoroantimonate (75.6 mg, 0.22 mmol, 1.10 equiv.) and dichloromethane (2 mL). Purification by flash column chromatography (heptane/ethyl acetate 90:10 to 50:50, Rf = 0.29 in heptane/ethyl acetate 50:50) gave the title compound (>20:1 r.r., 17.7 mg, 56%) as a colourless oil.

^{1}H NMR (400 MHz, CDCl$_3$): δ 3.79 (brs, 1H), 2.54 (t, J = 7.0 Hz, 2H), 2.31 (d, J = 7.0 Hz, 2H), 2.20 – 2.08 (m, 1H), 1.77 (dtd, J = 14.2, 7.2, 4.1 Hz, 2H), 1.66 (dt, J = 21.4, 7.0 Hz, 1H), 1.20 (d, J = 6.2 Hz, 3H), 0.91 (d, J = 6.6 Hz, 6H).

^{13}C NMR (101 MHz, CDCl$_3$): δ 211.8, 67.7, 52.1, 39.8, 32.7, 24.8, 23.9, 22.7, 22.7.

HRMS (ESI$^+$): exact mass calculated for $[M+Na]^+$ ($C_9H_{18}O_2Na$) requires m/z 181.1199, found m/z 181.1197.

IR (neat) ν_{max}: 3506, 3398, 2959, 2929, 2872, 1712, 1467, 1406, 1368, 1290, 1267, 1224, 1170, 1144, 1131, 1100, 1064, 1044, 1019, 962, 934, 740 cm^{-1}.

53: 4-hydroxy-1-(thiophen-2-yl)pentan-1-one

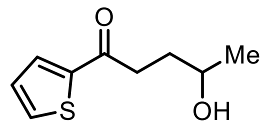

$C_9H_{12}O_2S$
MW: 184.25
Yield: 68%, >20:1 r.r. (δ)

Synthesised following the general procedure, using 1-butene (74.0 µL, 9% in hexane, 0.20 mmol, 1.00 equiv.), thiophene-2-carbonyl chloride (21.6 µL, 0.21 mmol, 1.05 equiv.), silver hexafluoroantimonate (75.6 mg, 0.22 mmol, 1.10 equiv.) and dichloromethane (2 mL). Purification by flash column chromatography (heptane/ethyl acetate 90:10 to 50:50, Rf = 0.39 in heptane/ethyl acetate 50:50) gave the title compound (>20:1 r.r., 25.2 mg, 68%) as a pale-yellow oil.

^{1}H NMR (400 MHz, $CDCl_3$): *δ* 7.75 (dd, *J* = 3.8, 1.1 Hz, 1H), 7.63 (dd, *J* = 5.0, 1.1 Hz, 1H), 7.12 (dd, *J* = 4.9, 3.8 Hz, 1H), 3.98 – 3.81 (m, 1H), 3.07 (t, *J* = 7.1 Hz, 2H), 1.95 (dtd, *J* = 11.2, 7.3, 3.9 Hz, 2H), 1.83 (ddt, *J* = 14.2, 8.2, 7.0 Hz, 1H), 1.24 (d, *J* = 6.2 Hz, 3H).

^{13}C NMR (101 MHz, $CDCl_3$): *δ* 193.8, 144.3, 133.8, 132.2, 128.3, 67.6, 35.8, 33.5, 23.9.

HRMS (ESI$^+$): exact mass calculated for $[M+Na]^+$ ($C_9H_{12}O_2SNa$) requires *m/z* 207.0450, found *m/z* 207.0449.

IR (neat) ν_{max}: 3422, 3088, 2966, 2871, 2847, 1712, 1662, 1649, 1455, 1413, 1374, 1355, 1284, 1268, 1235, 1094, 1081, 1058, 1037, 1017, 980, 953, 794 cm^{-1}.

54: ((1*R,2*R**,3*S**)-3-hydroxy-2-methylcyclohexyl)(phenyl)methanone**

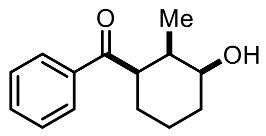

$C_{14}H_{18}O_2$
MW: 218.30
Yield: 40%, >20:1 r.r. (δ)

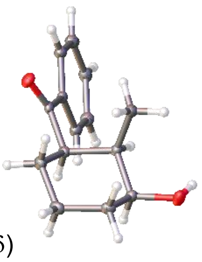

Synthesised following the general procedure, using 1-methyl-1-cyclohexene (24.2 µL, 0.20 mmol, 1.00 equiv.), benzoyl chloride (24.4 µL, 0.21 mmol, 1.05 equiv.), silver hexafluoroantimonate (75.6 mg, 0.22 mmol, 1.10 equiv.) and dichloromethane (2 mL). Purification by flash column chromatography (heptane/ethyl acetate 66:33, Rf = 0.23 in heptane/ethyl acetate 75:25) gave the title compound (>20:1 r.r., 17.6 mg, 40%) as a colourless solid.

^{1}H NMR (600 MHz, $CDCl_3$): *δ* 7.93 (dd, *J* = 8.3, 1.1 Hz, 2H), 7.59 – 7.54 (m, 1H), 7.46 (dd, *J* = 10.7, 4.9 Hz, 2H), 3.91 – 3.83 (m, 1H), 3.68 – 3.61 (m, 1H), 3.34 (app s, 1H), 2.29 – 2.21 (m, 1H), 1.89 – 1.80 (m, 1H), 1.75 – 1.57 (m, 4H), 1.43 – 1.34 (m, 1H), 0.86 (d, *J* = 7.1 Hz, 3H).

^{13}C NMR (151 MHz, $CDCl_3$): *δ* 203.6, 136.5, 133.3, 128.9 (2C), 128.6 (2C), 71.1, 47.7, 37.2, 31.3, 24.2, 19.7, 10.8.

HRMS (ESI$^+$): exact mass calculated for $[M+Na]^+$ ($C_{14}H_{18}O_2Na$) requires *m/z* 241.1199, found *m/z* 241.1197.

IR (neat) ν_{max}: 3296, 2971, 2852, 2364, 2326, 1751, 1718, 1680, 1668, 1597, 1579, 1470, 1447, 1326, 1268, 1229, 1126, 867, 765, 743 cm^{-1}.

Melting point: 117-118 °C.

3.8. Comparison with metal-catalysed 1,3-functionalisations (Pd and Ni)

Question: Are reported procedures capable of reagent controlled 1,3-functionalisations?

Answer 1. Reported procedure using *Ni-catalysis*:[3–5]

In the absence of substrate control – NO 1,3-functionalisation takes place.

1.1 Substrate-controlled 1,3-functionalisation (Benzylic position)

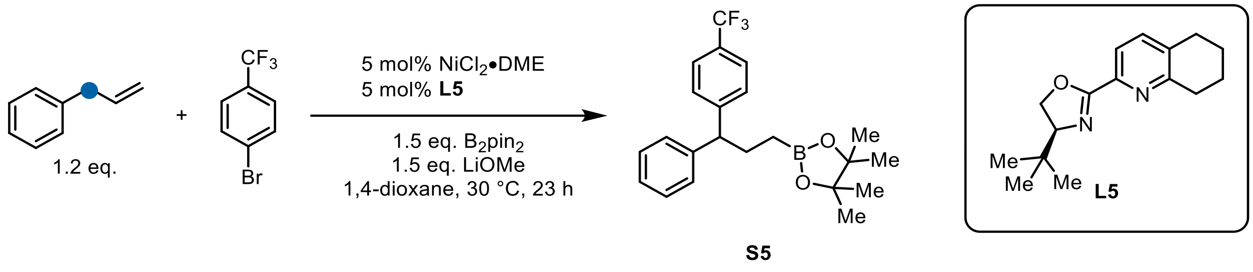

full conversion.
reported yield: 75%
isolated yield: 35%

1.2. Unfunctionalised alkene substrate:

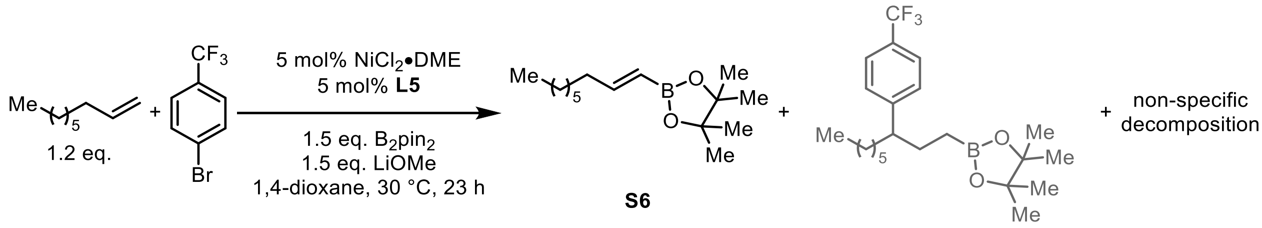

major product results from a Bora-Heck process

31% NMR yield

not detected in NMR or HRMS

hence no 1,3-functionalisation without benzylic position

Answer 2. Reported procedure using *Pd-catalysis*:[6]

In the absence of substrate control – NO 1,3-functionalisation takes place.

2.1. Alkene substrate-controlled 1,3-functionalisation (benzylic position and directing group)

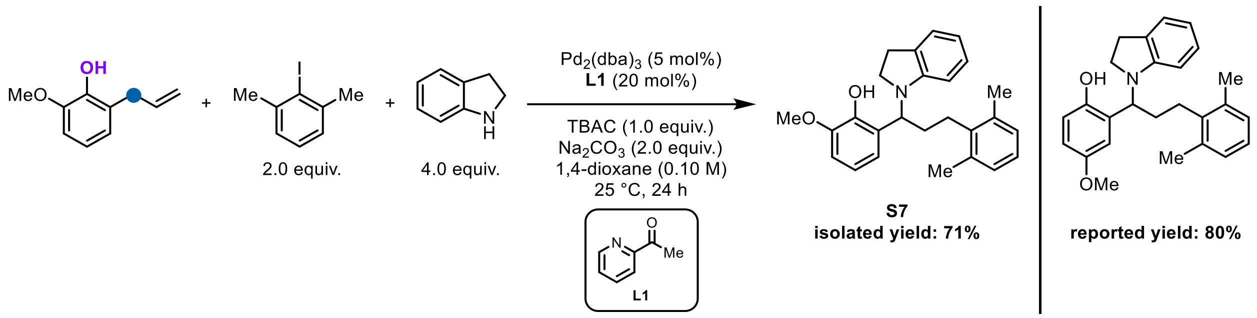

A longer alkyl chain leads to 1,4-functionalisation (benzylic position and directing group required)

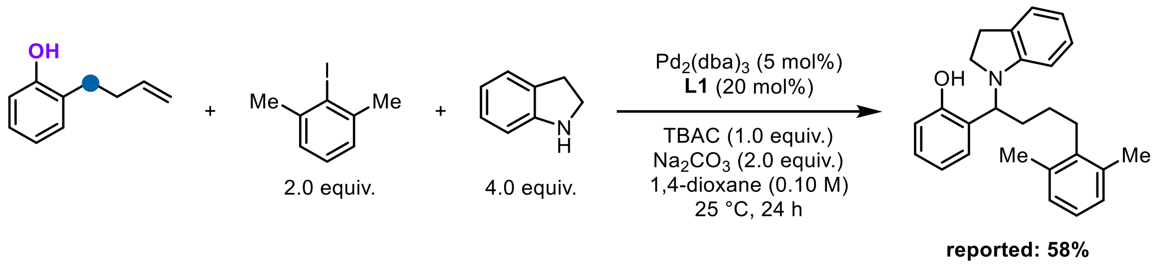

2.2. Alkene substrate not containing a directing group:

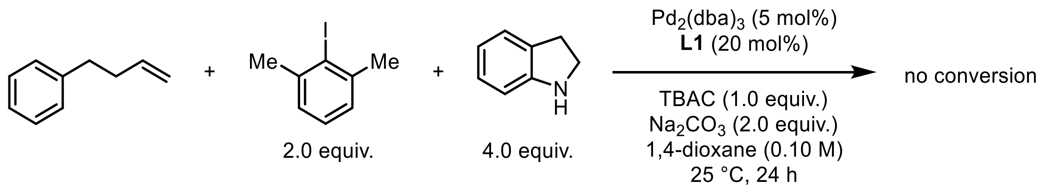

For less hindered aryl iodides, only Heck-type products were obtained.

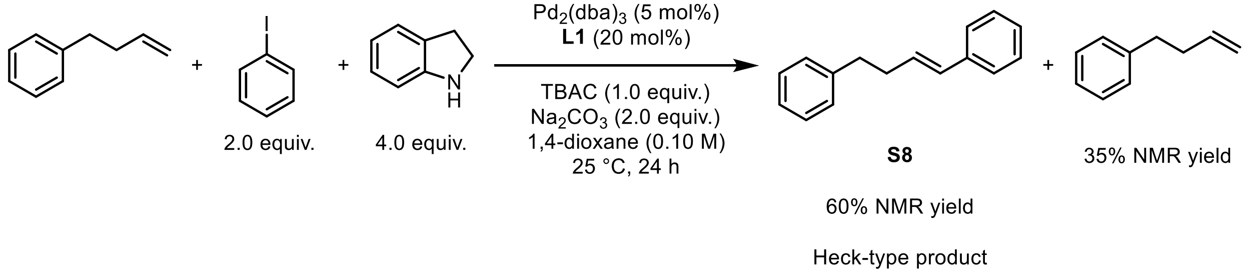

2.3. Unfunctionalised alkene substrates (no directing group and no benzylic position):

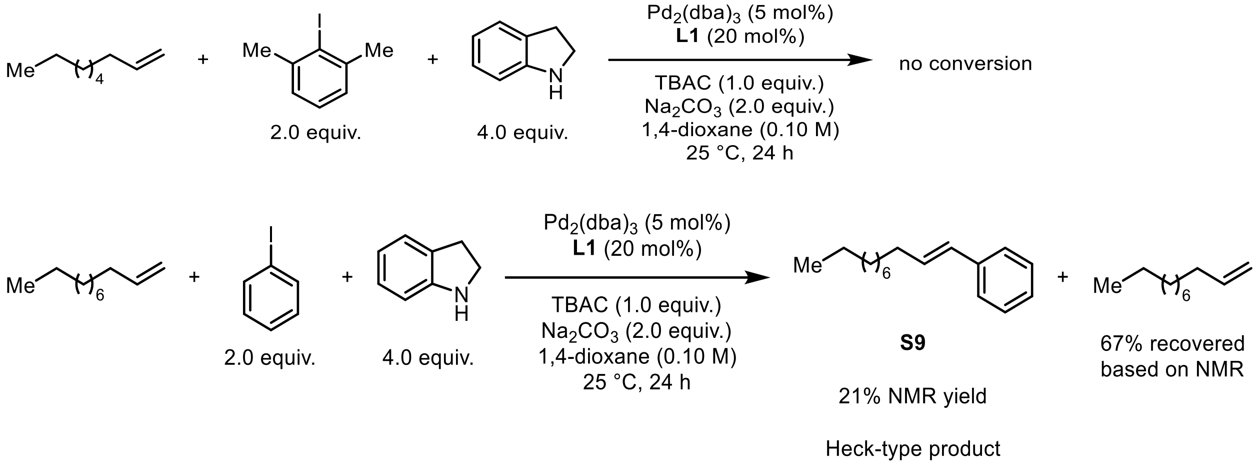

S5: 4,4,5,5-tetramethyl-2-(3-phenyl-3-(4-(trifluoromethyl)phenyl)propyl)-1,3,2-dioxaborolane

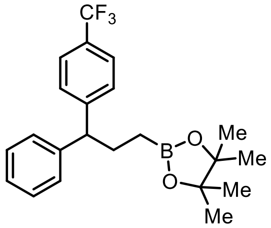

Spectroscopic data were in accordance with literature.[3]

^{1}H NMR (400 MHz, CDCl$_3$): δ 7.51 (d, J = 8.2 Hz, 2H), 7.35 (d, J = 8.1 Hz, 2H), 7.32 – 7.25 (m, 2H), 7.25 – 7.13 (m, 3H), 3.91 (t, J = 7.8 Hz, 1H), 2.16 (ddd, J = 9.8, 8.2, 2.5 Hz, 2H), 1.26 (s, 12H), 0.78 (td, J = 9.8, 6.6 Hz, 2H).

^{13}C NMR (101 MHz, CDCl$_3$): δ 149.3, 144.2, 128.7 (2C), 128.5 (2C), 128.1 (2C), 126.5, 125.4 (q, J = 3.8 Hz), 124.6 (q, J = 223.7 Hz), 83.2, 53.6, 29.9, 25.0 (4C).

S6: (*E*)-4,4,5,5-tetramethyl-2-(non-1-en-1-yl)-1,3,2-dioxaborolane

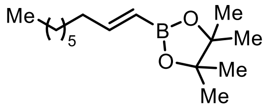

The spectroscopic data and purity of the isolated material were in good accordance with those reported in the literature.[7]

^{1}H NMR (400 MHz, CDCl$_3$): δ 6.63 (dt, J = 17.9, 6.4 Hz, 1H), 5.41 (dt, J = 18.0, 1.5 Hz, 1H), 2.13 (td, J = 7.9, 1.5 Hz, 2H), 1.40 (dd, J = 9.2, 4.9 Hz, 2H), 1.26 (s, 12H), 1.31 – 1.20 (m, 8H), 0.87 (t, J = 6.8 Hz, 3H).

S7: 2-(3-(2,6-dimethylphenyl)-1-(indolin-1-yl)propyl)-6-methoxyphenol

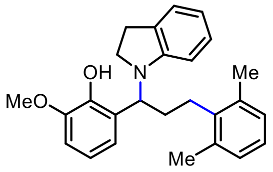

Structure determination based on comparison with previously reported products.[6]

^{1}H NMR (400 MHz, CDCl$_3$): δ 7.54 (s, 1H), 7.35 – 7.29 (m, 1H), 7.29 – 7.22 (m, 4H), 7.18 – 7.09 (m, 2H), 6.93 (td, *J* = 7.5, 0.8 Hz, 1H), 6.85 (d, *J* = 7.9 Hz, 1H), 5.16 (dd, *J* = 8.4, 5.9 Hz, 1H), 4.17 (s, 3H), 3.79 – 3.59 (m, 2H), 3.21 (dd, *J* = 12.1, 6.4 Hz, 2H), 3.08 – 2.96 (m, 1H), 2.92 – 2.82 (m, 1H), 2.52 (s, 6H), 2.47 – 2.29 (m, 3H).

^{13}C NMR (101 MHz, CDCl$_3$): δ 151.2, 147.4, 145.0, 138.9, 136.2 (2C), 130.5, 128.3 (2C), 127.4, 126.3, 125.9, 124.5, 120.1, 119.4, 118.3, 110.2, 108.8, 56.5, 56.2, 48.9, 30.0, 28.5, 27.4, 19.9 (2C).

HRMS (ESI$^+$): exact mass calculated for $[M+H]^+$ ($C_{26}H_{30}NO_2$) requires *m/z* 388.2271, found *m/z* 388.2270.

S8: (*E*)-but-1-ene-1,4-diyldibenzene

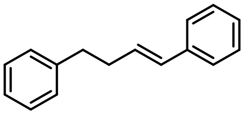

Due to the highly lipophilic character of the product, separation from its regioisomer was not possible using chromatographic methods. It was obtained as a 95:5 mixture with the external alkene (regioisomer) and the isolated material included small amounts of grease. Spectroscopic data in accordance with literature as reported below.[8]

^{1}H NMR (400 MHz, CDCl$_3$): δ 7.31-7.04 (m, 10H), 6.34 (d, *J* = 15.8 Hz, 1H), 6.19 (dt, *J* = 15.8, 6.8 Hz, 1H), 2.82 – 2.63 (m, 2H), 2.46 (dd, *J* = 14.6, 6.7 Hz, 2H).

^{13}C NMR (101 MHz, CDCl$_3$): δ 141.8, 137.7, 130.4, 130.0, 128.5 (4C), 128.4 (2C), 126.9, 126.0 (2C), 125.9, 35.9, 35.0.

S9: (*E*)-undec-1-en-1-ylbenzene

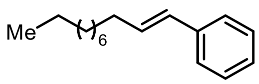

Structure determination based on comparison with previously reported products. [9-11] Due to the nature of the reaction, in accordance with

previous reports[9-11], the product was obtained as a mixture of isomers as supported also by GC analysis. The major isomer is reported below.

^{1}H NMR (400 MHz, CDCl$_3$) 7.38-7.25 (m, 5H), 6.38 (d, *J*= 15.8 Hz, 1H), 6.23 (dt, *J*= 15.8, 6.8 Hz, 1H), 2.20 (td, *J* = 8.0, 1.2 Hz, 2H), 1.41-1.27 (m, 14H), 0.97 – 0.81 (m, 3H).

^{13}C NMR (101 MHz, CDCl$_3$): δ 138.1, 131.4, 129.8, 128.6 (2C), 126.9, 126.0 (2C), 33.2, 32.1, 29.7, 29.7, 29.5, 29.5, 29.4, 22.8, 14.3.

GC analysis of compound S9

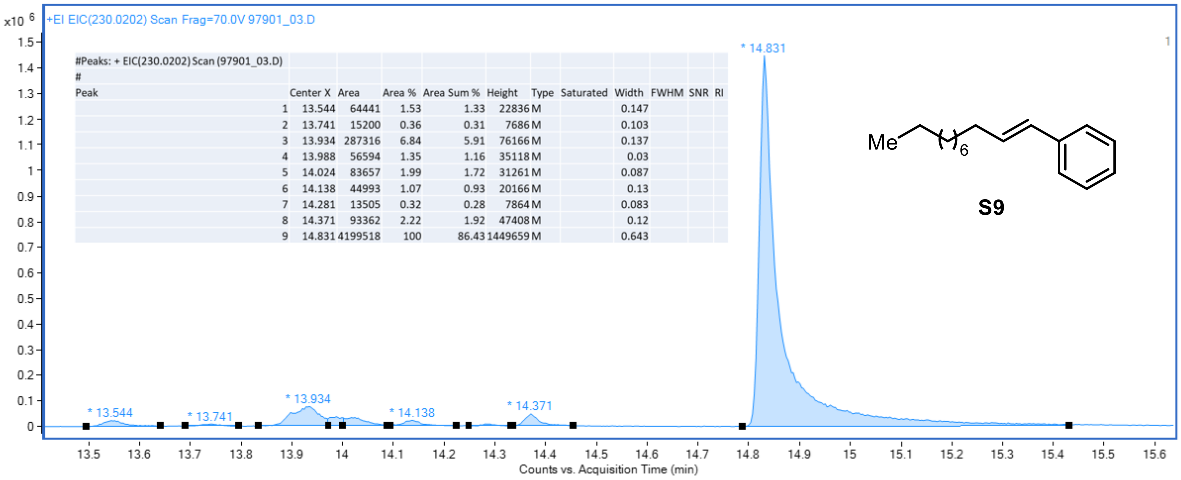

#Peaks: + EIC(230.0202) Scan (97901_03.D)											
#											
Peak	Center X	Area	Area %	Area Sum %	Height	Type	Saturated	Width	FWHM	SNR	RI
1	13.544	64441	1.53	1.33	22836	M		0.147			
2	13.741	15200	0.36	0.31	7686	M		0.103			
3	13.934	287316	6.84	5.91	76166	M		0.137			
4	13.988	56594	1.35	1.16	35118	M		0.03			
5	14.024	83657	1.99	1.72	31261	M		0.087			
6	14.138	44993	1.07	0.93	20166	M		0.13			
7	14.281	13505	0.32	0.28	7864	M		0.083			
8	14.371	93362	2.22	1.92	47408	M		0.12			
9	14.831	4199518	100	86.43	1449659	M		0.643			

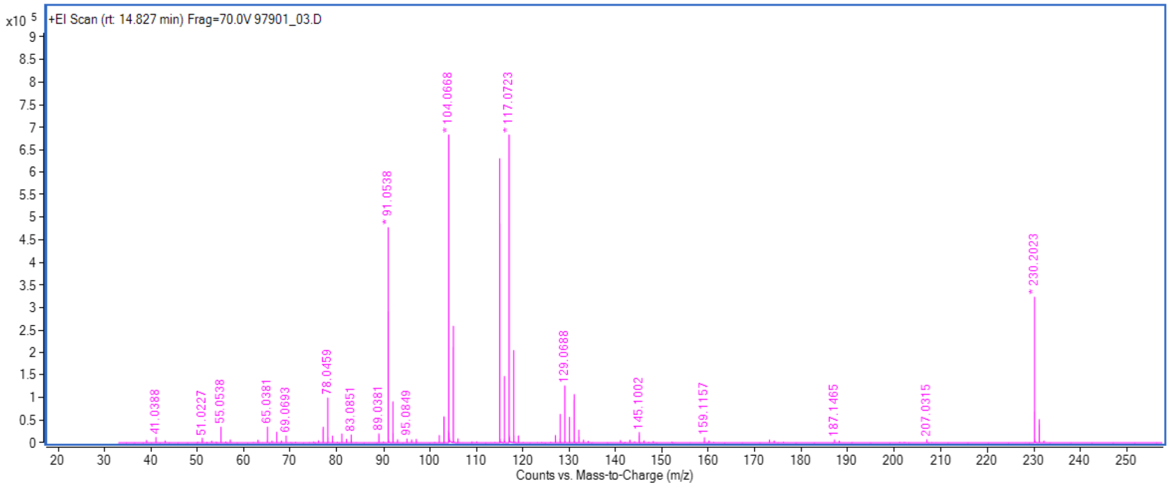

3.9. Mechanistic study: Demonstration of charge relocation

3.9.1. Synthesis of bromides

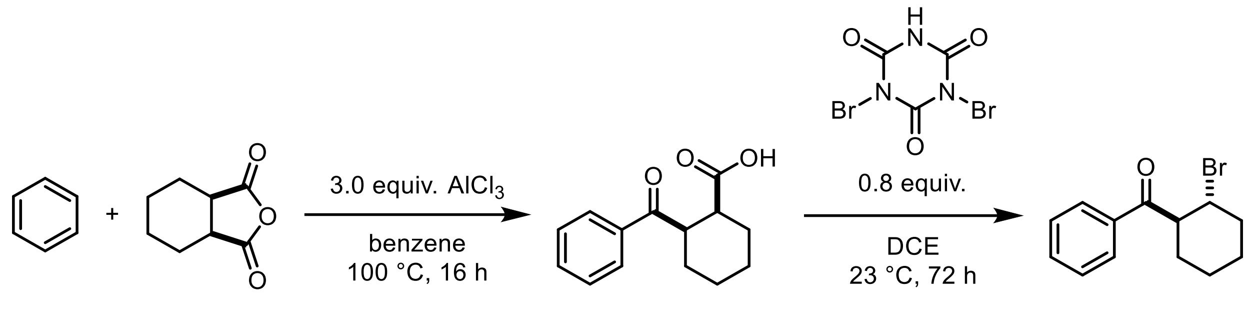

S10 **S11**

S10: *syn*-2-benzoylcyclohexane-1-carboxylic acid

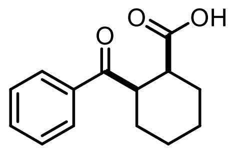

$C_{14}H_{16}O_3$
MW: 232.28

In a flame-dried Schlenk tube, aluminium trichloride (4.00 g, 30.0 mmol, 3.00 equiv.) was carefully added to hexahydro-1,3-isobenzofurandione (1.54 g, 10.0 mmol, 1.00 equiv.) in benzene (12 mL). The Schlenk tube was sealed and the mixture was stirred at 100 °C for 16 h. After allowing the reaction mixture to cool to ambient temperature, the reaction vessel was placed in an ice-water bath and 1N HCl (15 mL) was carefully added at 0 °C. The phases were subsequently separated and the aqueous phase was extracted with ethyl acetate (3 x 20 mL). The combined organic layers were treated with NaOH 1N (25 mL). The aqueous layer was acidified with HCl (37% w/w) until a pH of 1 was reached, subsequently extracted with chloroform (3x20 mL), dried over anhydrous sodium sulfate, filtered and concentrated *in vacuo*. The resulting crude material was purified by flash column chromatography on silica gel (heptane/ethyl acetate/acetic acid 50:50:1 to 0:100:1, Rf = 0.52 in heptane/ethyl acetate/acetic acid 50:50:1) to afford the desired compound as a beige solid (455 mg, 20%). The analytical data was in accordance with the literature.[12]

^{1}H NMR (400 MHz, $CDCl_3$): δ 11.40 (s, 1H), 7.84 (dd, *J* = 5.2, 3.4 Hz, 2H), 7.63 – 7.49 (m, 1H), 7.43 (dd, *J* = 10.4, 4.7 Hz, 2H), 3.90 (dd, *J* = 9.7, 4.9 Hz, 1H), 2.70 (dt, *J* = 9.4, 4.5 Hz, 1H), 2.23 (ddd, *J* = 16.0, 10.1, 5.1 Hz, 1H), 2.08 (dt, *J* = 12.5, 5.9 Hz, 1H), 1.99 – 1.91 (m, 1H), 1.79 (ddd, *J* = 17.6, 9.8, 4.6 Hz, 2H), 1.44 (dt, *J* = 12.1, 6.2 Hz, 1H), 1.40 – 1.20 (m, 2H).

^{13}C NMR (101 MHz, CDCl$_3$): δ 202.5, 180.3, 136.7, 132.7, 128.7 (2C), 128.4 (2C), 44.3, 42.8, 27.8, 25.3, 24.6, 22.6.

S11: *anti*-(2-bromocyclohexyl)(phenyl)methanone

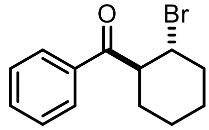

$C_{13}H_{15}BrO$
MW: 267.17

Compound **S11** was obtained as a 1:1 mixture of the two diastereomers, following a reported Hunsdiecker reaction procedure,[13] using compound **S10** (232 mg, 1.00 mmol, 1.00 equiv.) and dibromoisocyanuric acid (229 mg, 0.80 mmol, 0.80 equiv.). The resulting crude material was purified by flash column chromatography on silica gel (heptane/ethyl acetate 95:5 to 50:50, Rf = 0.36 in heptane/ethyl acetate 90:10) to afford the desired compound as a colourless oil (72 mg, 27%).

Note: The diastereomers were found to be separable by column chromatography. However, the *syn*-configured isomer decomposed at –20 °C during storage overnight. The analytical data was in accordance with the literature reported.[13]

^{1}H NMR (400 MHz, CDCl$_3$): δ 7.99 (d, *J* = 7.9 Hz, 2H), 7.59 (t, *J* = 7.2 Hz, 1H), 7.49 (t, *J* = 7.5 Hz, 2H), 4.45 (td, *J* = 11.2, 4.2 Hz, 1H), 3.82 (td, *J* = 10.8, 3.9 Hz, 1H), 2.50 (d, *J* = 14.1 Hz, 1H), 2.05 – 1.92 (m, 2H), 1.83 (d, *J* = 2.3 Hz, 2H), 1.52 – 1.34 (m, 3H).

^{13}C NMR (176 MHz, CDCl$_3$): δ 201.2, 136.5, 133.5, 128.9 (2C), 128.6 (2C), 54.1, 51.6, 37.5, 32.1, 27.1, 24.9.

S12: (1-bromocyclohexyl)(phenyl)methanone

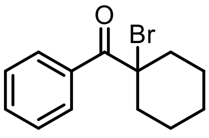

$C_{13}H_{15}BrO$
MW: 267.17
Yield: 81%

Compound **S12** was prepared using a previously reported procedure,[14] employing benzoylcyclohexane (1.90 g, 10.0 mmol, 1.00 equiv.), acetic acid (57.0 µL, 1.00 mmol, 0.10 equiv.), bromine (3.20 g, 20.0 mmol, 2.00 equiv.) and dichloromethane (15 mL). The resulting crude material was purified by flash column chromatography on silica gel (heptane/ethyl acetate 95:5 to 50:50, Rf = 0.52 in heptane/ethyl acetate 90:10) to afford the desired compound as a as a colourless solid (2.2 g, 8.1 mmol, 81%). The analytical data was in accordance with the literature.[15]

^{1}H NMR (400 MHz, $CDCl_3$) *δ* 8.07 (d, *J* = 7.4 Hz, 2H), 7.51 (t, *J* = 7.4 Hz, 1H), 7.41 (t, *J* = 7.6 Hz, 2H), 2.41 – 2.26 (m, 2H), 2.26 – 2.11 (m, 2H), 1.85 – 1.67 (m, 2H), 1.65 – 1.48 (m, 3H), 1.48 – 1.33 (m, 1H).

^{13}C NMR (101 MHz, $CDCl_3$) *δ* 197.6, 136.0, 132.1, 129.9 (2C), 128.2 (2C), 68.1, 38.4 (2C), 25.1 (2C), 23.7.

3.9.2. Bromide abstraction - regioconvergence

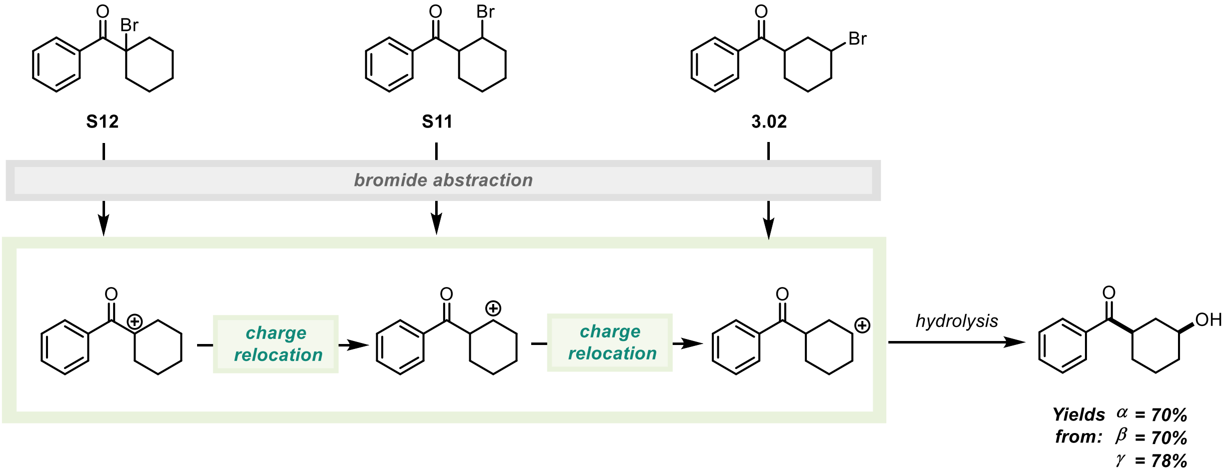

In a flame-dried vial, the corresponding bromide (27.0 mg, 0.10 mmol, 1.00 equiv.) was dissolved in dichloromethane (1 mL). Then, silver hexafluoroantimonate (36.0 mg, 0.10 mmol, 1.00 equiv.) was added and the reaction was stirred for 10 min at 23 °C. Following this time, a saturated aqueous solution of sodium bicarbonate (1 mL) was added and the biphasic mixture was extracted with dichloromethane (3 x 2 mL). The combined organic phases were dried over anhydrous magnesium sulfate, and concentrated under reduced pressure. The resulting crude material was purified by flash column chromatography on silica gel (heptane/ethyl acetate 80:20 to 50:50, Rf = 0.36 in heptane/ethyl acetate 66:33) to afford the desired compound as a colourless solid (average yield 72%).[16]

3.10. NOESY experiment to demonstrate the relative stereochemistry

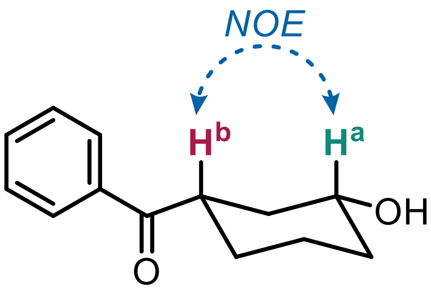

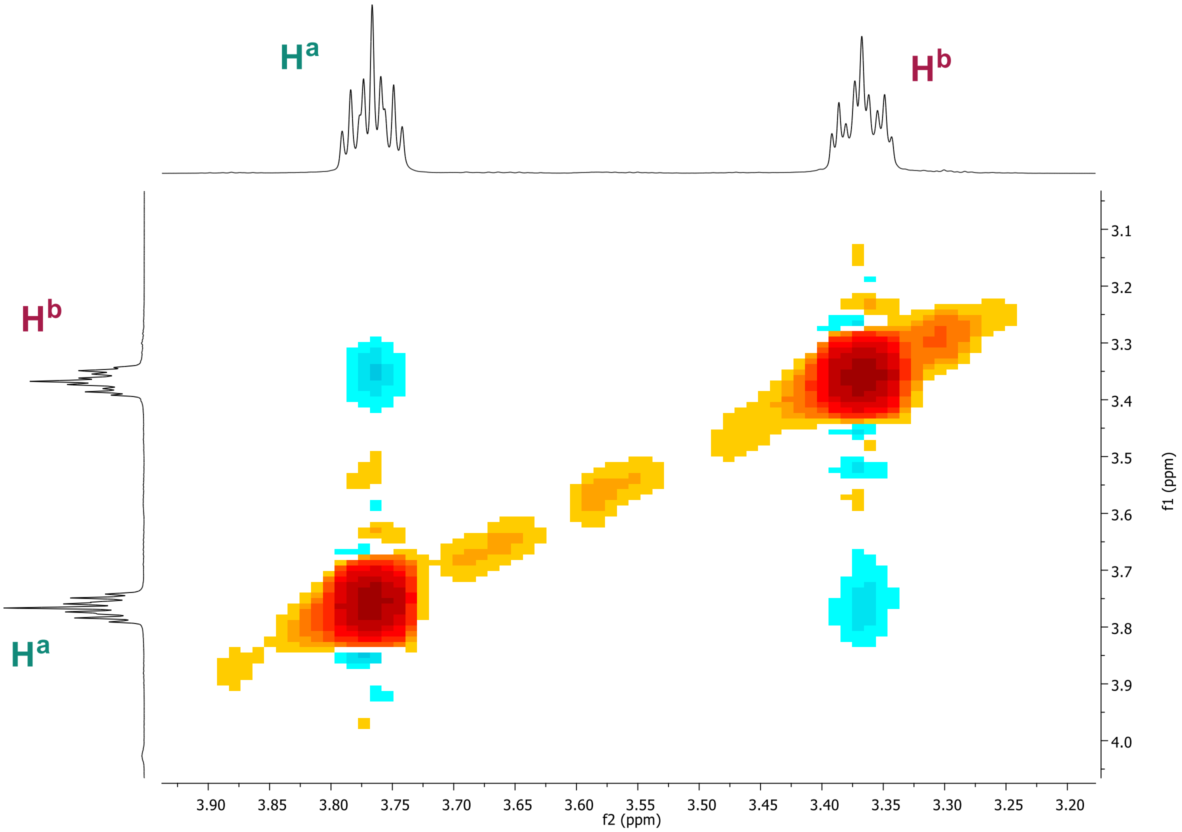

4. NMR Spectra

Information regarding the reporting of NMR spectra of regioisomeric mixtures:

^{1}H NMR and ^{13}C NMR spectra:

Where signals of the minor regioisomers can be seen, for simplicity, only defining and representative peak of that isomer are integrated and labeled, in order to illustrate the ratio of the regioisomers.

1: 4-hydroxy-1-phenylheptan-1-one

^{1}H NMR (400 MHz, $CDCl_3$)

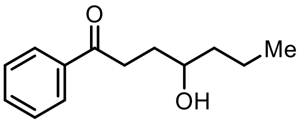

1

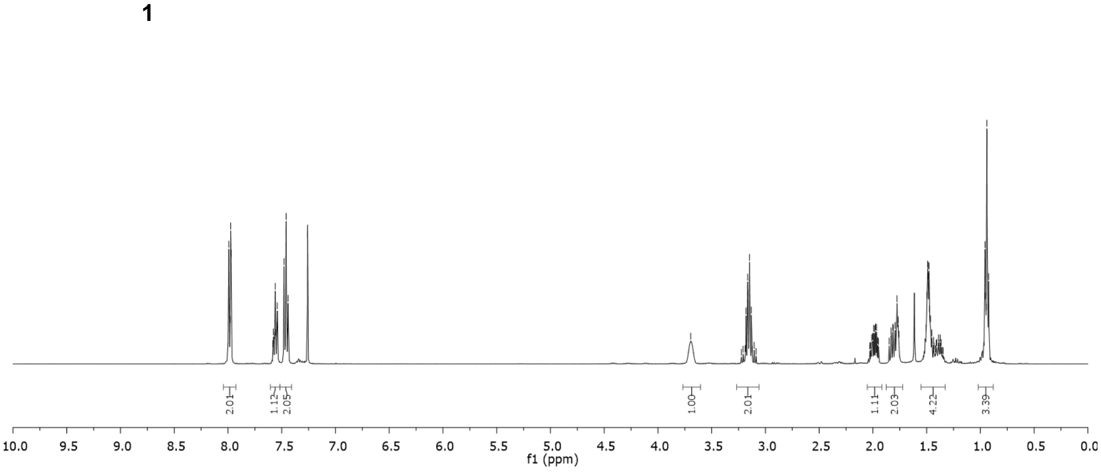

^{13}C NMR (151 MHz, $CDCl_3$)

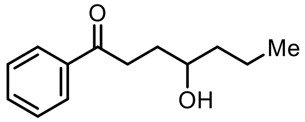

1

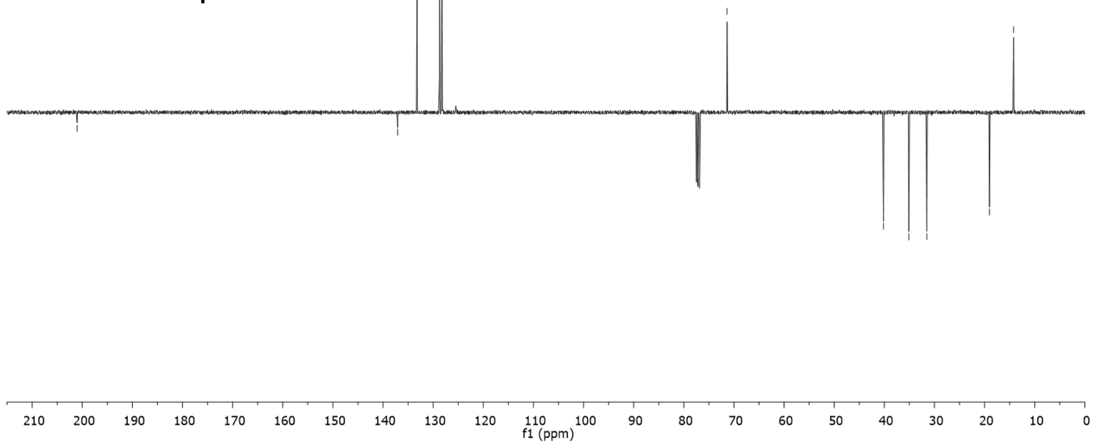

2: 4-hydroxy-1-phenyldecan-1-one

^{1}H NMR (400 MHz, CDCl$_3$)

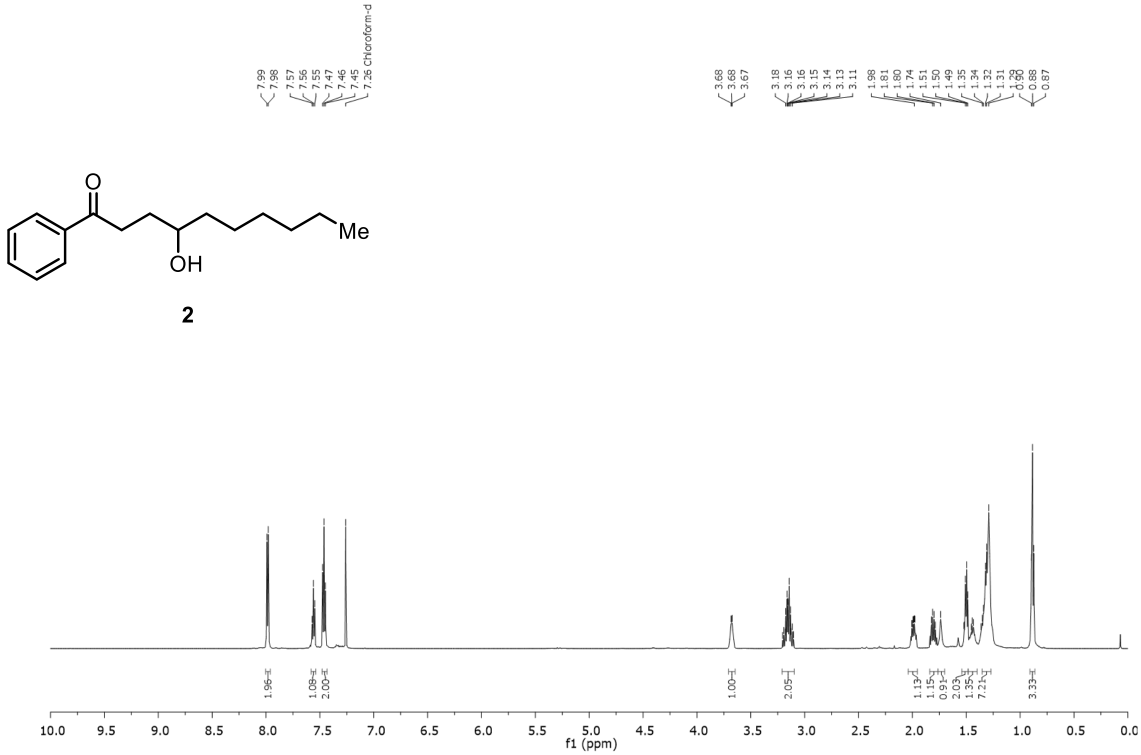

^{13}C NMR (151 MHz, CDCl$_3$)

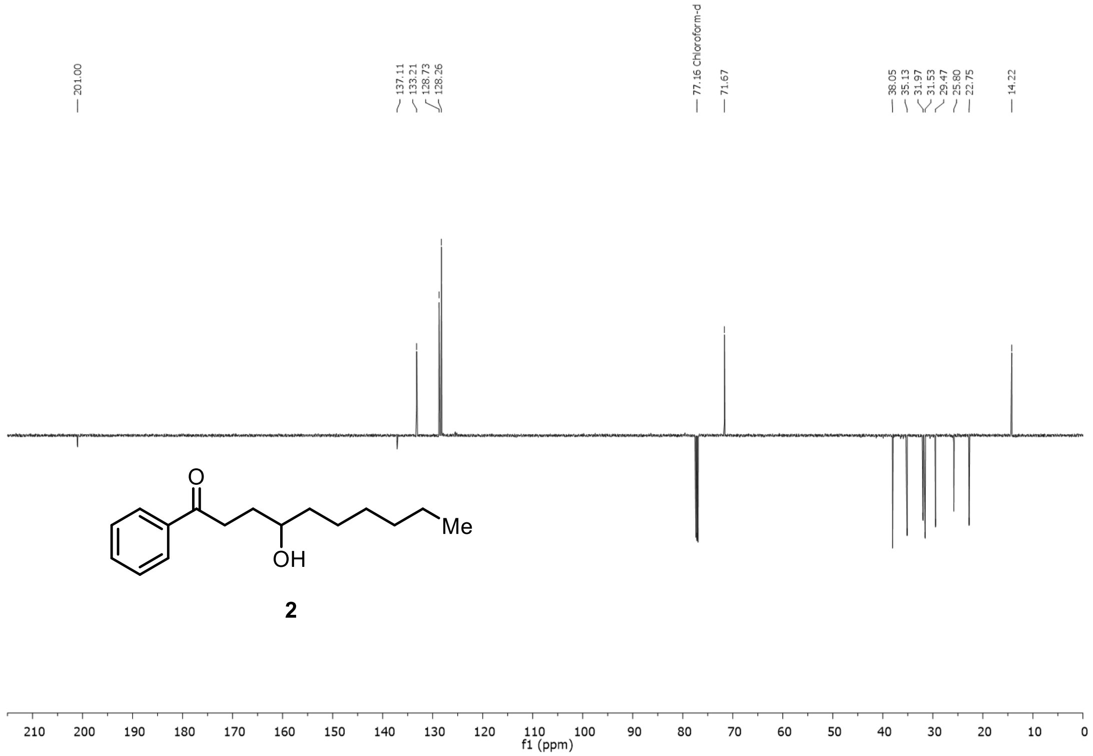

3: 4-hydroxy-7-oxo-7-phenylheptyl 2,2,2-trifluoroacetate

^{1}H NMR (400 MHz, $CDCl_3$)

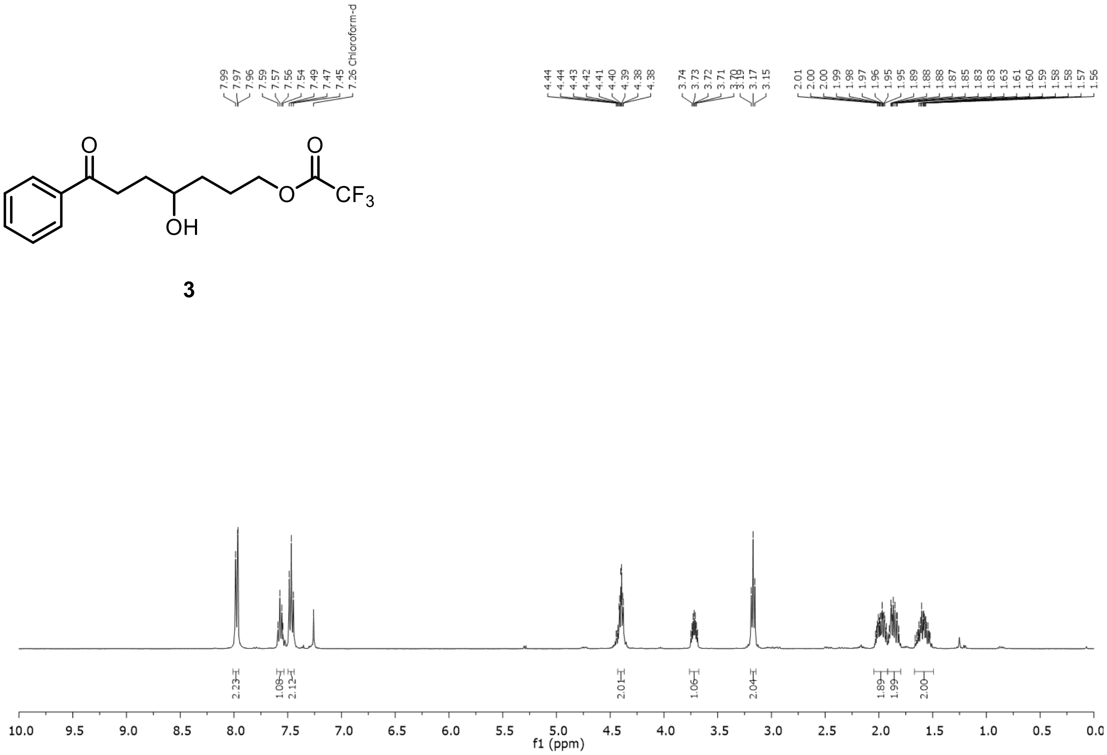

^{19}F NMR (377 MHz, $CDCl_3$)

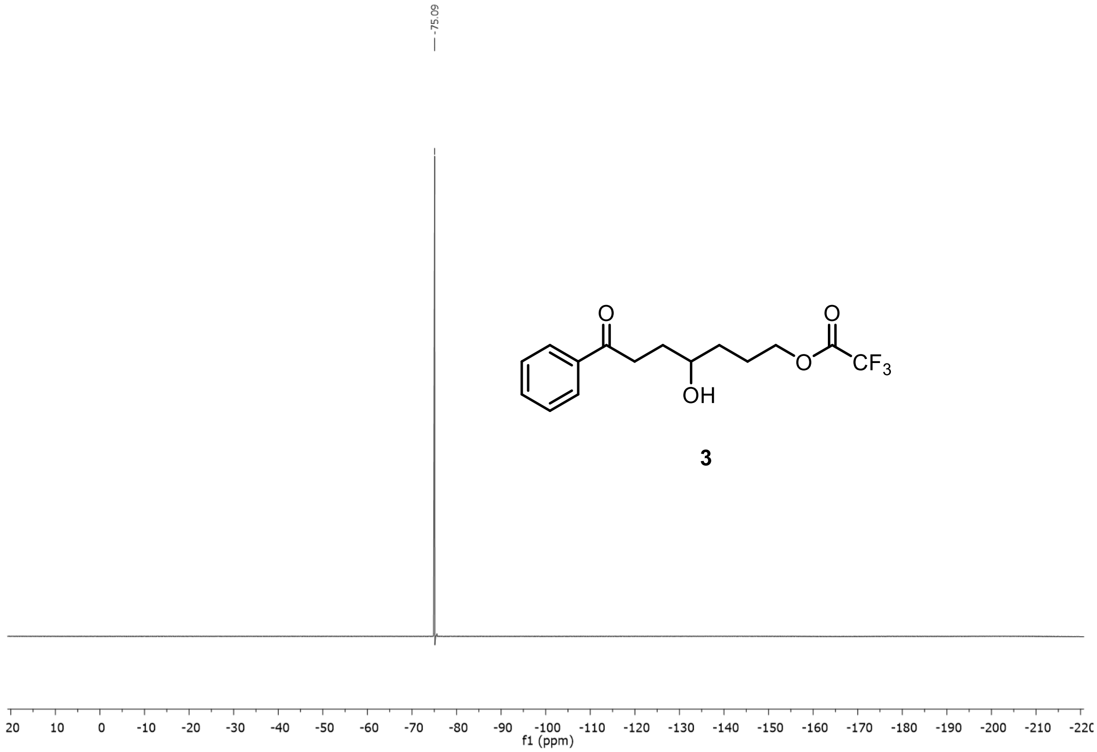

As a long acquisition time in chloroform induces a partial decomposition of the compound, both ^{13}C CPD (using a shorter acquisition time) and ^{13}C DEPTQ-135 NMR are attached.

^{13}C NMR (101 MHz, $CDCl_3$)

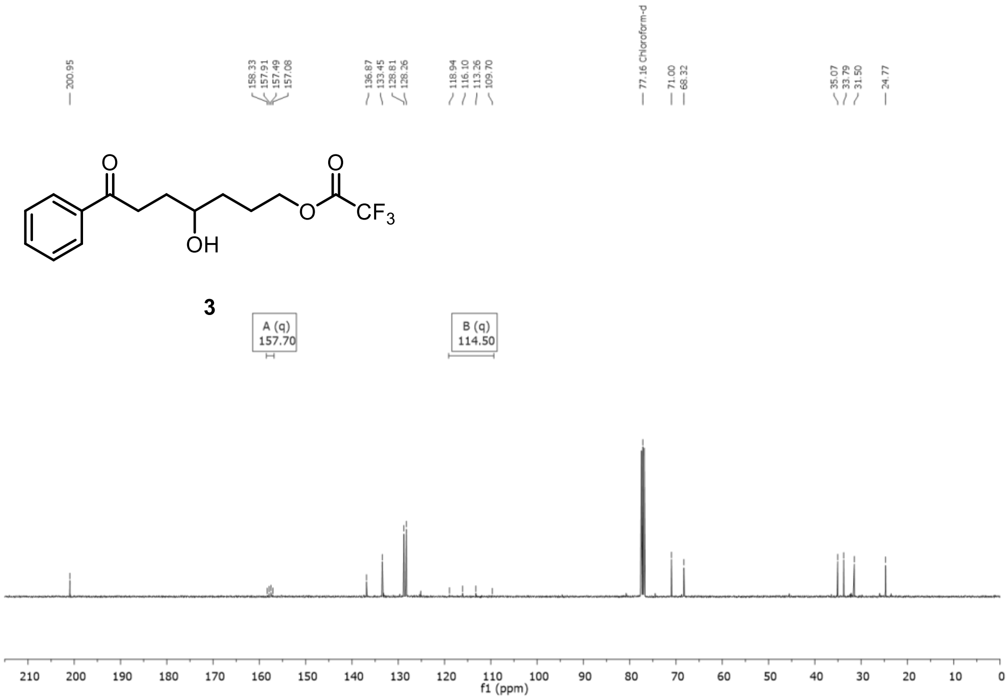

^{13}C NMR (101 MHz, $CDCl_3$)

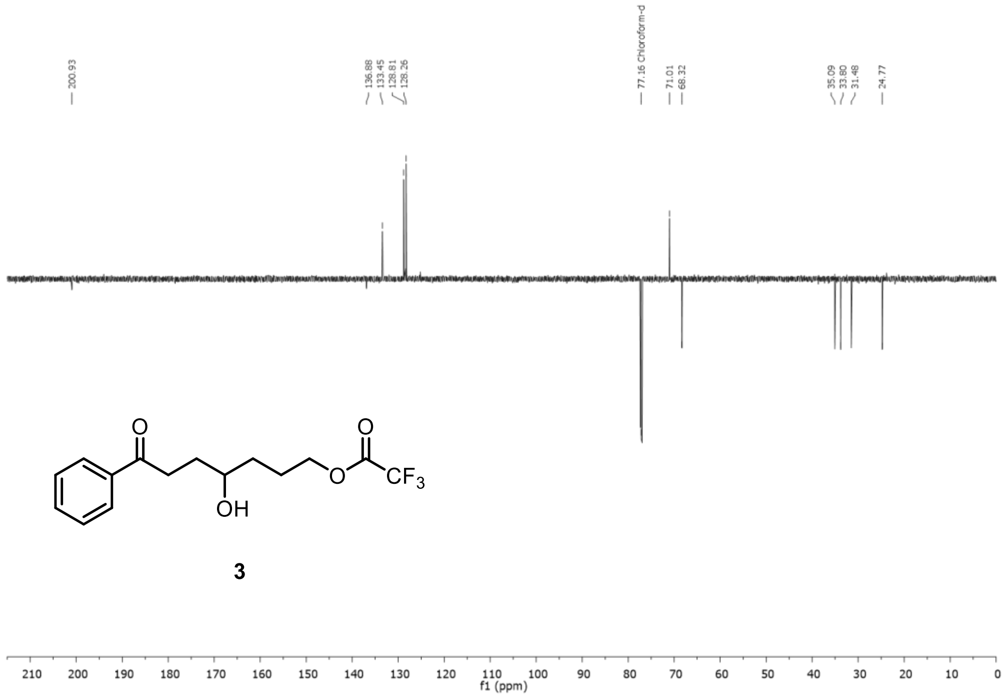

4: 2-(2-hydroxy-5-oxo-5-phenylpentyl)isoindoline-1,3-dione

^{1}H NMR (400 MHz, $CDCl_3$)

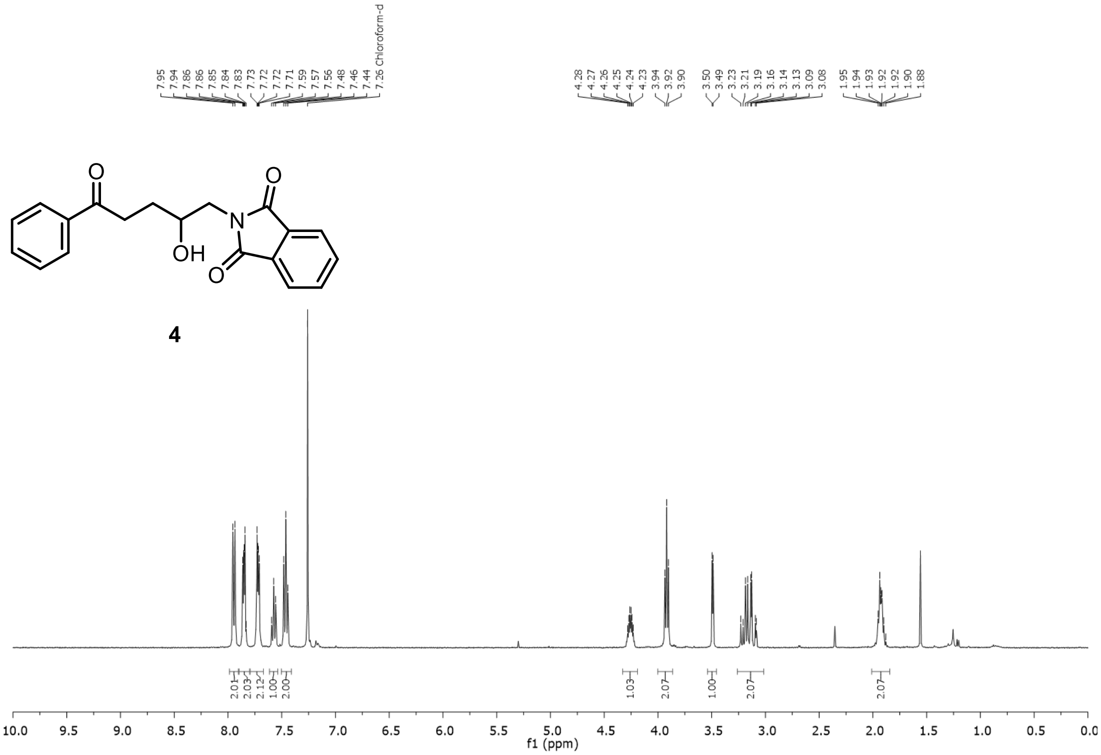

^{13}C NMR (101 MHz, $CDCl_3$)

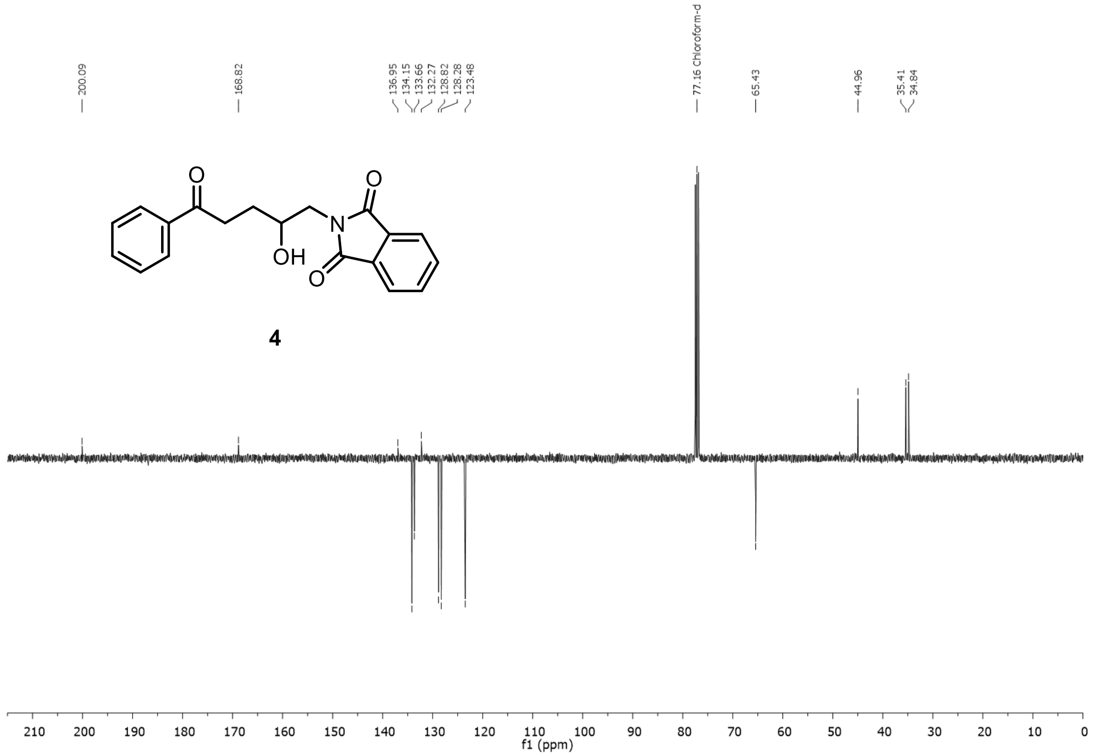

5: 4-hydroxy-1,5-diphenylpentan-1-one

^{1}H NMR (400 MHz, CDCl$_3$)

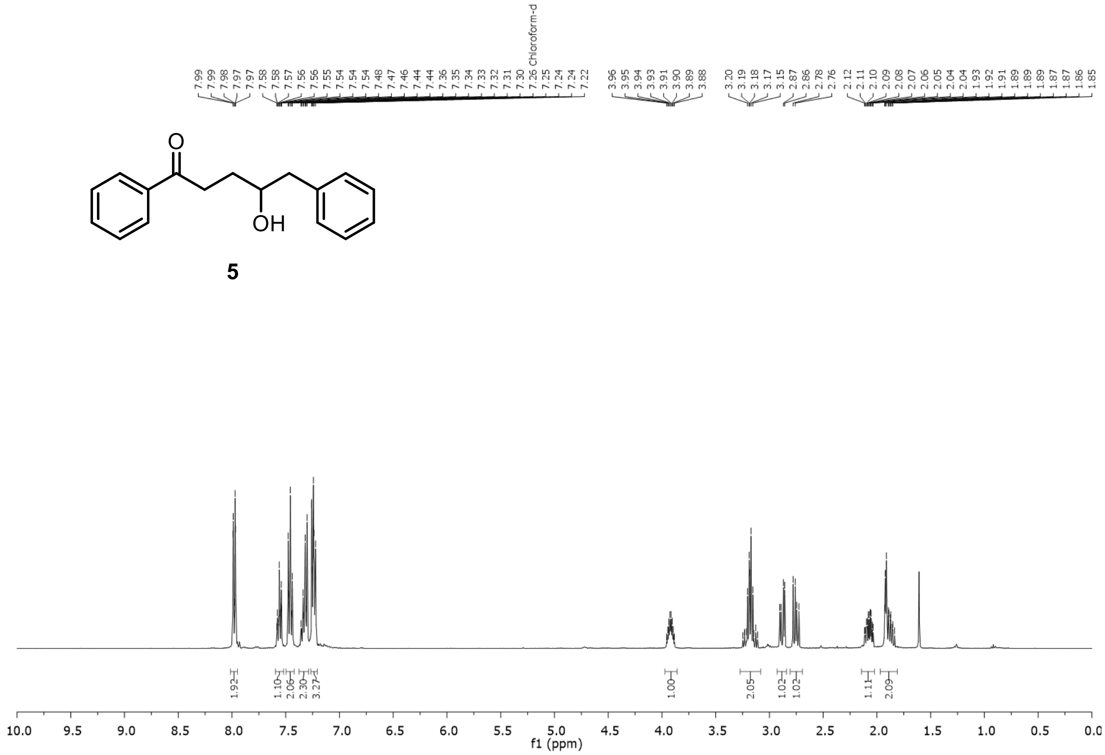

^{13}C NMR (101 MHz, CDCl$_3$)

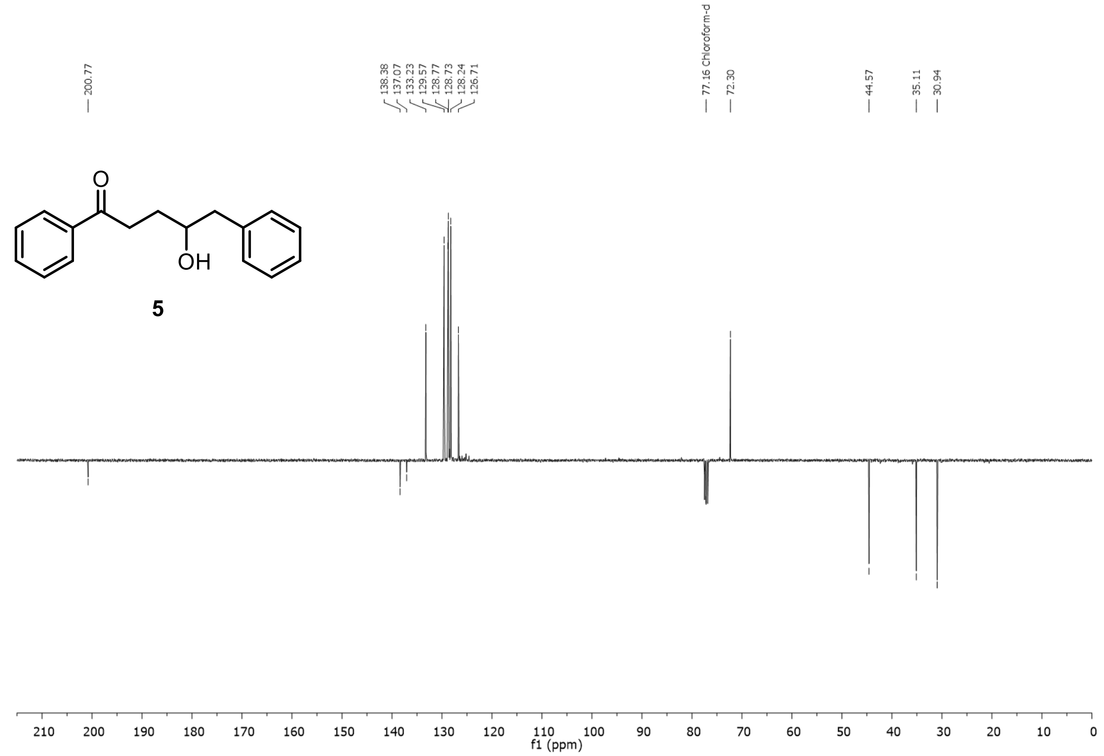

6: *syn*-(3-hydroxycyclohexyl)(phenyl)methanone

^{1}H NMR (600 MHz, $CDCl_3$)

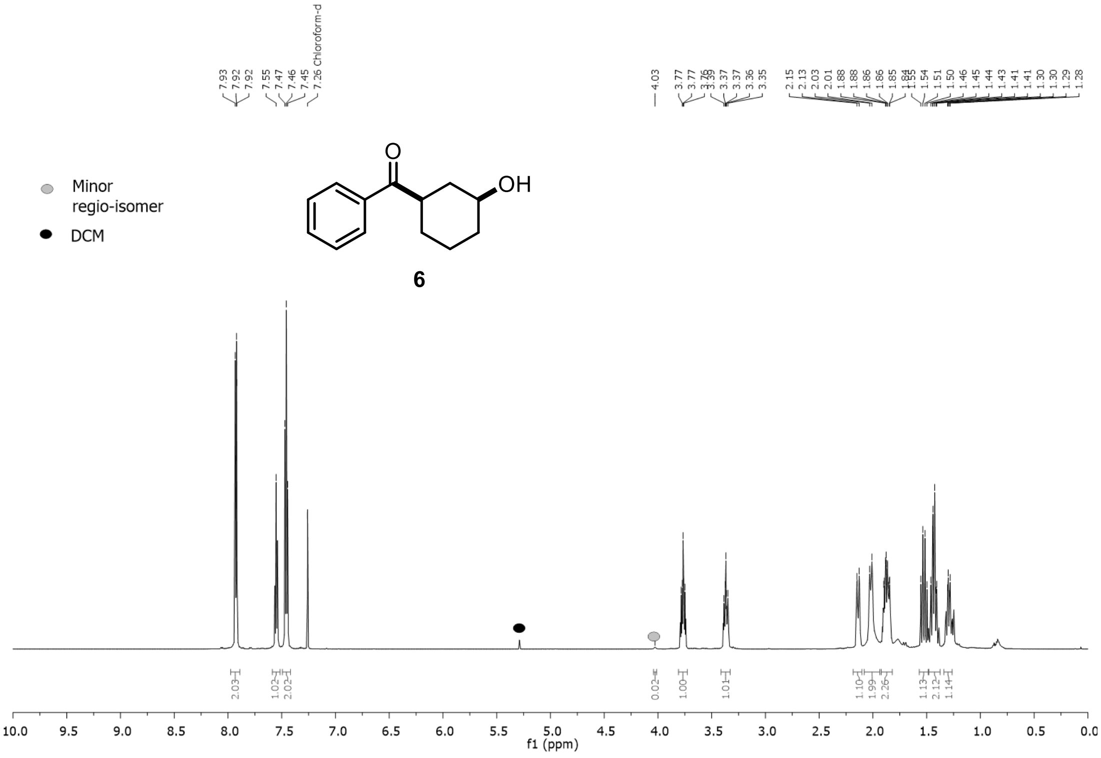

^{13}C NMR (151 MHz, $CDCl_3$)

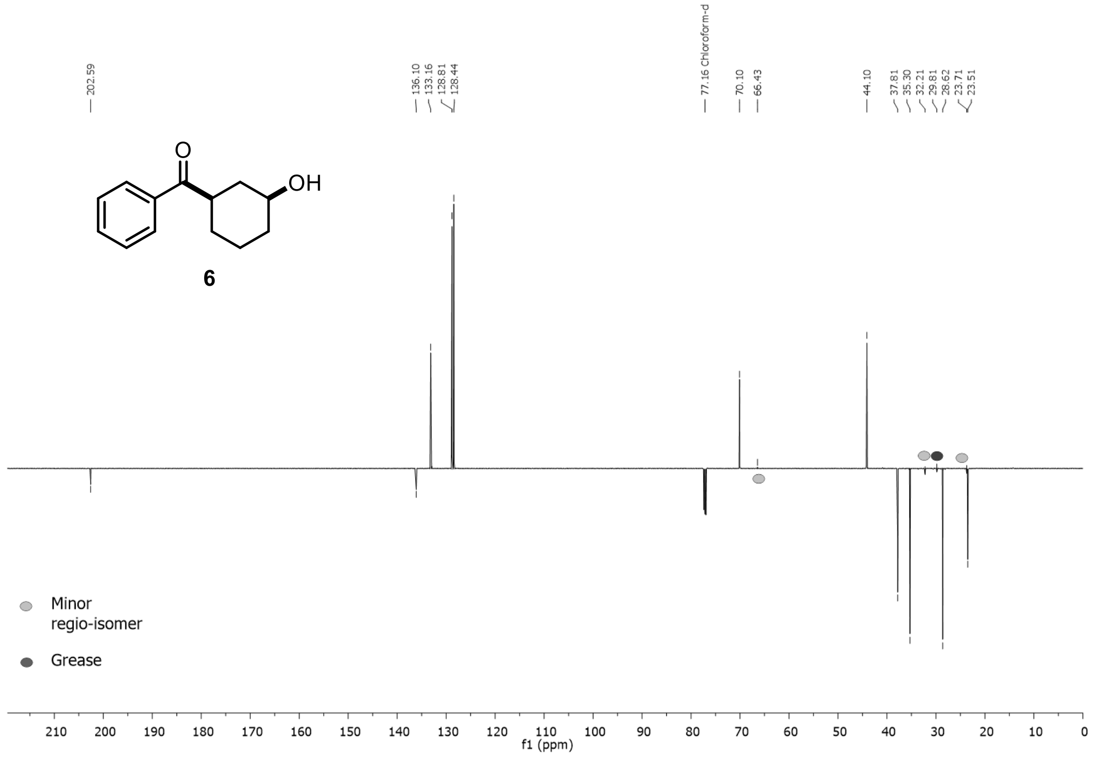

7: *syn*-(4-(tert-butyl)phenyl)(3-hydroxycyclohexyl)methanone

^{1}H NMR (600 MHz, CDCl$_3$)

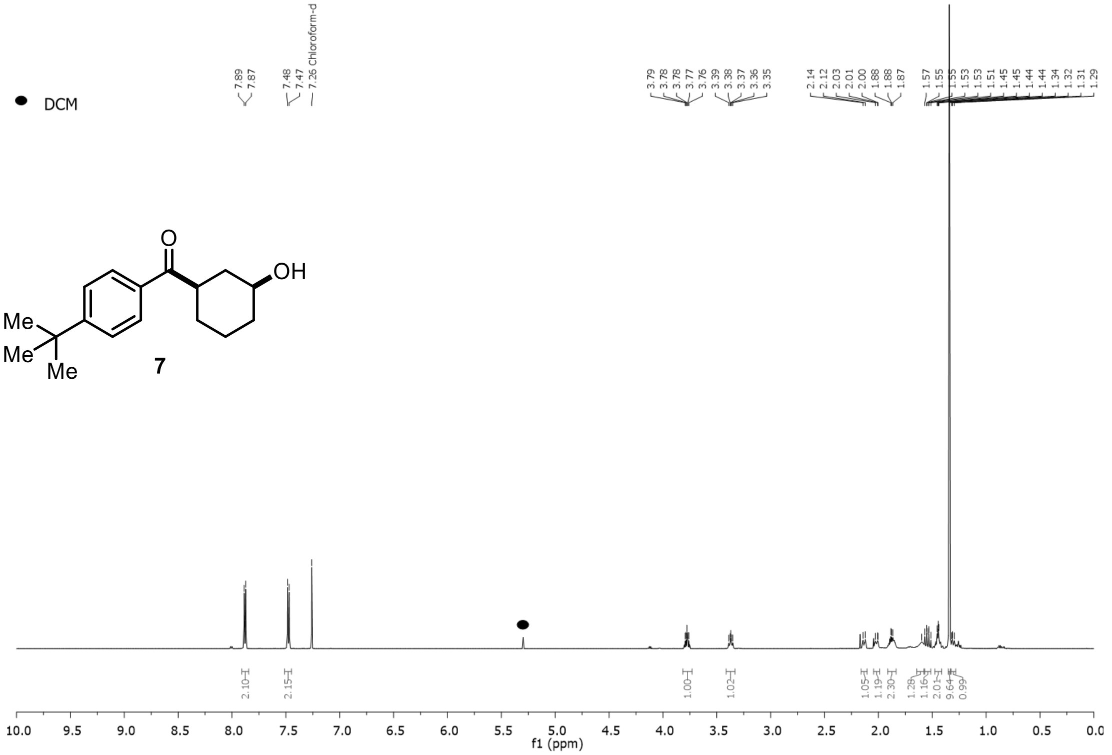

^{13}C NMR (151 MHz, CDCl$_3$)

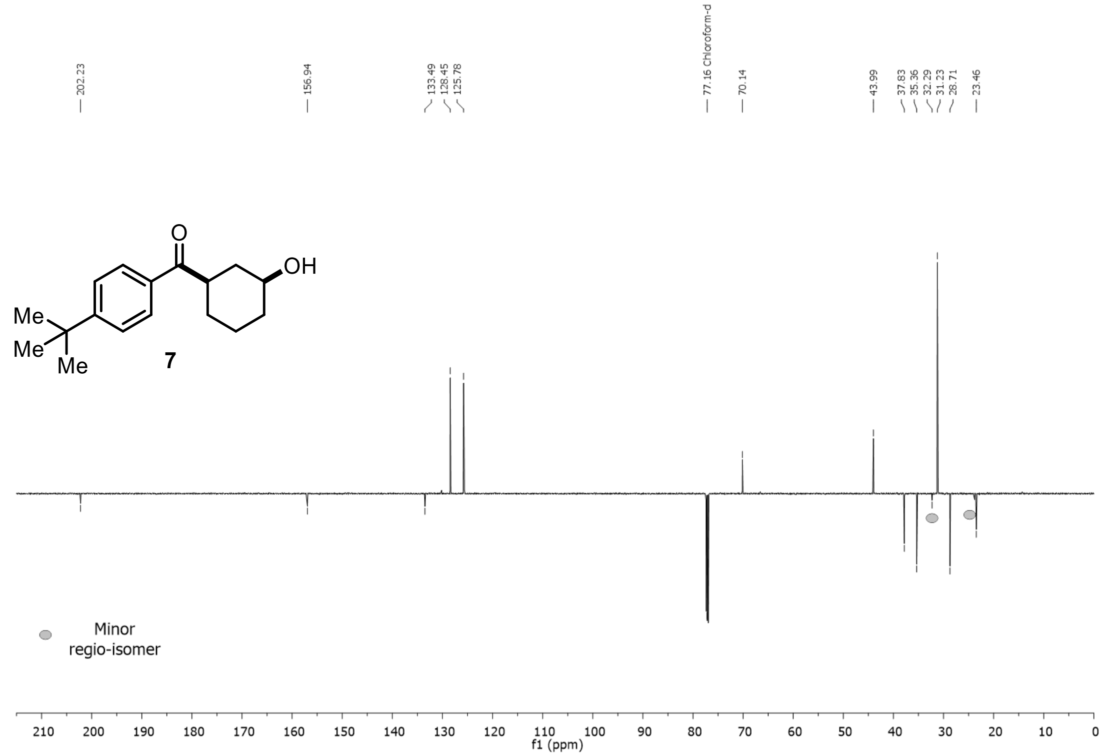

8: *syn*-(3-hydroxycyclohexyl)(3-methoxyphenyl)methanone

^{1}H NMR (400 MHz, $CDCl_3$)

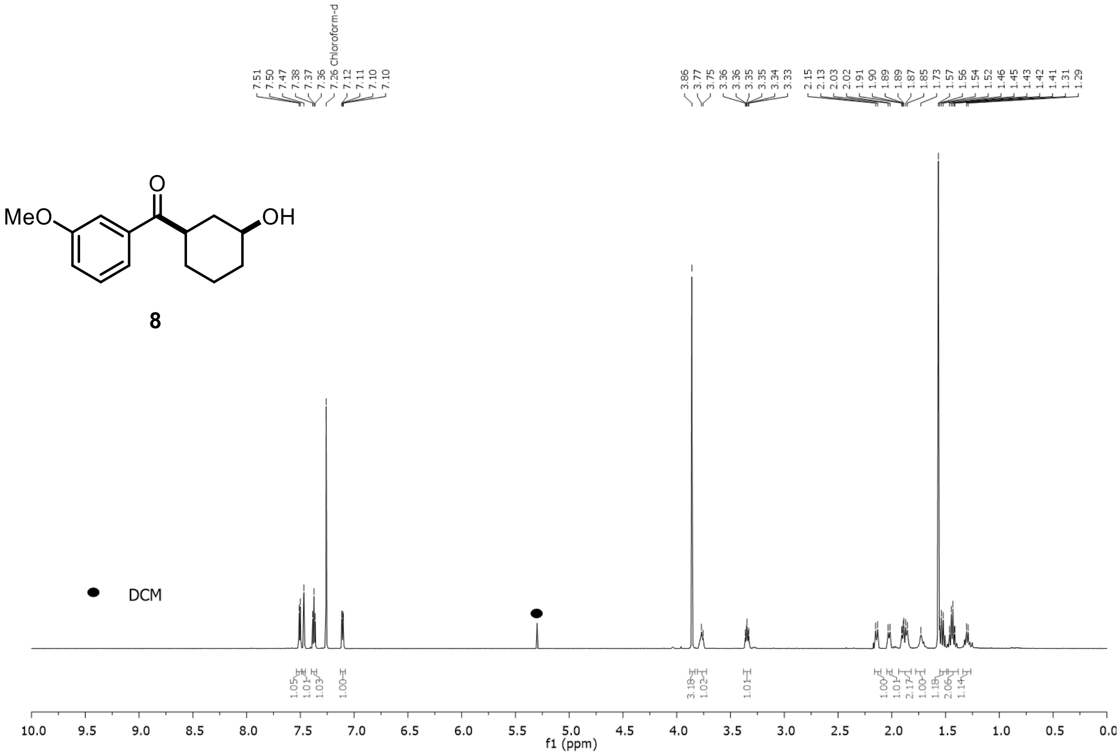

^{13}C NMR (101 MHz, $CDCl_3$)

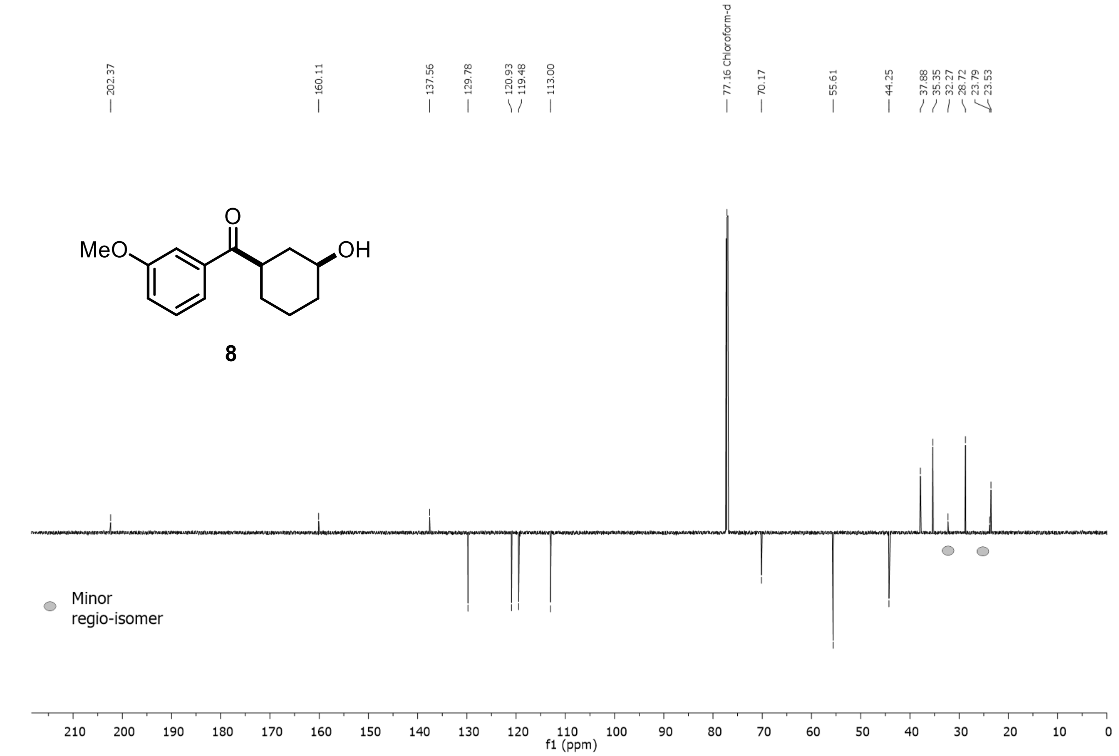

9: *syn*-3-hydroxycyclohexyl(2-iodophenyl)methan one

^{1}H NMR (700 MHz, $CDCl_3$)

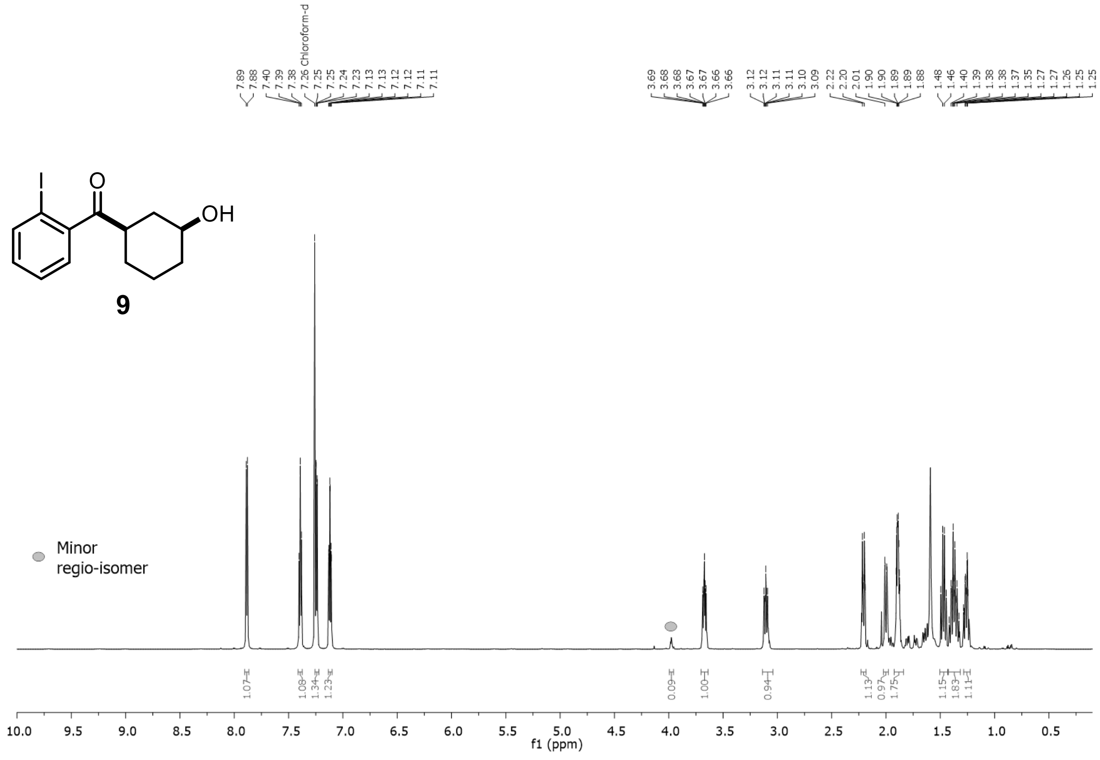

^{13}C NMR (176 MHz, $CDCl_3$)

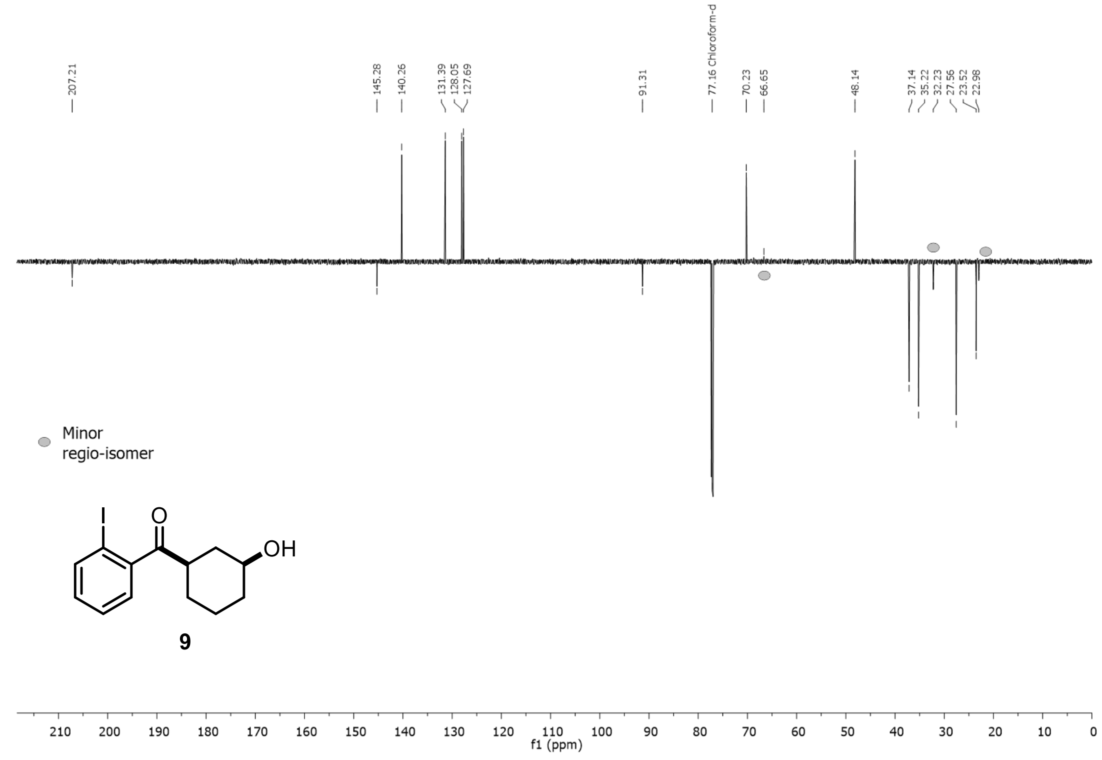

10: *syn*-(3-hydroxycyclohexyl)(4-(trifluoromethyl)phenyl)methanone

^{1}H NMR (600 MHz, $CDCl_3$)

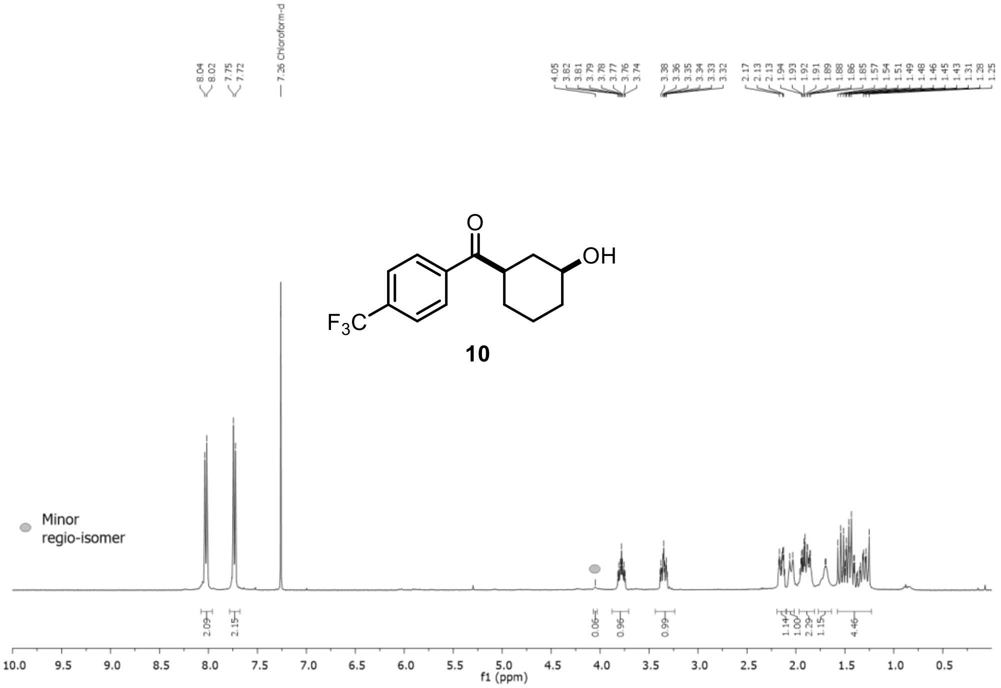

^{13}C NMR (151 MHz, $CDCl_3$)

As the ^{13}C DEPTQ-135 NMR spectrum does not allow visualisation of several key peaks, only the ^{13}C CPD NMR is attached.

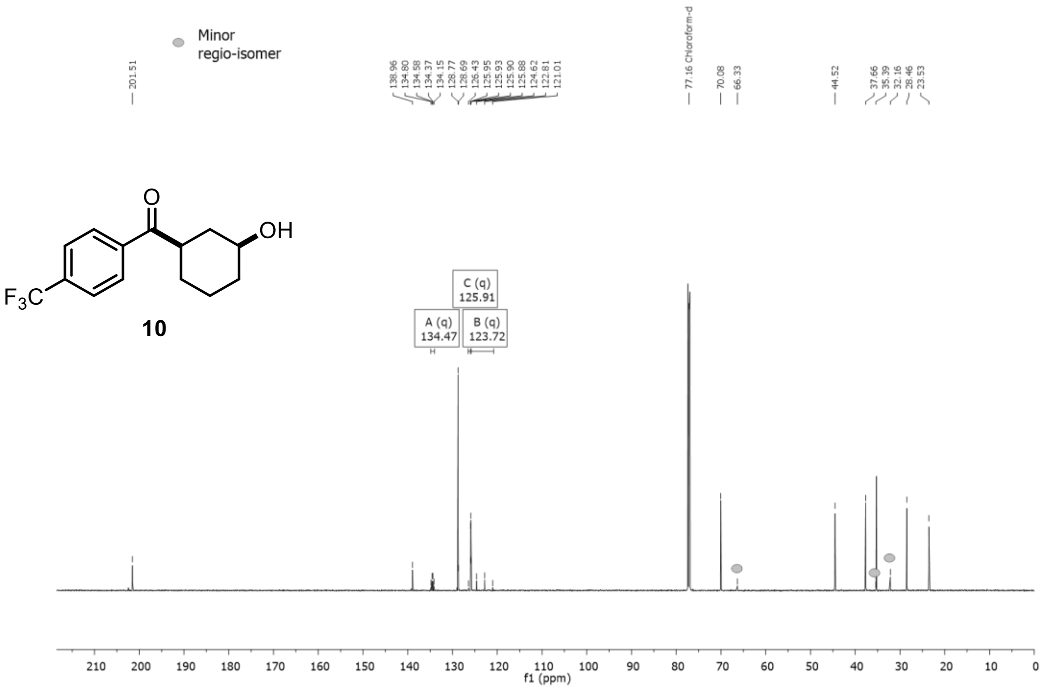

^{19}F NMR (377 MHz, $CDCl_3$)

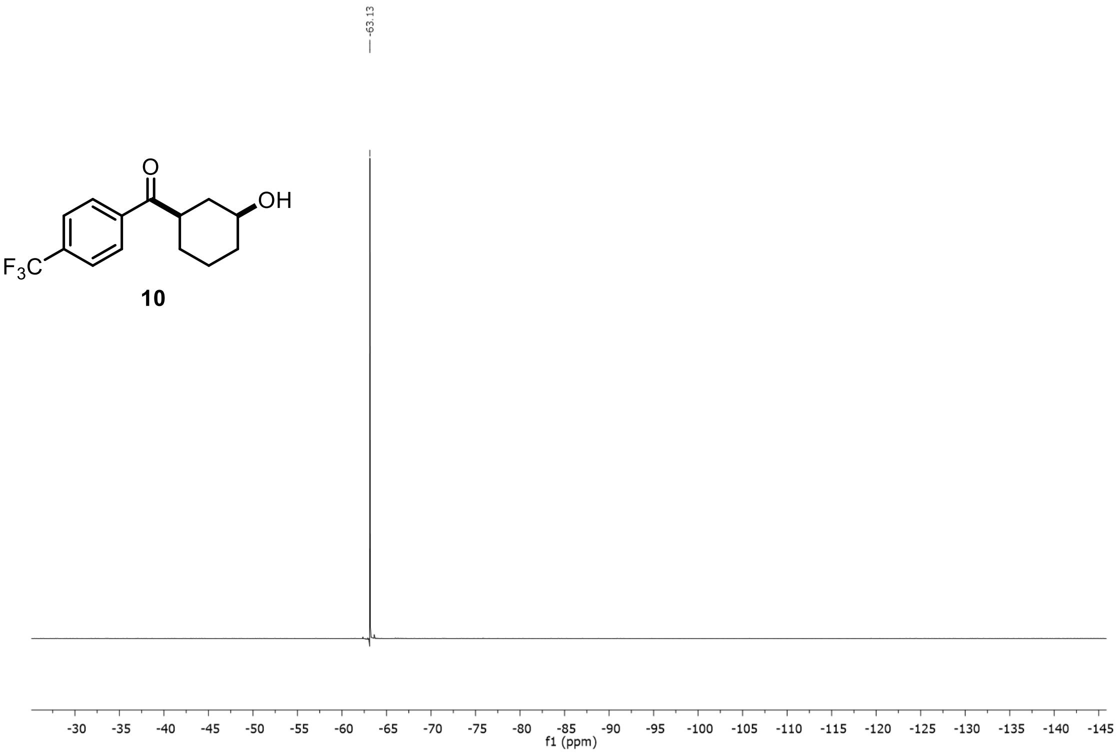

10

11: *syn*-(3,4-dichlorophenyl)(-3-hydroxycyclohexyl)methanone

^{1}H NMR (400 MHz, $CDCl_3$)

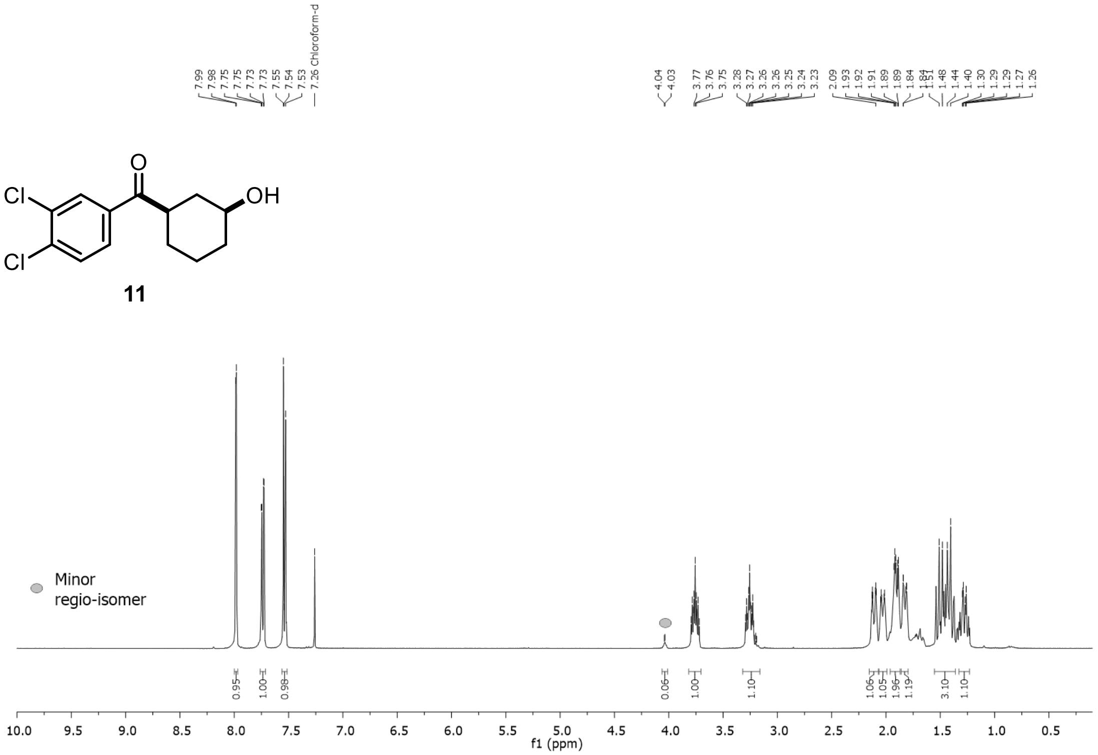

^{13}C NMR (101 MHz, $CDCl_3$)

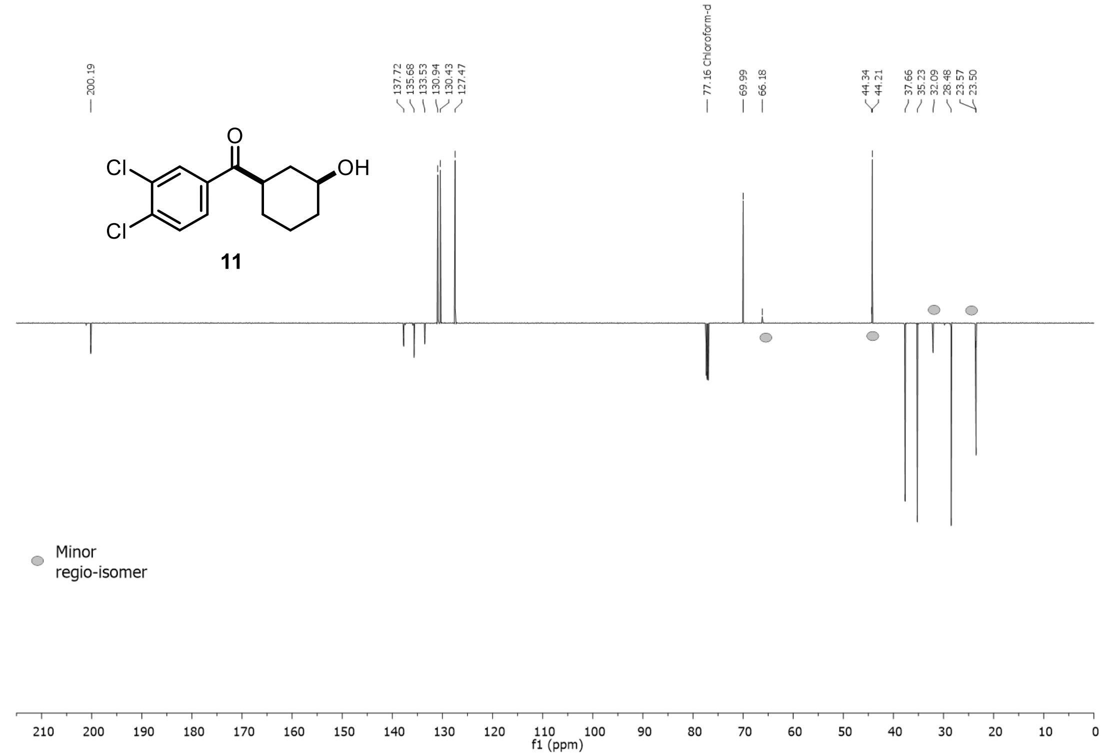

12: *syn*-(3-hydroxycyclohexyl)(mesityl)methanone

^{1}H NMR (500 MHz, $CDCl_3$)

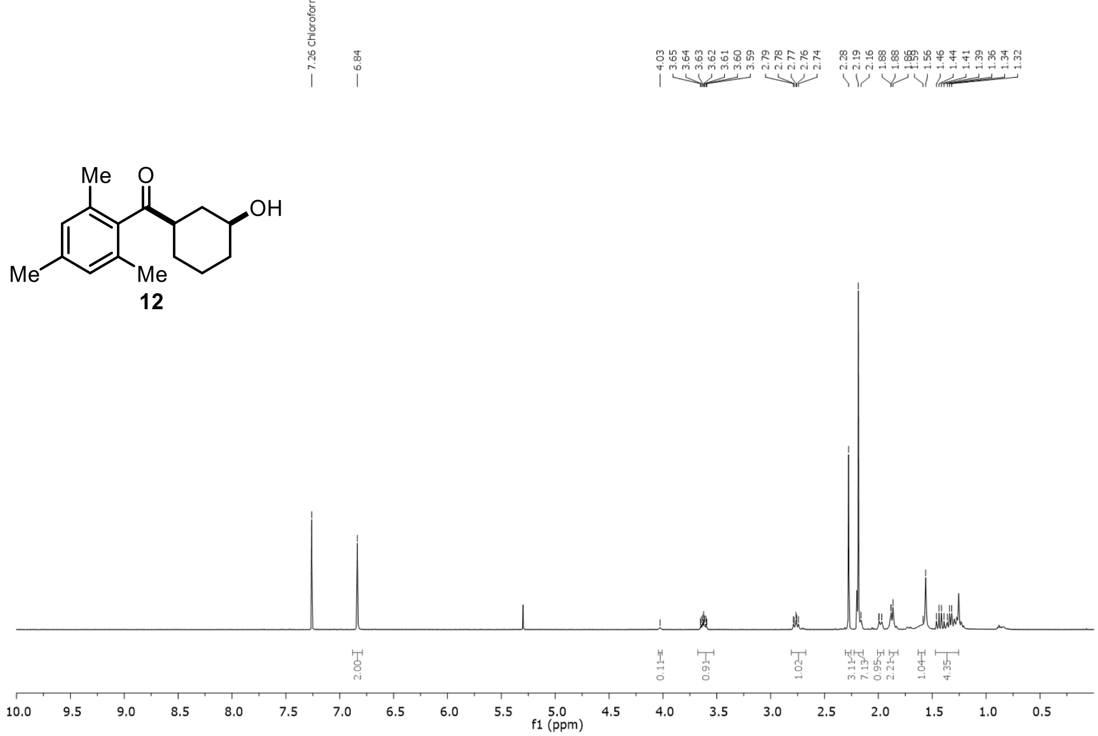

^{13}C NMR (126 MHz, $CDCl_3$)

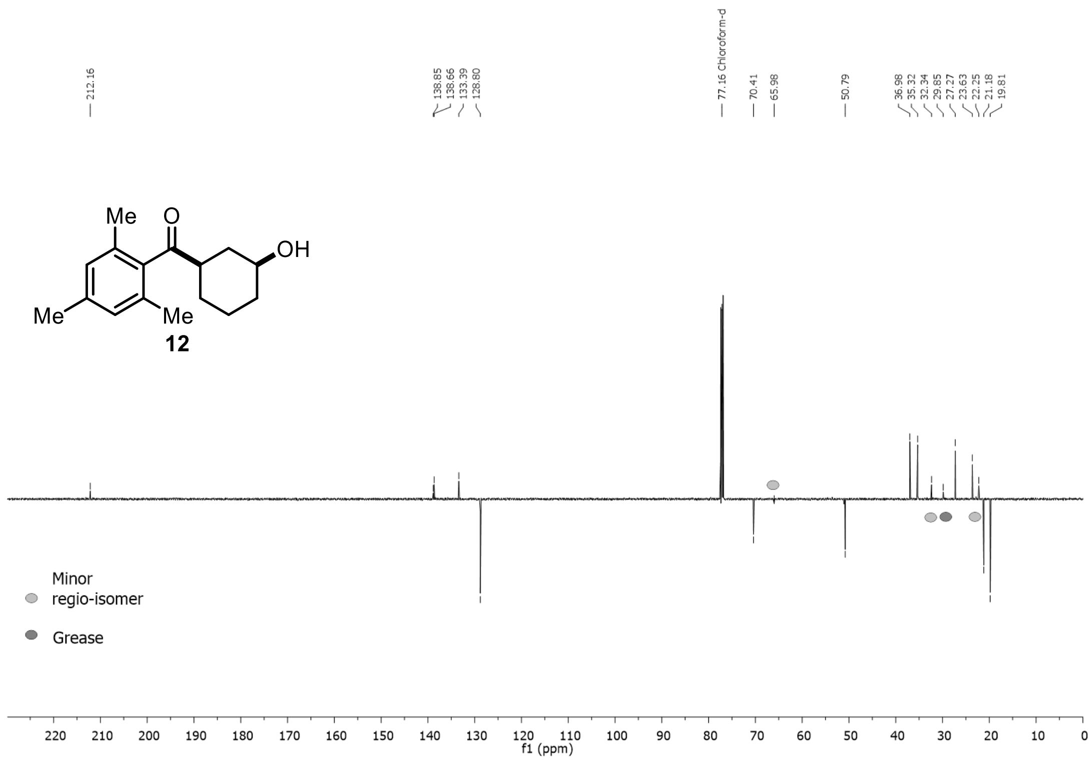

13: *syn*-3-hydroxycyclohexyl(4-nitrophenyl)methanone

^{1}H NMR (700 MHz, $CDCl_3$)

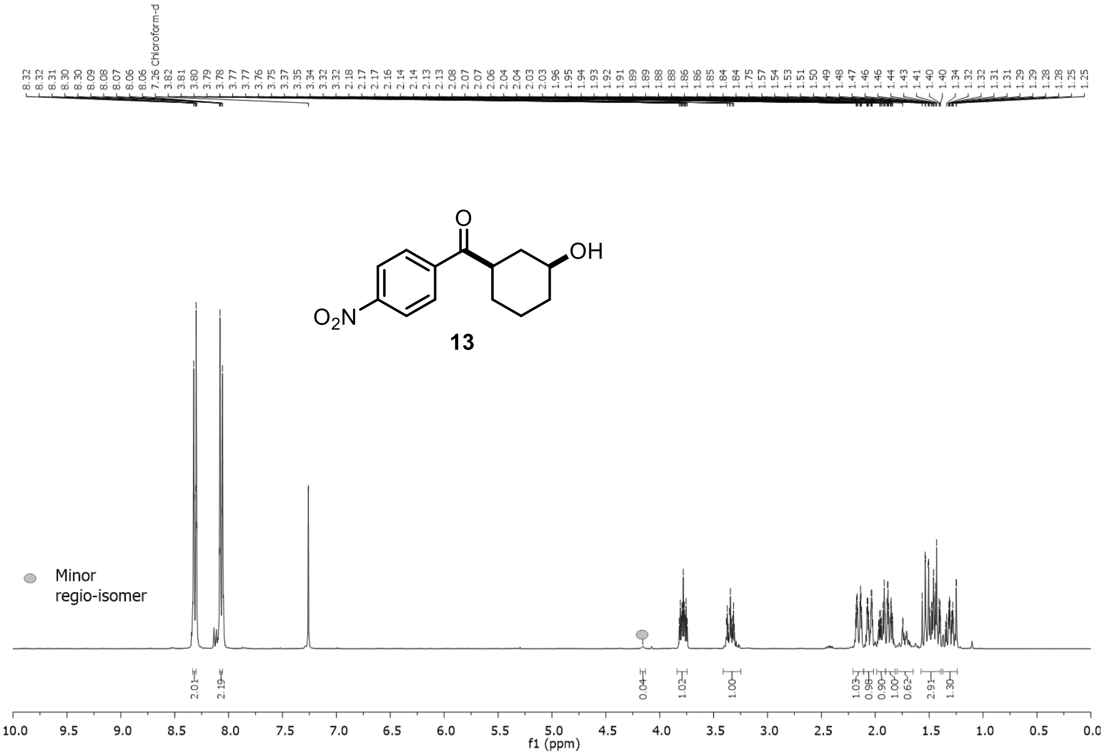

^{13}C NMR (176 MHz, $CDCl_3$)

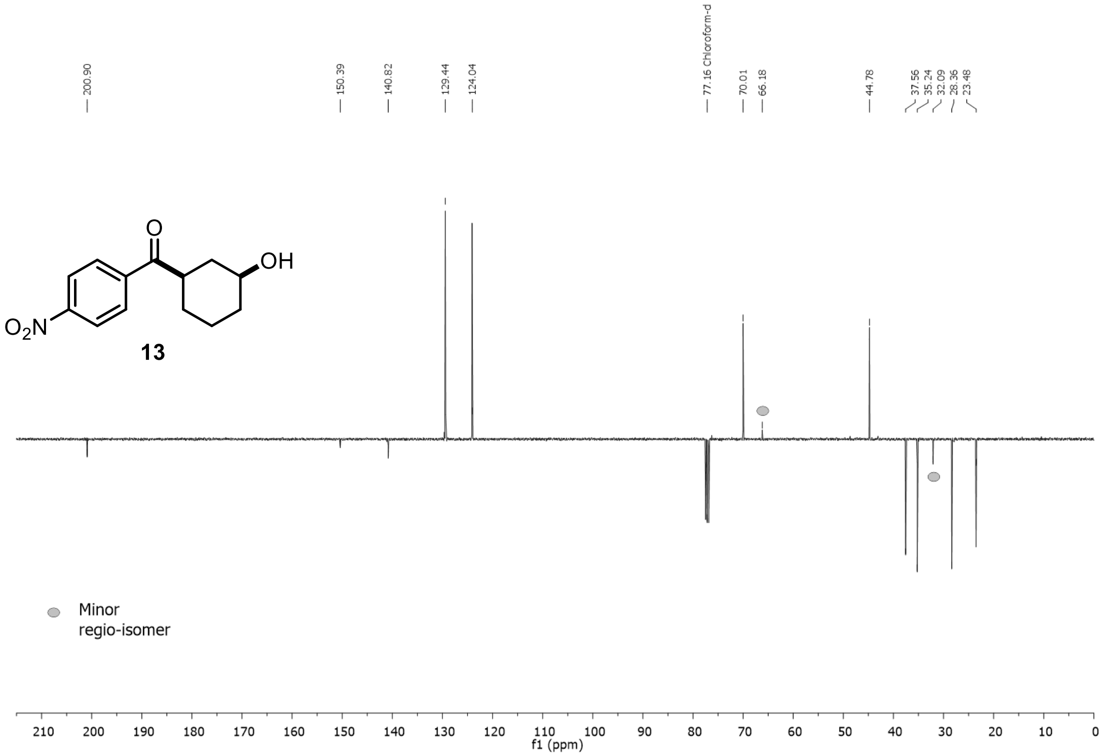

14: ***syn*-methyl 4-(-3-hydroxycyclohexane-1-carbonyl)benzoate**

^{1}H NMR (700 MHz, $CDCl_3$)

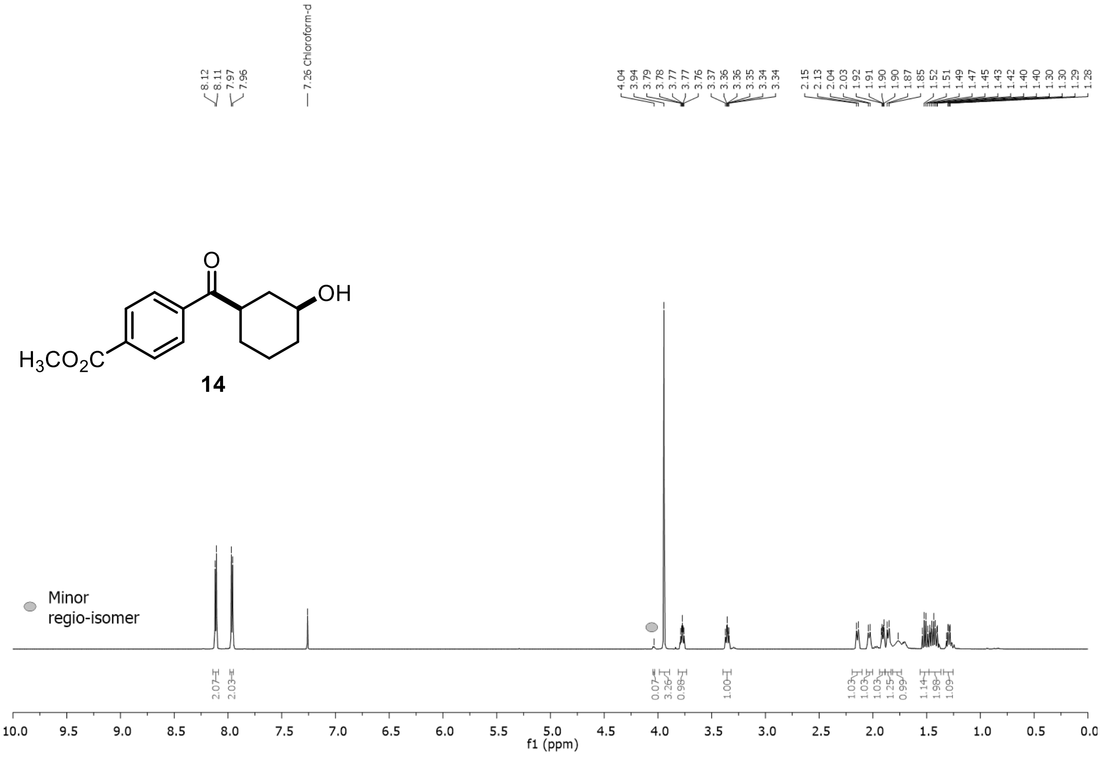

^{13}C NMR (176 MHz, $CDCl_3$)

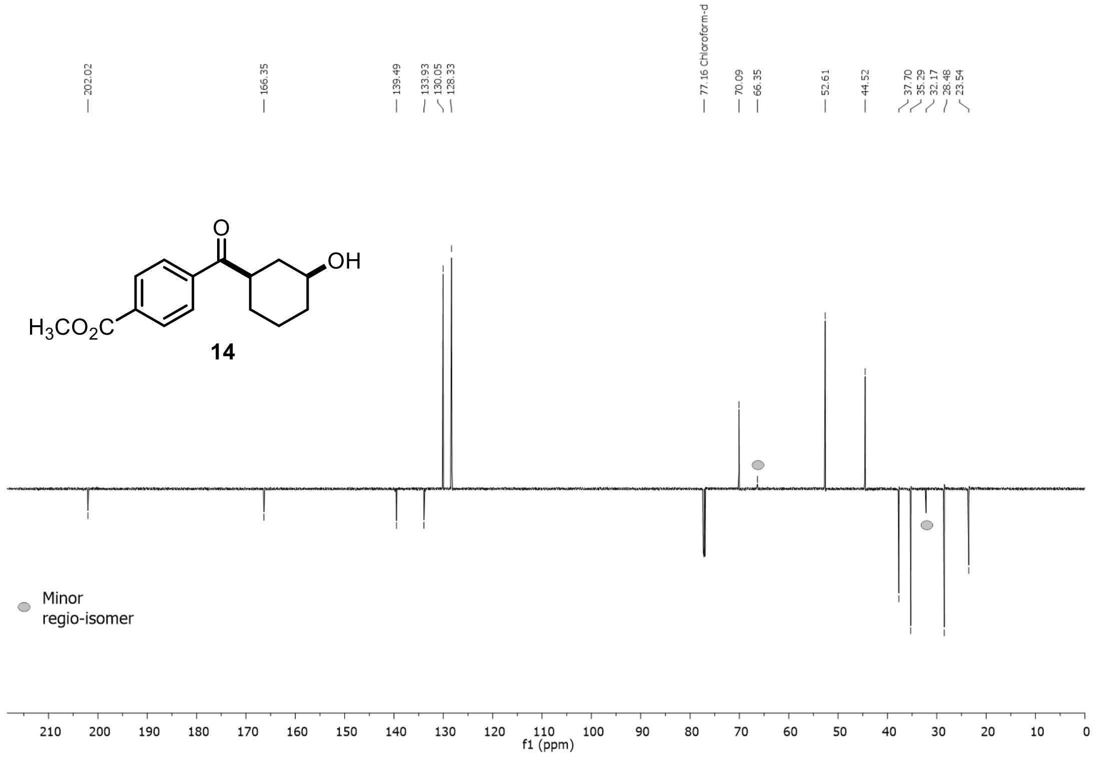

15: *syn*-(3-hydroxycyclohexyl)-2,2-dimethylpropan-1-one

^{1}H NMR (500 MHz, $CDCl_3$)

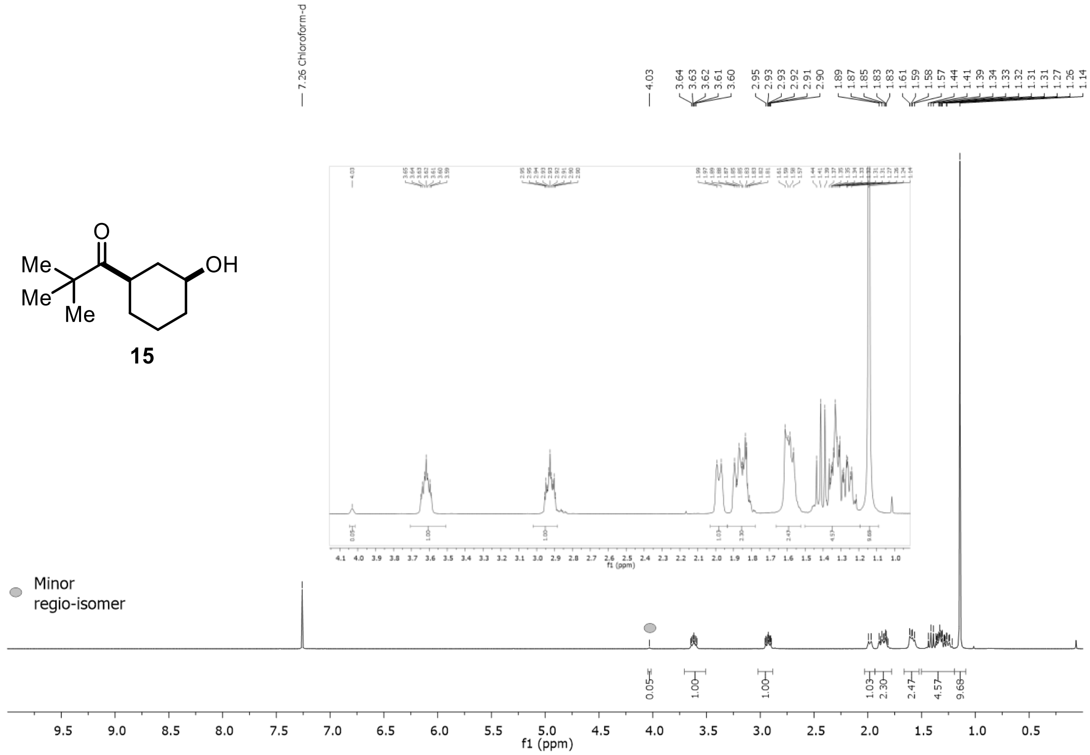

^{13}C NMR (126 MHz, $CDCl_3$)

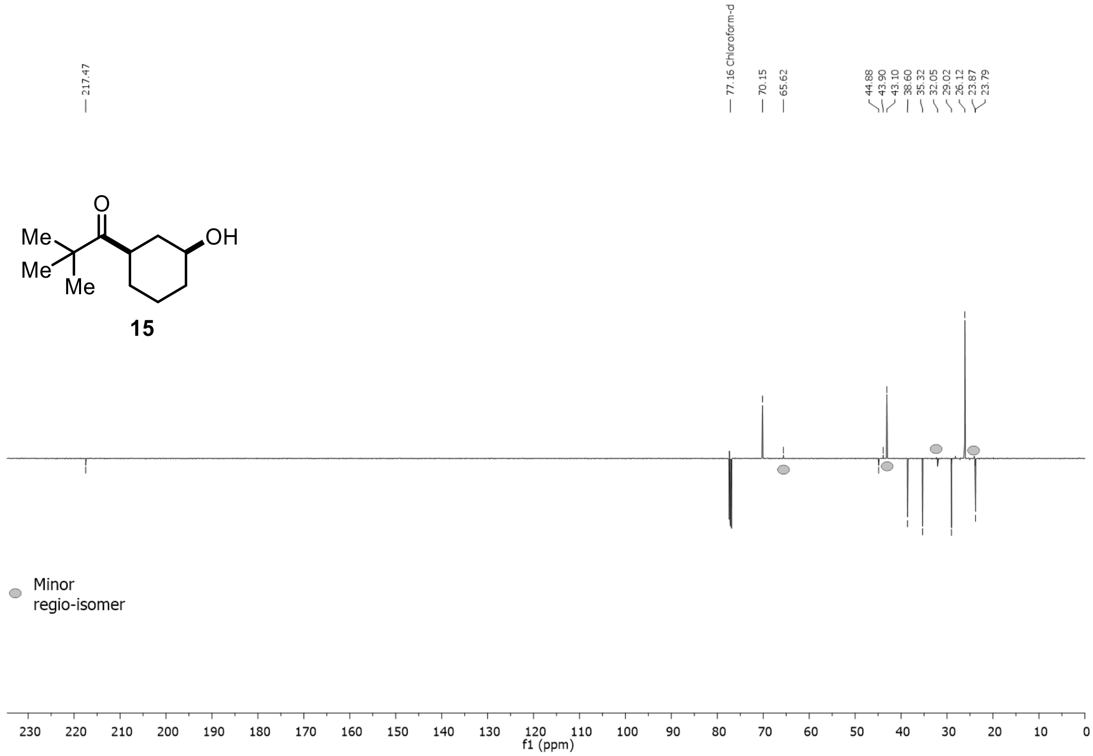

16: *syn*-Benzo[d][1,3]dioxol-5-yl(-3-hydroxycyclohexyl)methanone

^{1}H NMR (600 MHz, $CDCl_3$)

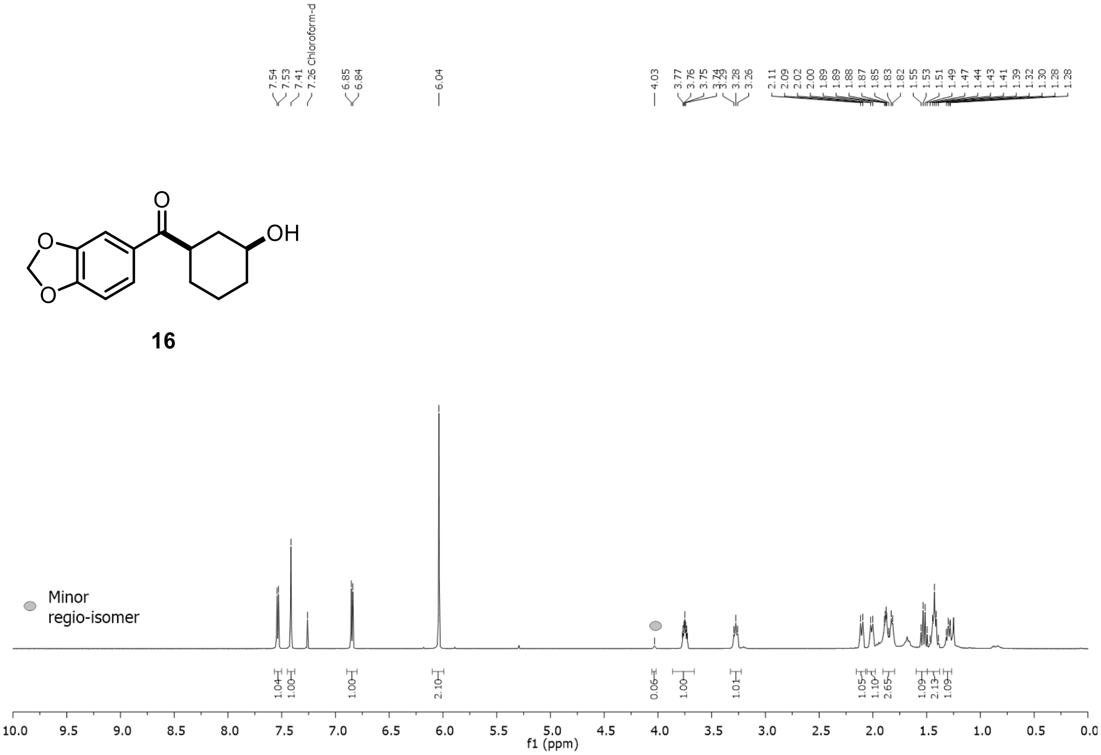

^{13}C NMR (151 MHz, $CDCl_3$)

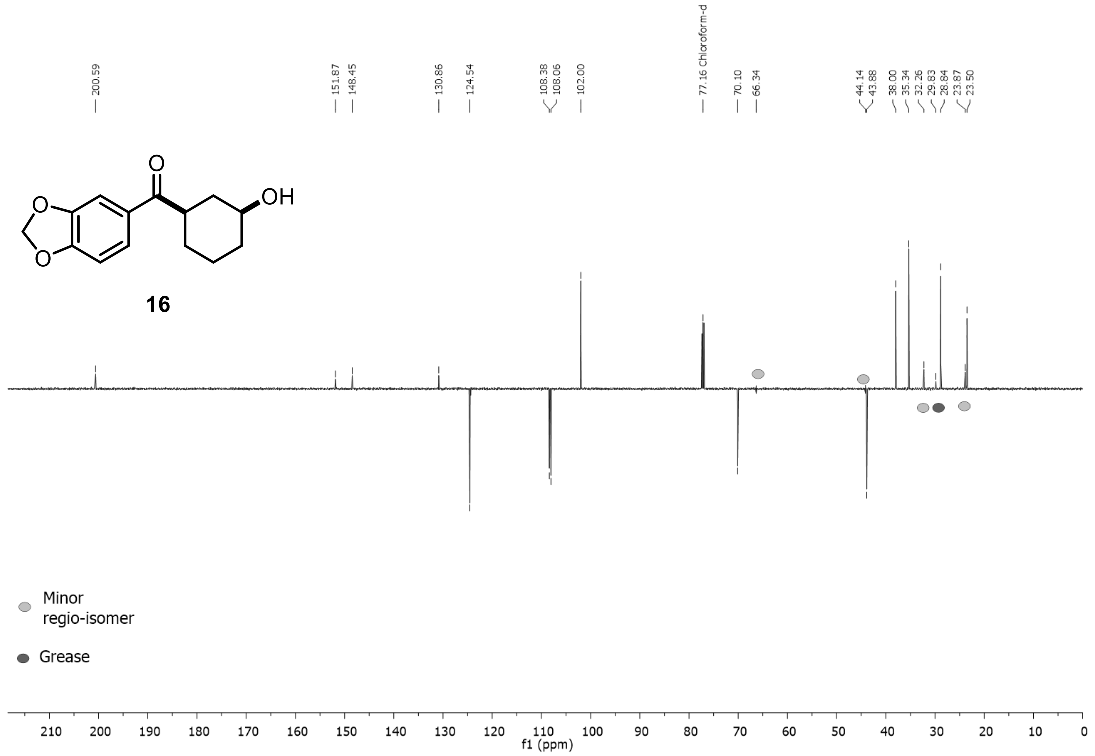

17: *syn*-(3-hydroxycyclohexyl)(naphthalen-2-yl)methanone

^{1}H NMR (700 MHz, $CDCl_3$)

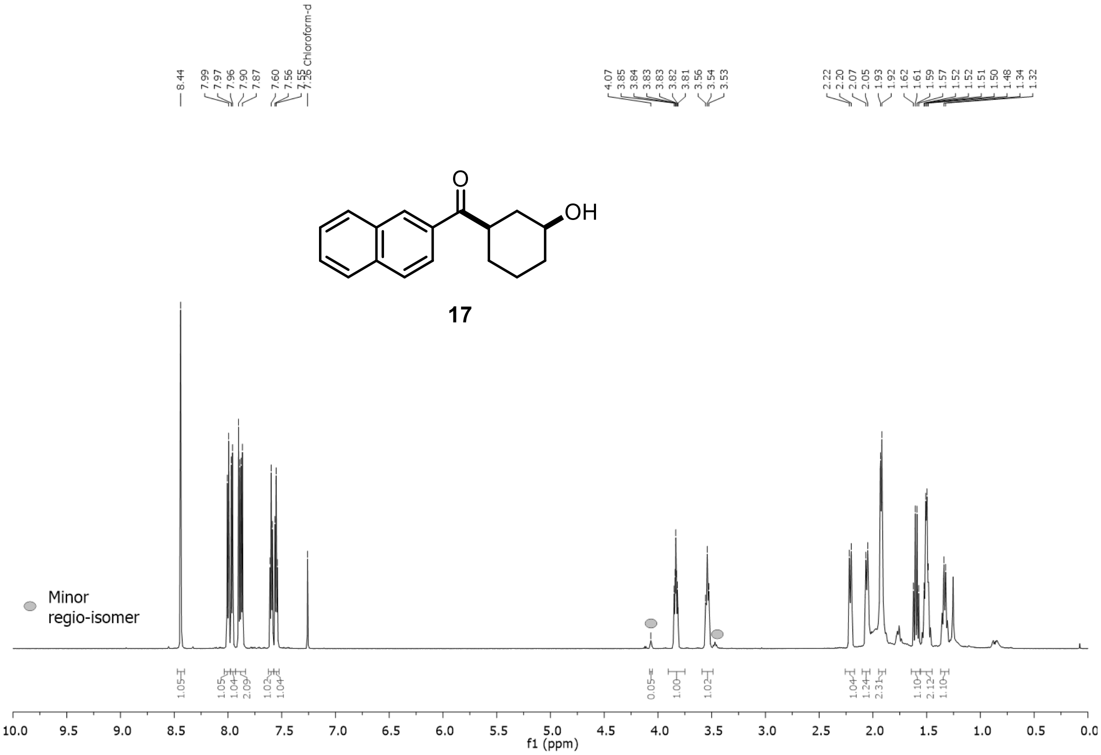

^{13}C NMR (176 MHz, $CDCl_3$)

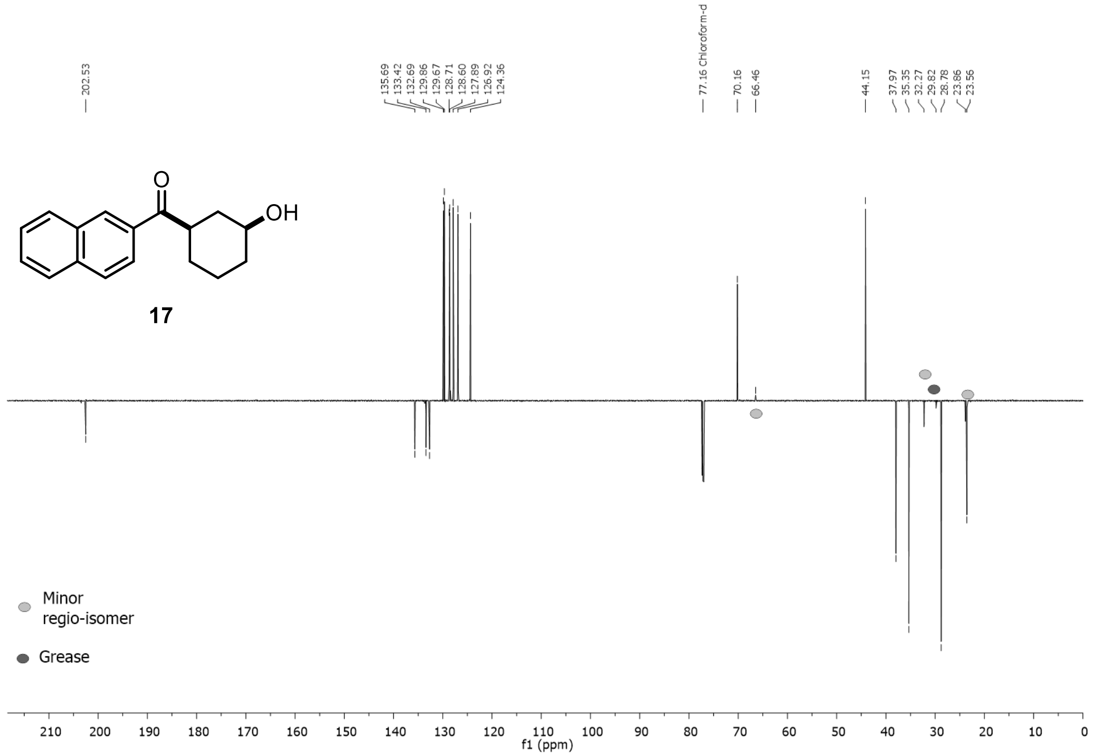

18: *syn*-(3-hydroxycyclopentyl)(3-methoxyphenyl)methanone

^{1}H NMR (400 MHz, CDCl$_3$)

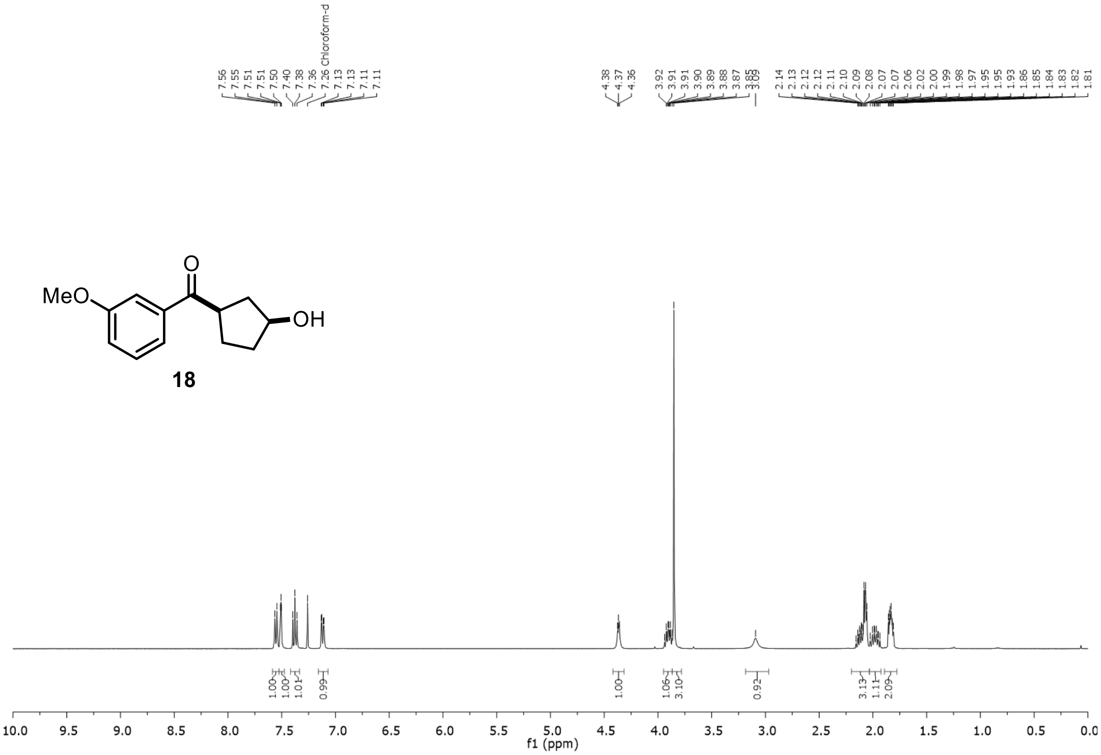

^{13}C NMR (101 MHz, CDCl$_3$)

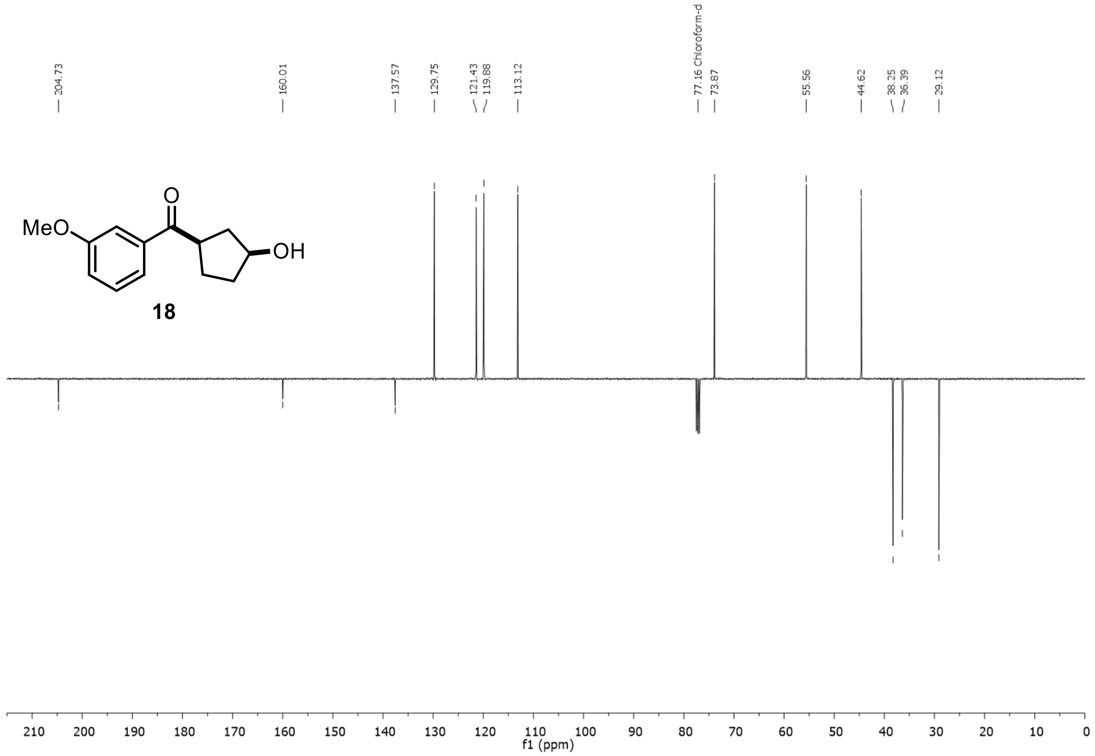

19: *syn*-(3-hydroxycyclohexyl)undecan-1-one

^{1}H NMR (600 MHz, $CDCl_3$)

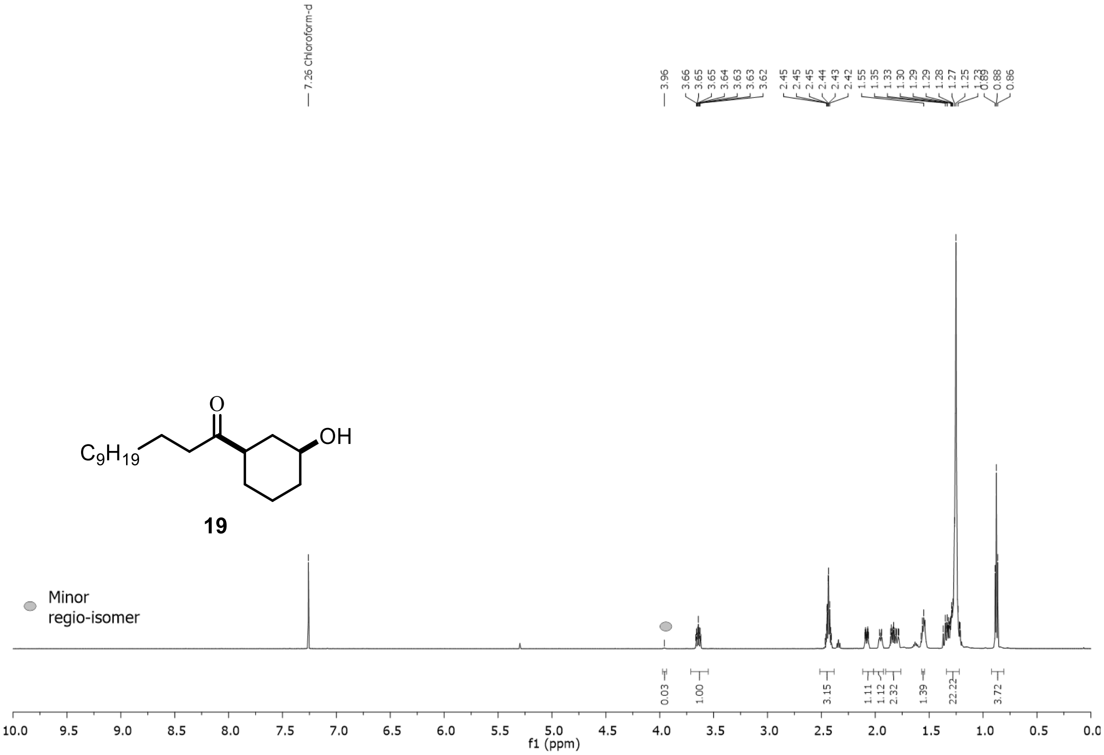

^{13}C NMR (151 MHz, $CDCl_3$)

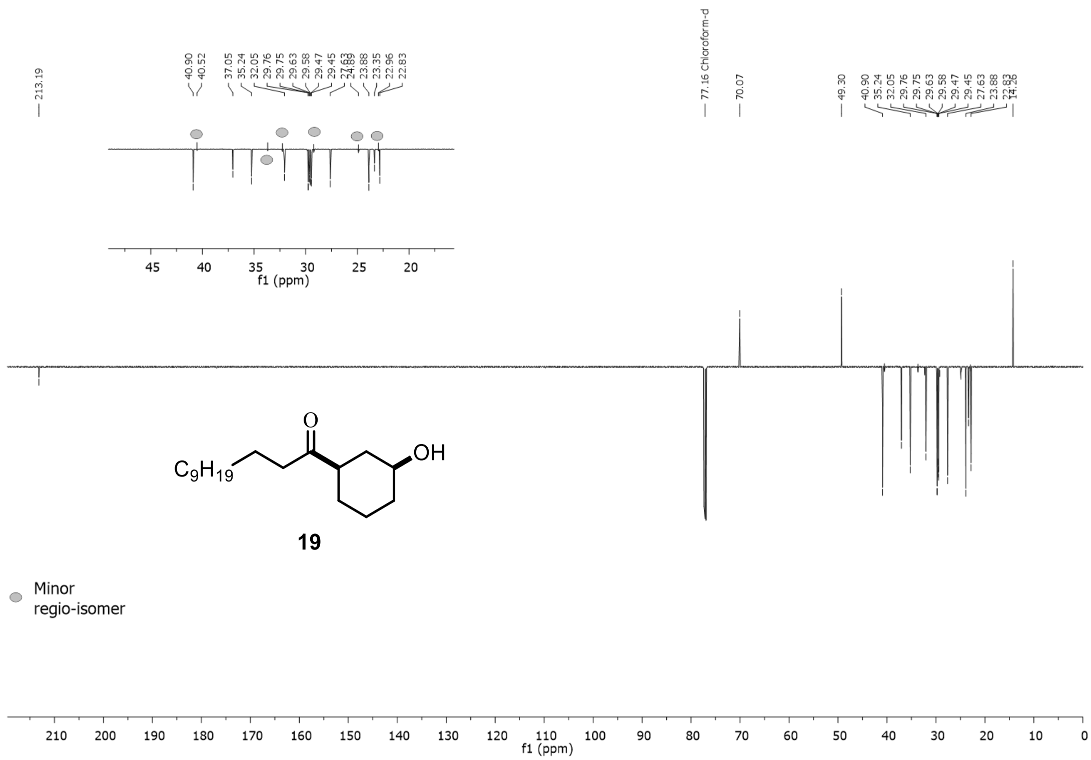

20: *syn*-1-(3-hydroxycyclohexyl)-3-methylbutan-1-one

^{1}H NMR (400 MHz, $CDCl_3$)

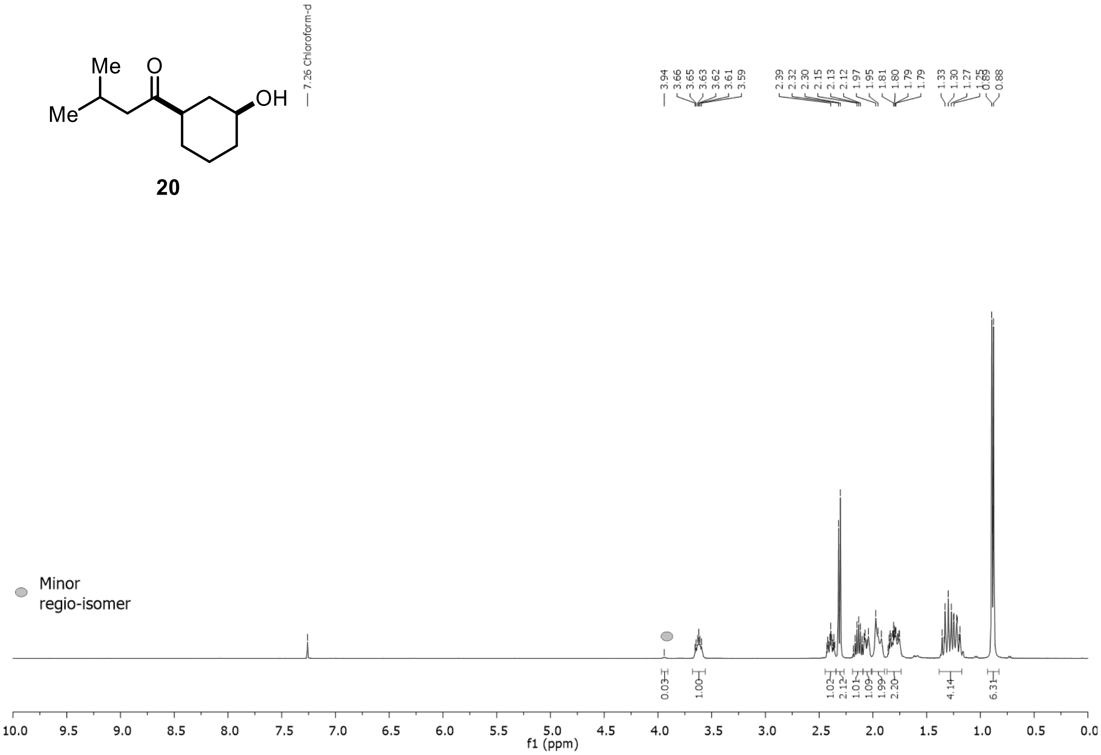

^{13}C NMR (101 MHz, $CDCl_3$)

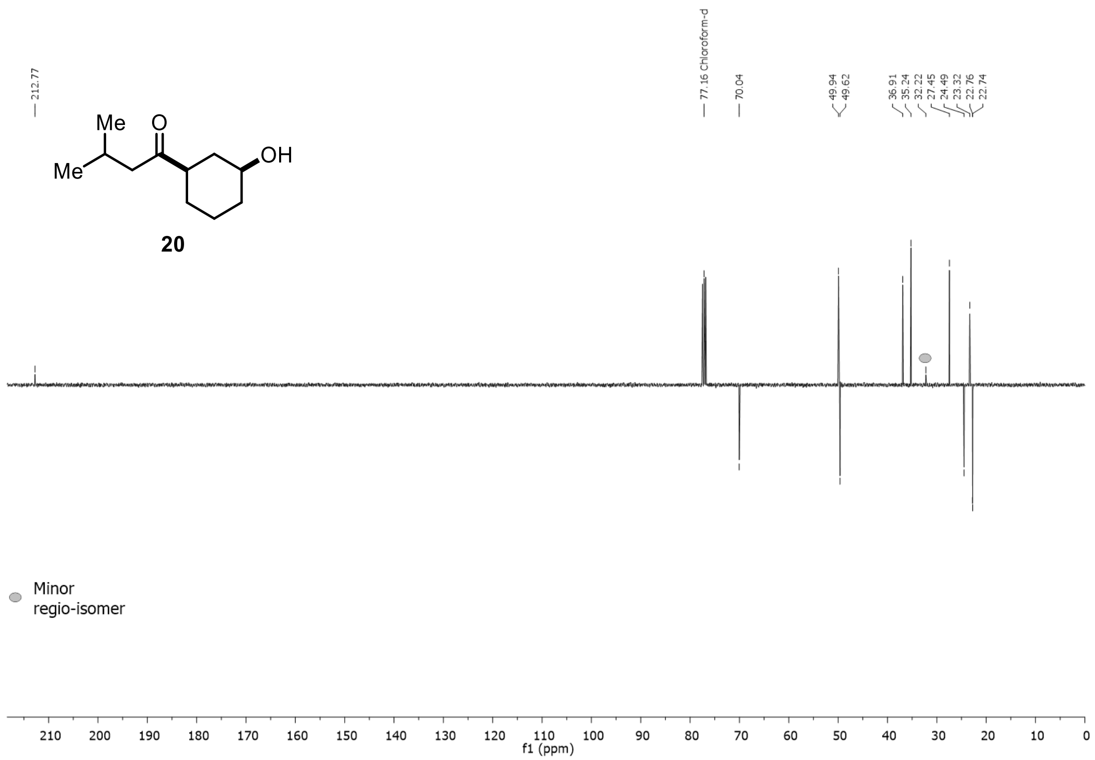

21: ***syn*****-1-(3-hydroxycyclopentyl)-2-methylpropan-1-one**

^{1}H NMR (600 MHz, $CDCl_3$)

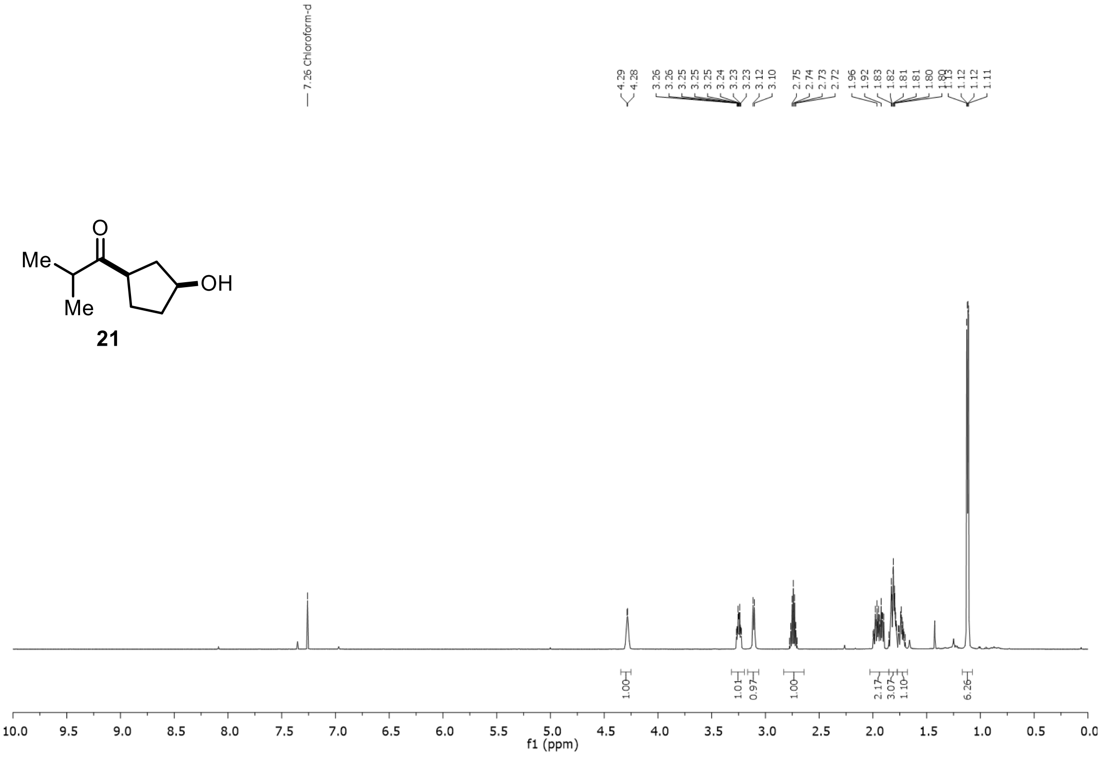

^{13}C NMR (176 MHz, $CDCl_3$)

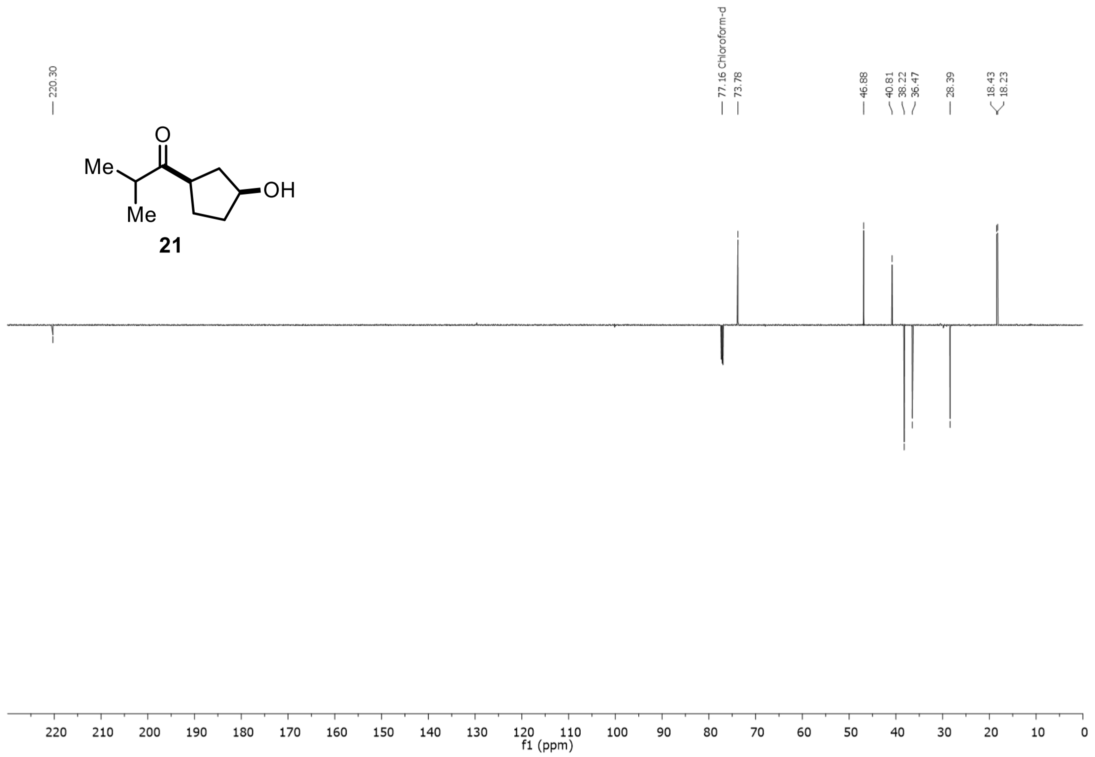

22: *syn*-4-chloro-1-(3-hydroxycyclohexyl)butan-1-one

^{1}H NMR (400 MHz, $CDCl_3$)

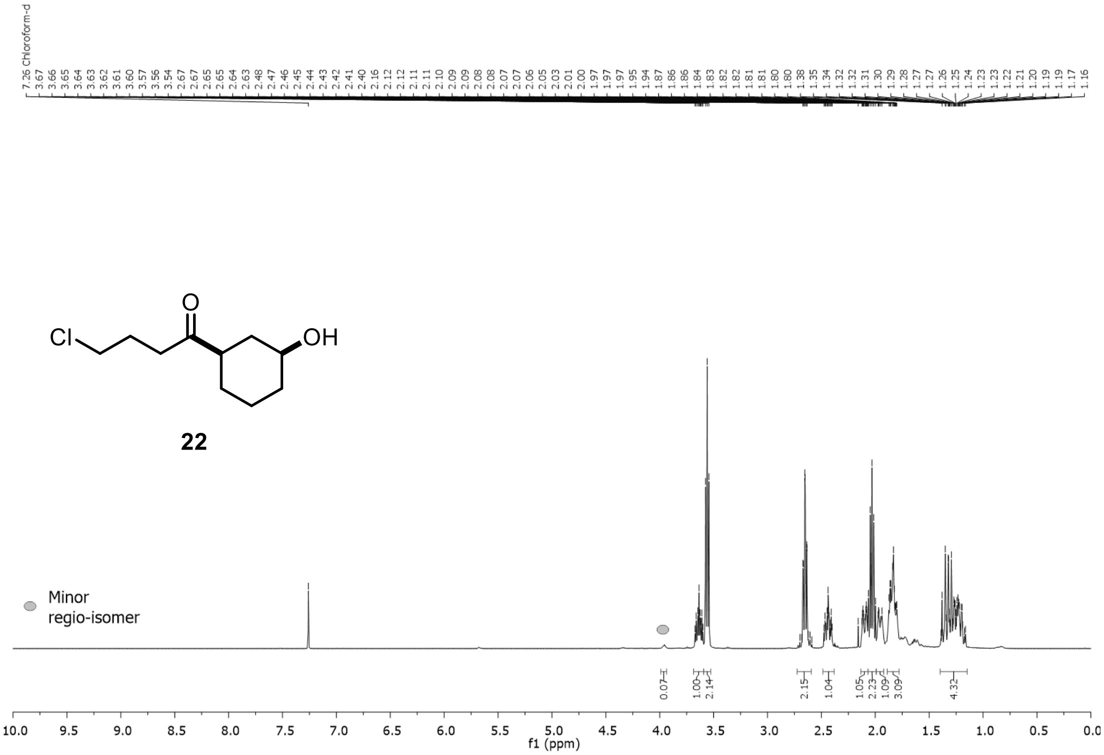

^{13}C NMR (101 MHz, $CDCl_3$)

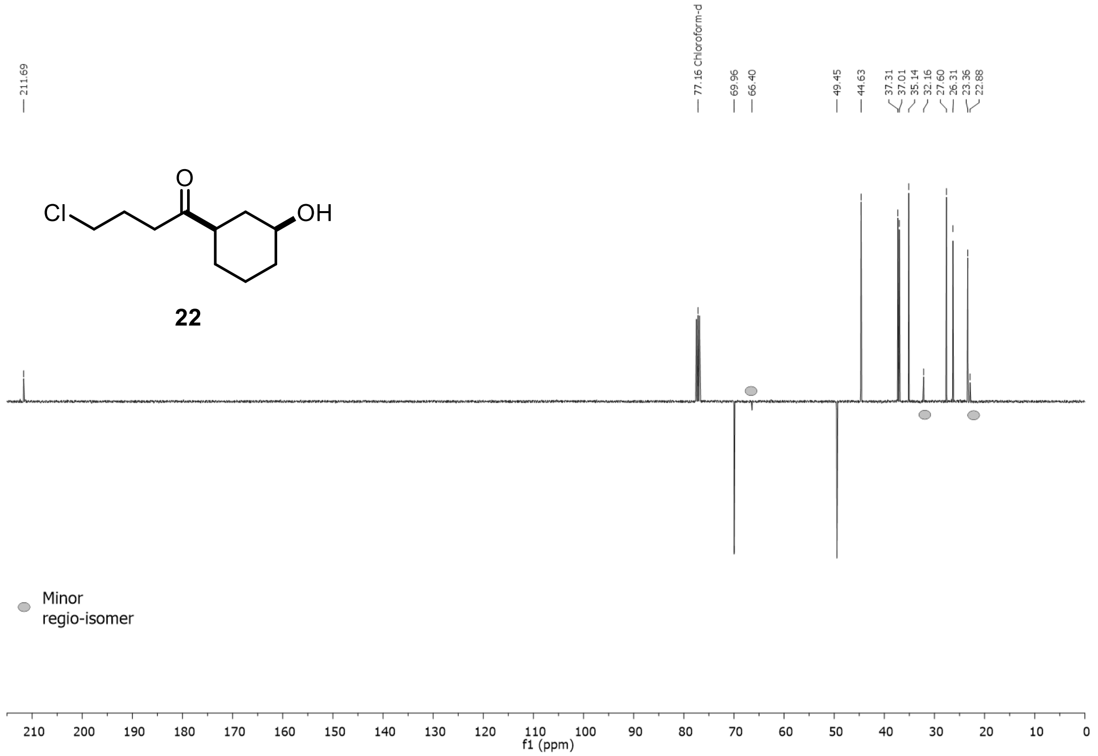

23: syn-2-chloro-1-(3-hydroxycyclohexyl)propan-1-one

^{1}H NMR (400 MHz, $CDCl_3$)

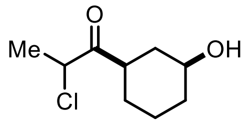

23

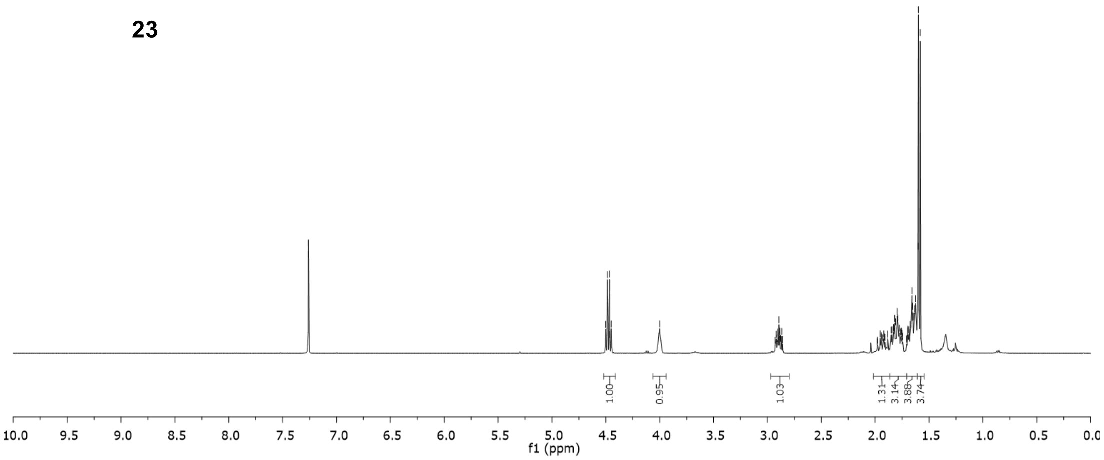

^{13}C NMR (101 MHz, $CDCl_3$)

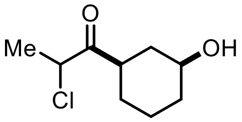

23

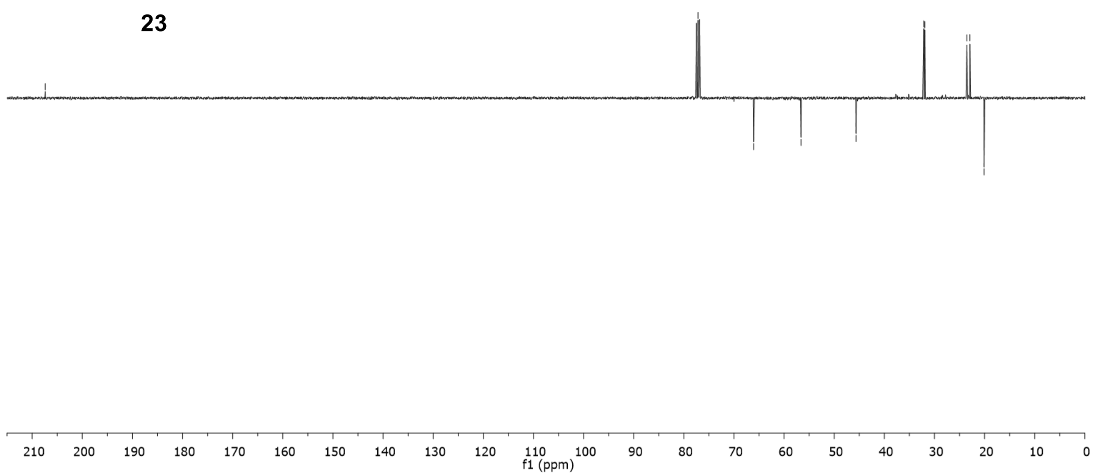

24: ***syn*-1-(3-hydroxycyclohexyl)-2-(4-(trifluoromethyl)phenyl)ethan-1-one**

^{1}H NMR (400 MHz, $CDCl_3$)

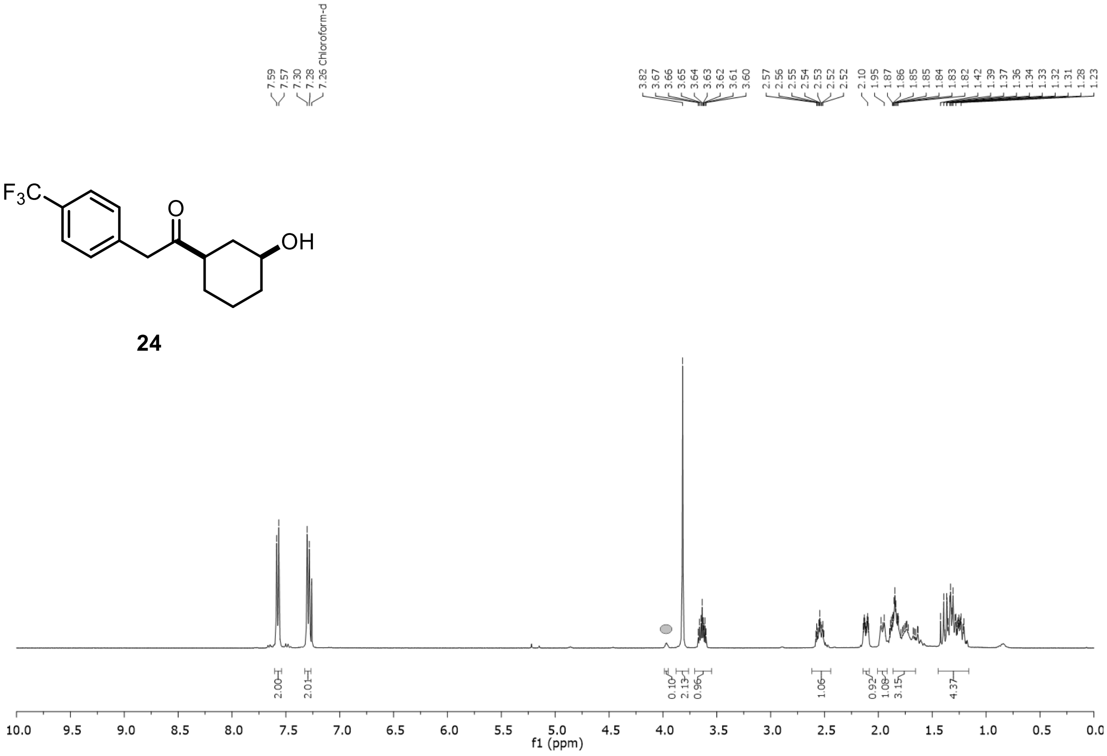

^{13}C NMR (101 MHz, $CDCl_3$)

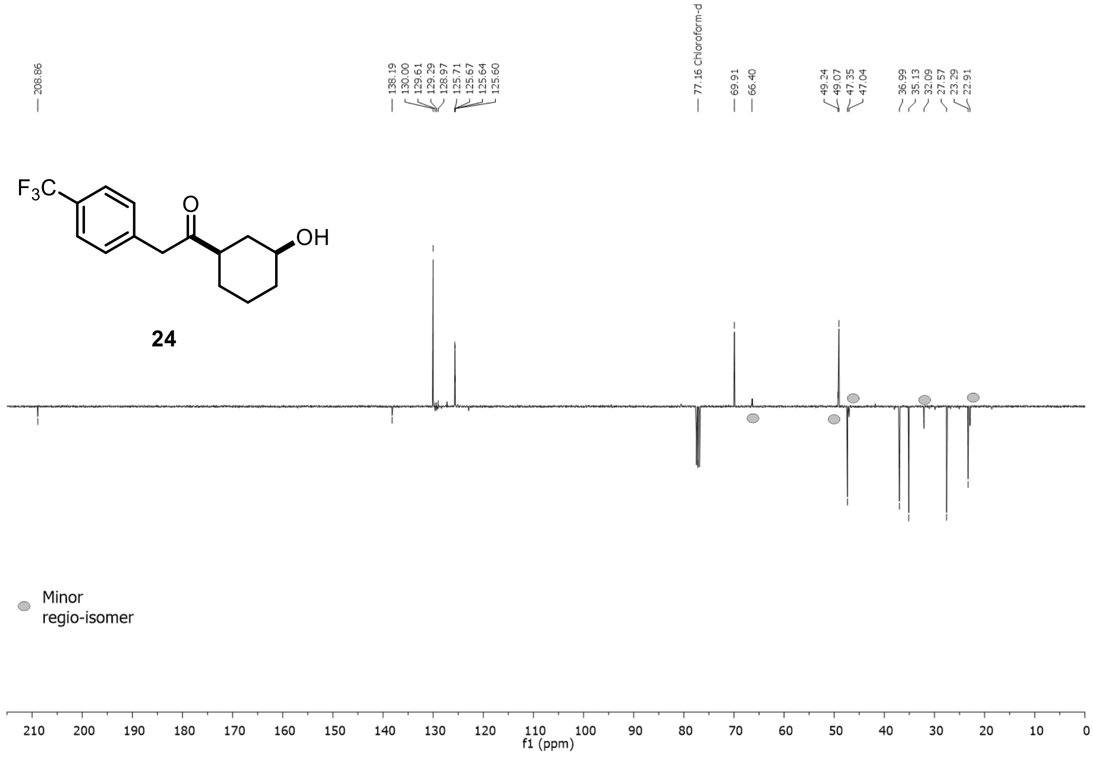

^{19}F NMR (377 MHz, $CDCl_3$)

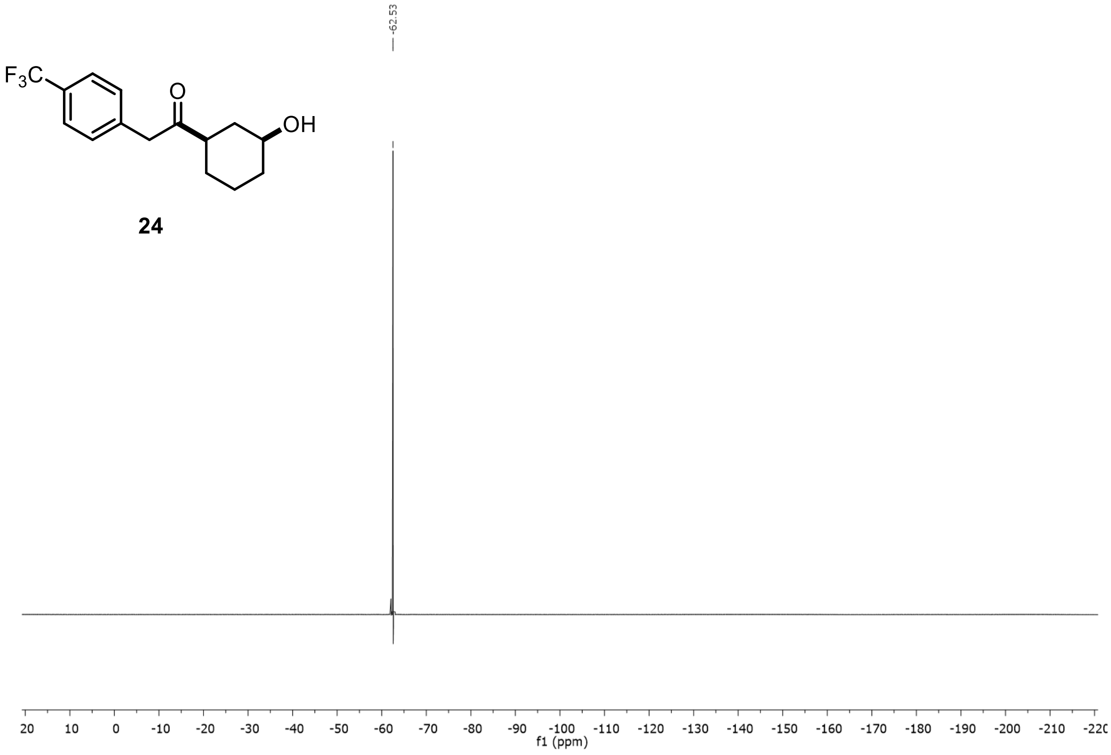

25: *syn*-((*E*)-3-hydroxycyclohexyl)(4-(trifluoromethoxy)phenyl)methanone

^{1}H NMR (400 MHz, $CDCl_3$)

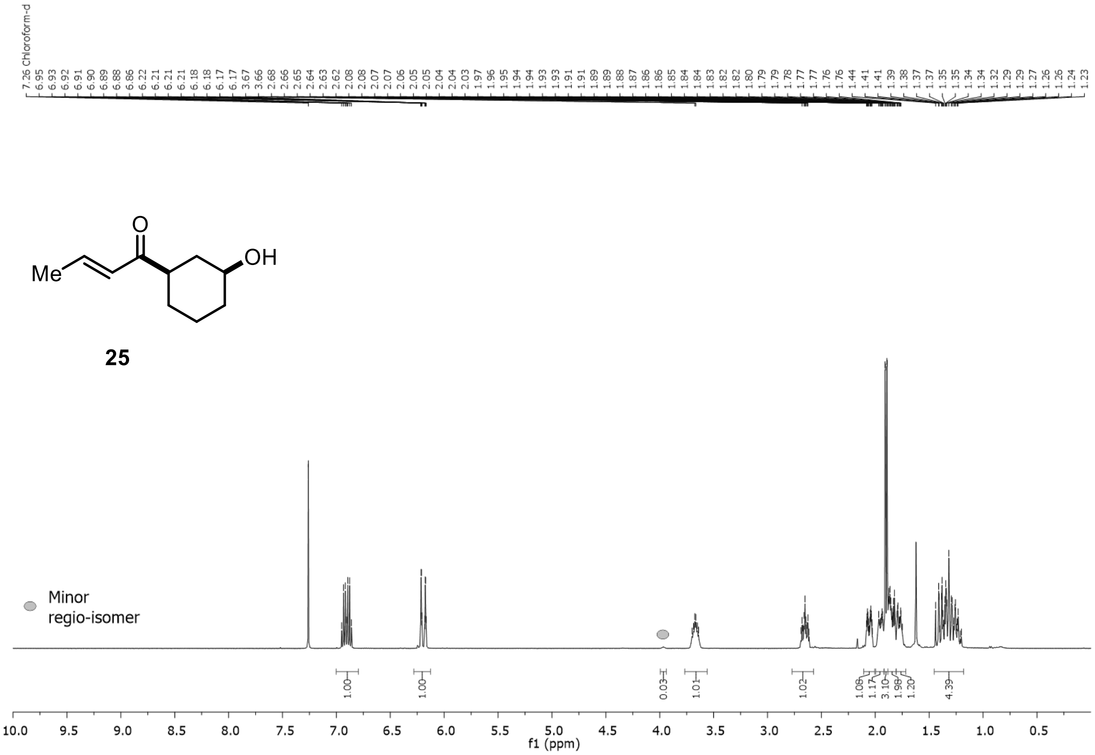

^{13}C NMR (101 MHz, $CDCl_3$)

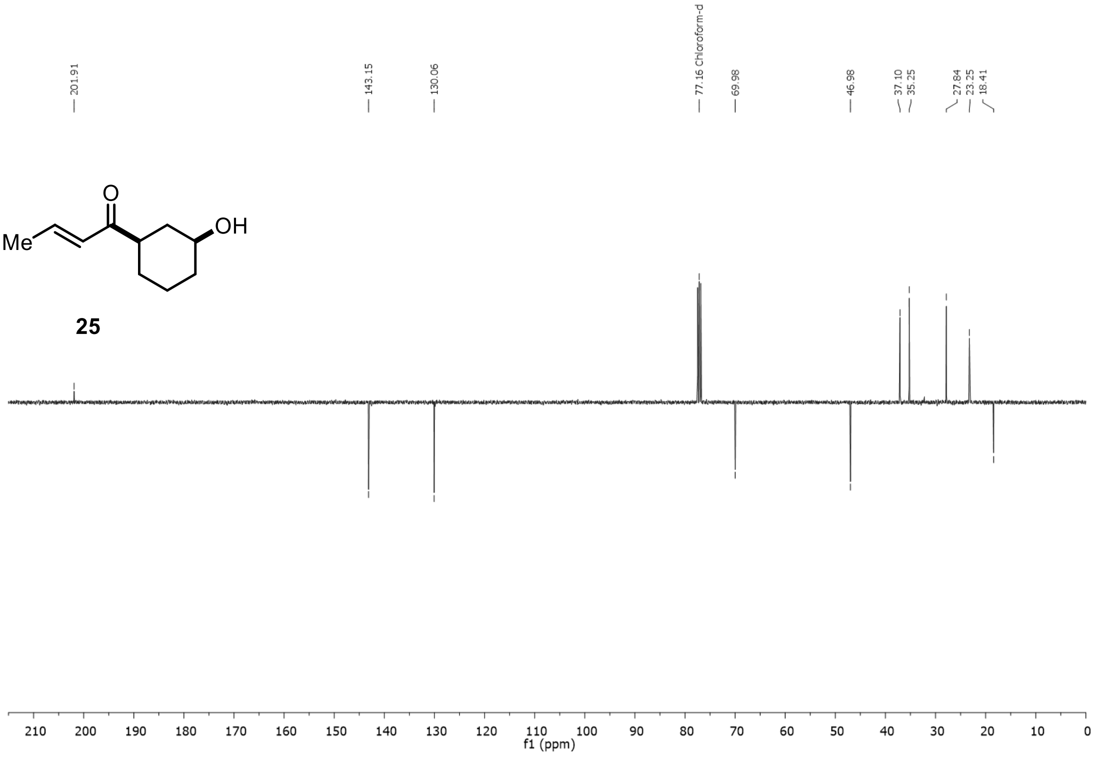

26: *syn*-furan-3-yl(3-hydroxycyclohexyl)methanone

^{1}H NMR (400 MHz, $CDCl_3$)

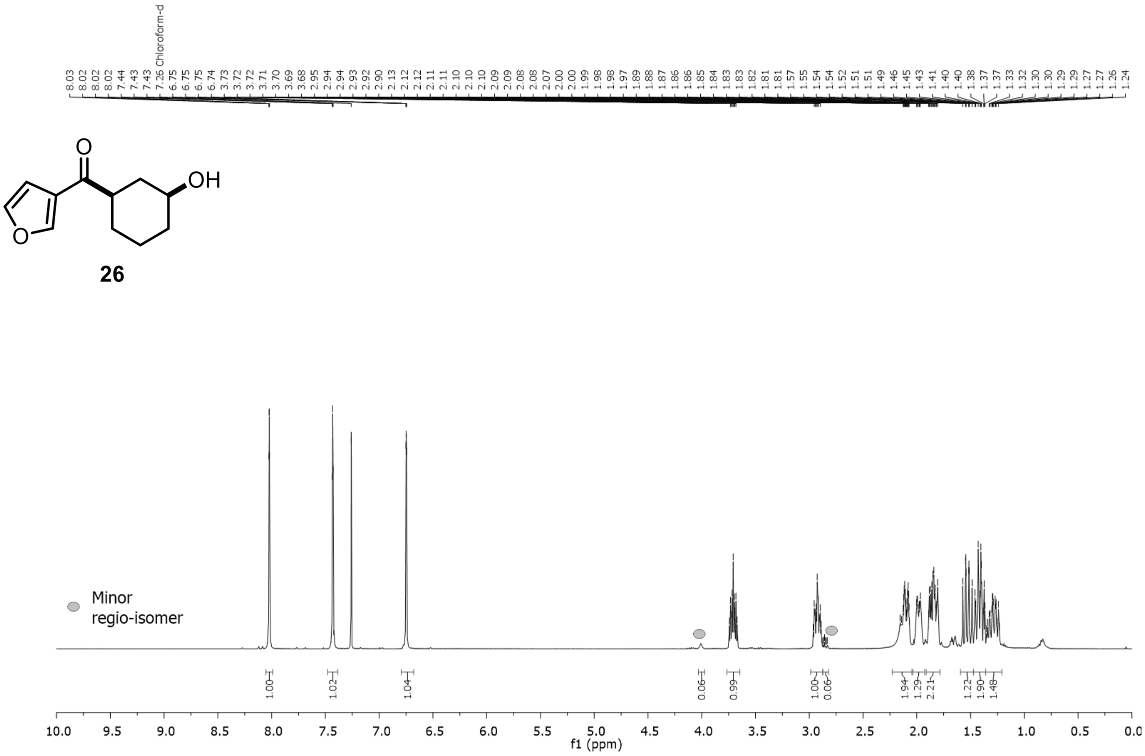

^{13}C NMR (156 MHz, $CDCl_3$)

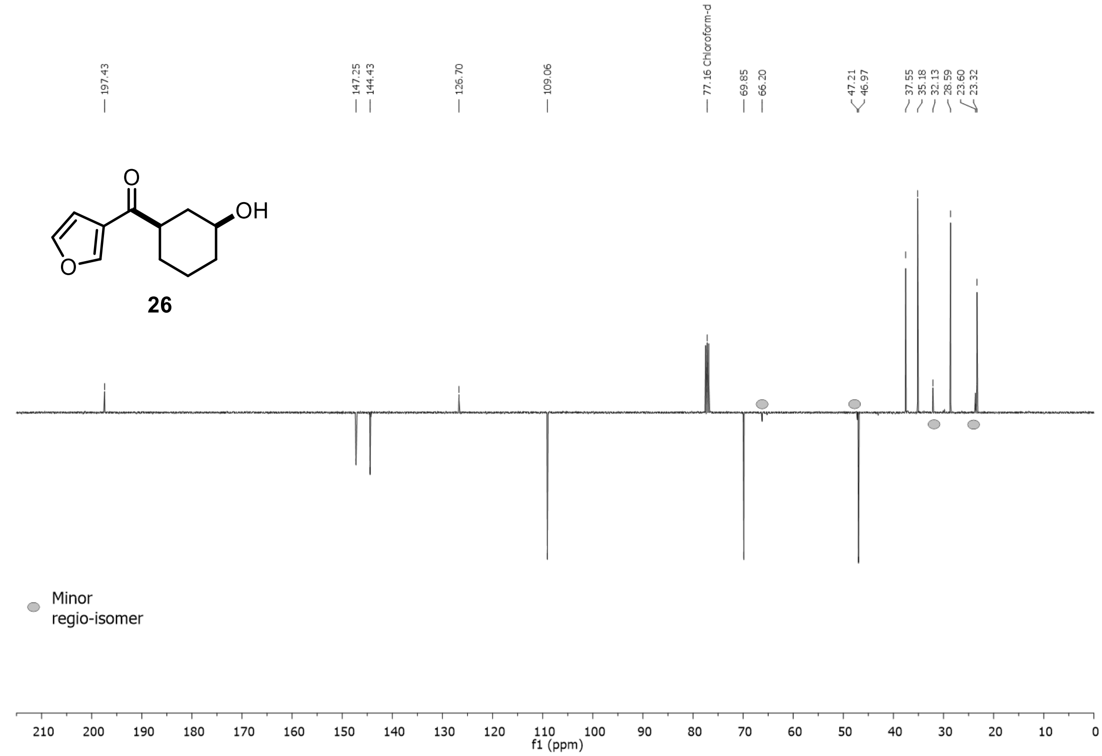

27: ***syn*-benzo[b]thiophen-2-yl(3-hydroxycyclohexyl)methanone**

^{1}H NMR (700 MHz, CDCl$_3$)

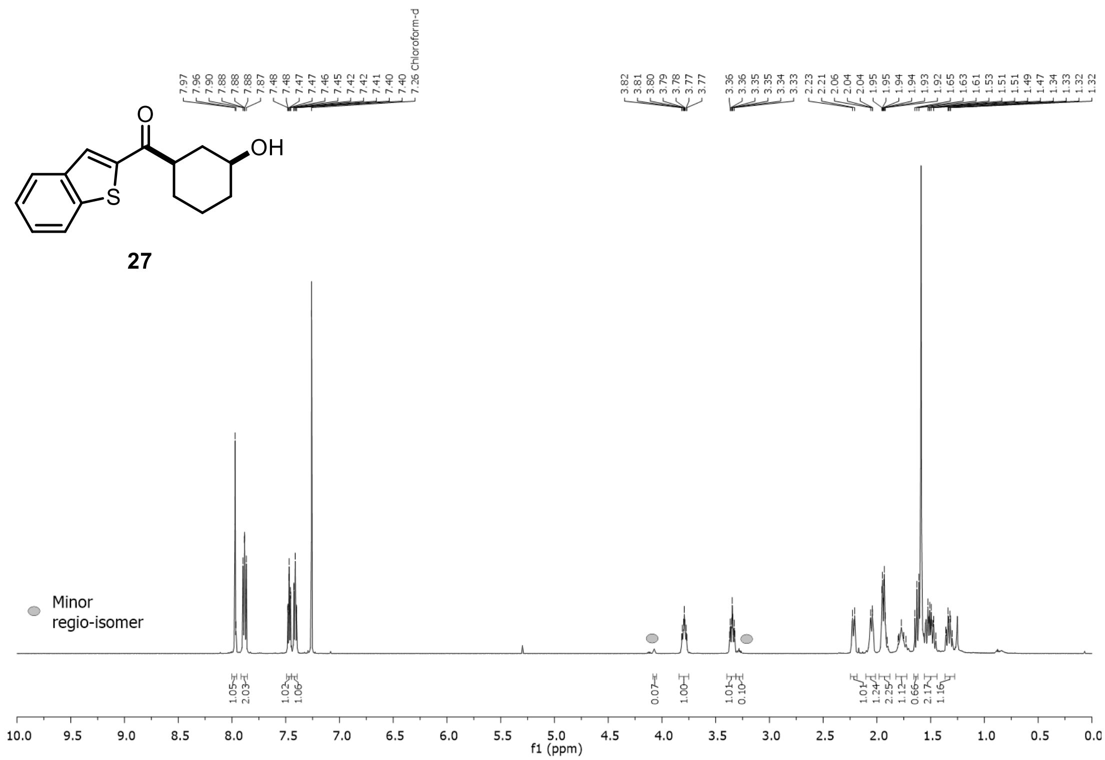

^{13}C NMR (176 MHz, CDCl$_3$)

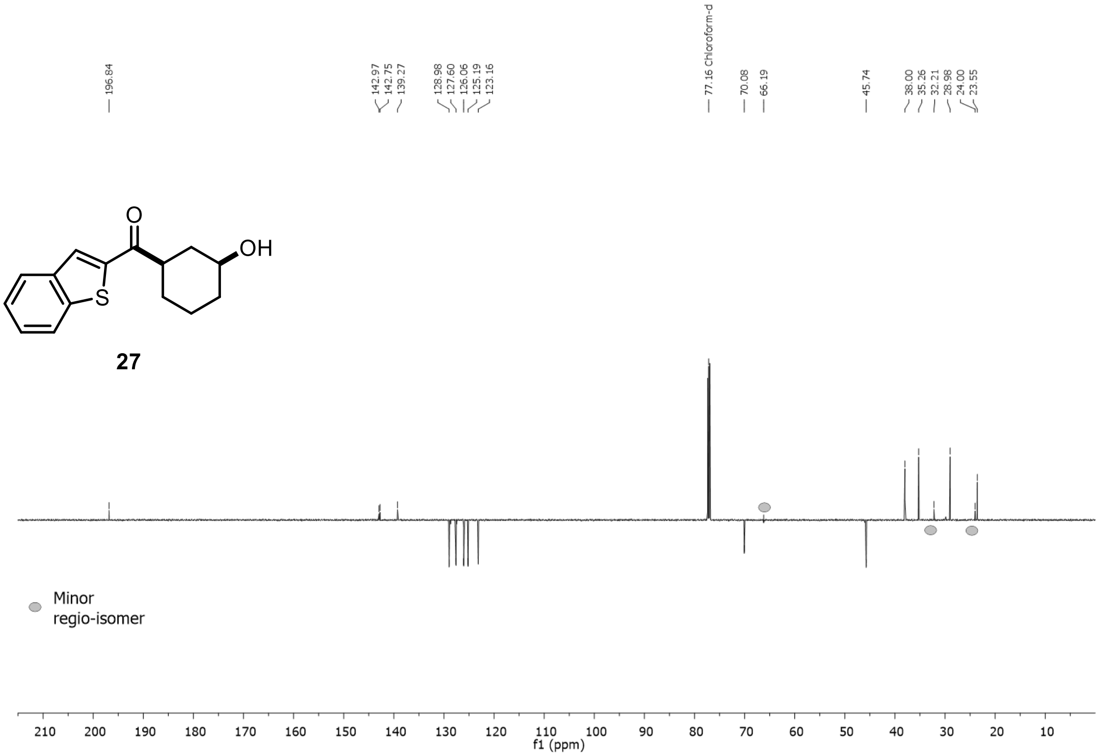

28: ((1*S**,2*S**,4*S**,7*R**)-2-hydroxybicyclo[2.2.1]heptan-7-yl)(phenyl)methanone

^{1}H NMR (400 MHz, $CDCl_3$)

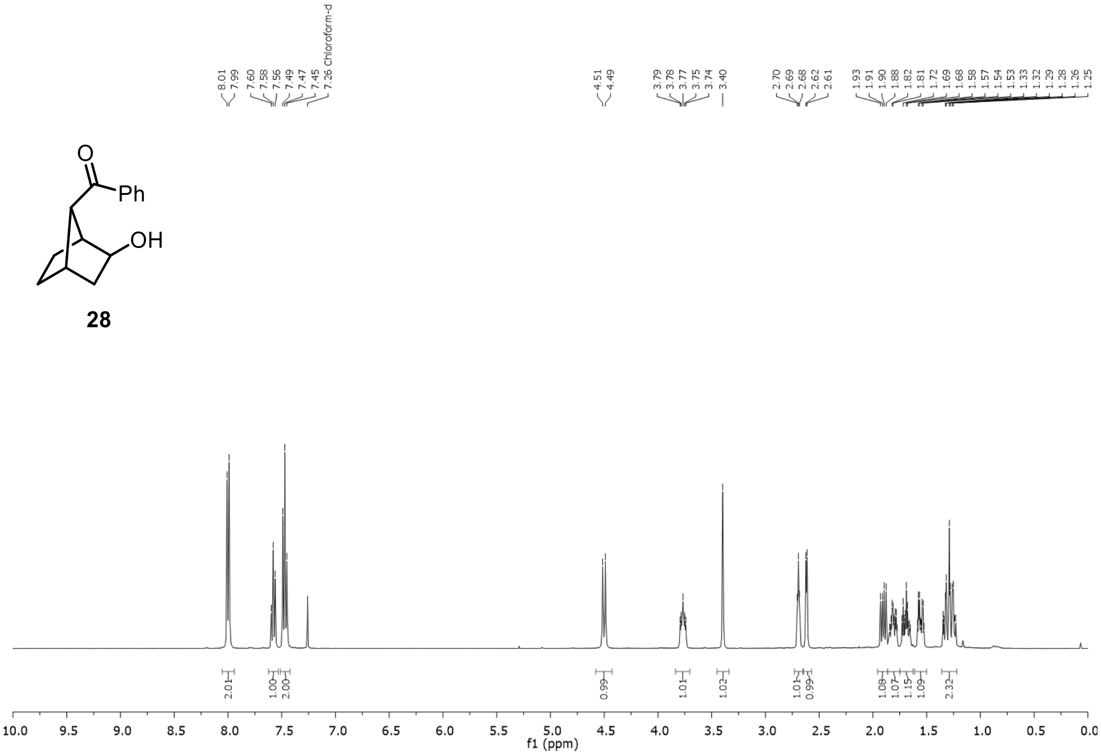

^{13}C NMR (101 MHz, $CDCl_3$)

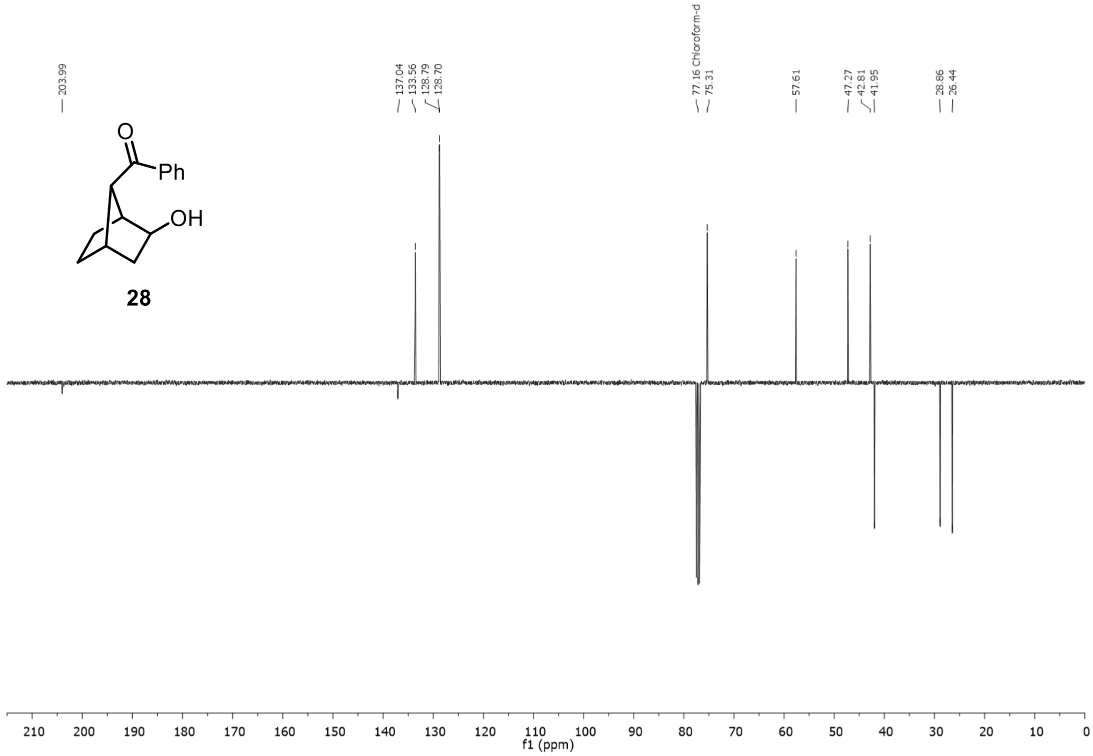

55: 4-hydroxy-1-phenylnonan-1-one

^{1}H NMR (400 MHz, $CDCl_3$)

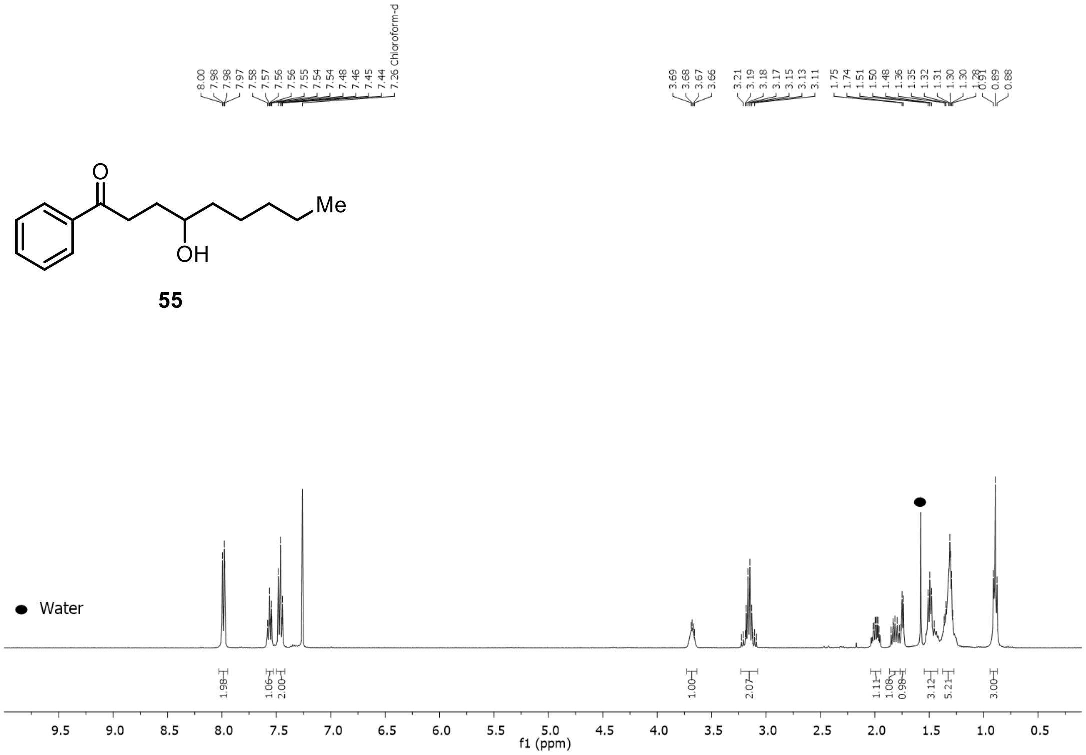

^{13}C NMR (151 MHz, $CDCl_3$)

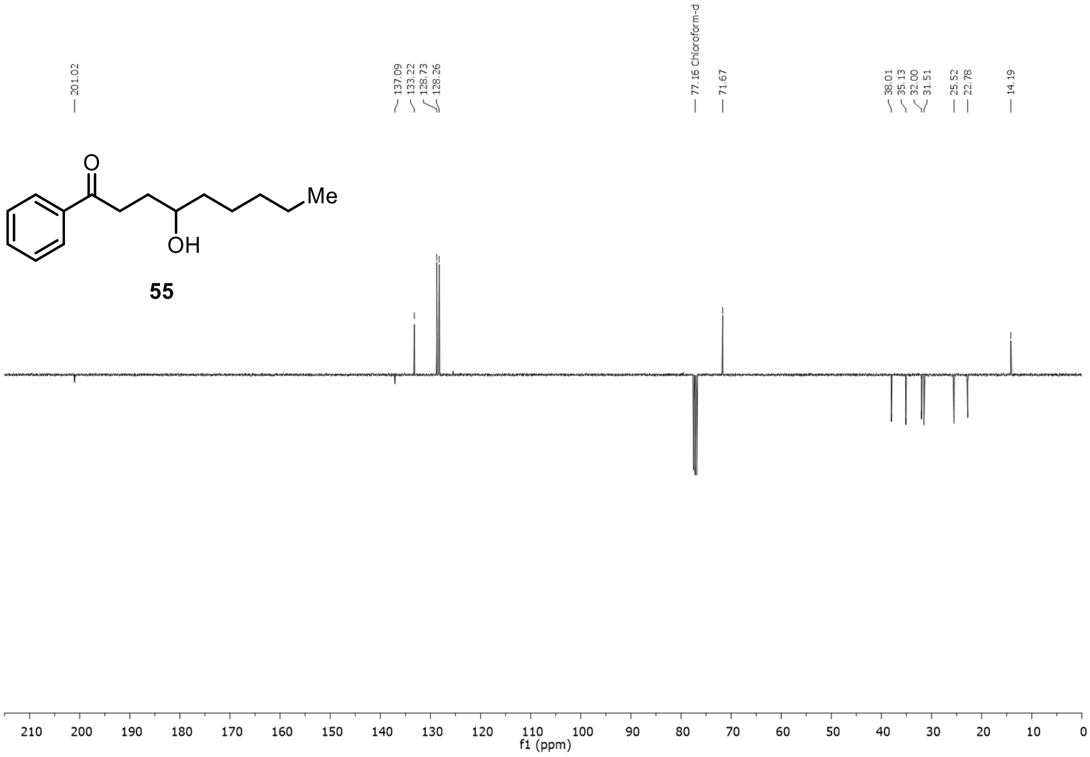

56: 4-hydroxy-1-phenyldodecan-1-one

^{1}H NMR (400 MHz, $CDCl_3$)

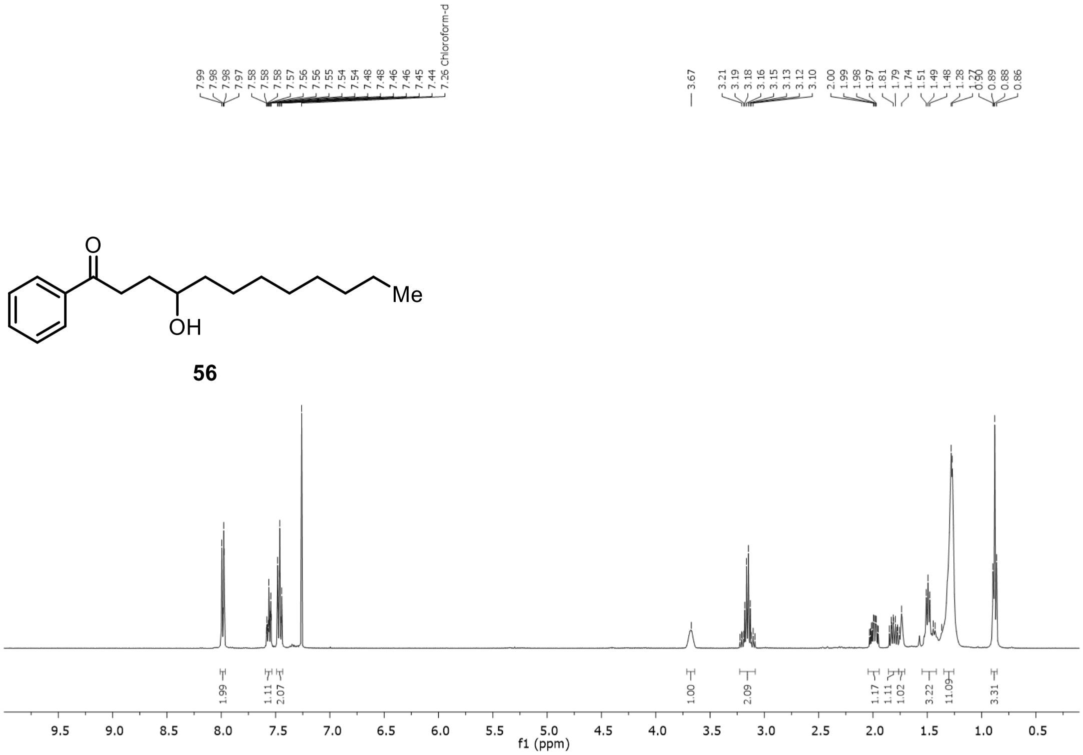

^{13}C NMR (151 MHz, $CDCl_3$)

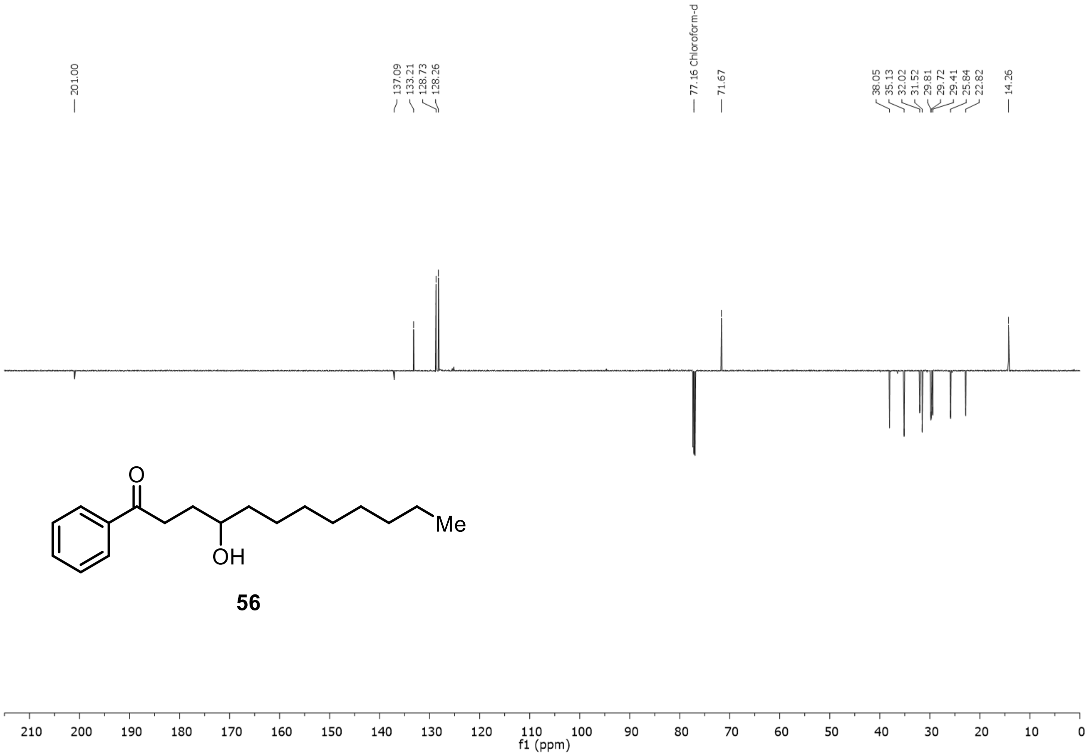

57: *syn*-(3-hydroxycyclohexyl)(4-methoxyphenyl)methanone

^{1}H NMR (700 MHz, $CDCl_3$)

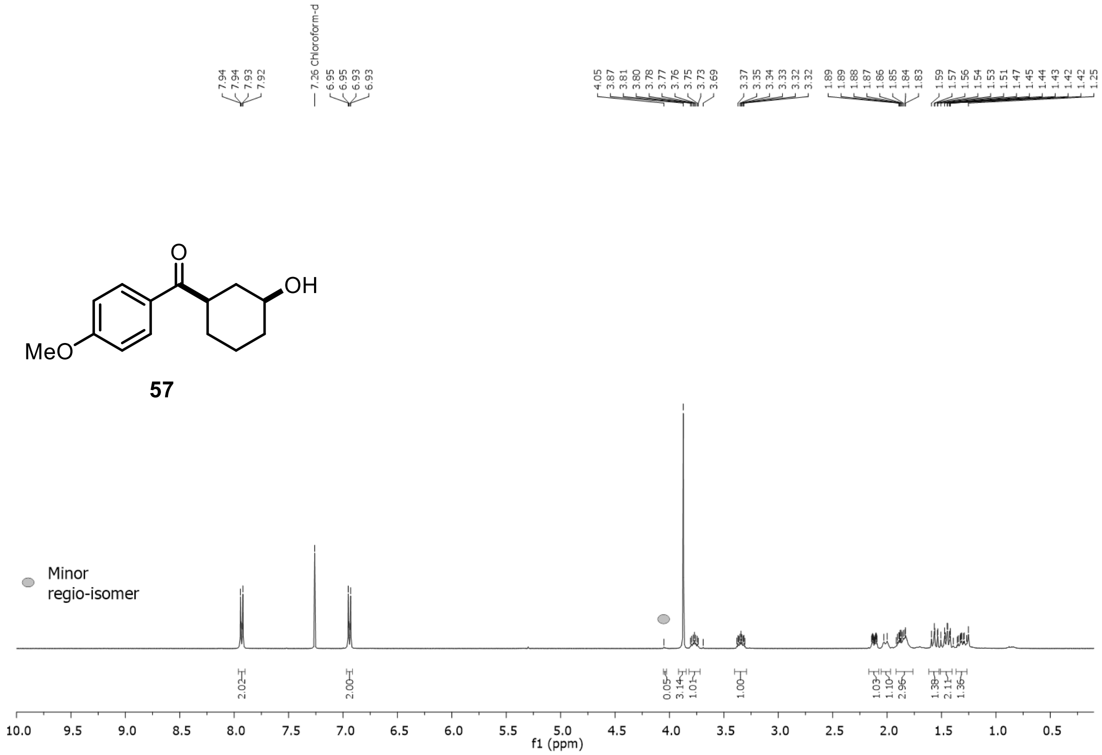

^{13}C NMR (176 MHz, $CDCl_3$)

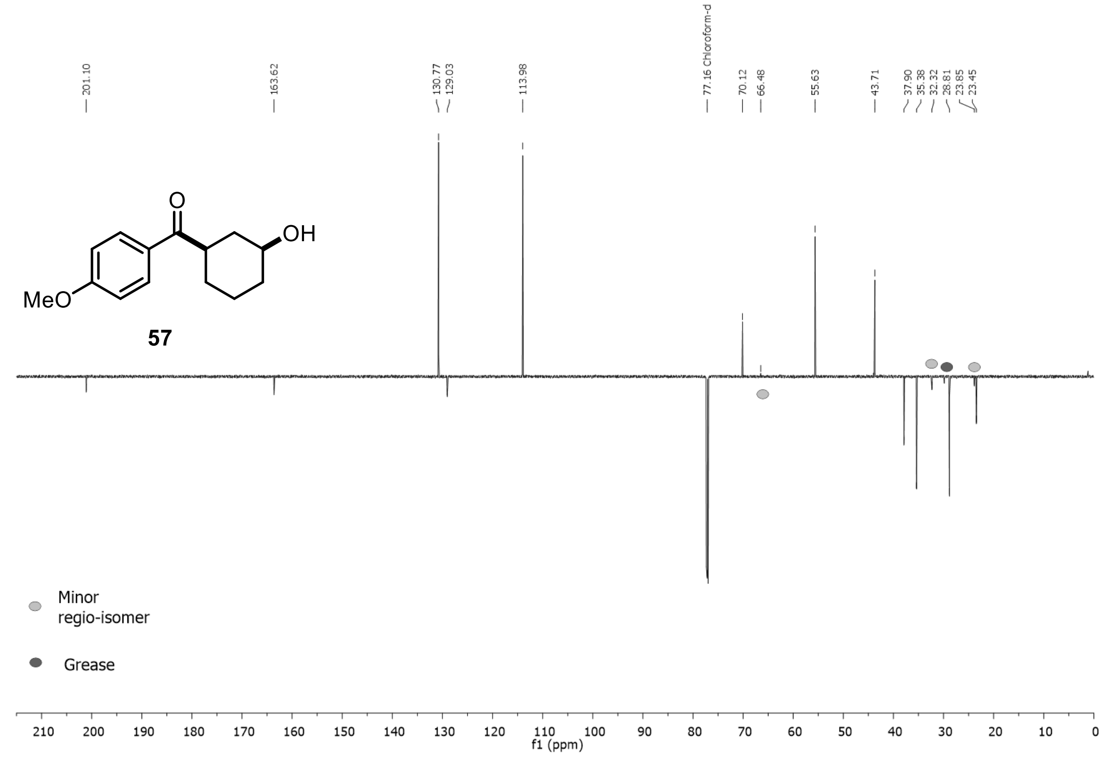

58: *syn*-(3-hydroxycyclohexyl)(*o*-tolyl)methanone

^{1}H NMR (400 MHz, $CDCl_3$)

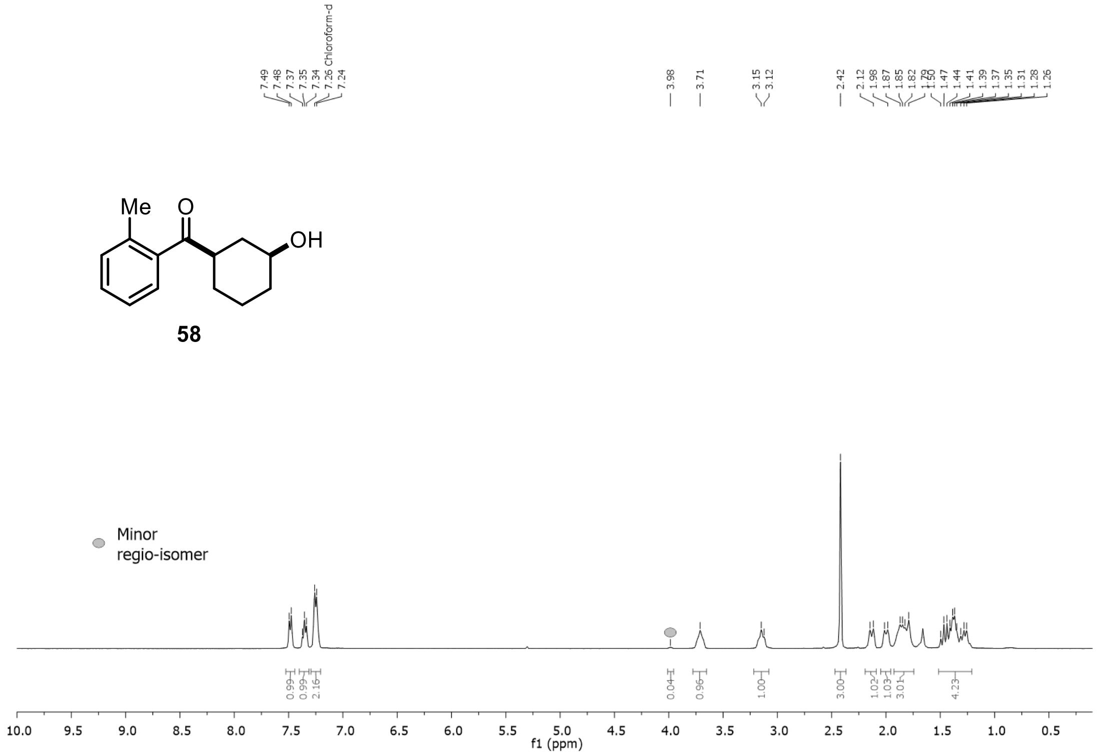

^{13}C NMR (101 MHz, $CDCl_3$)

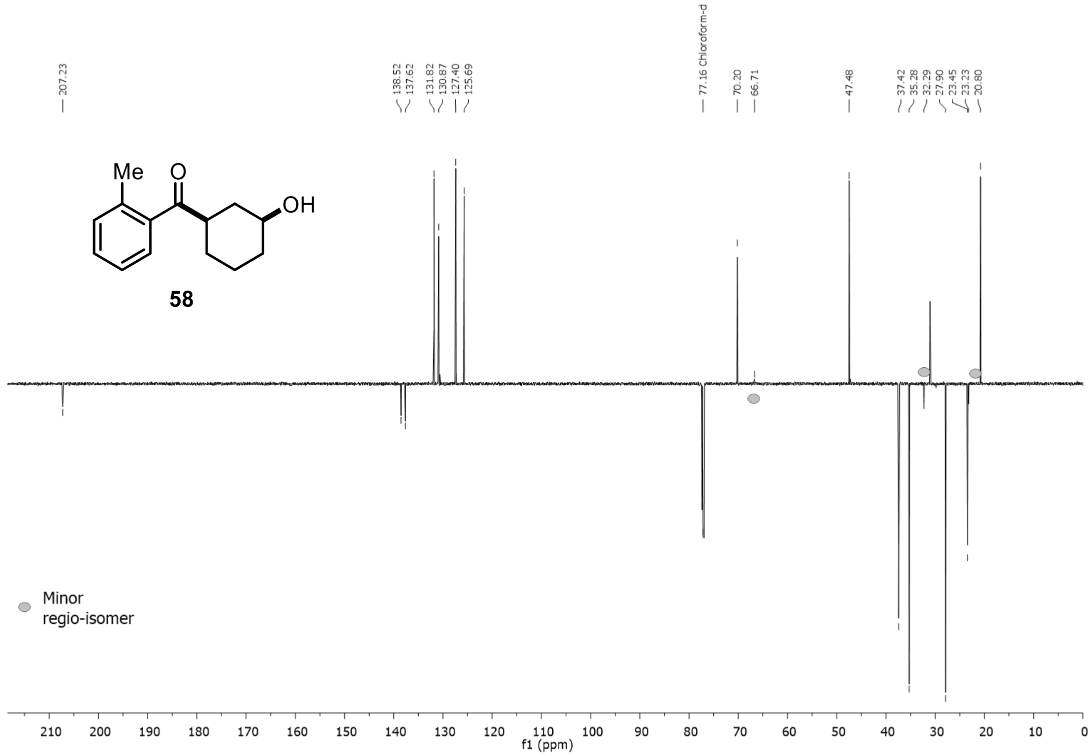

59: *syn*-fluorophenyl-(3-hydroxycyclohexyl)methanone

^{1}H NMR (600 MHz, $CDCl_3$)

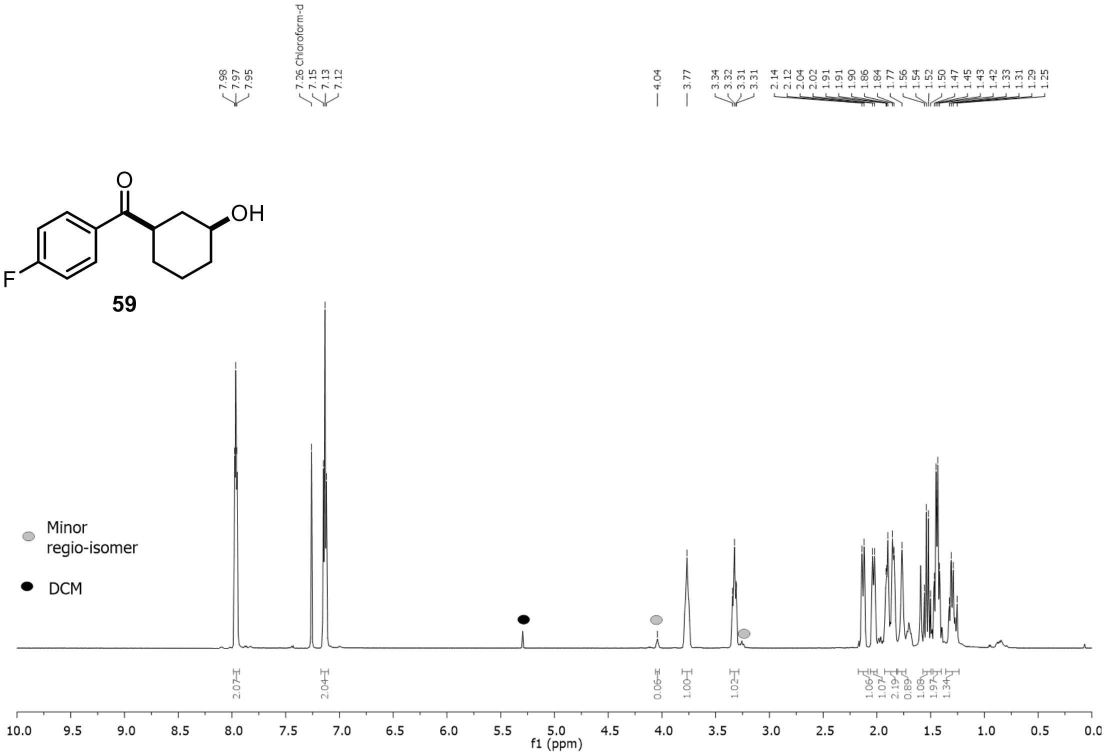

^{13}C NMR (151 MHz, $CDCl_3$)

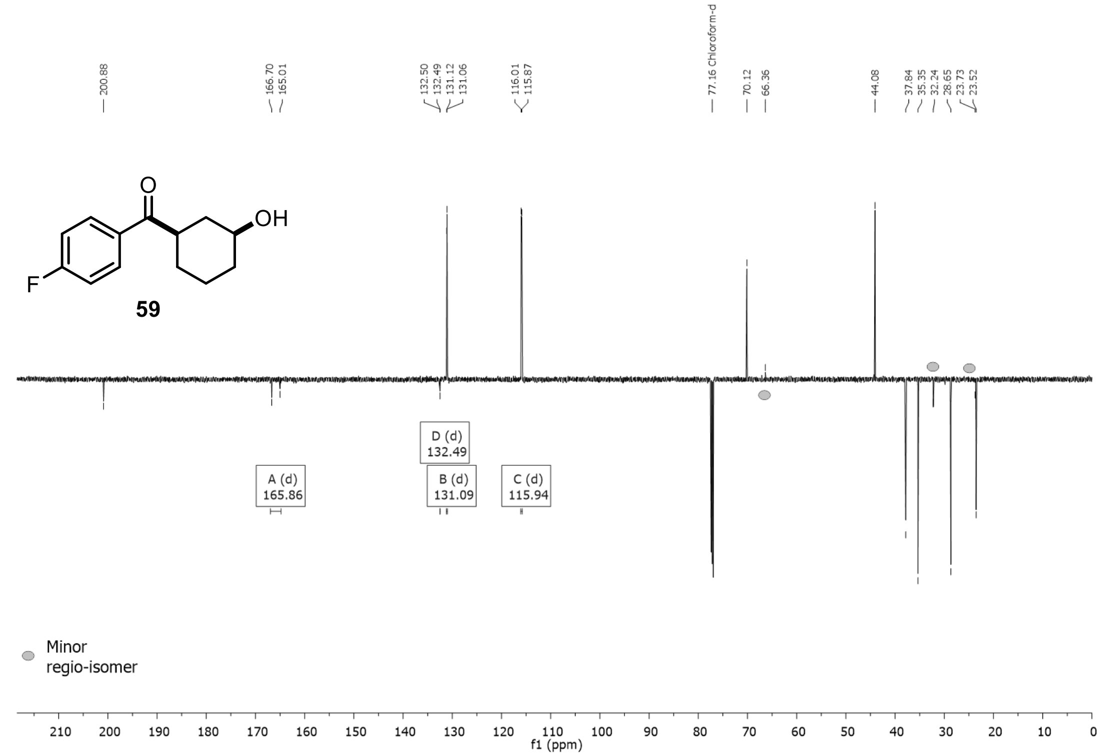

^{19}F NMR (101 MHz, CDCl$_3$)

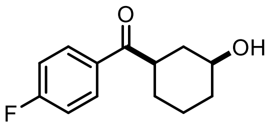

59

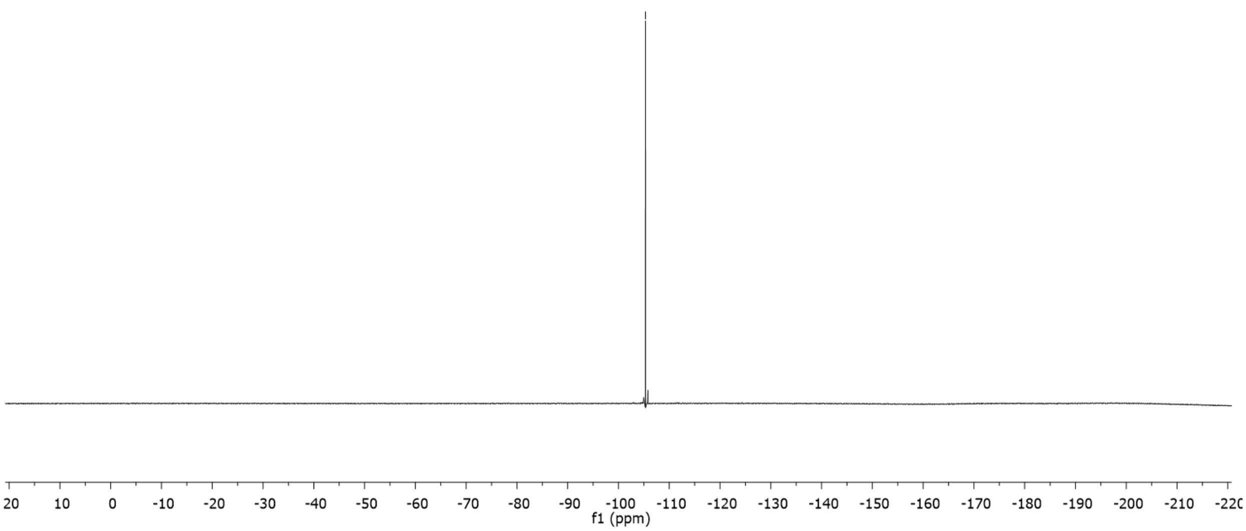

60: *syn*-(3-hydroxycyclohexyl)(4-(trifluoromethoxy)phenyl)methanone

^{1}H NMR (400 MHz, $CDCl_3$)

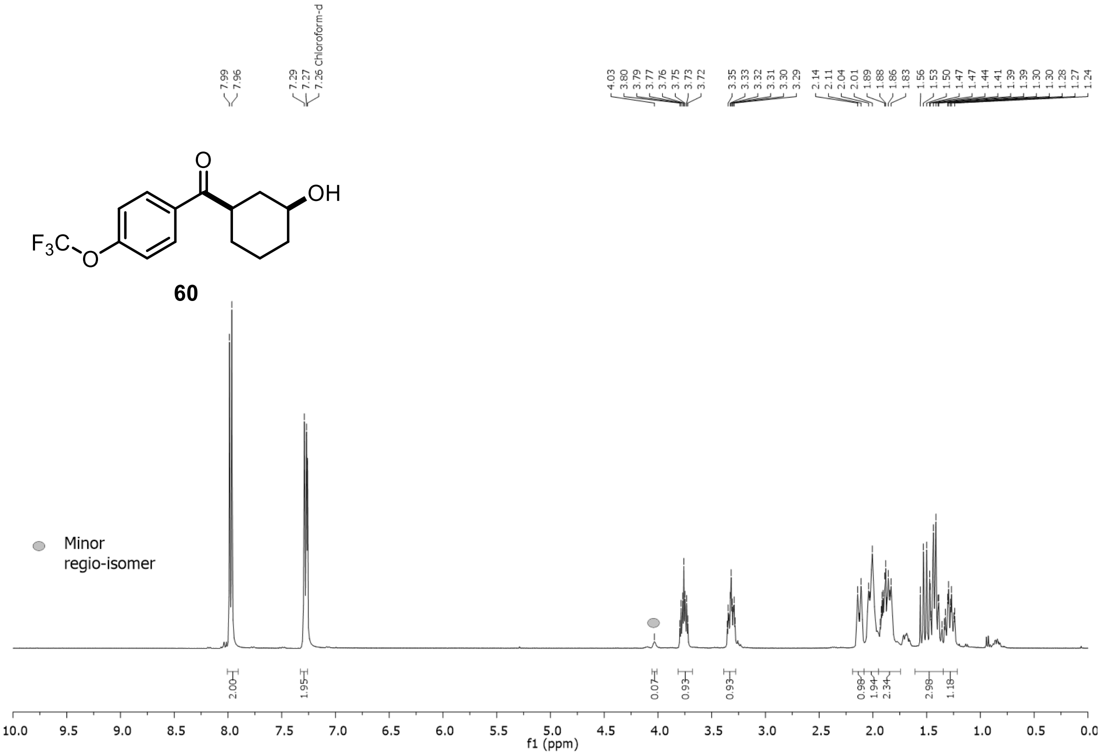

^{13}C NMR (101 MHz, $CDCl_3$)

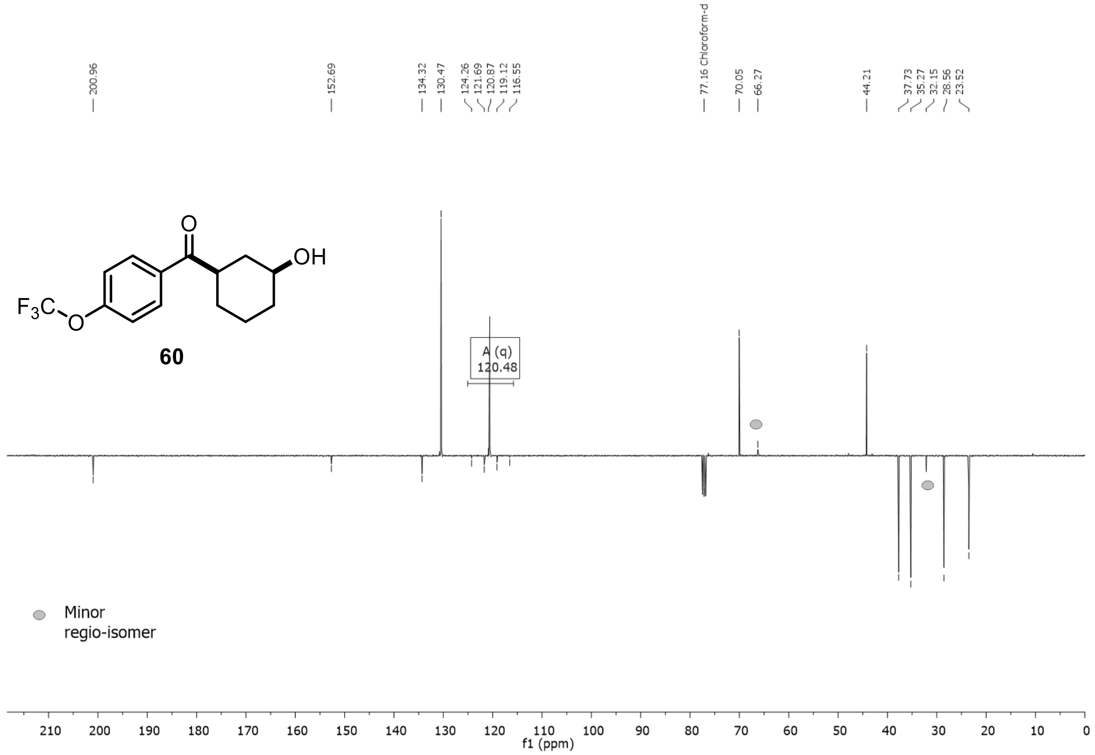

^{19}F NMR (377 MHz, $CDCl_3$)

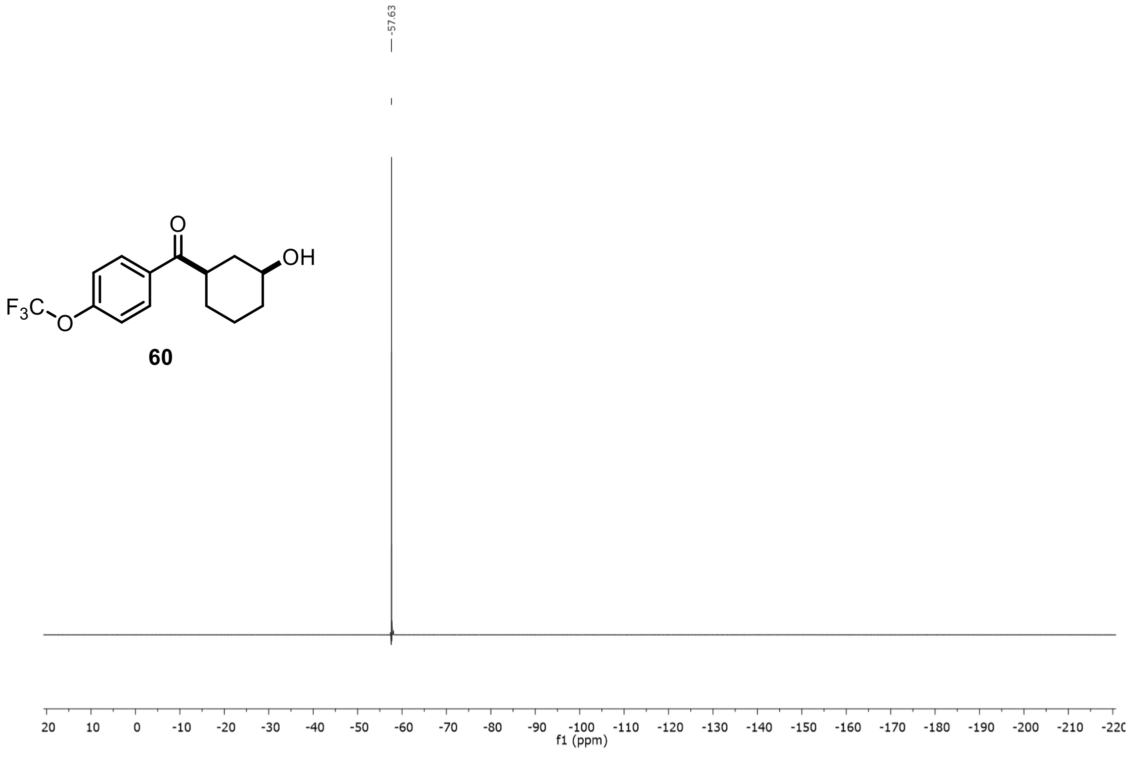

61: *syn*-(3-hydroxycyclopentyl)(phenyl)methanone

^{1}H NMR (400 MHz, $CDCl_3$)

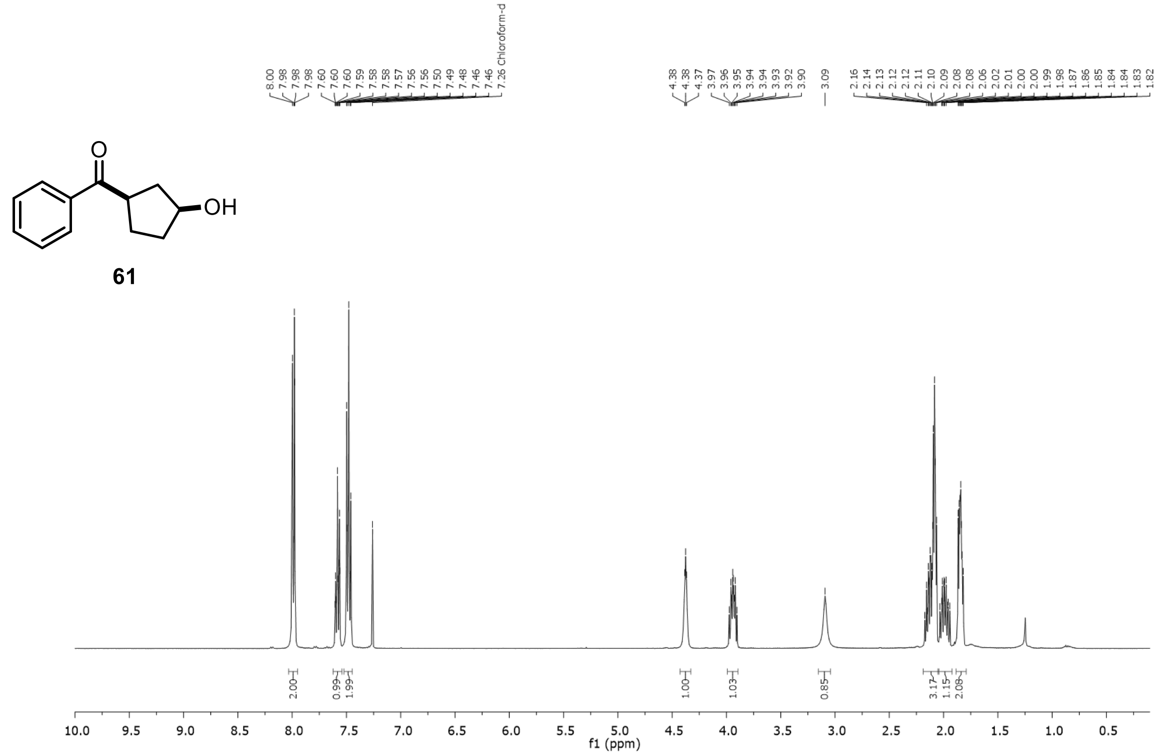

^{13}C NMR (101 MHz, $CDCl_3$)

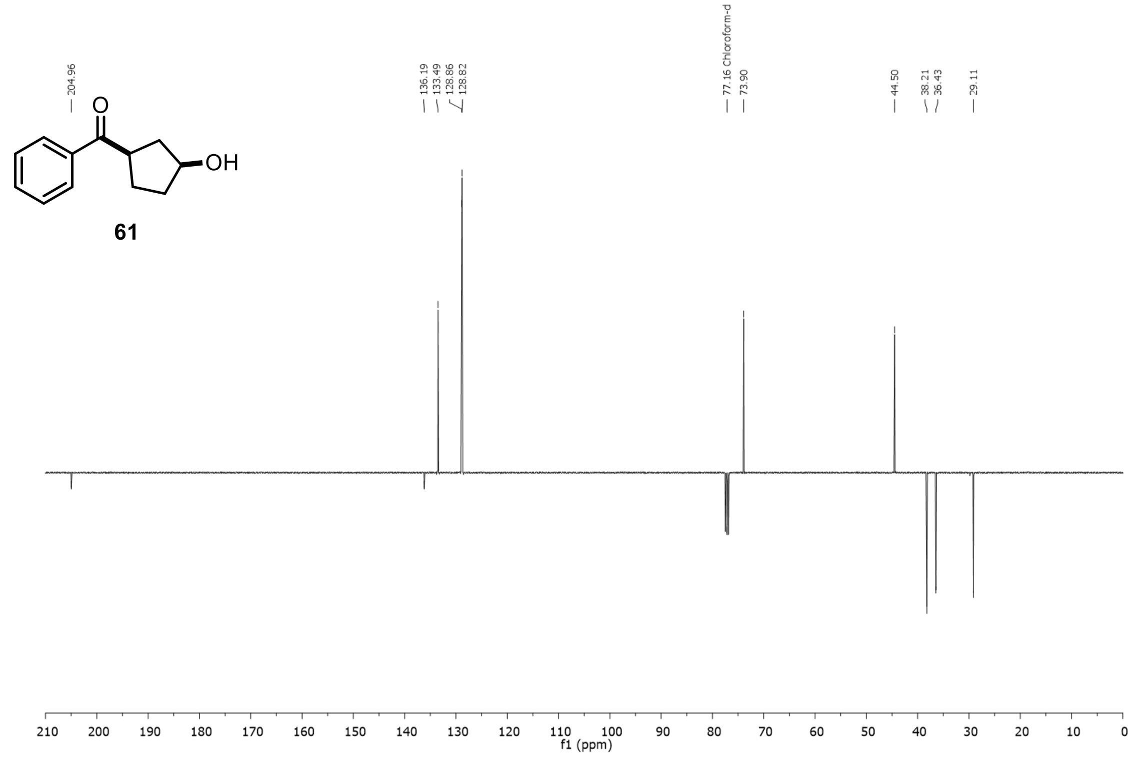

62: *syn*-(4-fluorophenyl)(3-hydroxycyclopentyl)methanone

^{1}H NMR (600 MHz, $CDCl_3$)

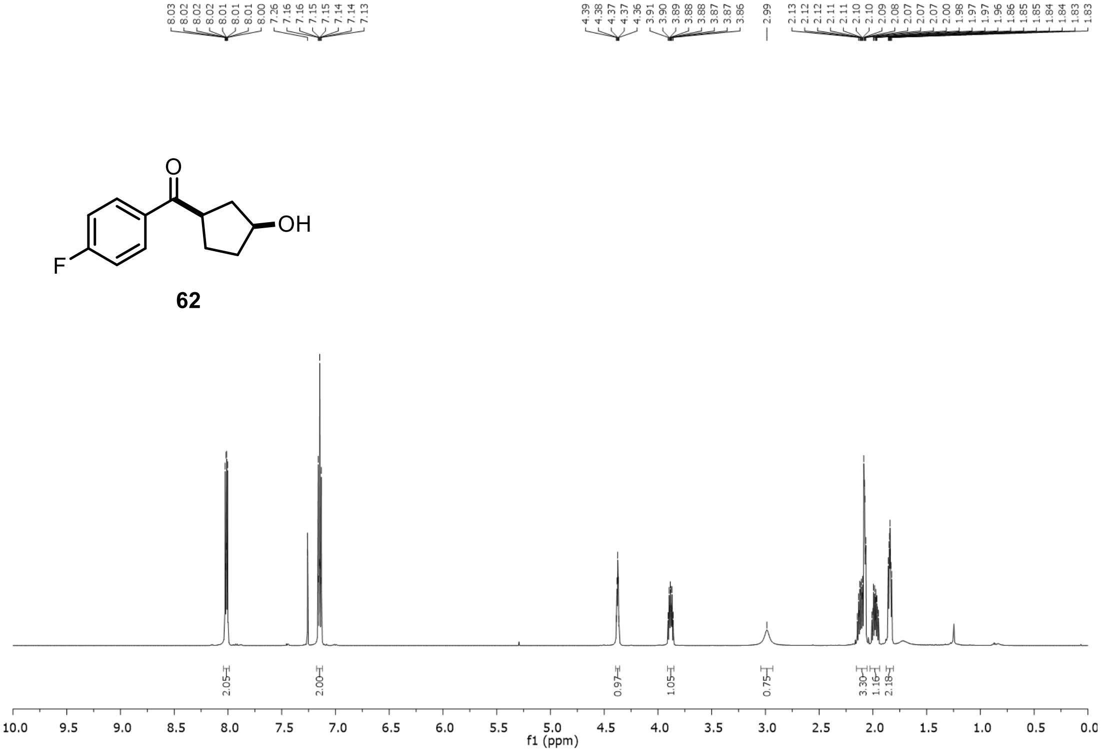

^{13}C NMR (151 MHz, $CDCl_3$)

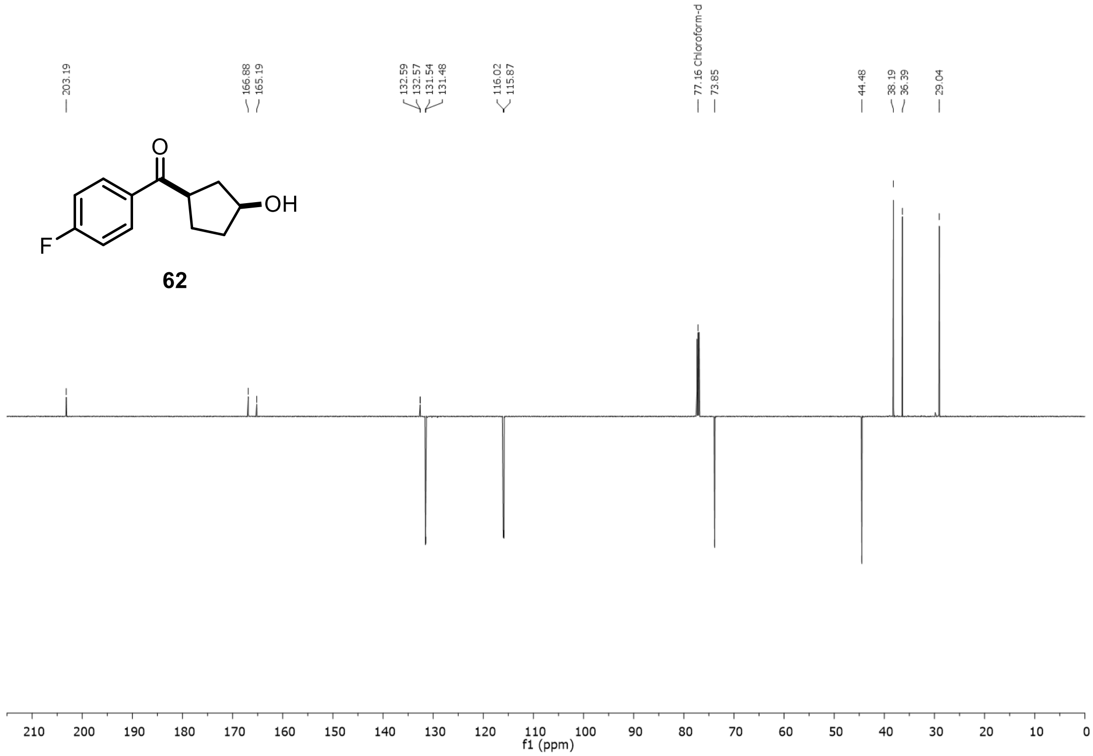

^{19}F NMR (377 MHz, CDCl$_3$)

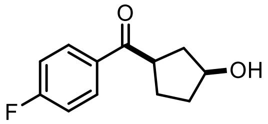

62

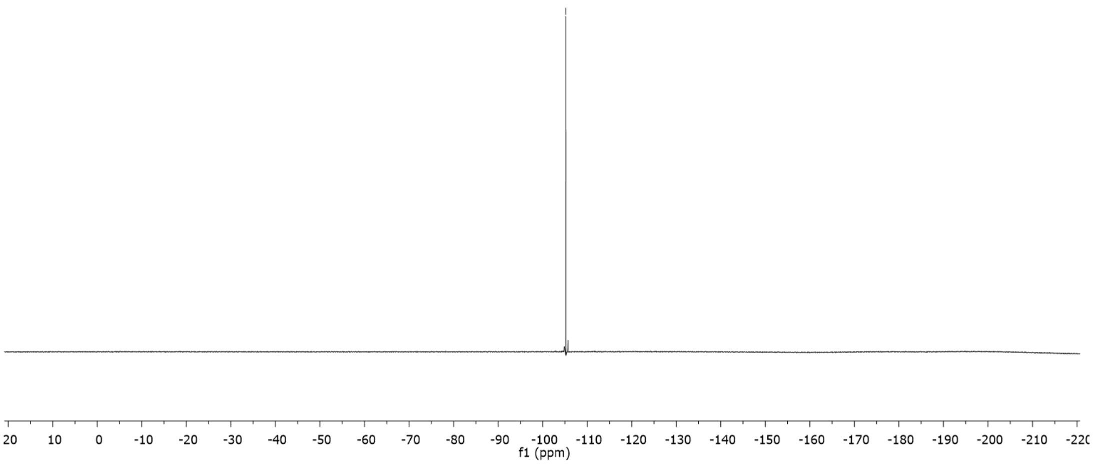

63: *syn*-(3-hydroxycyclohexyl)nonan-1-one

^{1}H NMR (600 MHz, $CDCl_3$)

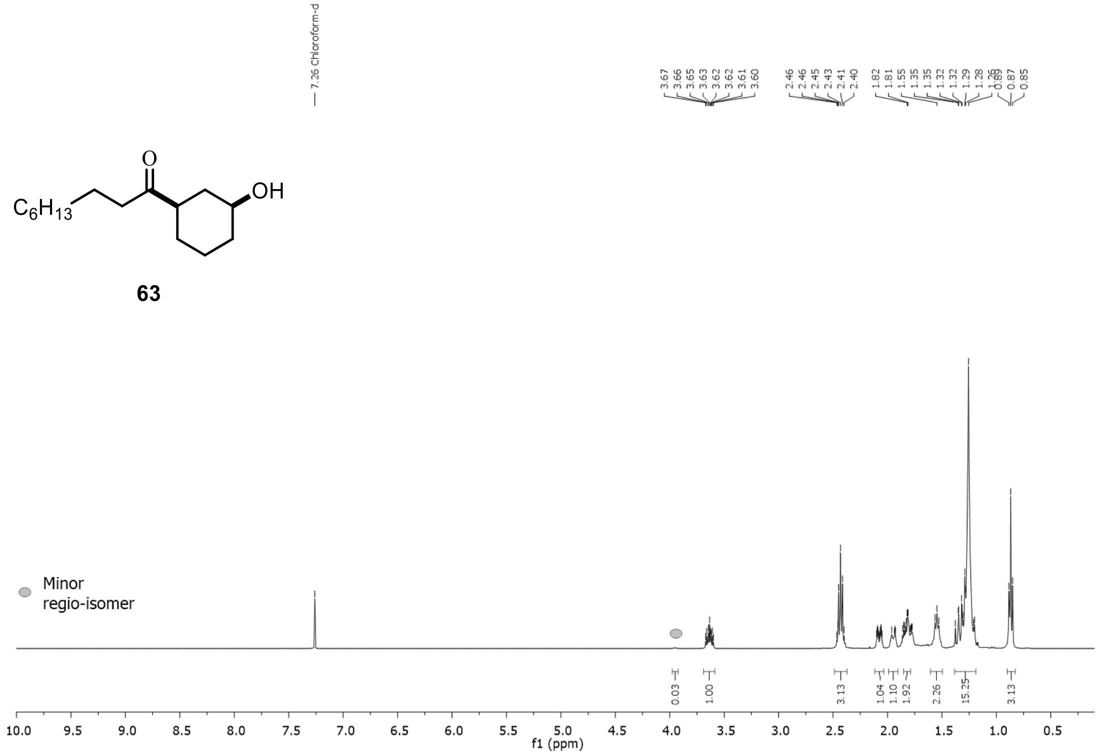

^{13}C NMR (151 MHz, $CDCl_3$)

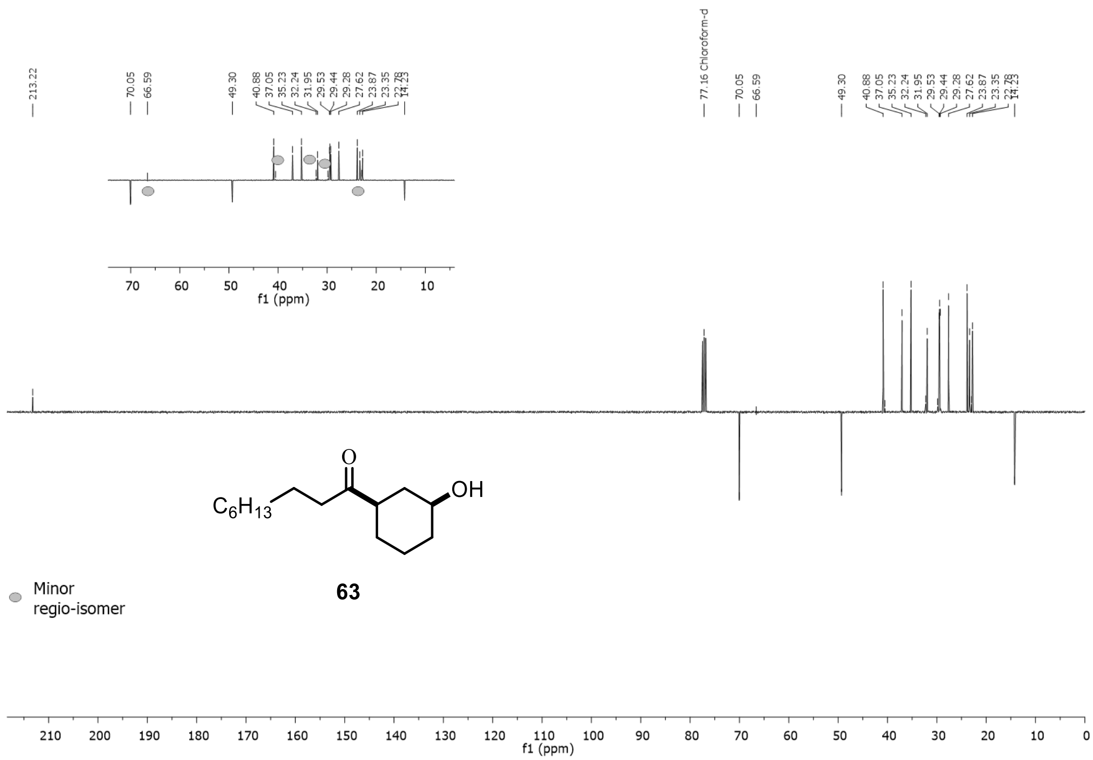

64: *syn*-3-cyclopentyl-1-(-3-hydroxycyclohexyl)propan-1-one

^{1}H NMR (600 MHz, $CDCl_3$)

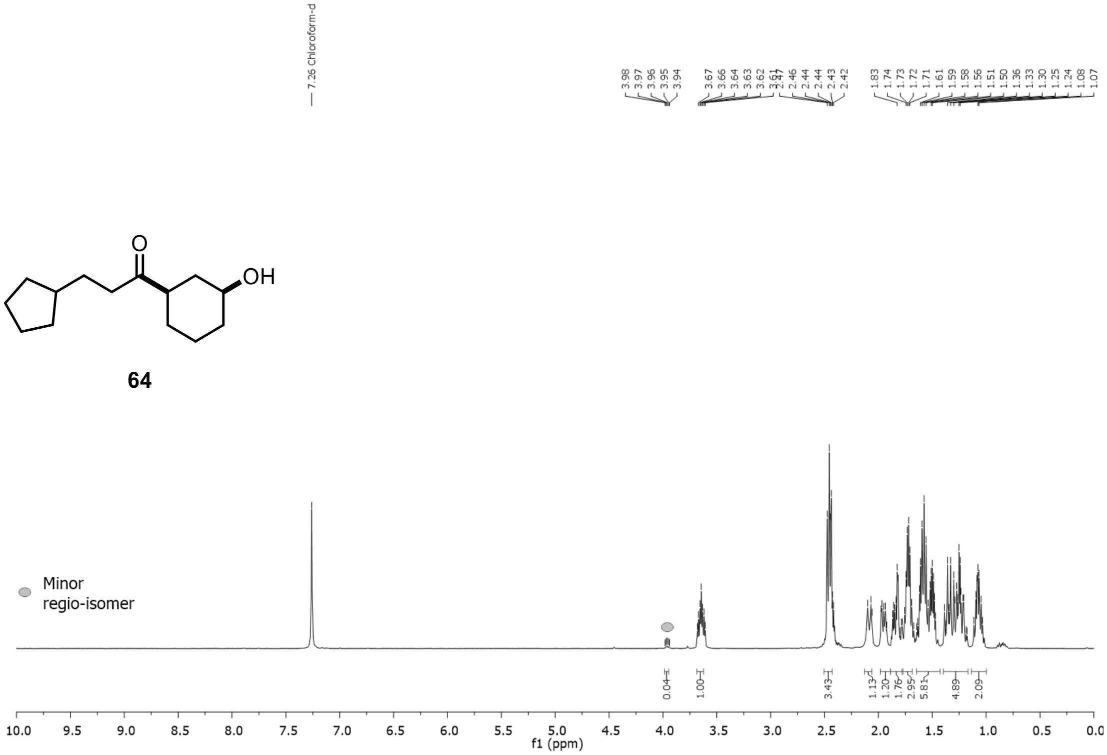

^{13}C NMR (151 MHz, $CDCl_3$)

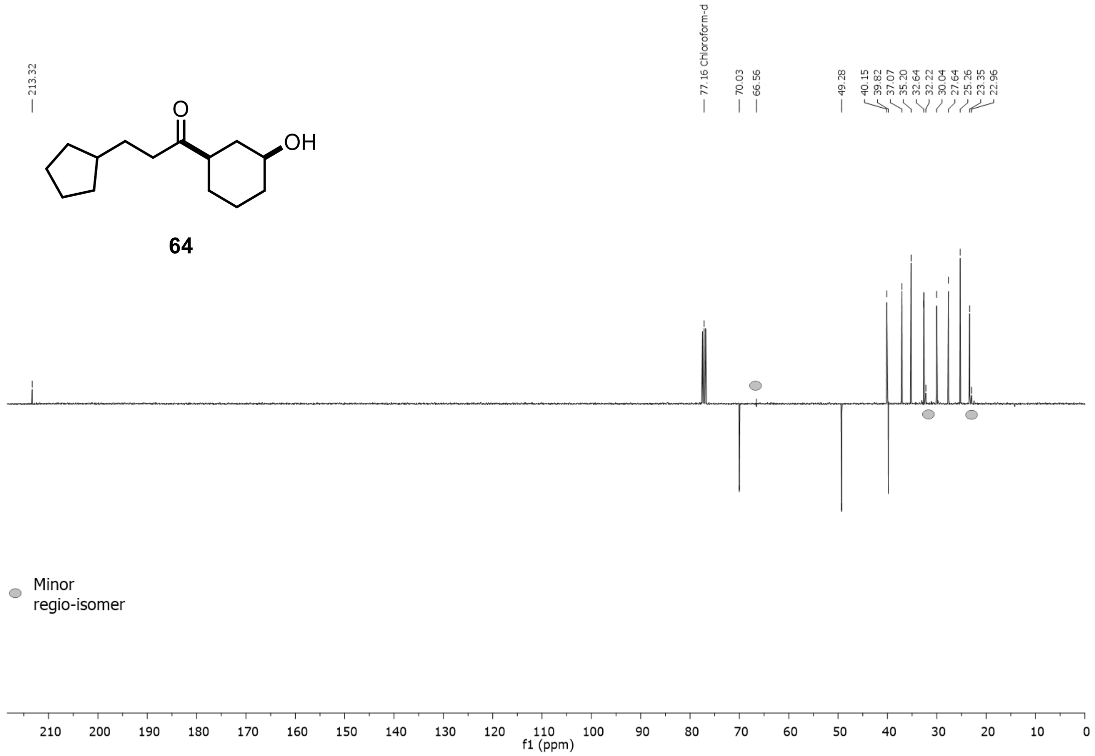

65: *syn*-adamantan-1-yl(-3-hydroxycyclohexyl)methanone

^{1}H NMR (400 MHz, $CDCl_3$)

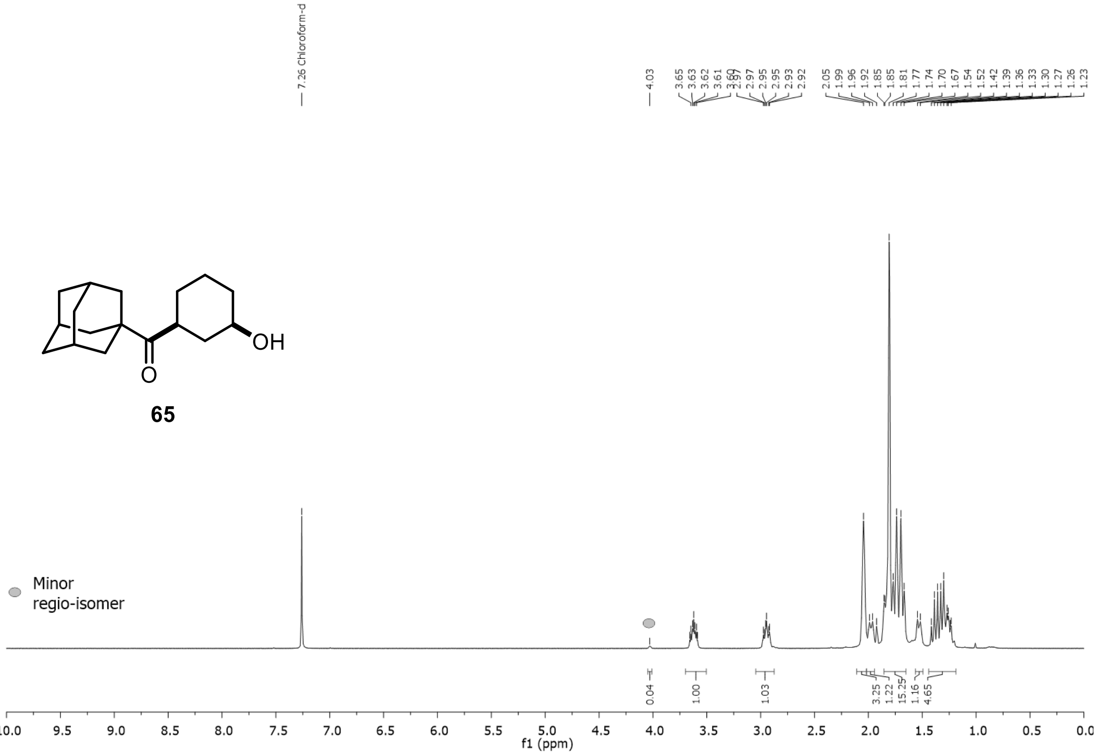

^{13}C NMR (101 MHz, $CDCl_3$)

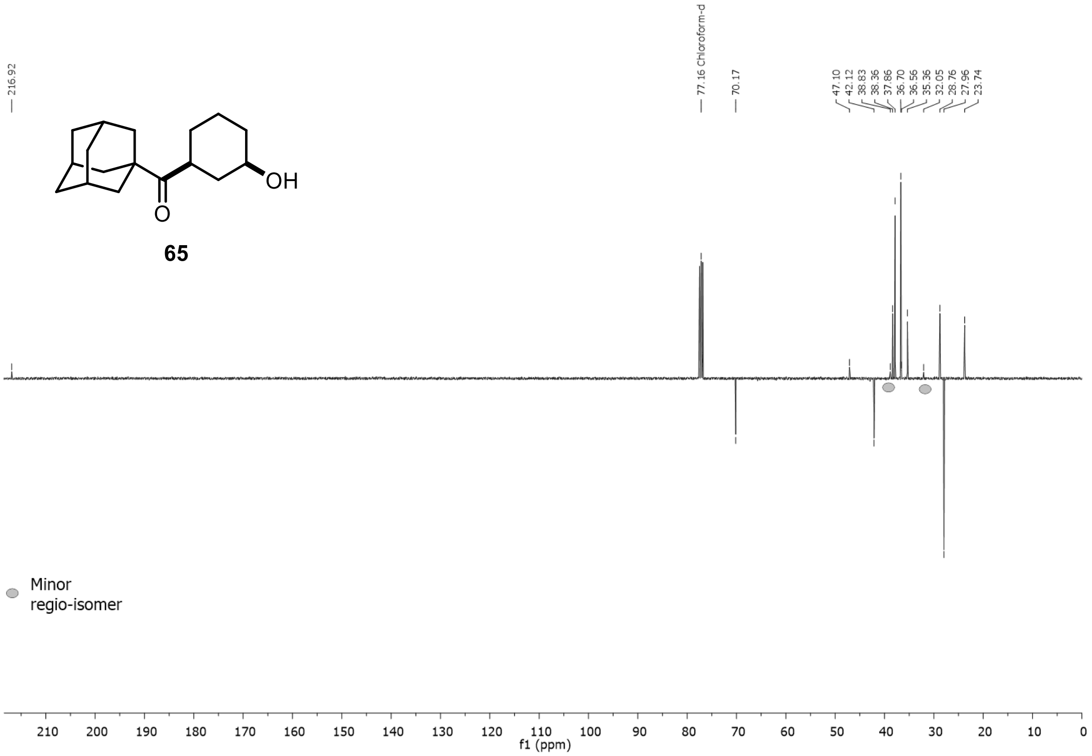

66: *syn*-cyclopropyl(3-hydroxycyclohexyl)methanone

^{1}H NMR (600 MHz, $CDCl_3$)

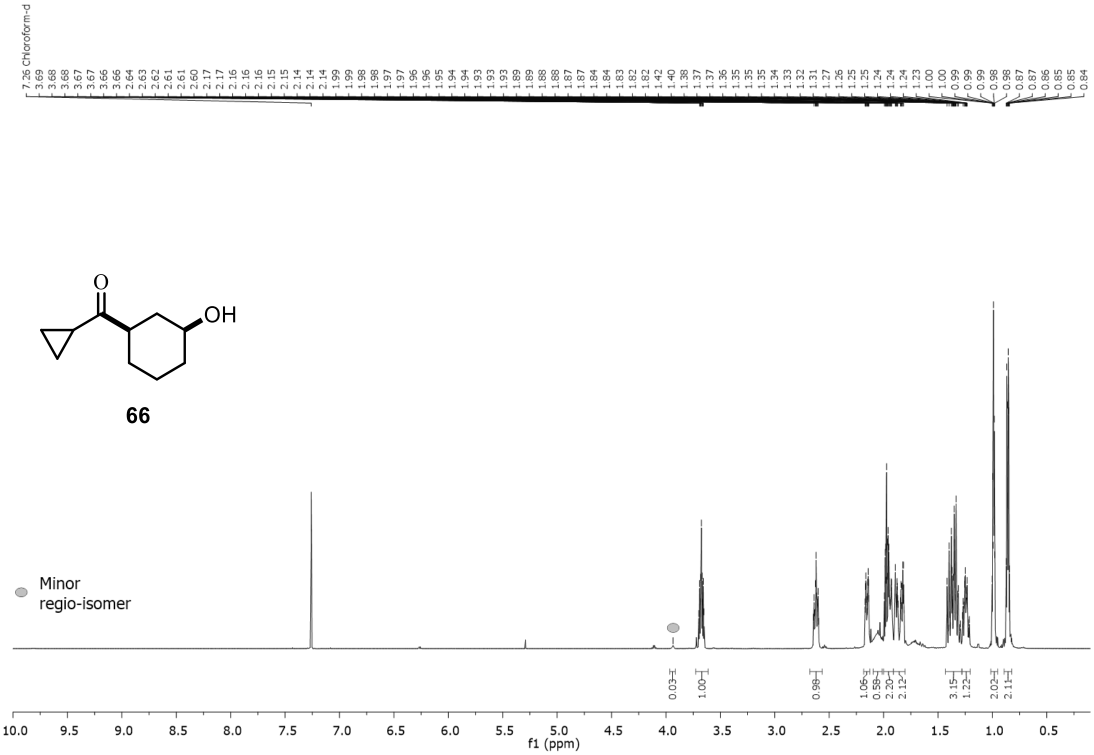

^{13}C NMR (151 MHz, $CDCl_3$)

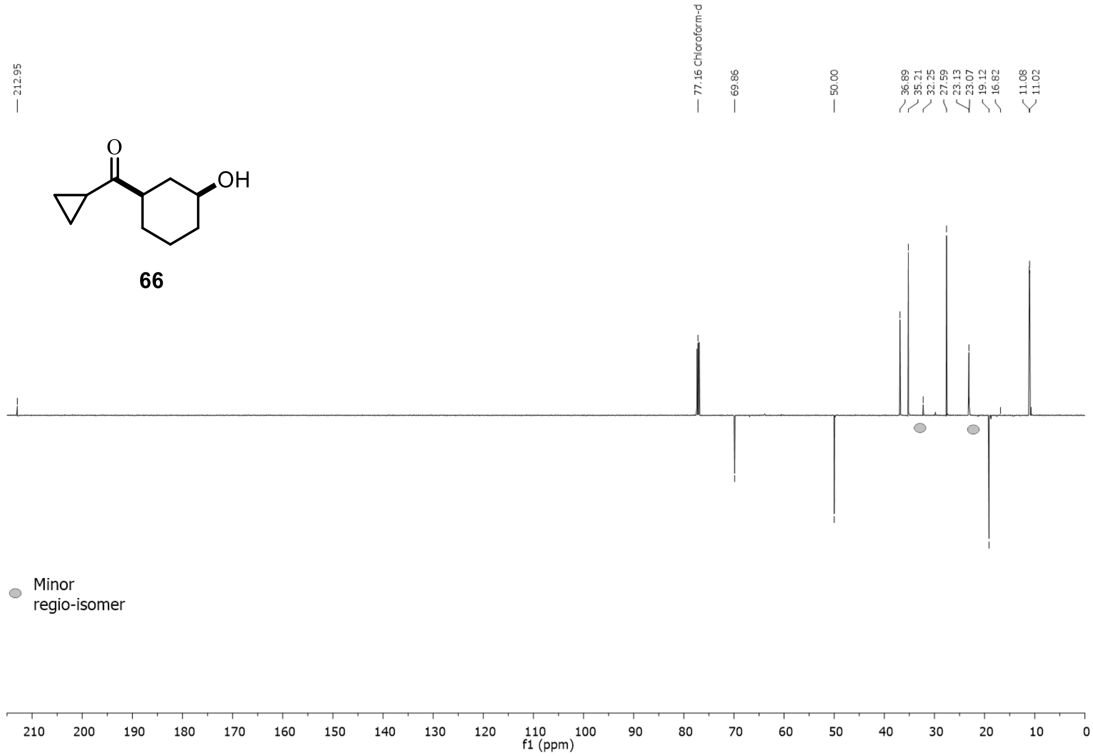

67: *syn*-cyclopropyl(3-hydroxycyclopentyl)methanone

^{1}H NMR (400 MHz, $CDCl_3$)

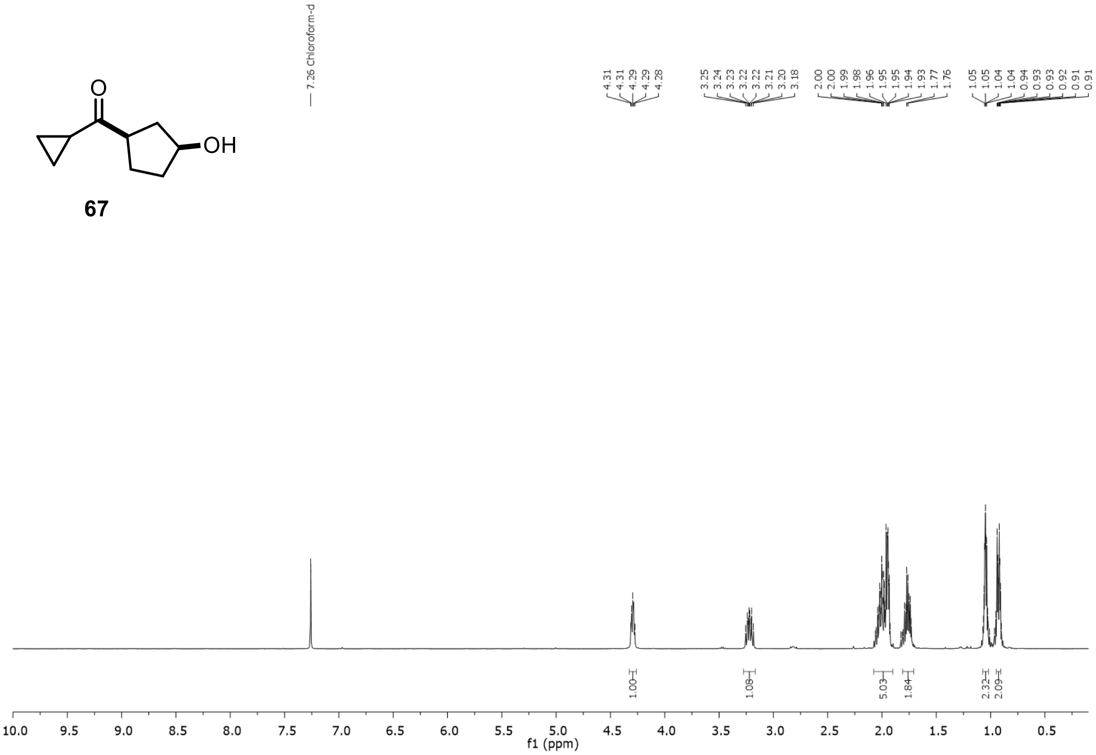

^{13}C NMR (101 MHz, $CDCl_3$)

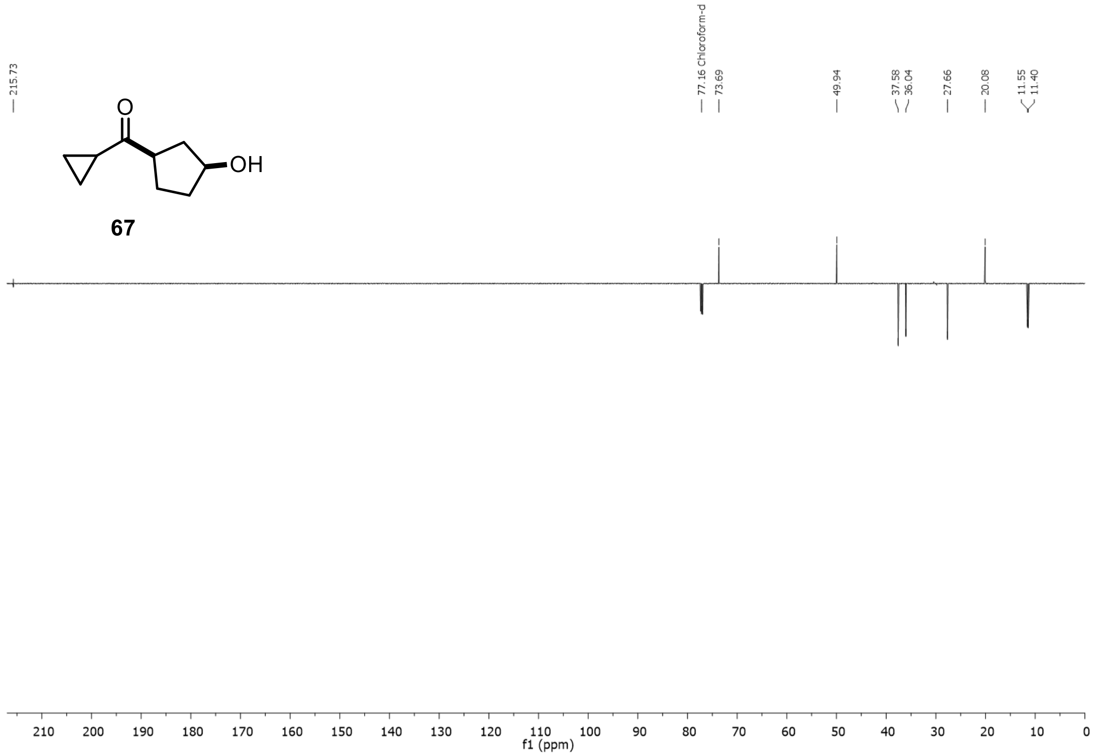

68: *syn*-(3-hydroxycyclopentyl)nonan-1-one

^{1}H NMR (500 MHz, $CDCl_3$)

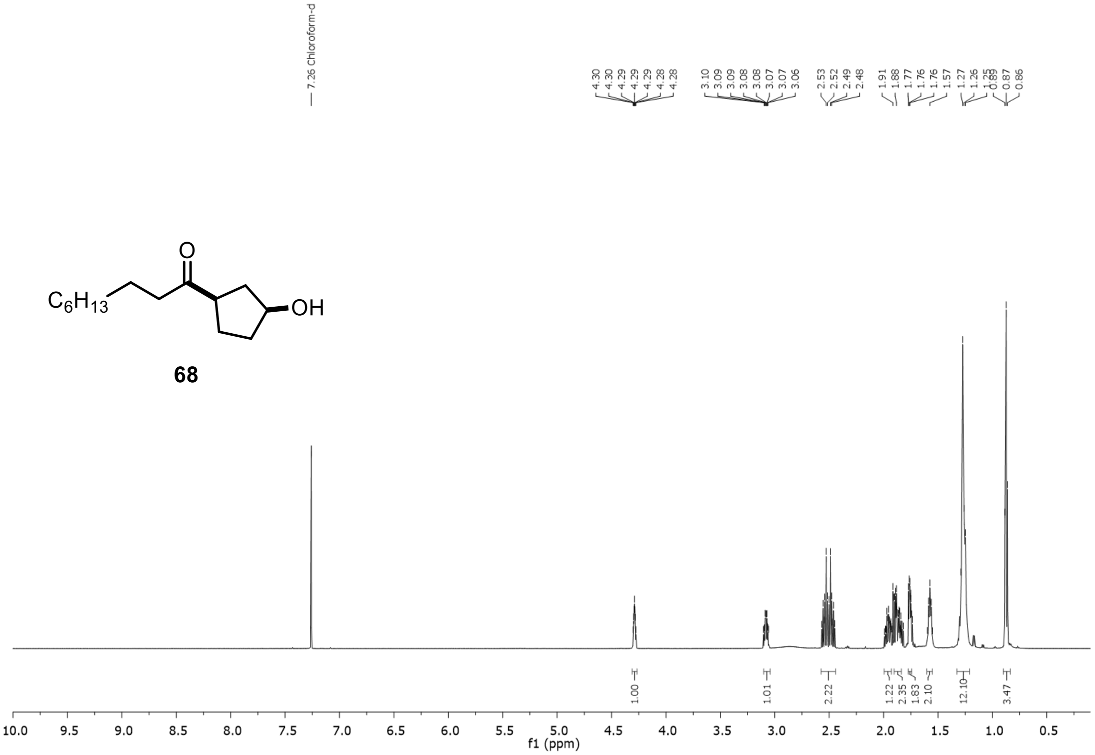

^{13}C NMR (126 MHz, $CDCl_3$)

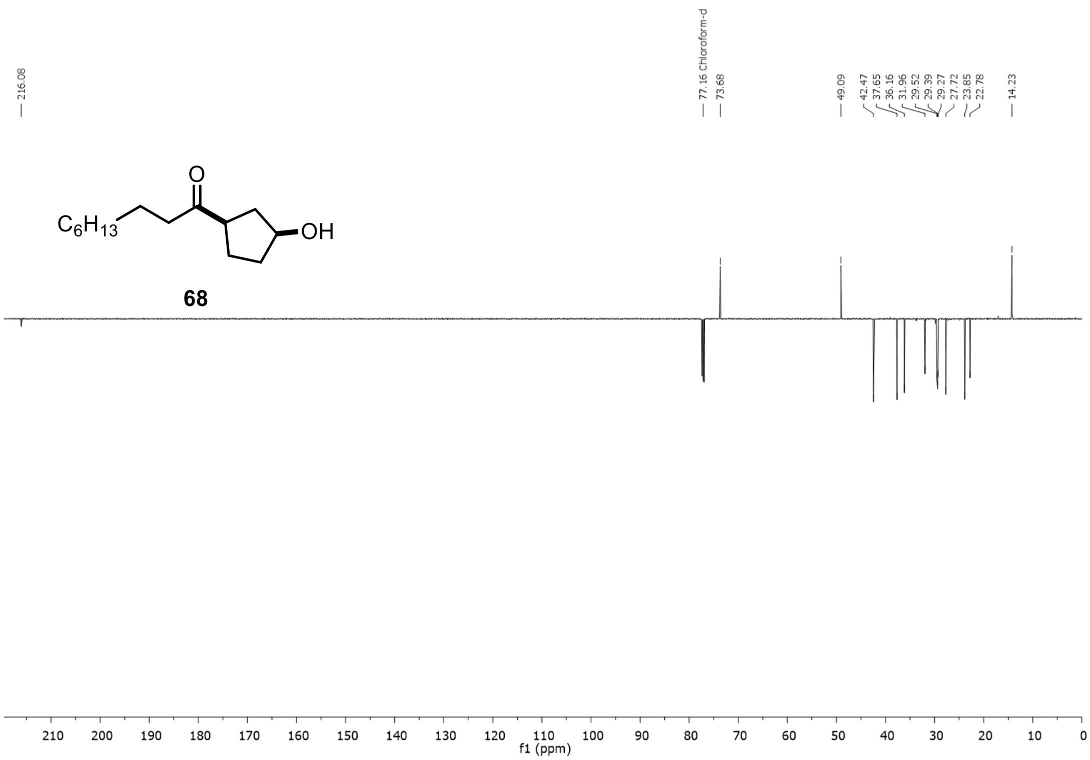

69: *syn*-1-(3-hydroxycyclohexyl)-3-phenylpropan-1-one

^{1}H NMR (400 MHz, $CDCl_3$)

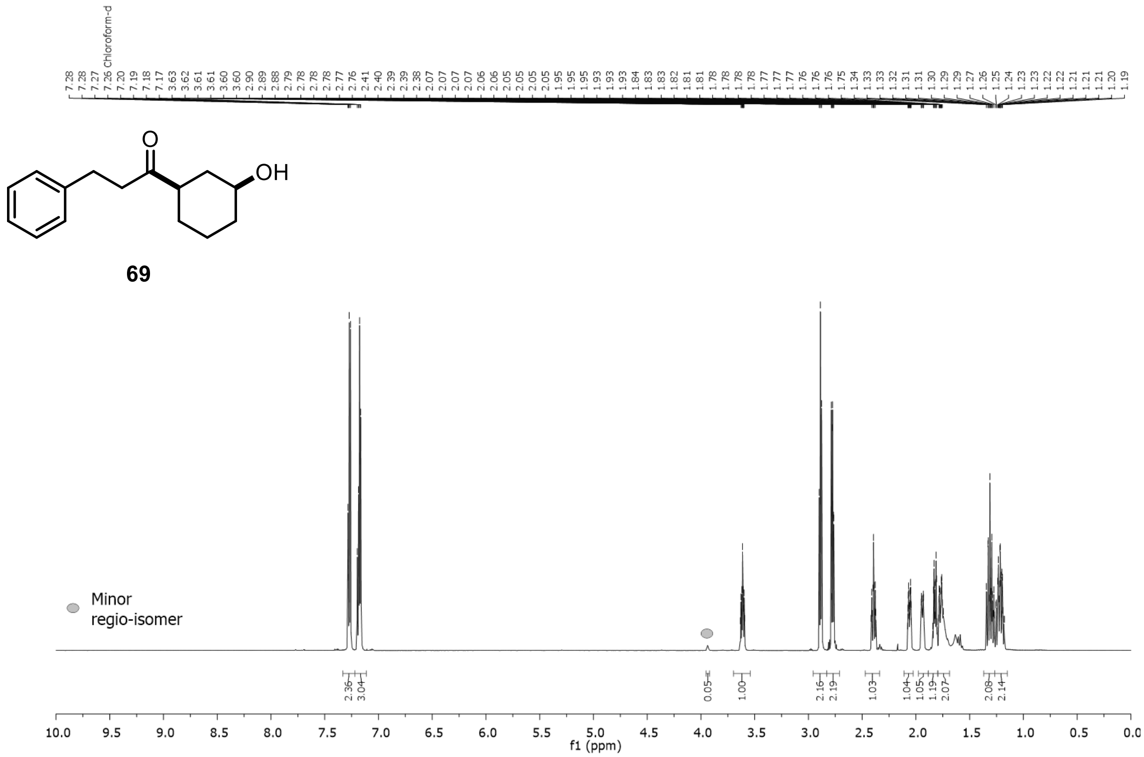

^{13}C NMR (101 MHz, $CDCl_3$)

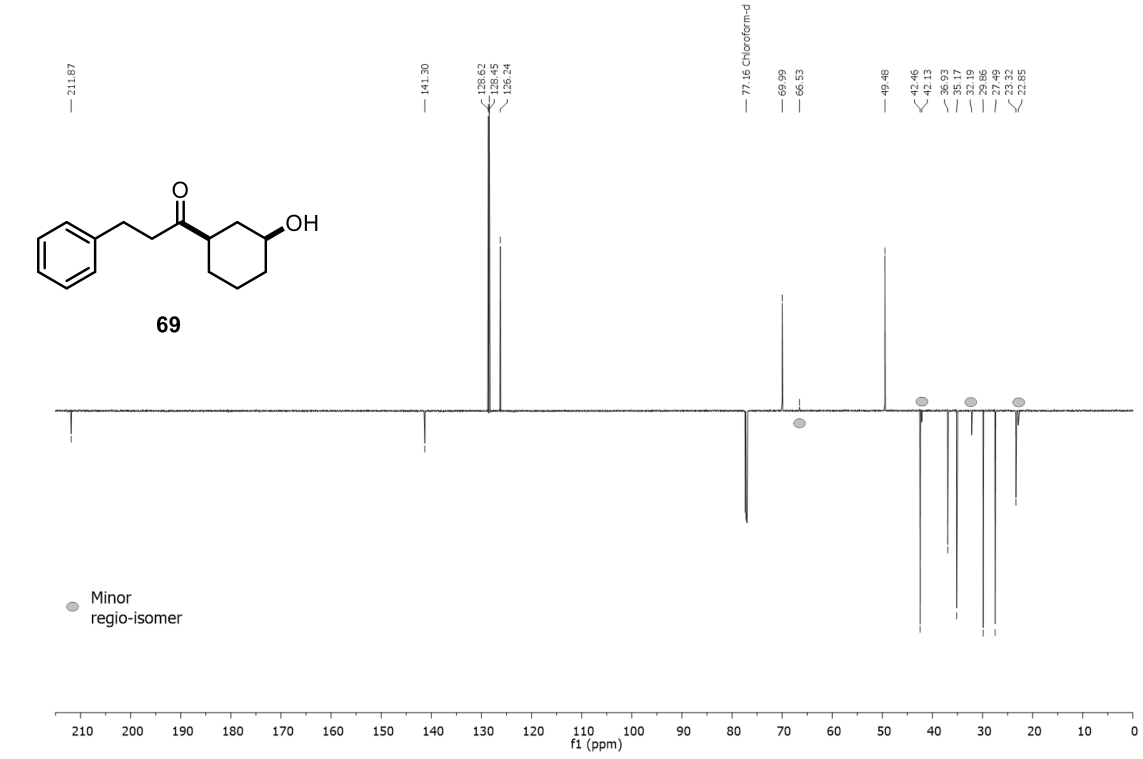

70: *syn*-(3-hydroxycyclohexyl)-3-(4-(trifluoromethyl)phenyl)propan-1-one

^{1}H NMR (400 MHz, $CDCl_3$)

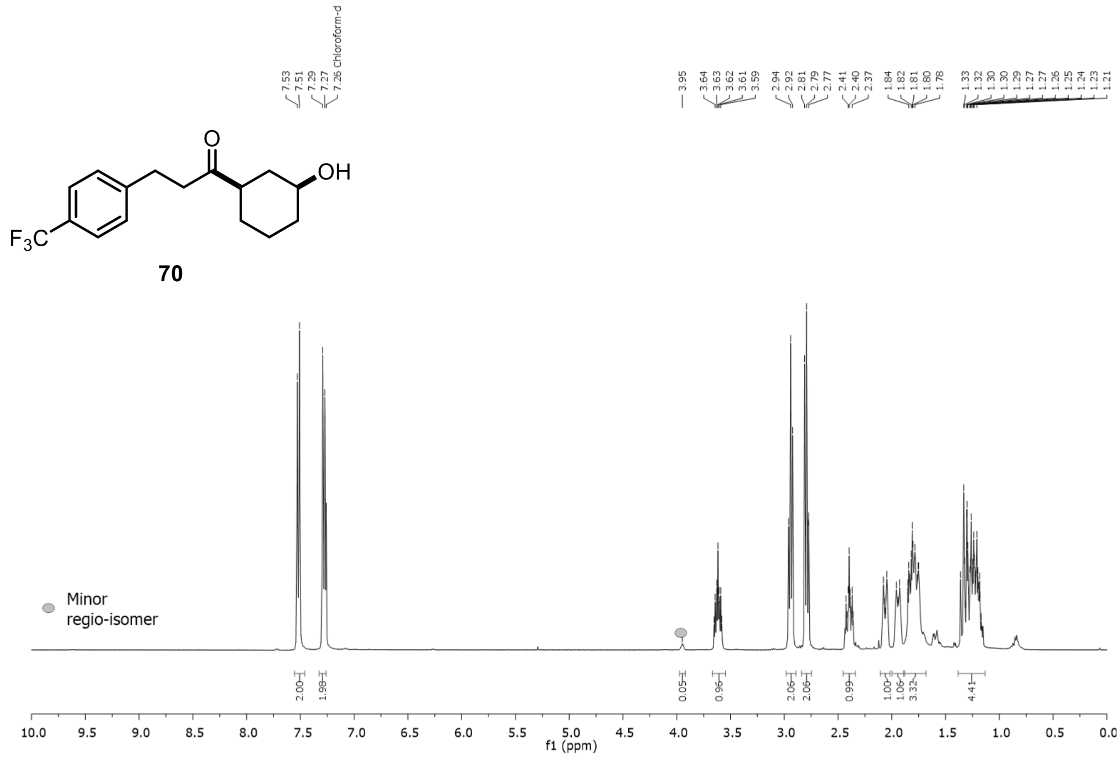

^{13}C NMR (101 MHz, $CDCl_3$)

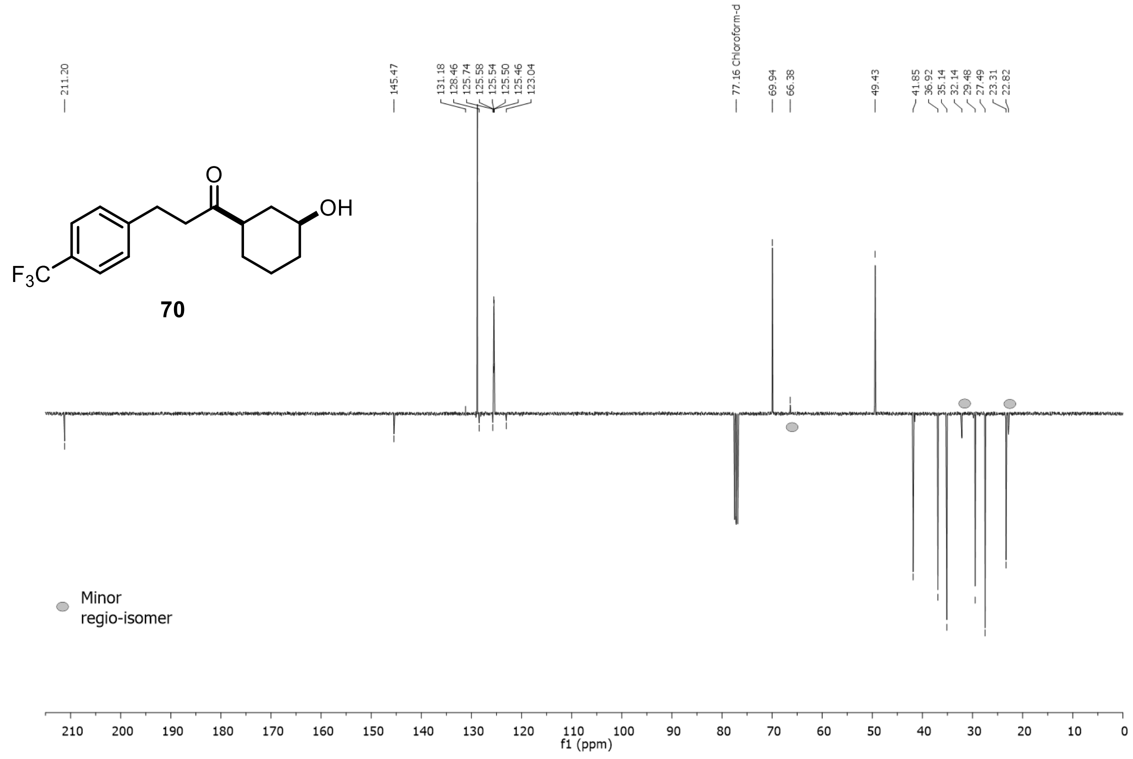

^{19}F NMR (377 MHz, $CDCl_3$)

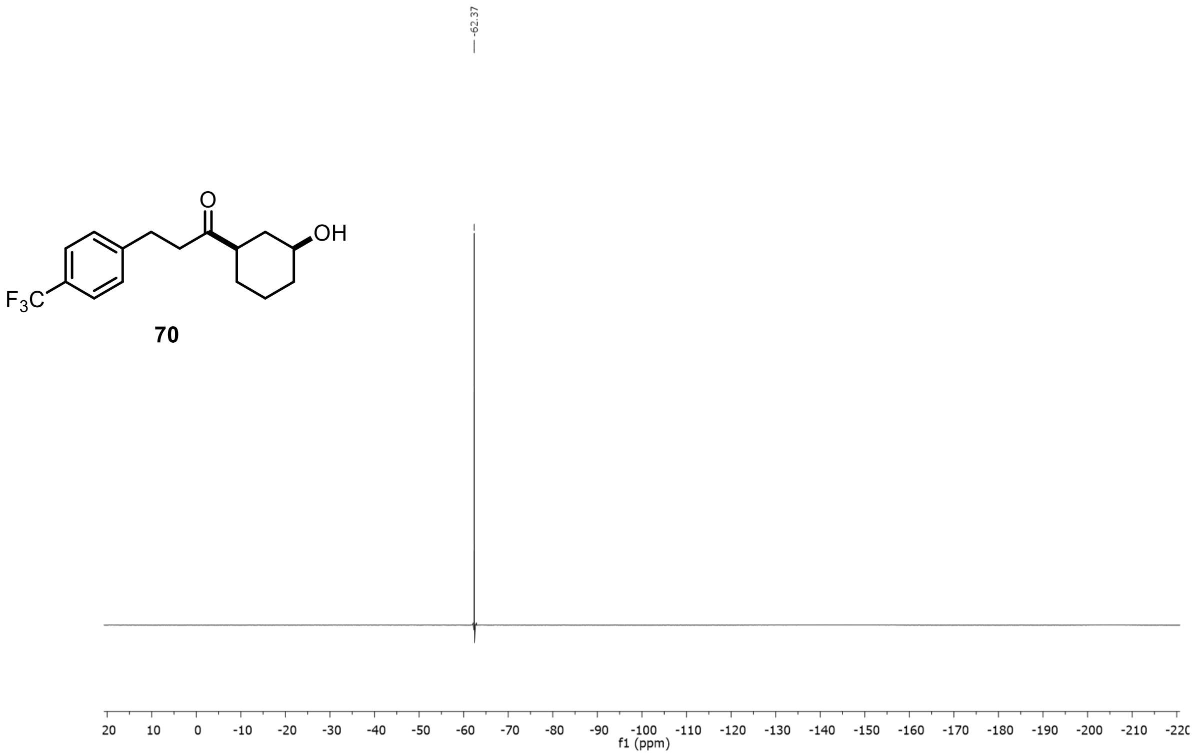

71: *syn*-furan-2-yl(-3-hydroxycyclohexyl)methanone

^{1}H NMR (600 MHz, $CDCl_3$)

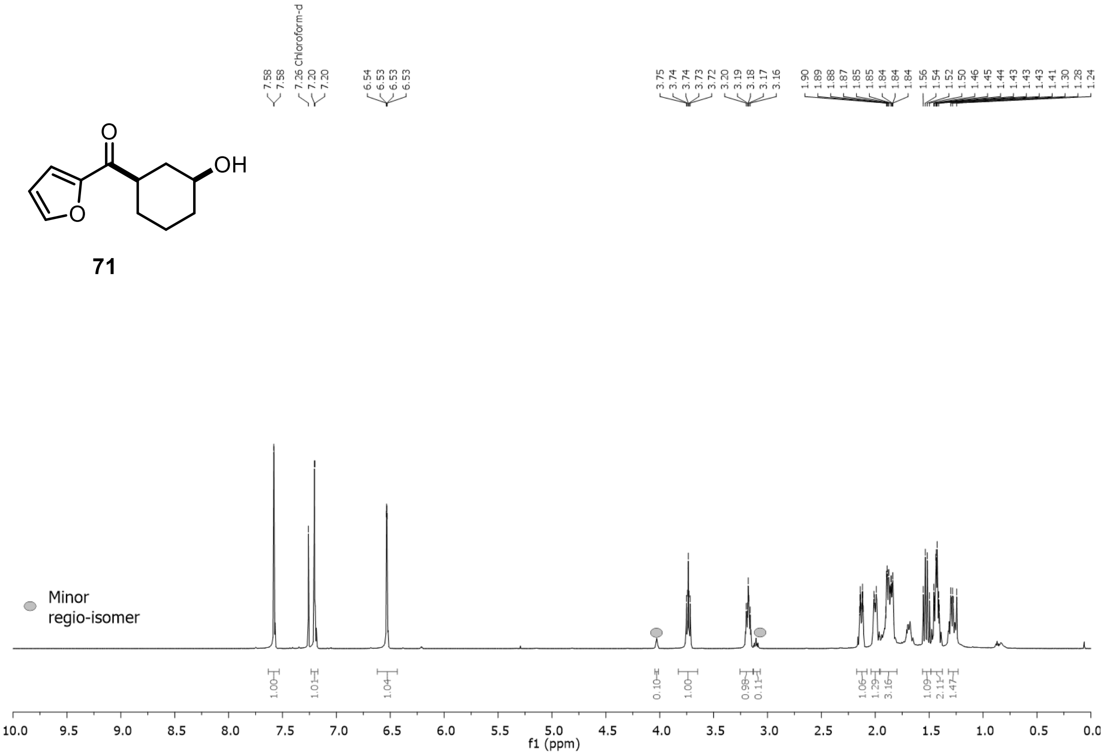

^{13}C NMR (151 MHz, $CDCl_3$)

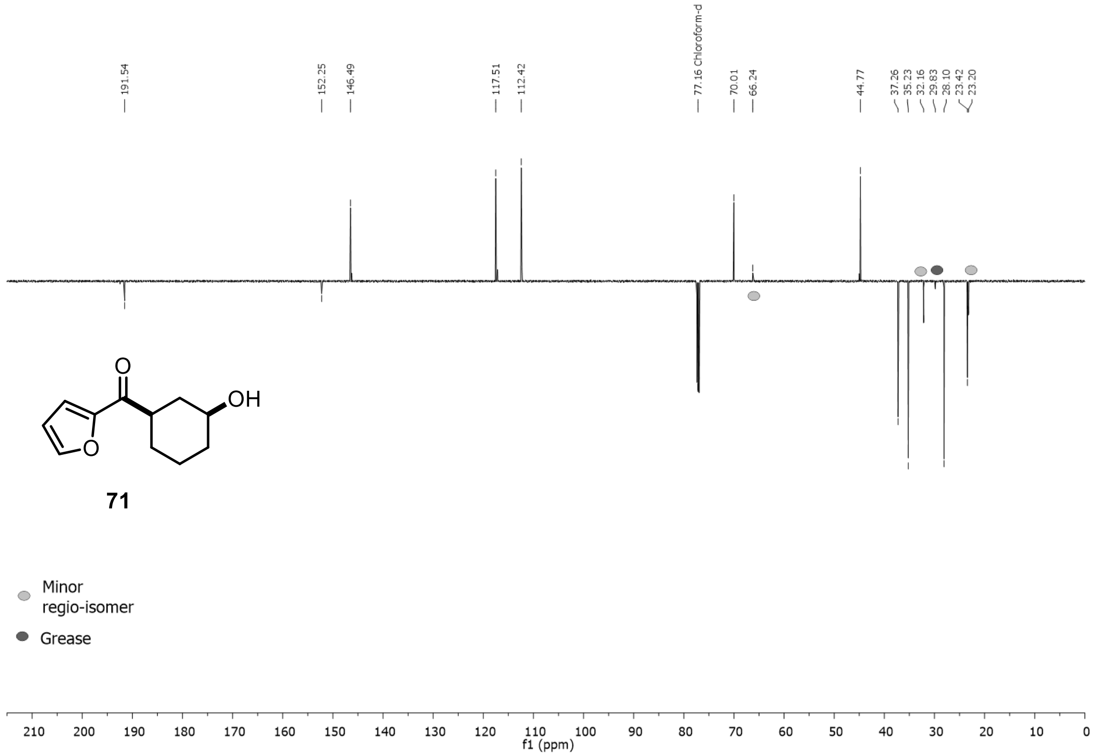

72: *syn*-(3-hydroxycyclohexyl)(thiophen-2-yl)methanone

^{1}H NMR (400 MHz, $CDCl_3$)

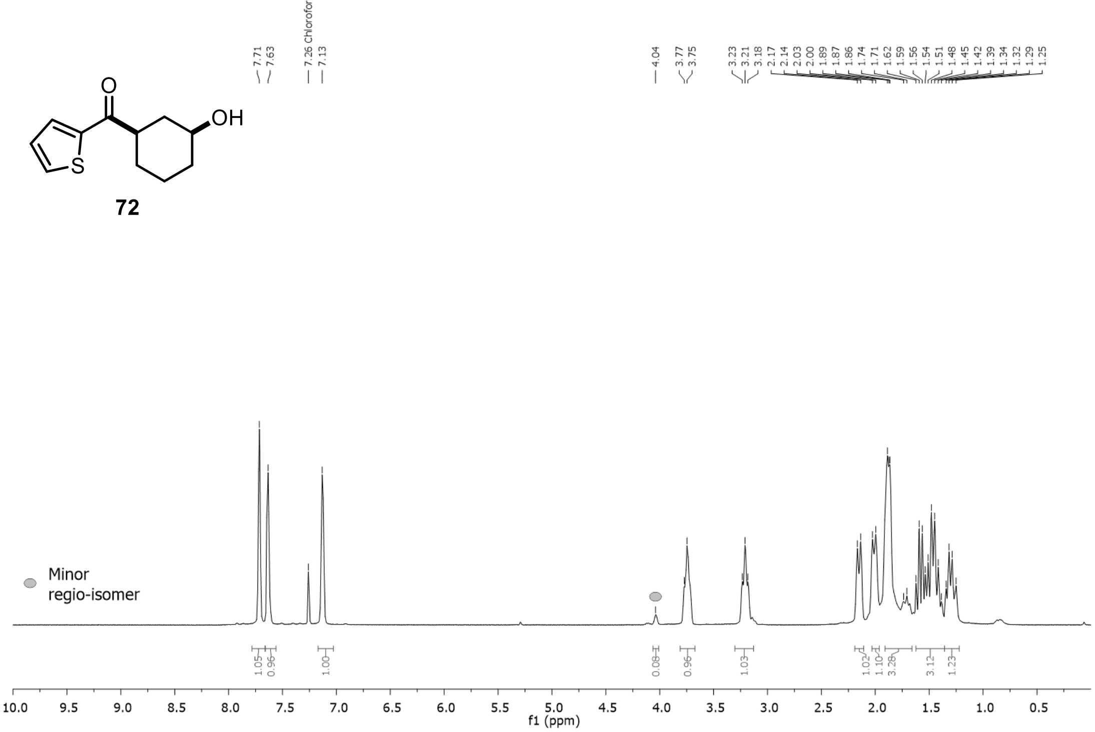

^{13}C NMR (101 MHz, $CDCl_3$)

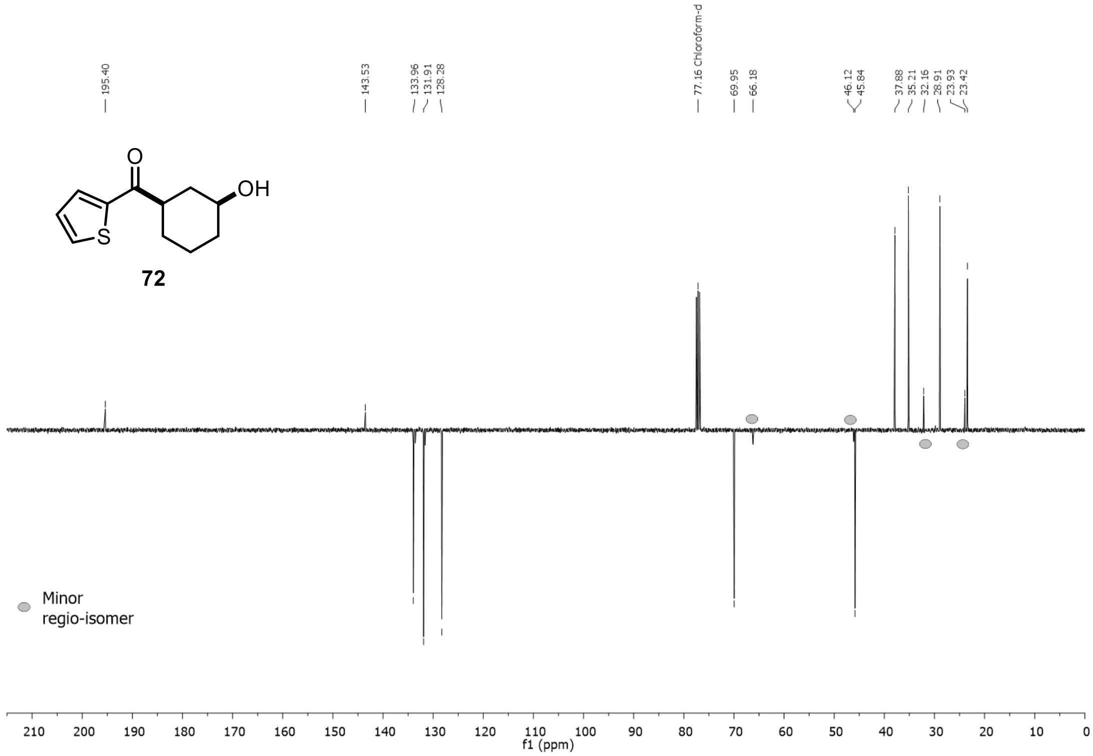

73: *syn*-(3-hydroxycyclopentyl)(thiophen-2-yl)methanone

^{1}H NMR (400 MHz, $CDCl_3$)

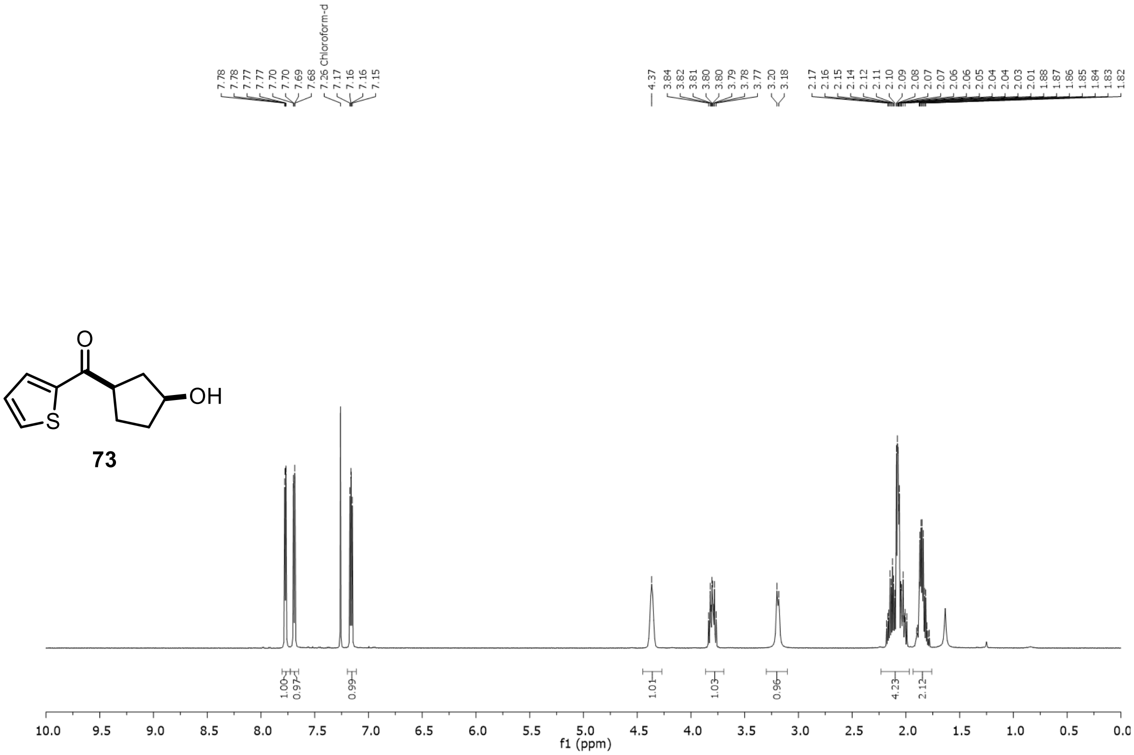

^{13}C NMR (101 MHz, $CDCl_3$)

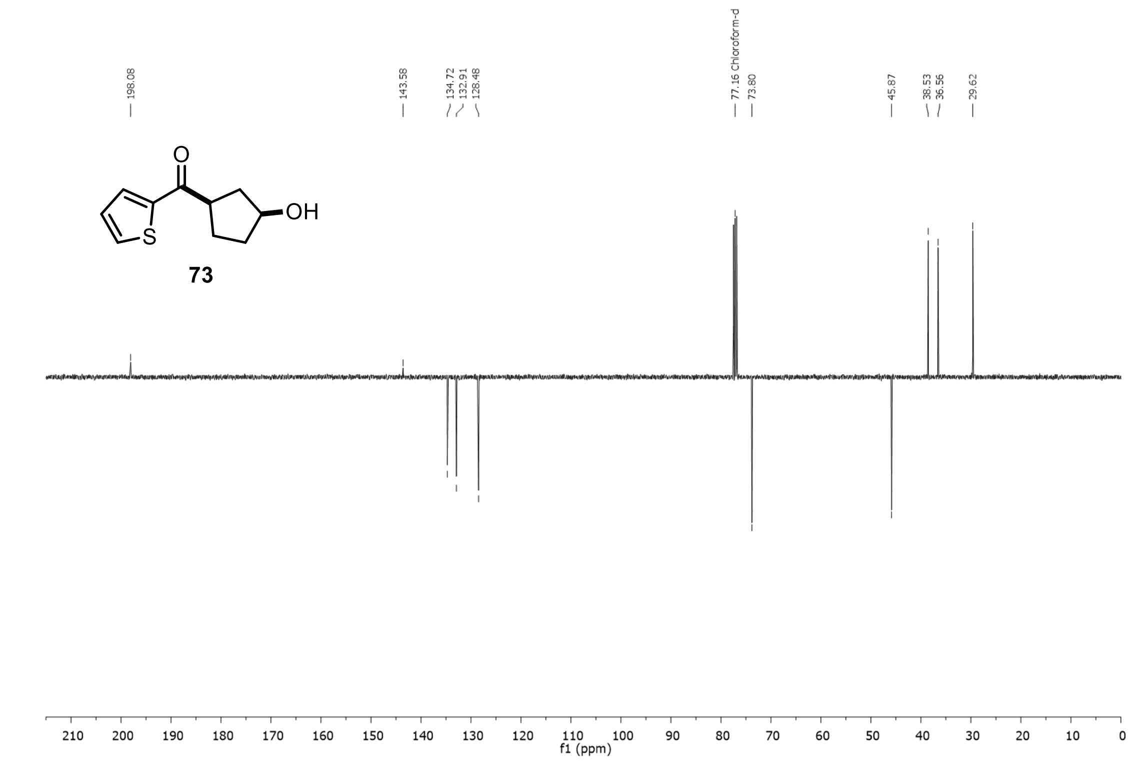

74: *syn*-(3-hydroxycyclododecyl)(phenyl)methanone

^{1}H NMR (600 MHz, $CDCl_3$)

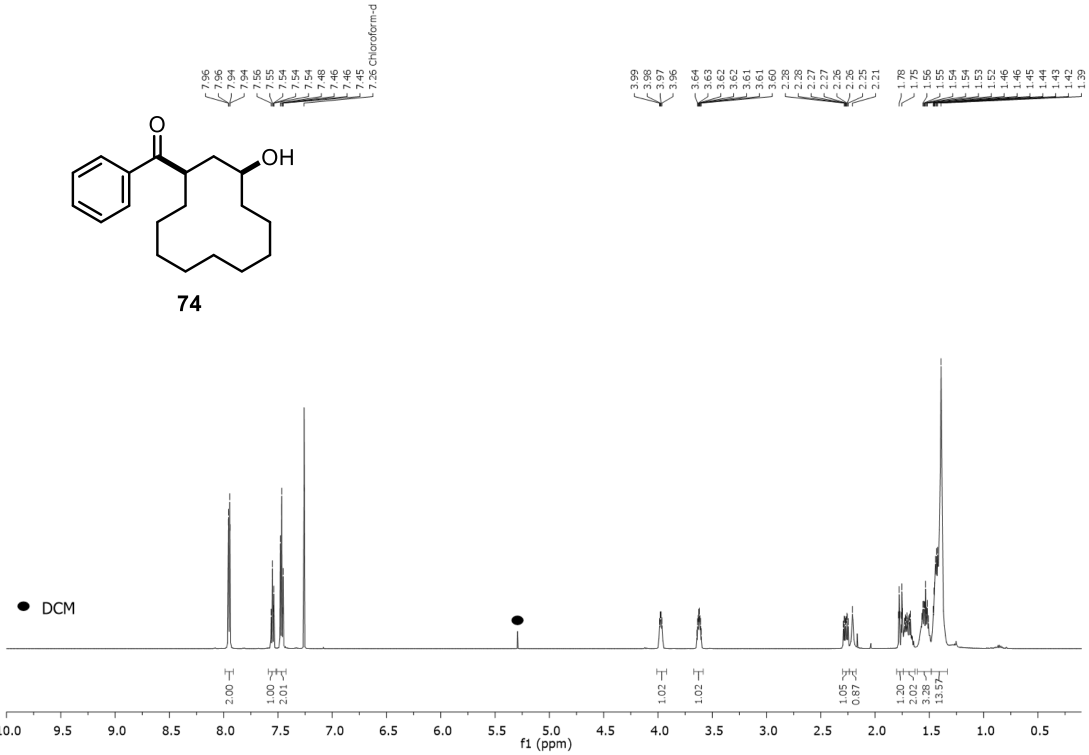

^{13}C NMR (151 MHz, $CDCl_3$)

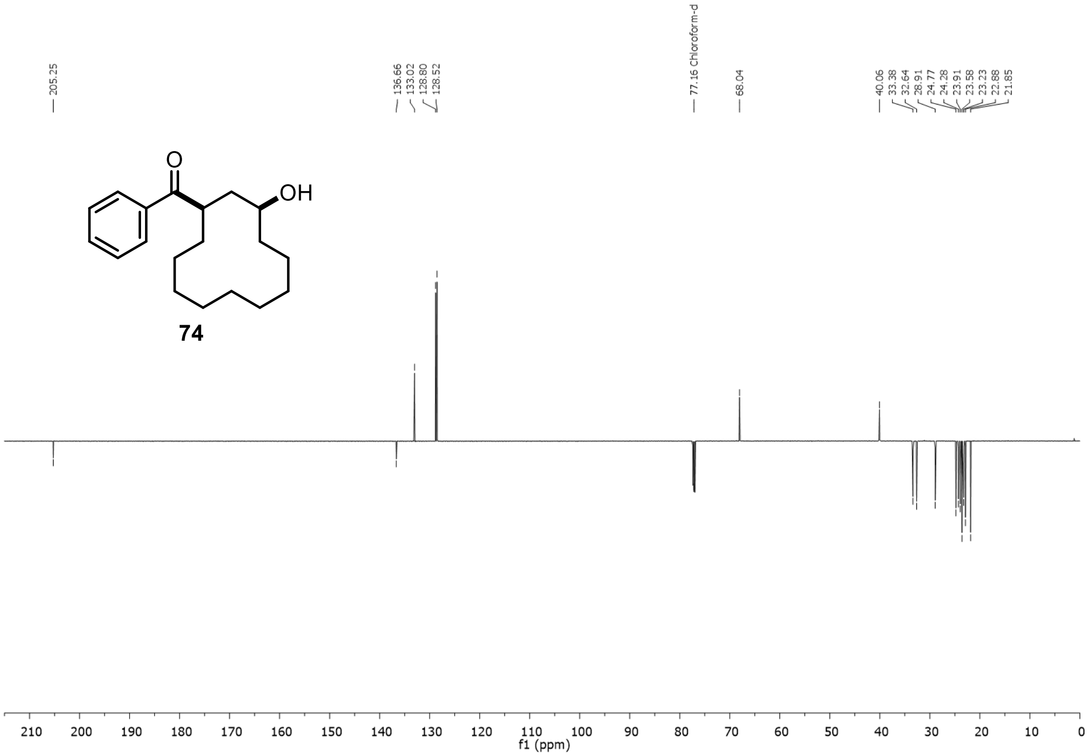

75: *syn*-(3-hydroxycyclohexyl)(*m*-tolyl)methanone

^{1}H NMR (500 MHz, CDCl$_3$)

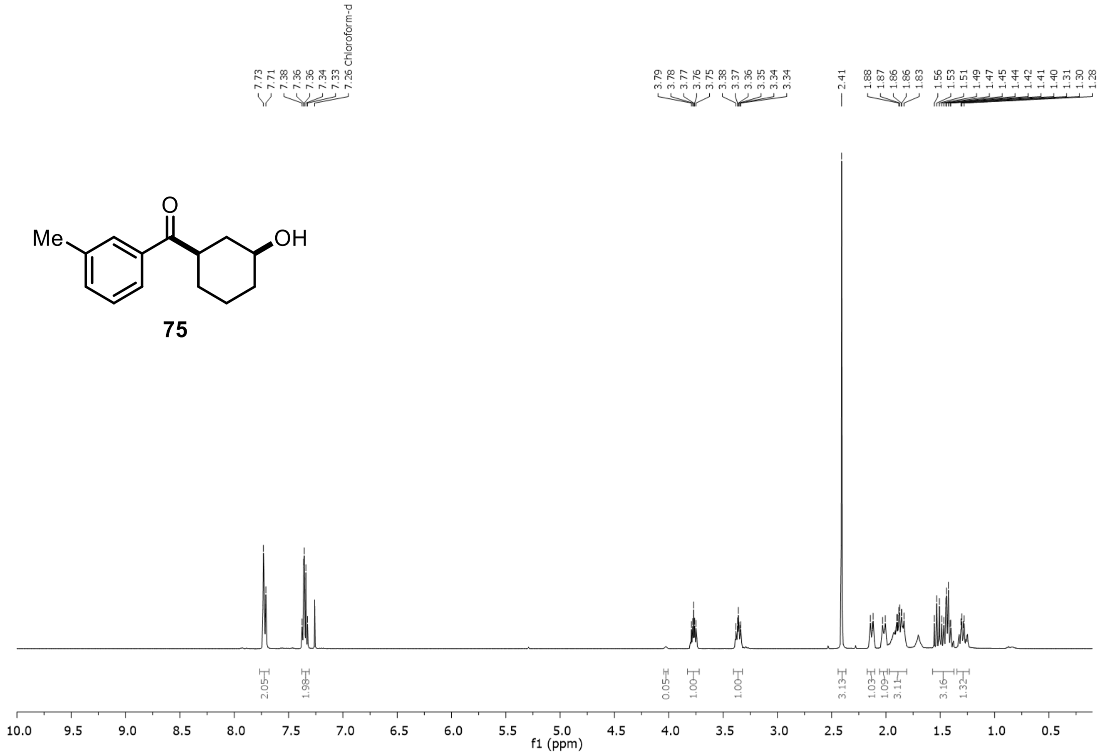

^{13}C NMR (126 MHz, CDCl$_3$)

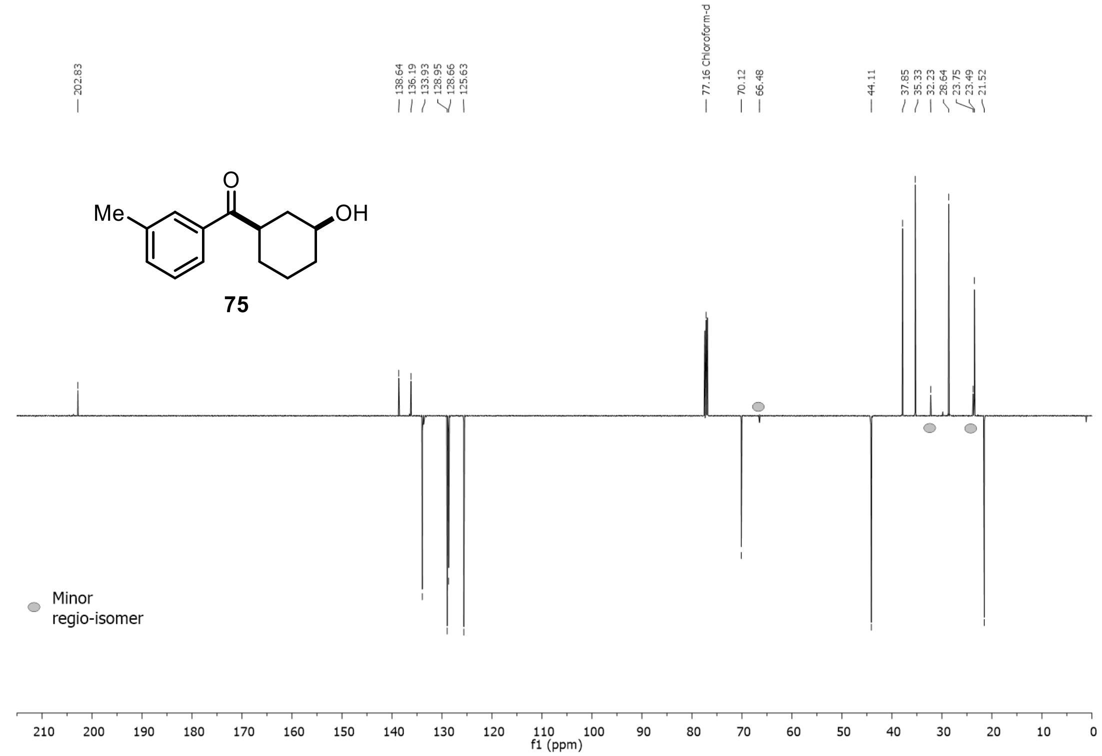

76: *syn*-3-hydroxycyclohexyl(2-bromophenyl)methanone

^{1}H NMR (600 MHz, $CDCl_3$)

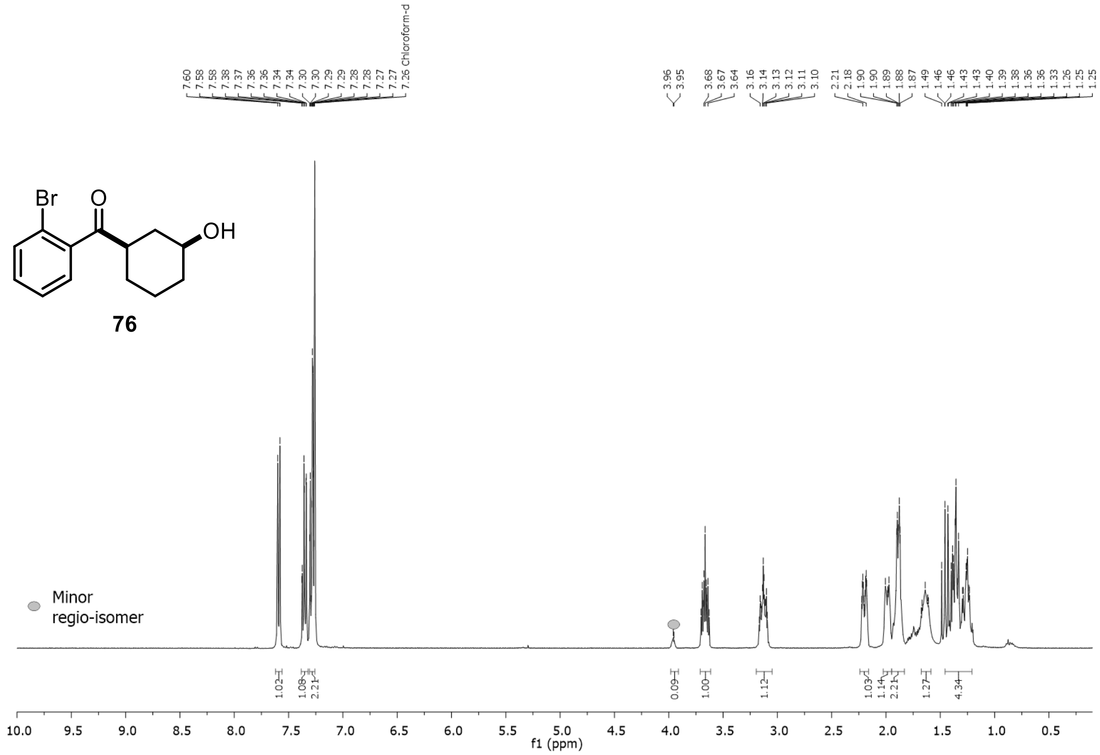

^{13}C NMR (151 MHz, $CDCl_3$)

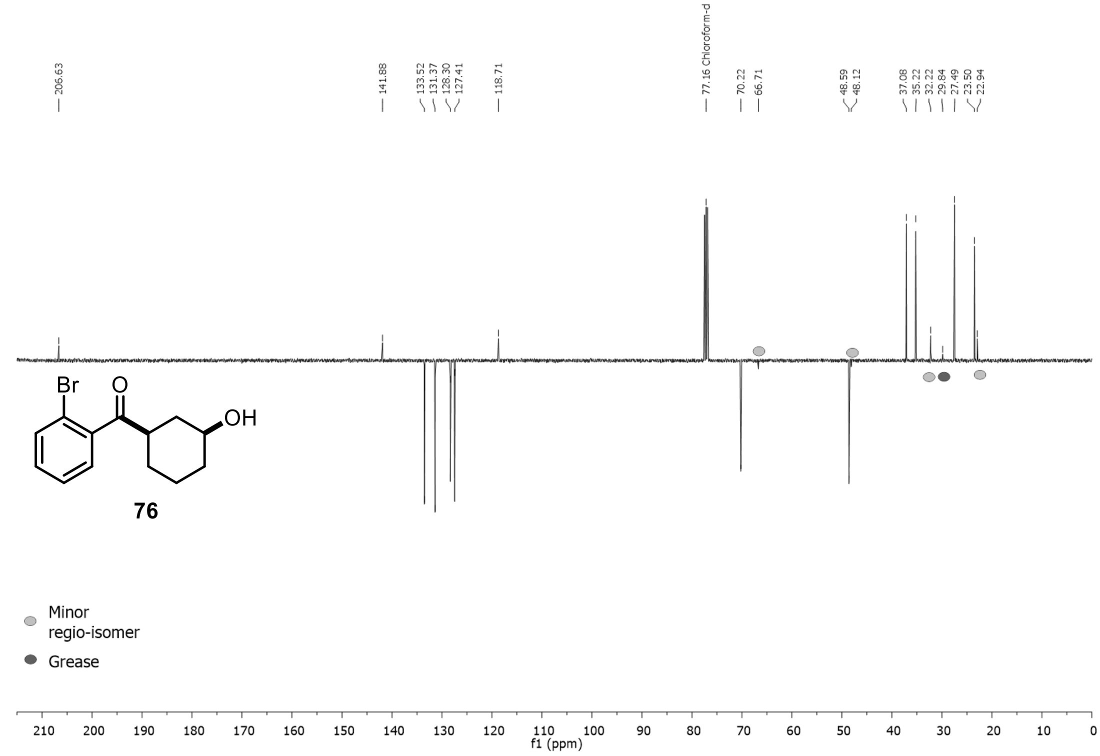

77: *syn*-(3-hydroxycyclohexyl)(*p*-tolyl)methanone

^{1}H NMR (400 MHz, $CDCl_3$)

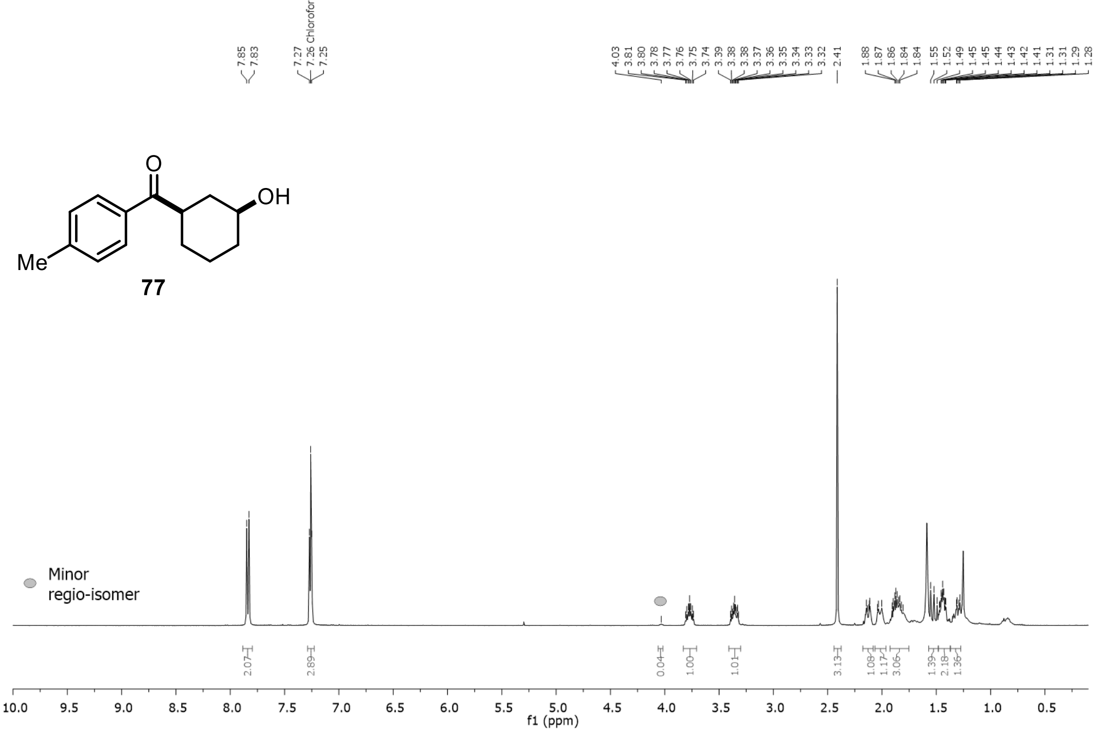

^{13}C NMR (101 MHz, $CDCl_3$)

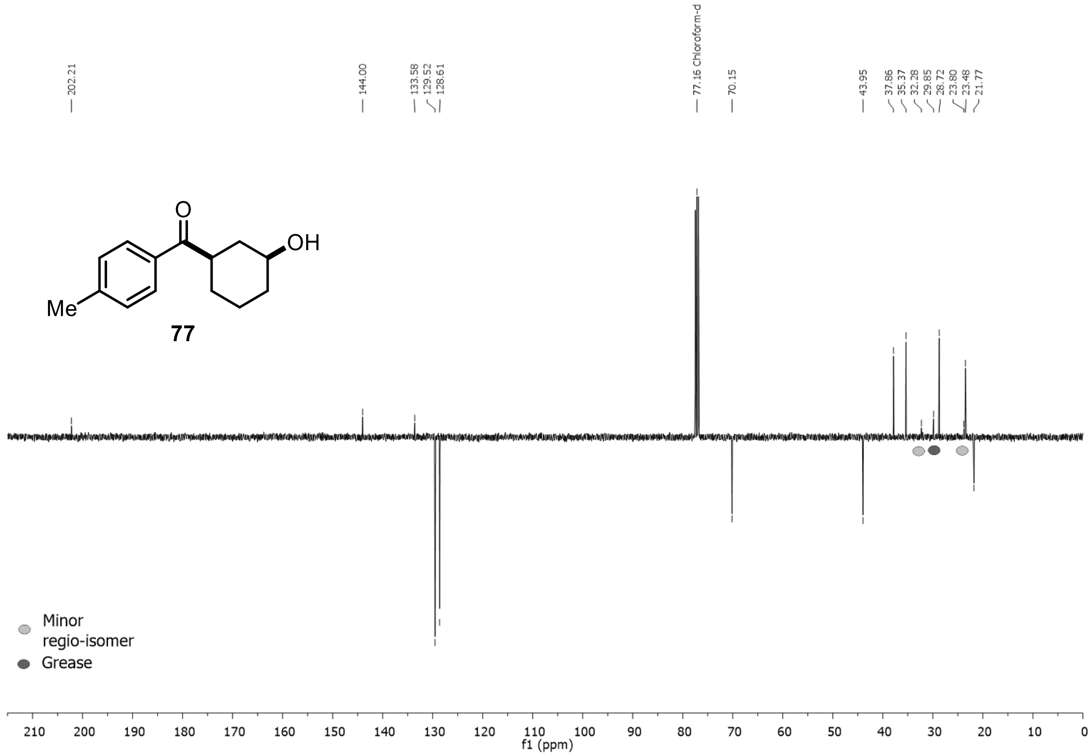

78: *rac*-(2-hydroxy-7,7-dimethylbicyclo[2.2.1]heptan-1-yl)-1-phenylethan-1-one

^{1}H NMR (400 MHz, CDCl$_3$)

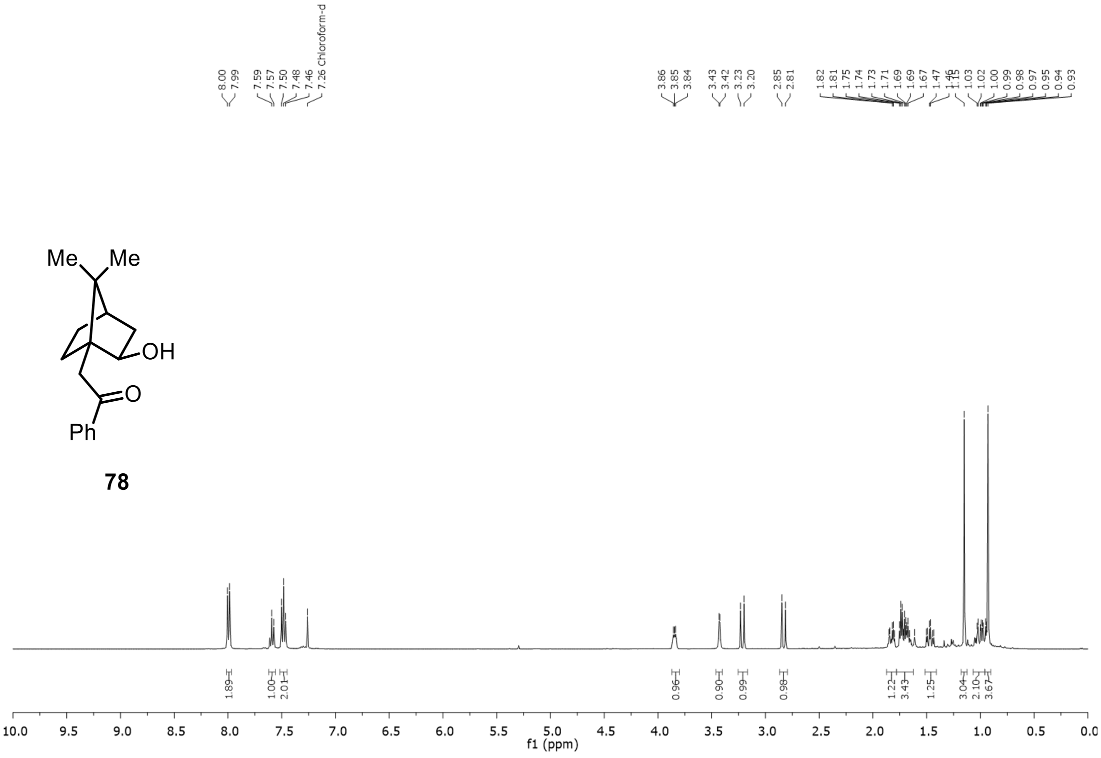

^{13}C NMR (101 MHz, CDCl$_3$)

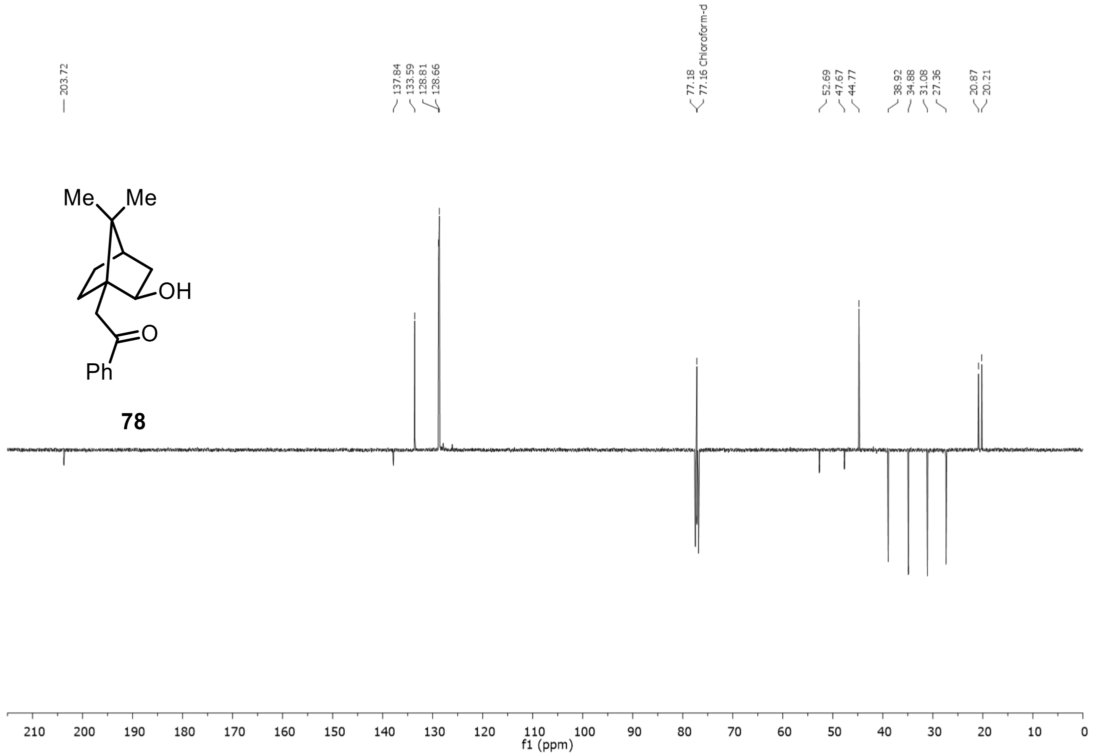

79: *syn*-(3-hydroxycyclododecyl)-2-methylpropan-1-one

^{1}H NMR (400 MHz, $CDCl_3$)

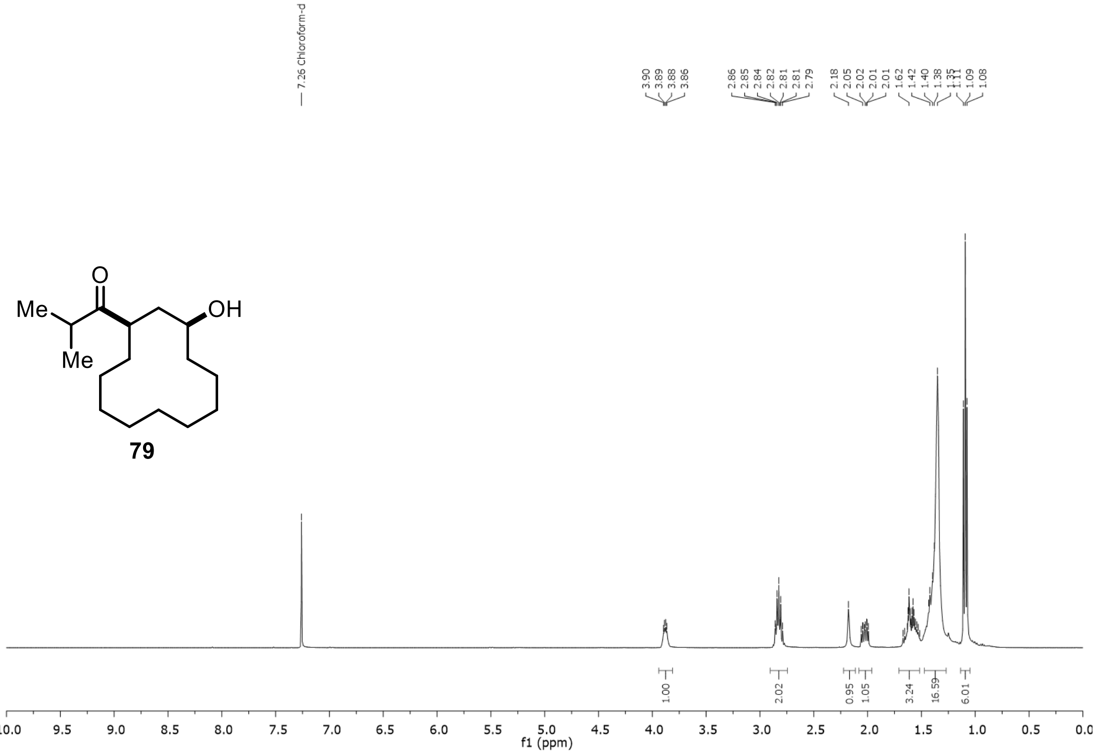

^{13}C NMR (176 MHz, $CDCl_3$)

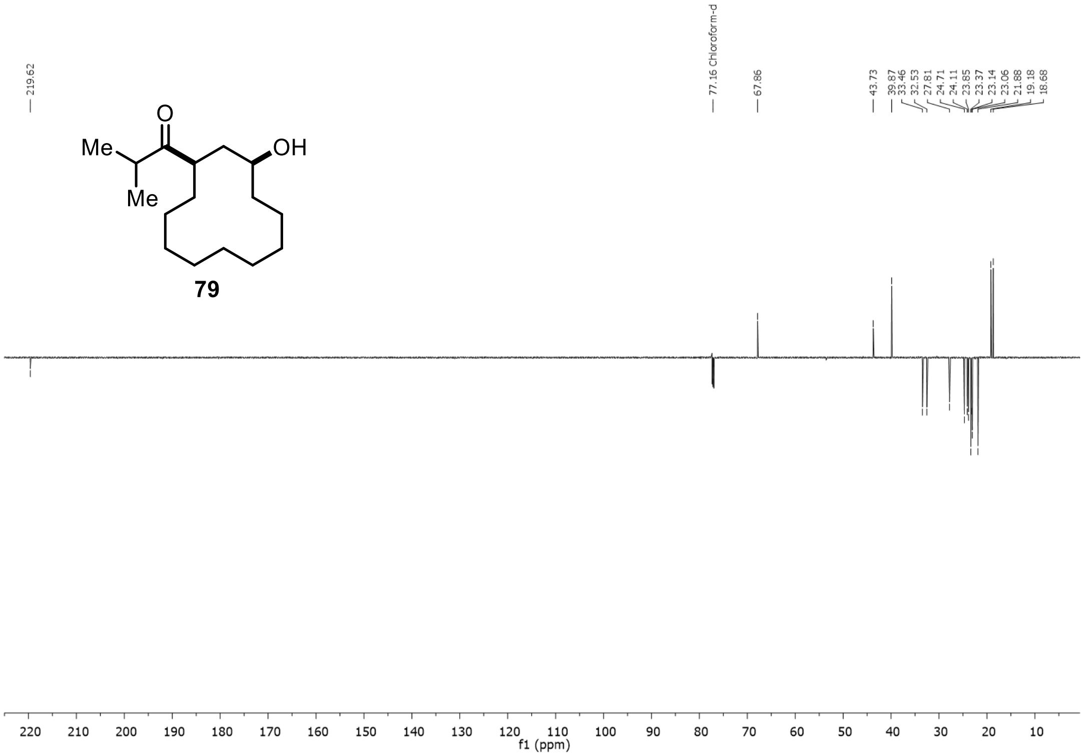

80: *syn*-(3-hydroxycyclohexyl)-3,3-dimethylbutan-1-one

^{1}H NMR (400 MHz, $CDCl_3$)

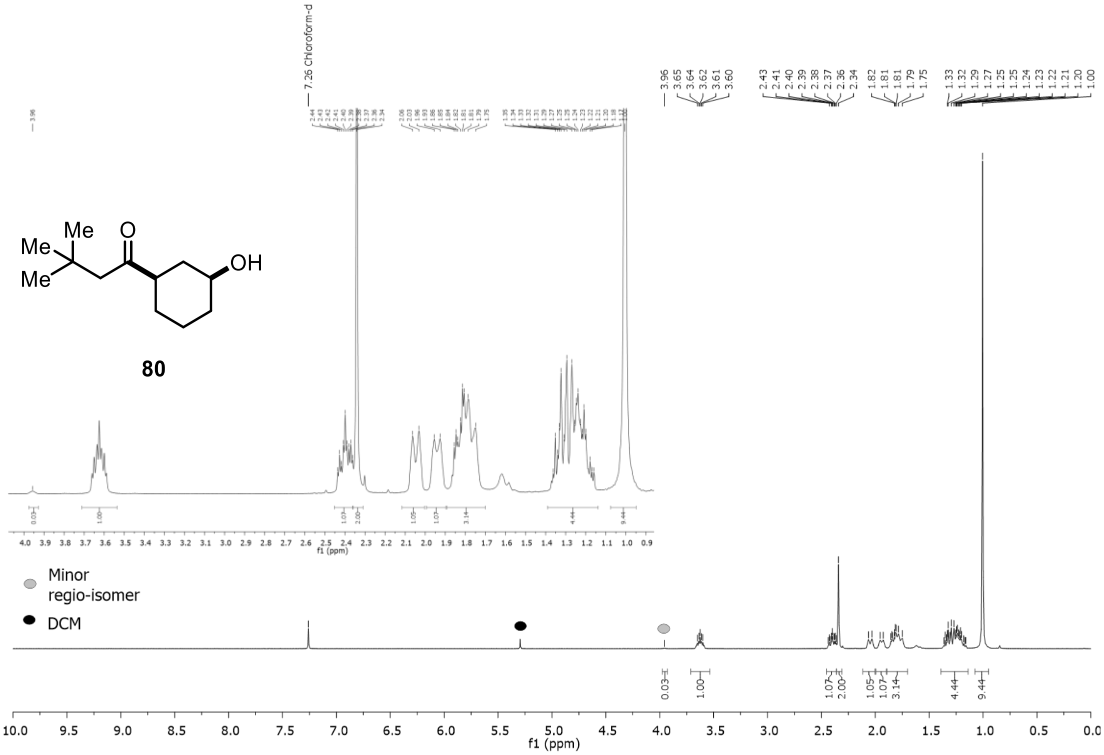

^{13}C NMR (101 MHz, $CDCl_3$)

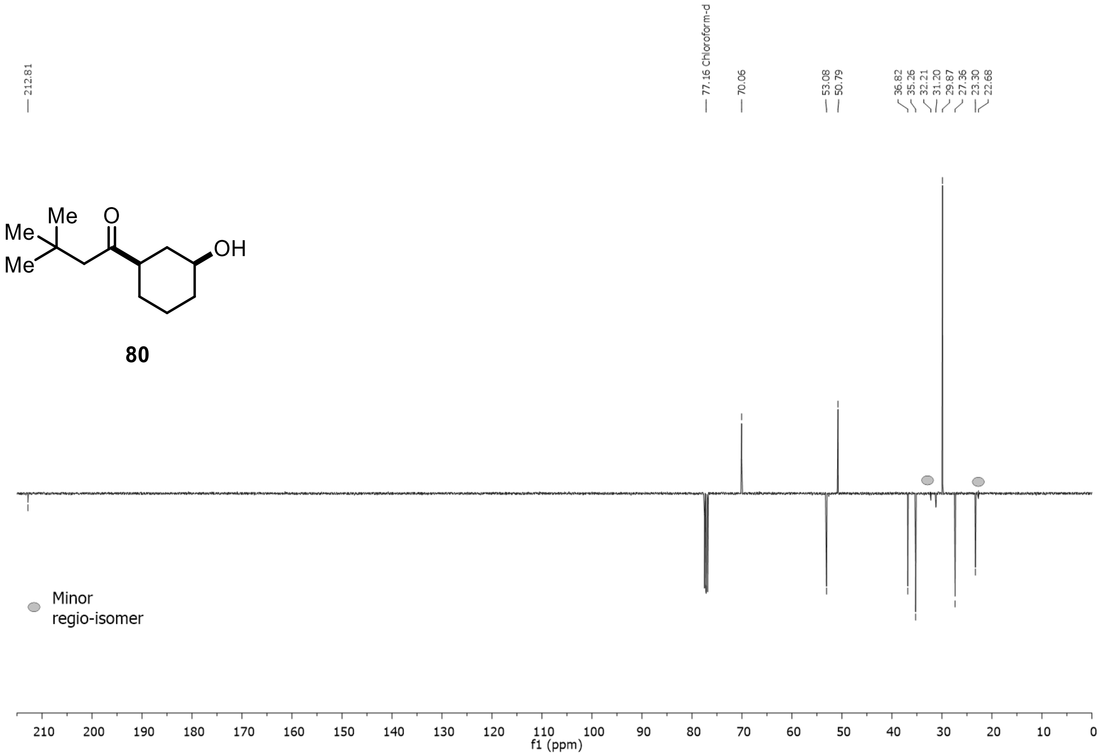

81: ***syn*-1-(3-hydroxycyclopentyl)-2,2-dimethylpropan-1-one**

^{1}H NMR (700 MHz, CDCl$_3$)

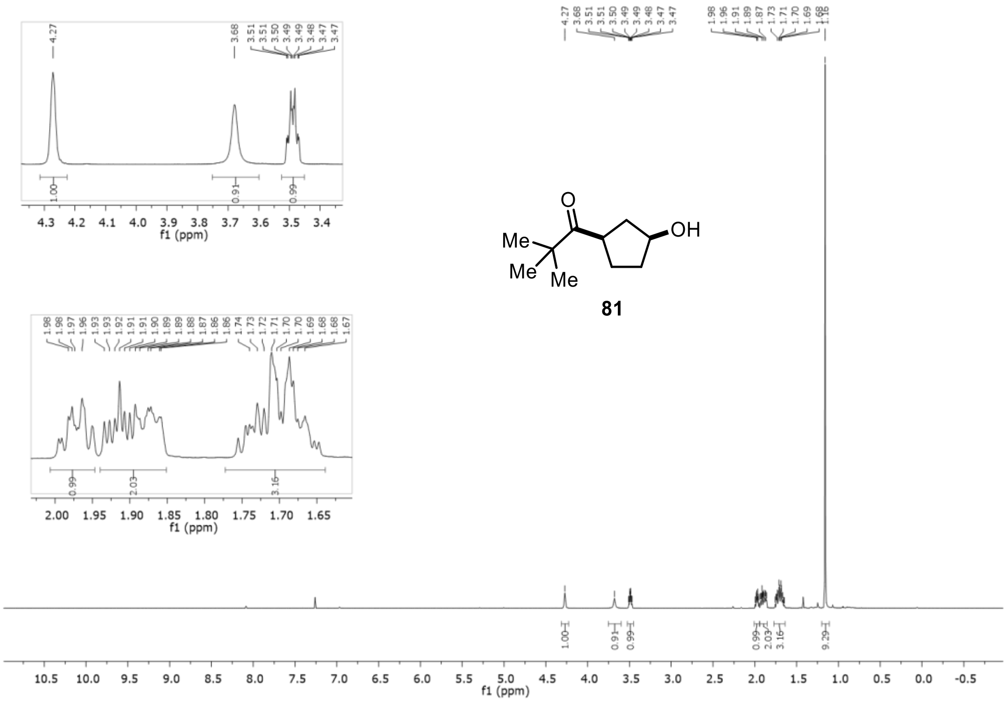

^{13}C NMR (176 MHz, CDCl$_3$)

As the carbonyl was not sufficiently visible in the ^{13}C DEPTQ-135 NMR spectrum, an additional ^{13}C CPD NMR spectrum is attached.

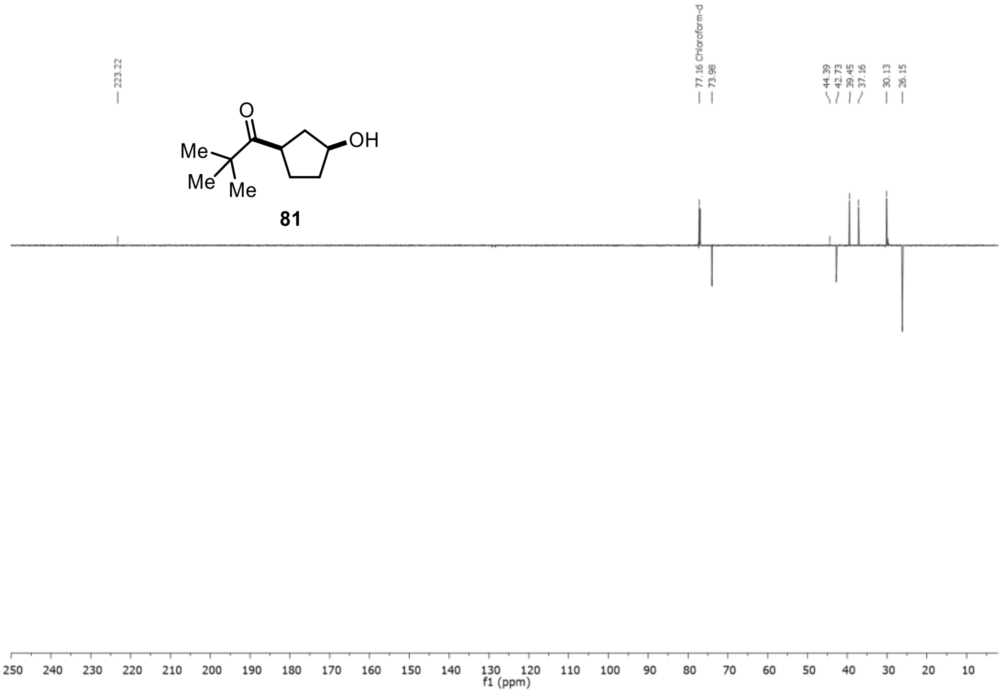

^{13}C NMR (176 MHz, $CDCl_3$)

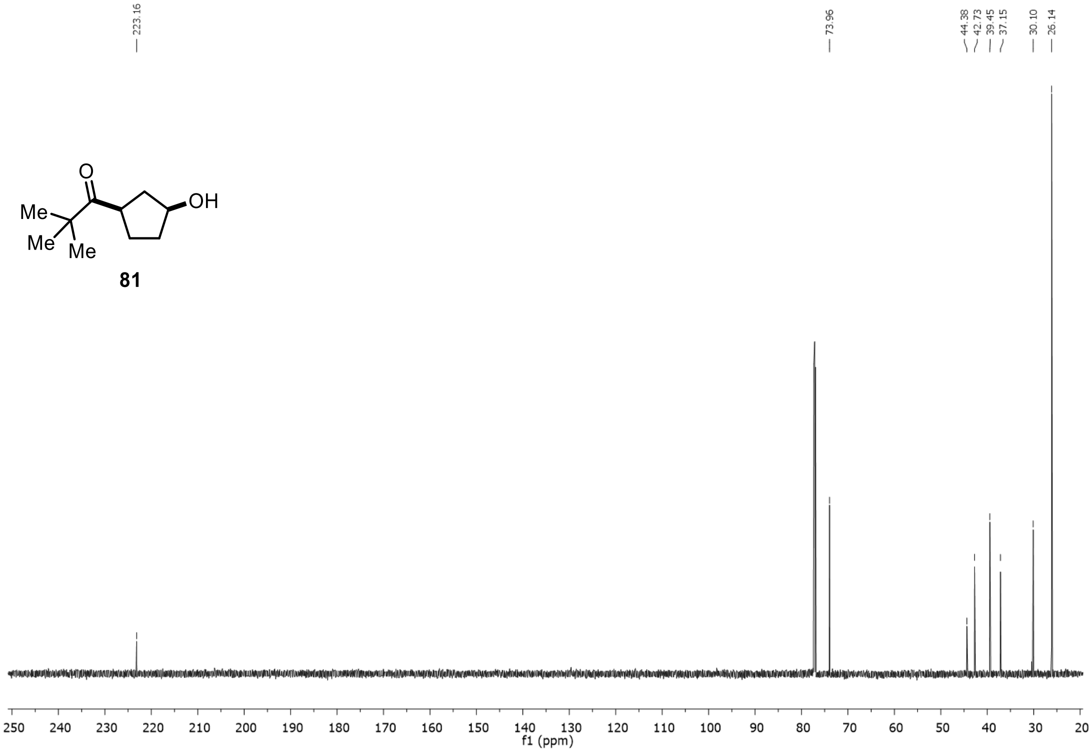

82: *syn*-(3-hydroxycyclohexyl)-2-methylpropan-1-one

^{1}H NMR (600 MHz, $CDCl_3$)

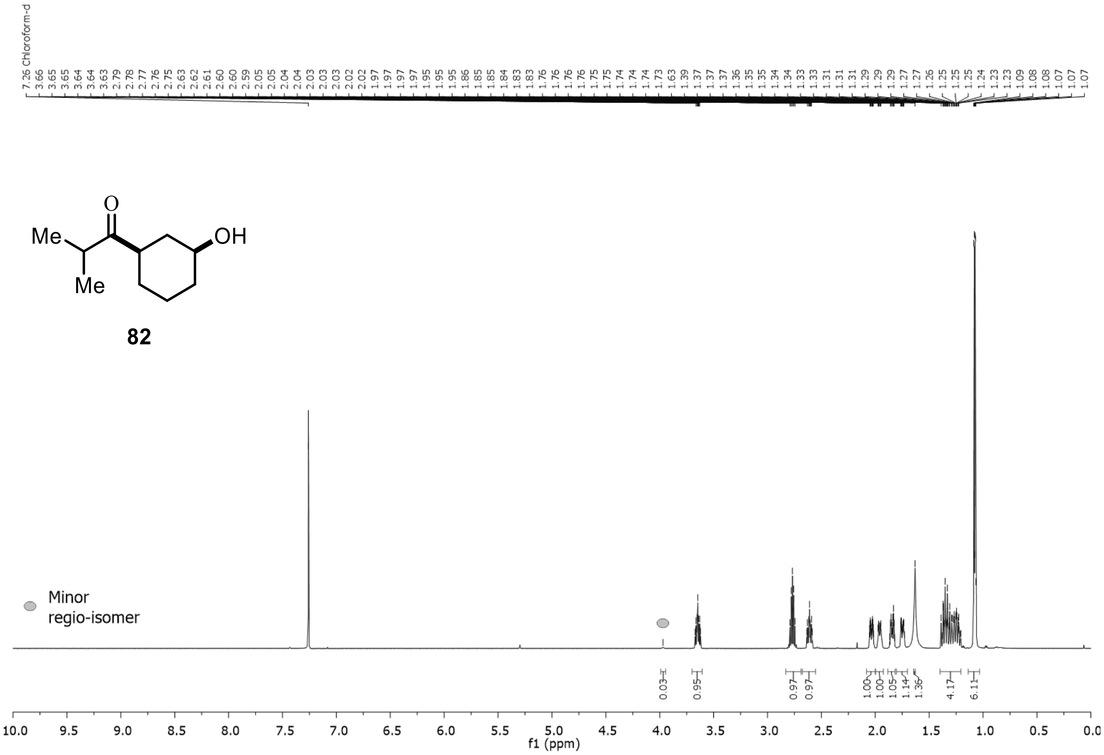

^{13}C NMR (151 MHz, $CDCl_3$)

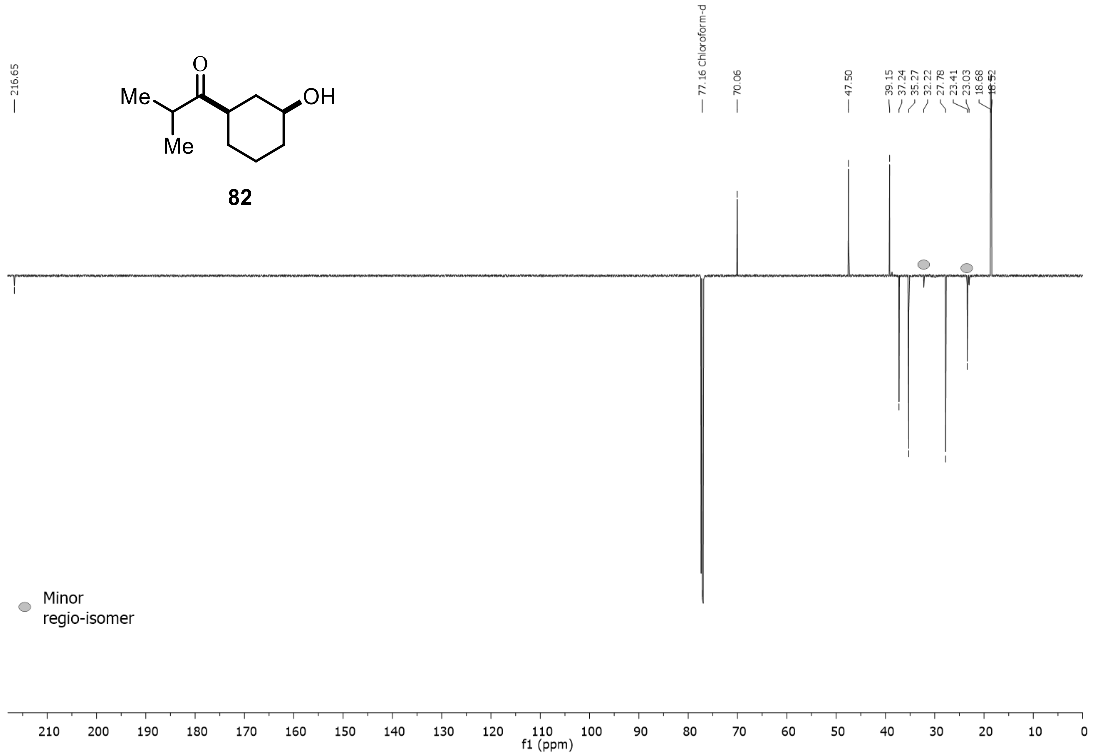

29: *anti*-(3-hydroxycyclohexyl)(4-(trifluoromethyl)phenyl)methanone

^{1}H NMR (400 MHz, $CDCl_3$)

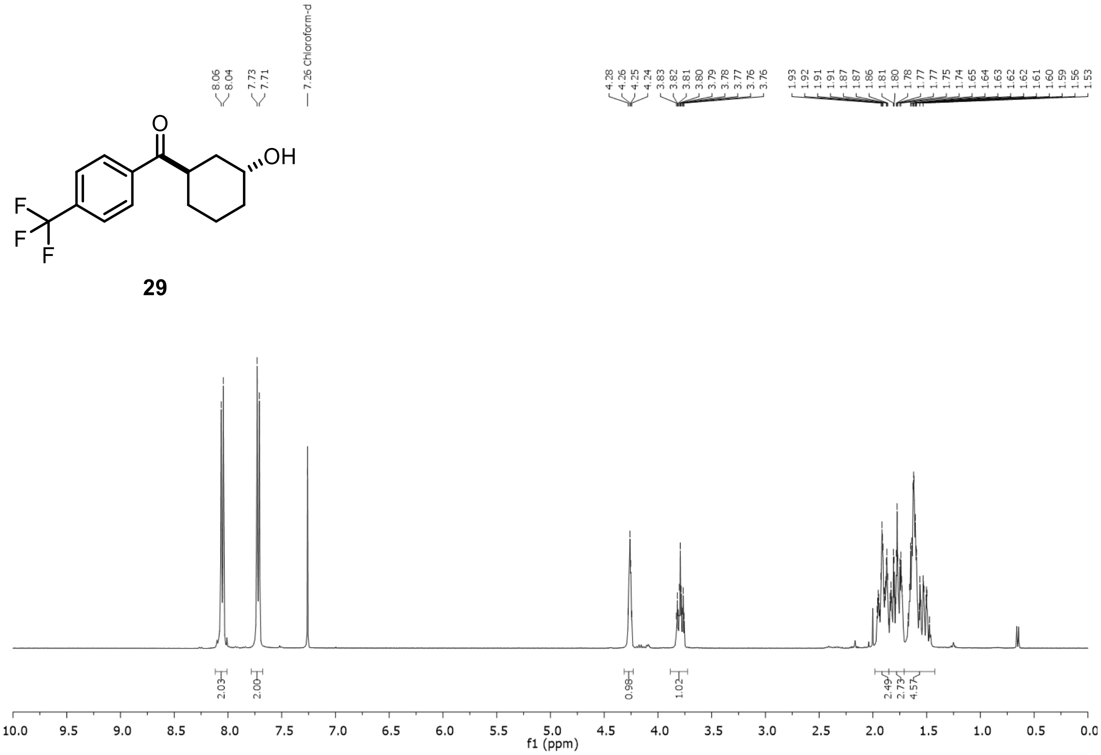

^{13}C NMR (101 MHz, $CDCl_3$)

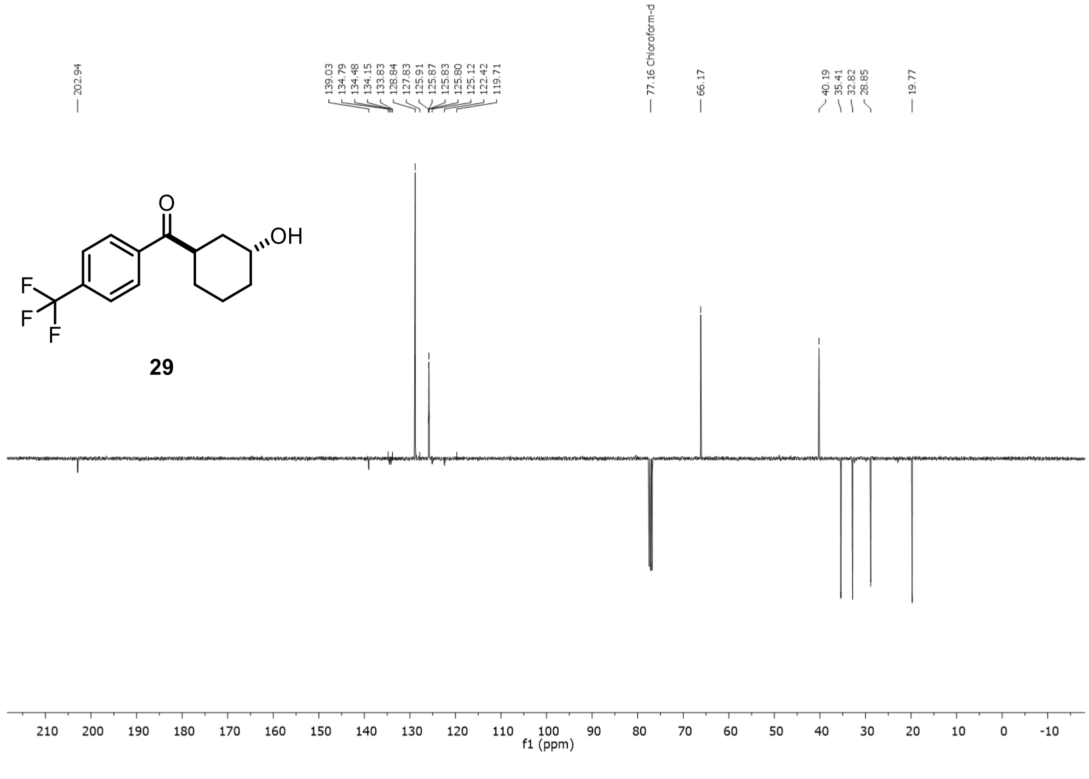

^{19}F NMR (377 MHz, $CDCl_3$)

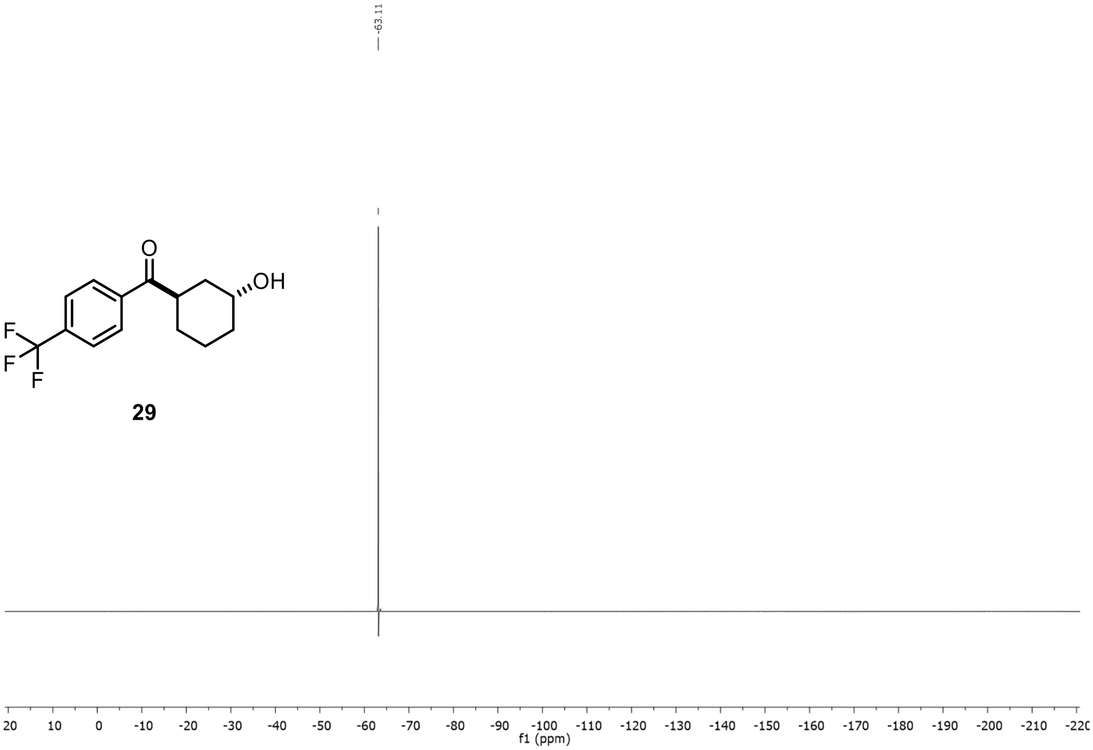

30: *anti*-(3-hydroxycyclohexyl)-2,2-dimethylpropan-1-one

^{1}H NMR (700 MHz, $CDCl_3$)

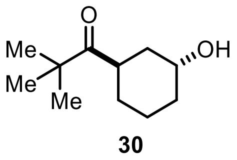

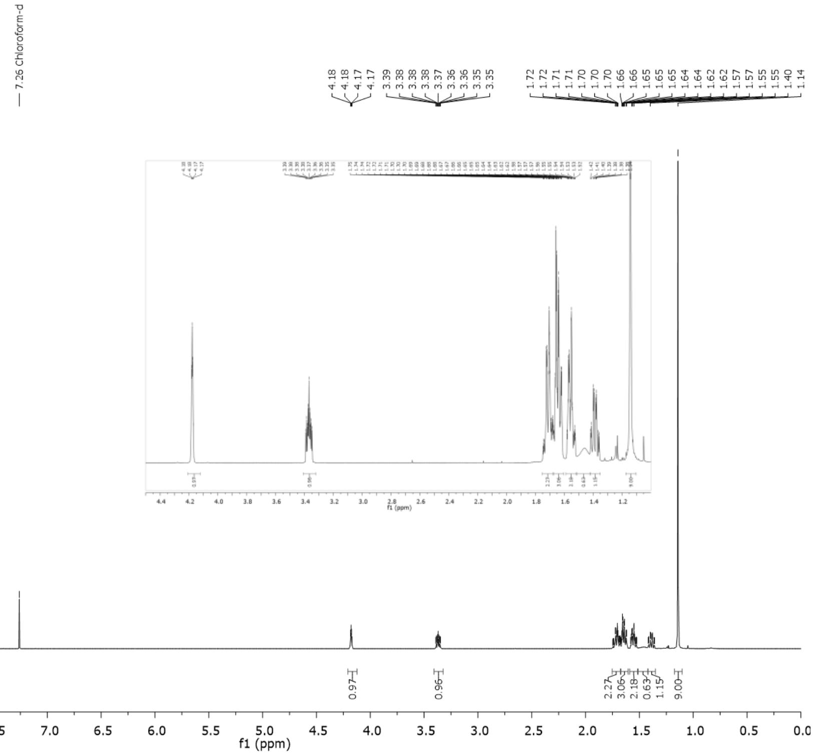

^{13}C NMR (176 MHz, $CDCl_3$)

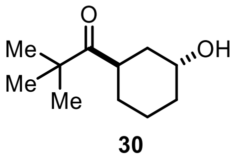

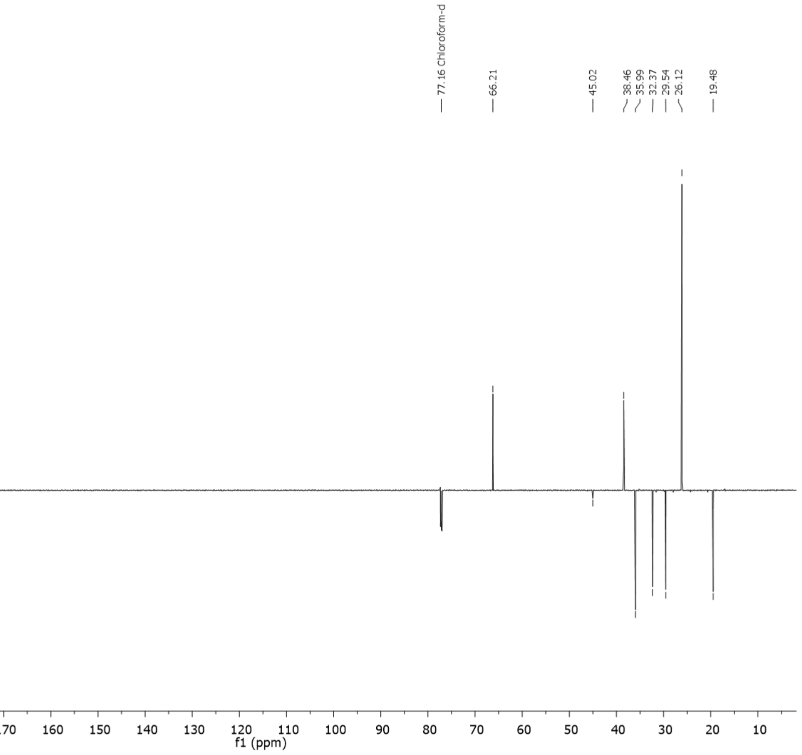

31: *anti*-(4-(tert-butyl)phenyl)(3-hydroxycyclopentyl)methanone

^{1}H NMR (400 MHz, $CDCl_3$)

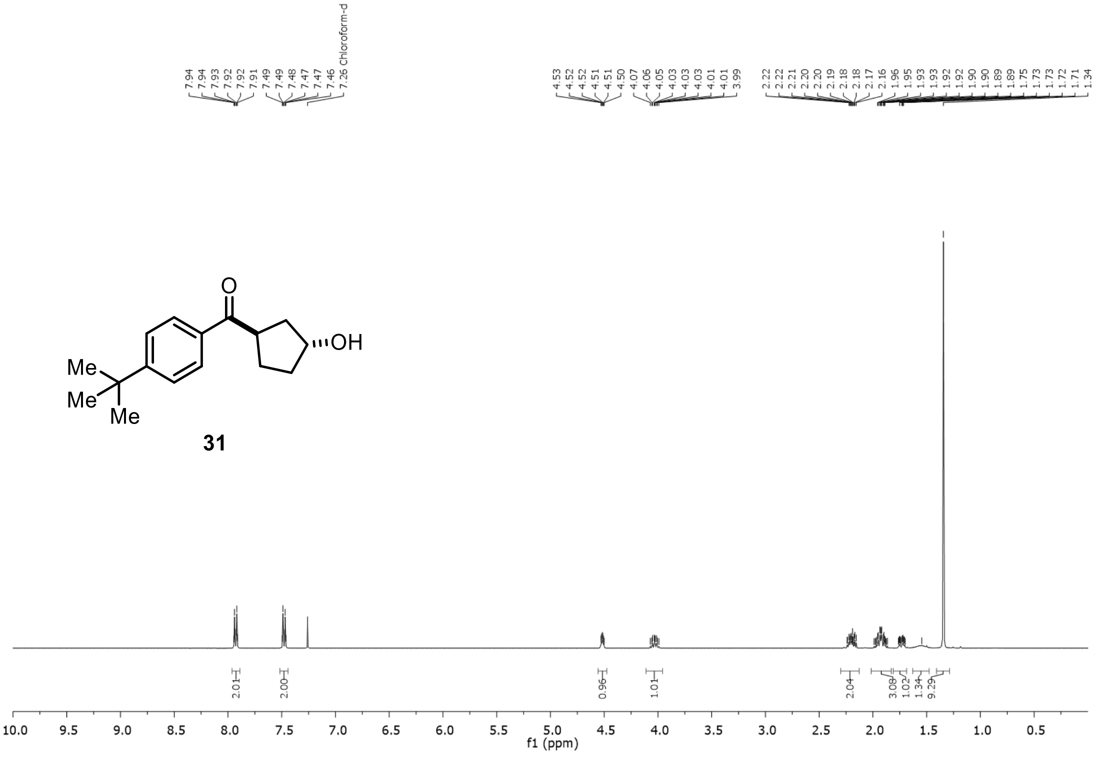

^{13}C NMR (101 MHz, $CDCl_3$)

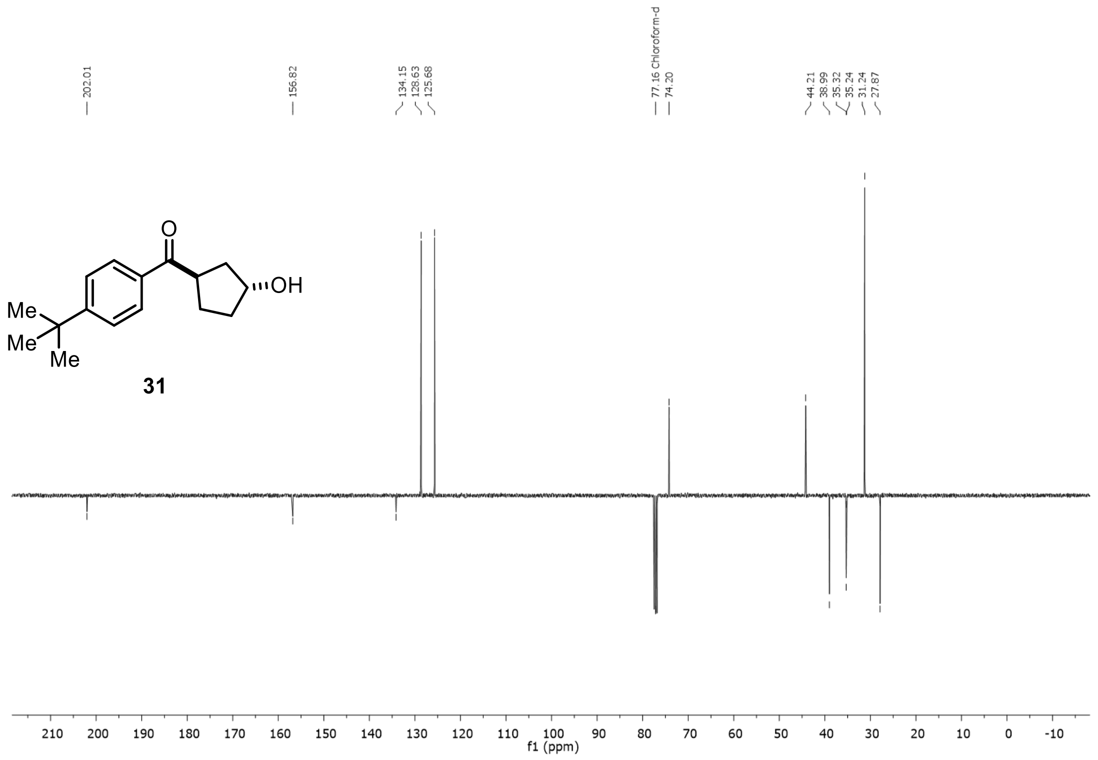

32: *anti*-(3-hydroxycyclopentyl)(m-tolyl)methanone

^{1}H NMR (400 MHz, $CDCl_3$)

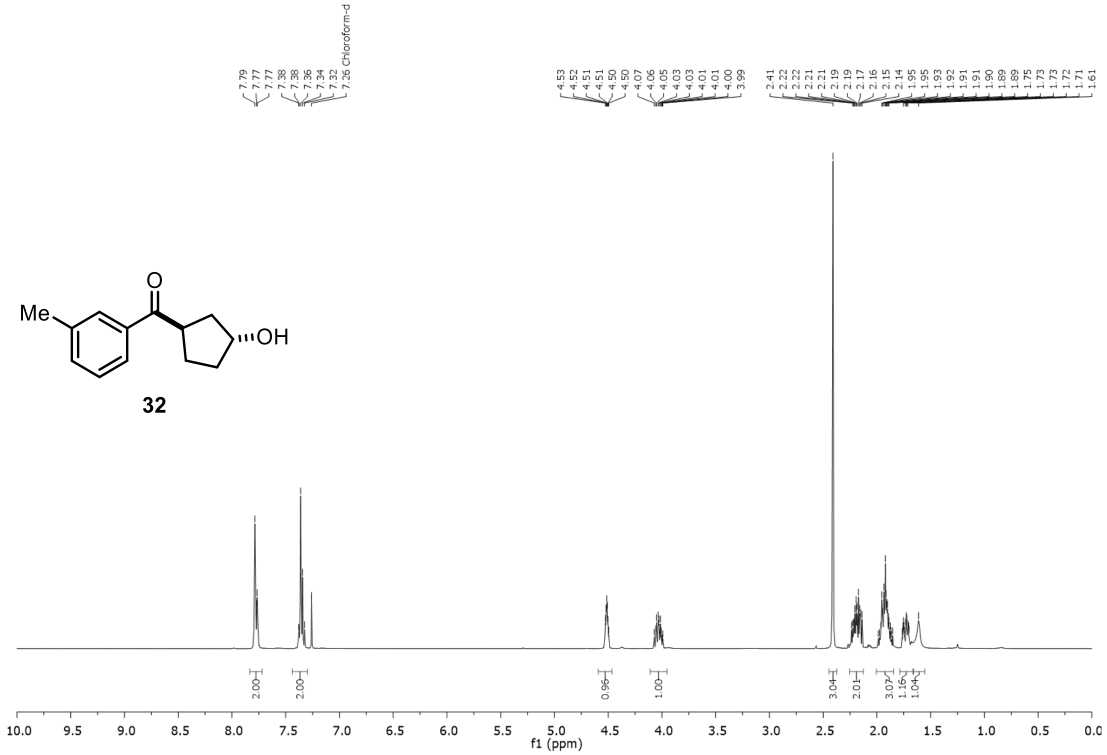

^{13}C NMR (101 MHz, $CDCl_3$)

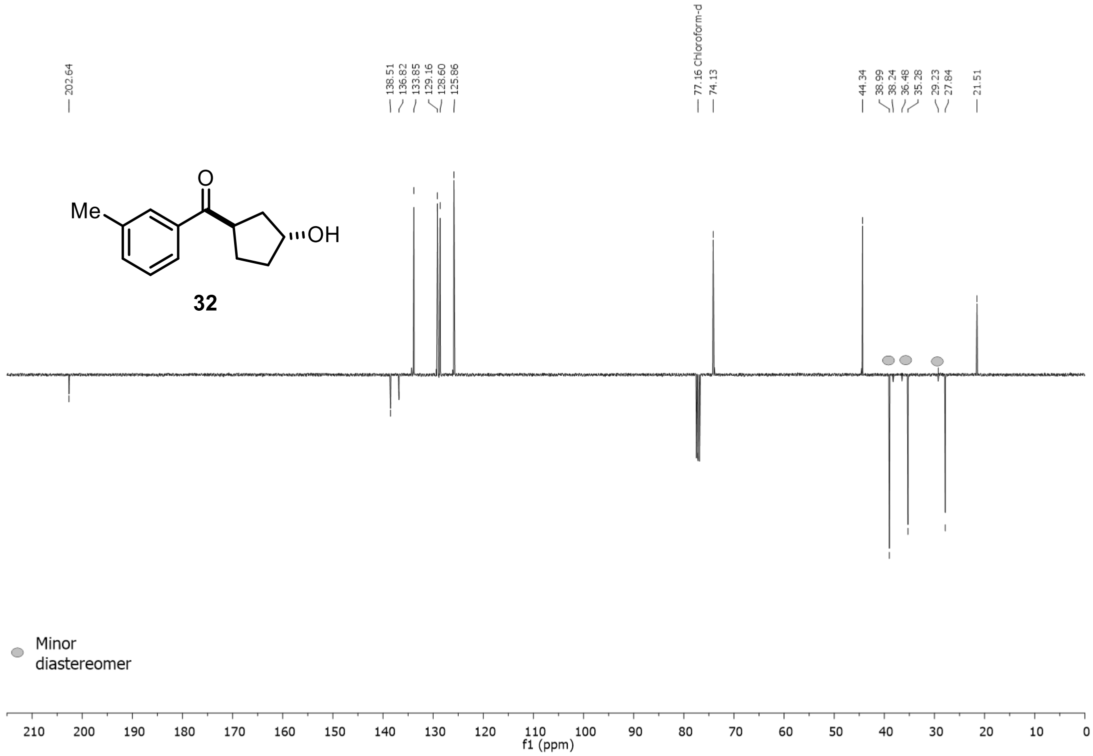

33: *anti*-(3-hydroxycyclohexyl)(thiophen-2-yl)methanone

^{1}H NMR (400 MHz, $CDCl_3$)

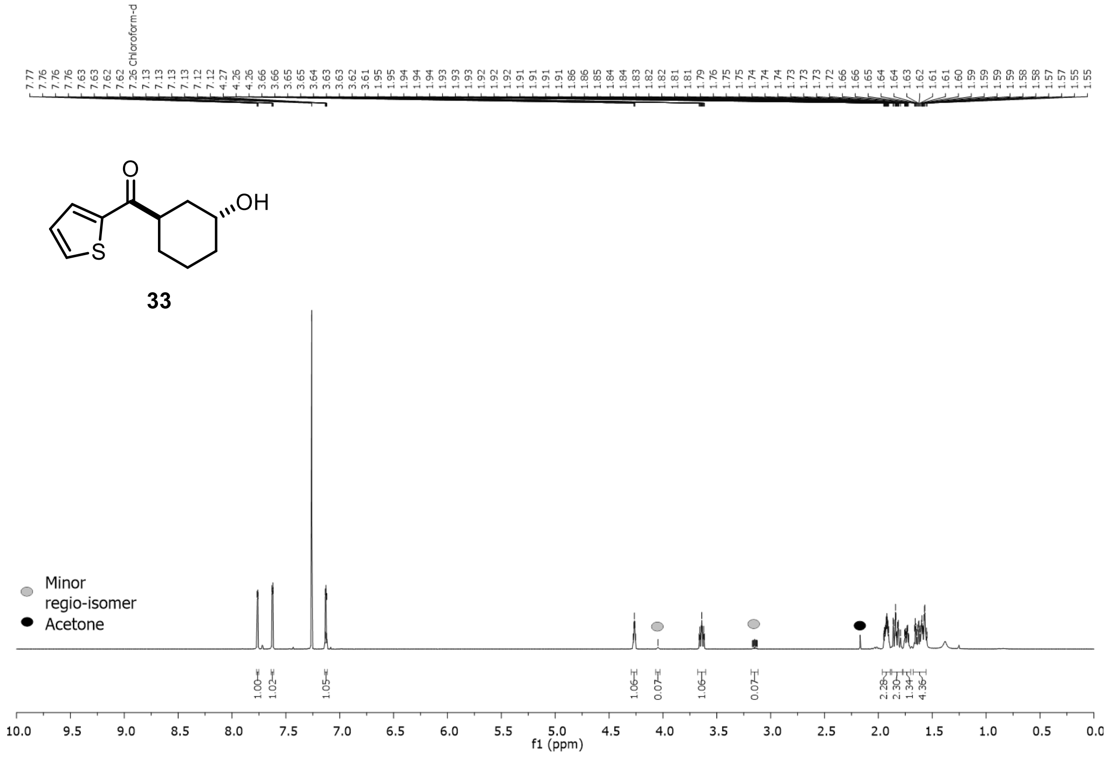

^{13}C NMR (101 MHz, $CDCl_3$)

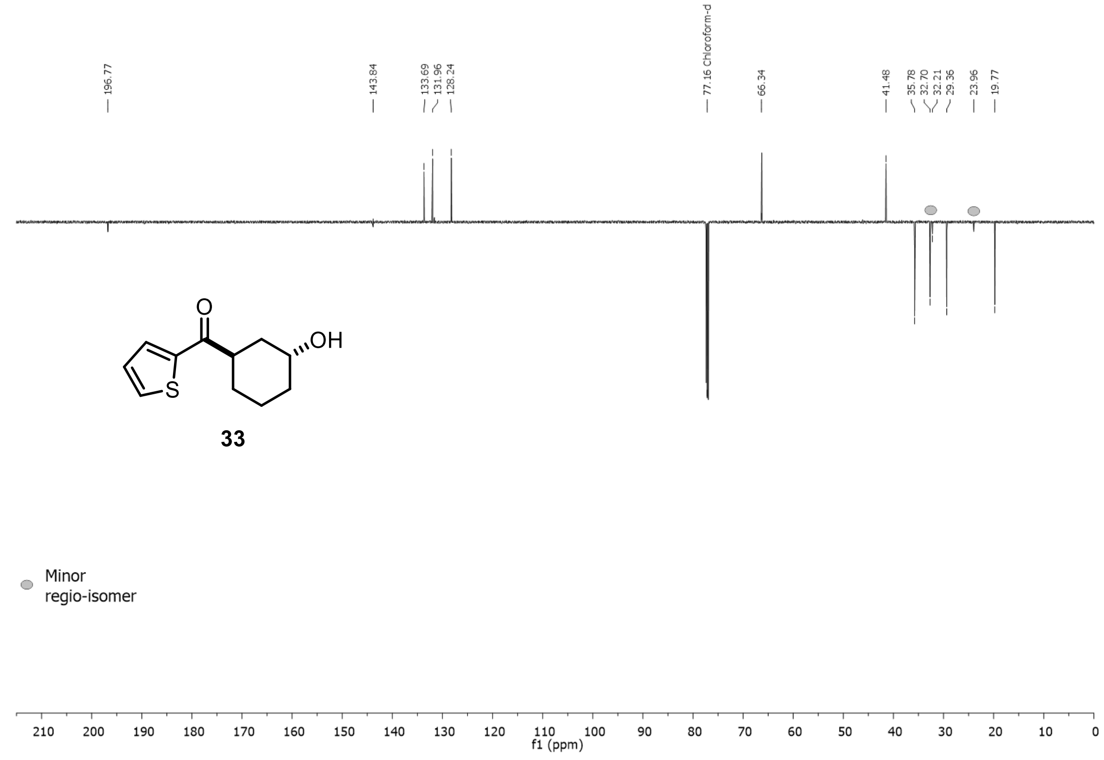

34: *anti*-(3-hydroxycyclohexyl)(naphthalen-2-yl)methanone

^{1}H NMR (400 MHz, $CDCl_3$)

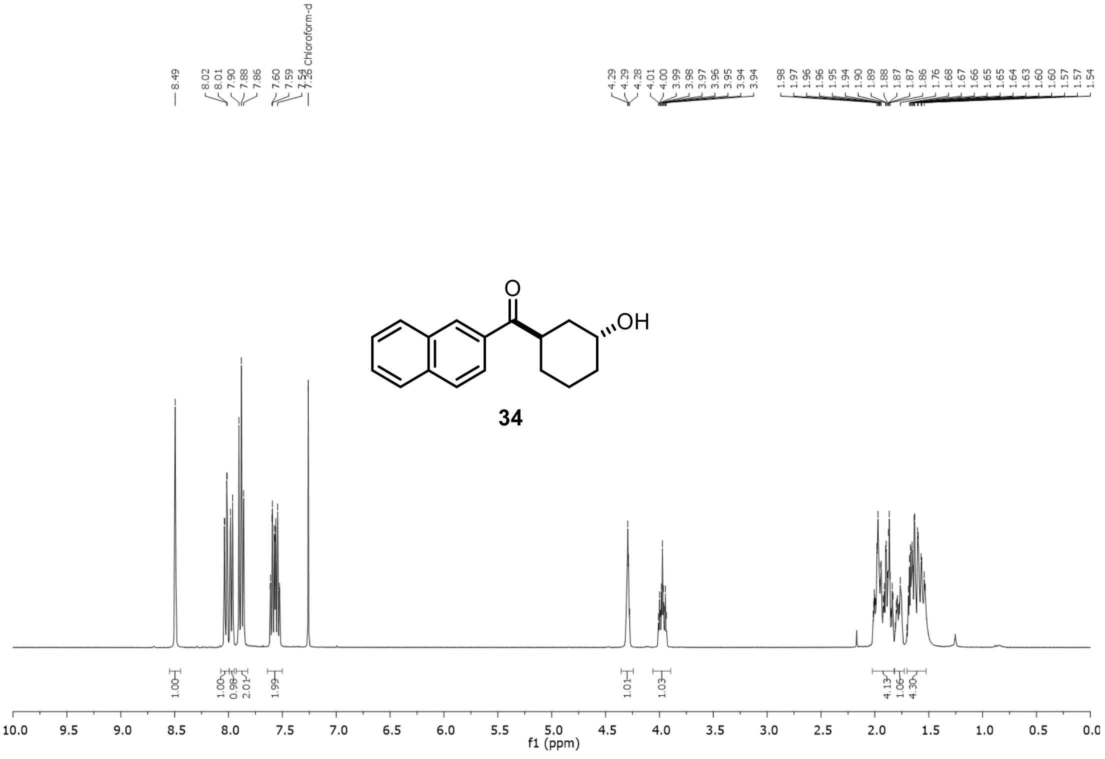

^{13}C NMR (101 MHz, $CDCl_3$)

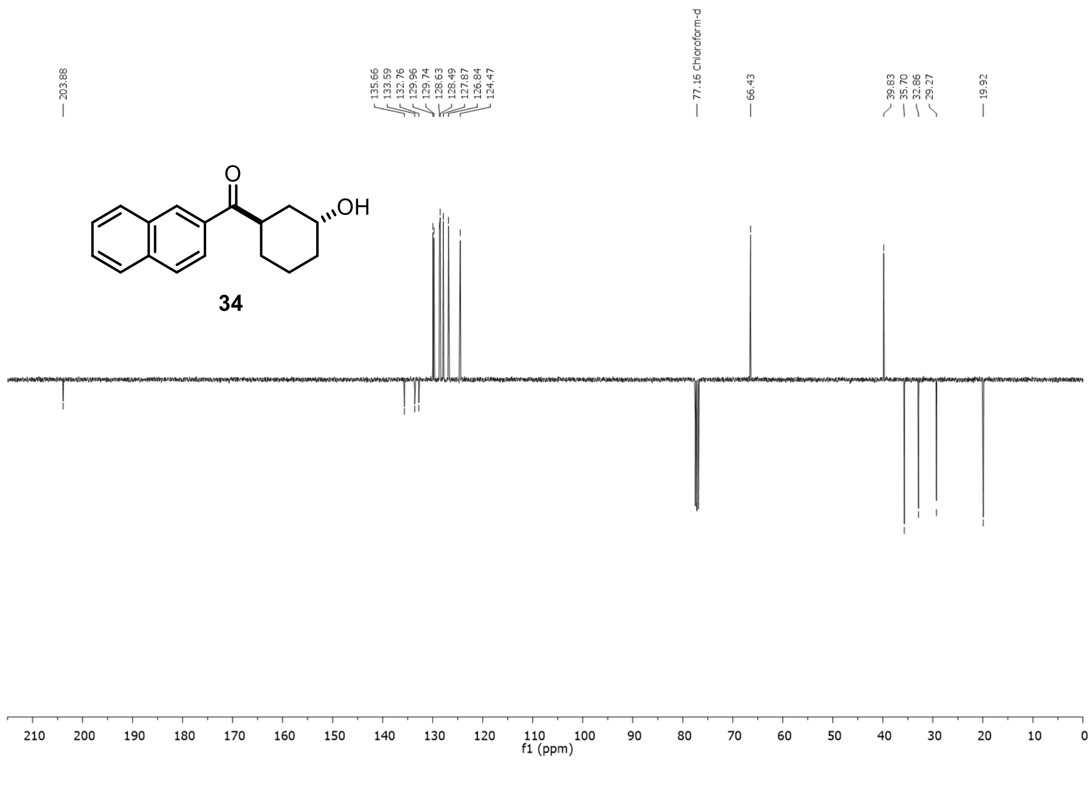

35: *anti*-(3-hydroxycyclohexyl)(thiophen-2-yl)methanone

^{1}H NMR (400 MHz, $CDCl_3$)

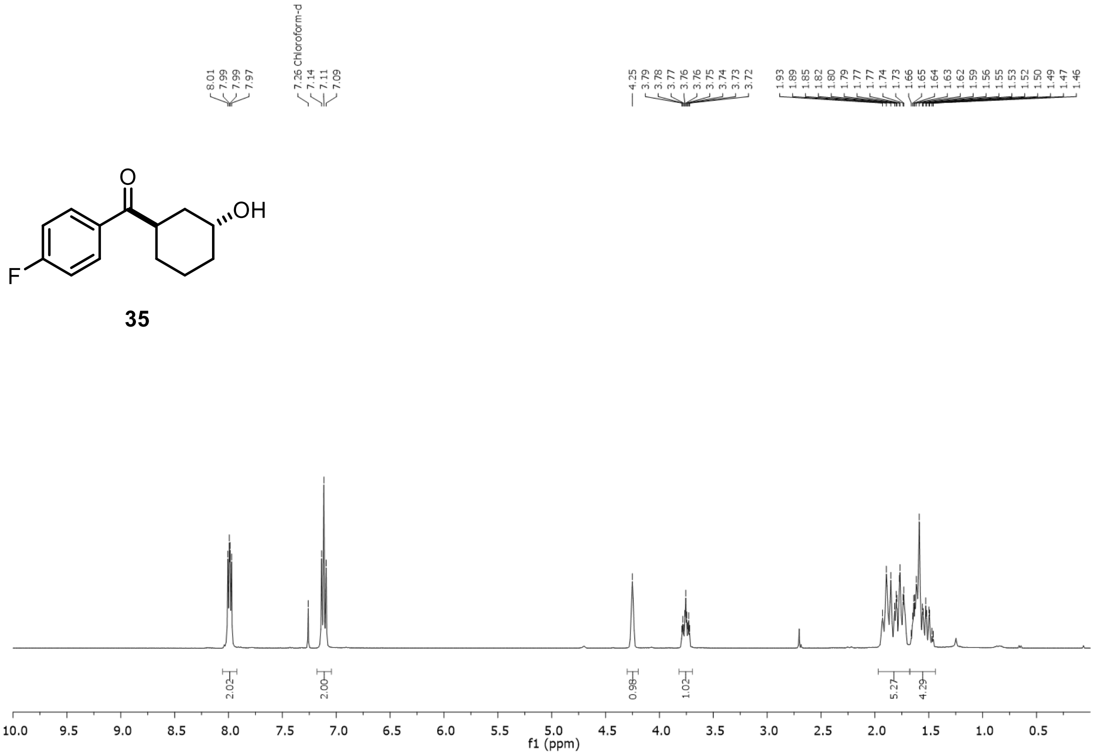

^{13}C NMR (151 MHz, $CDCl_3$)

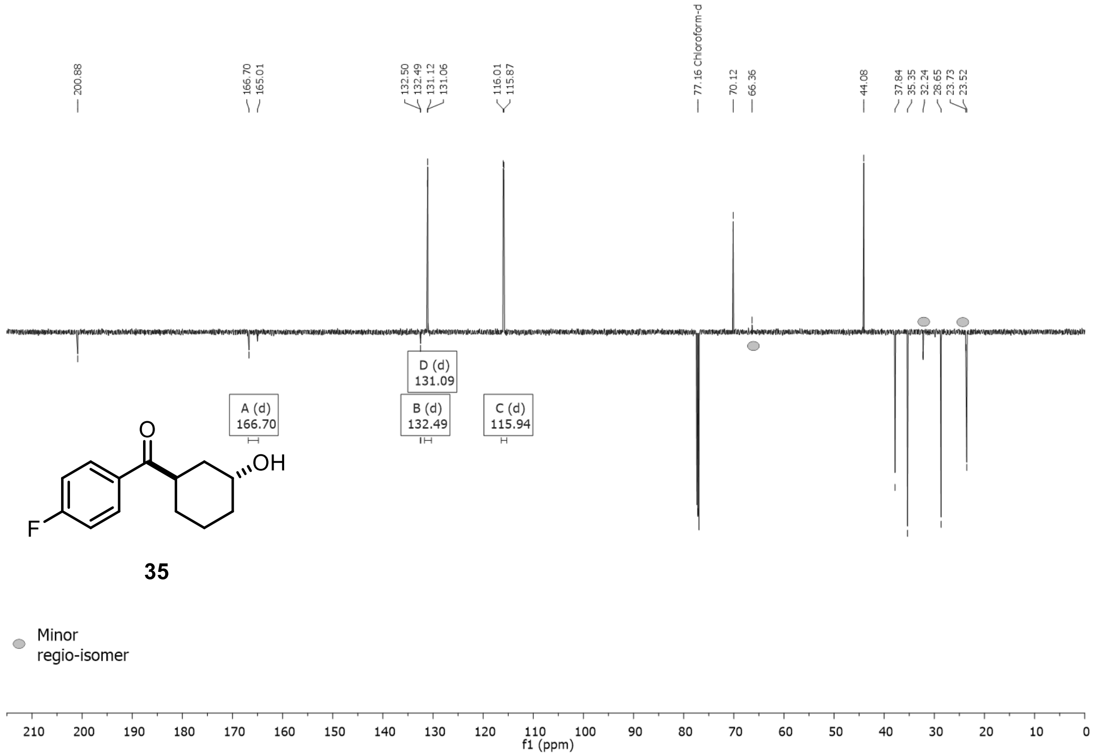

^{19}F NMR (377 MHz, $CDCl_3$)

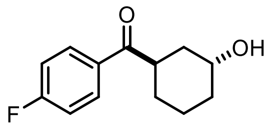

35

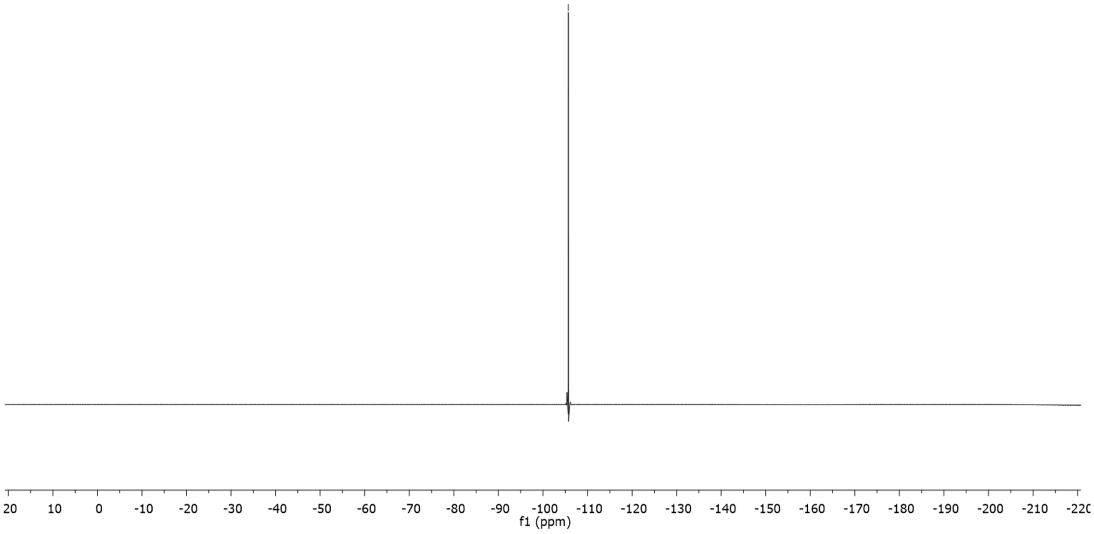

36: *anti*-(3-hydroxycyclopentyl)(phenyl)methanone

^{1}H NMR (400 MHz, $CDCl_3$)

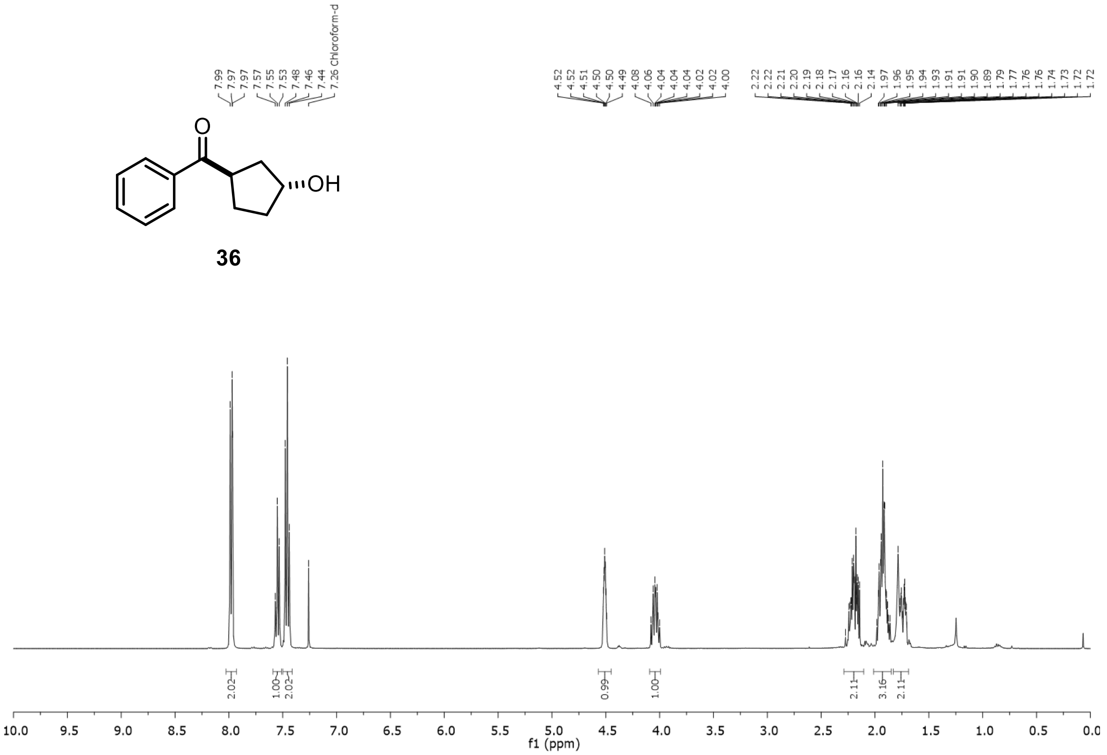

^{13}C NMR (101 MHz, $CDCl_3$)

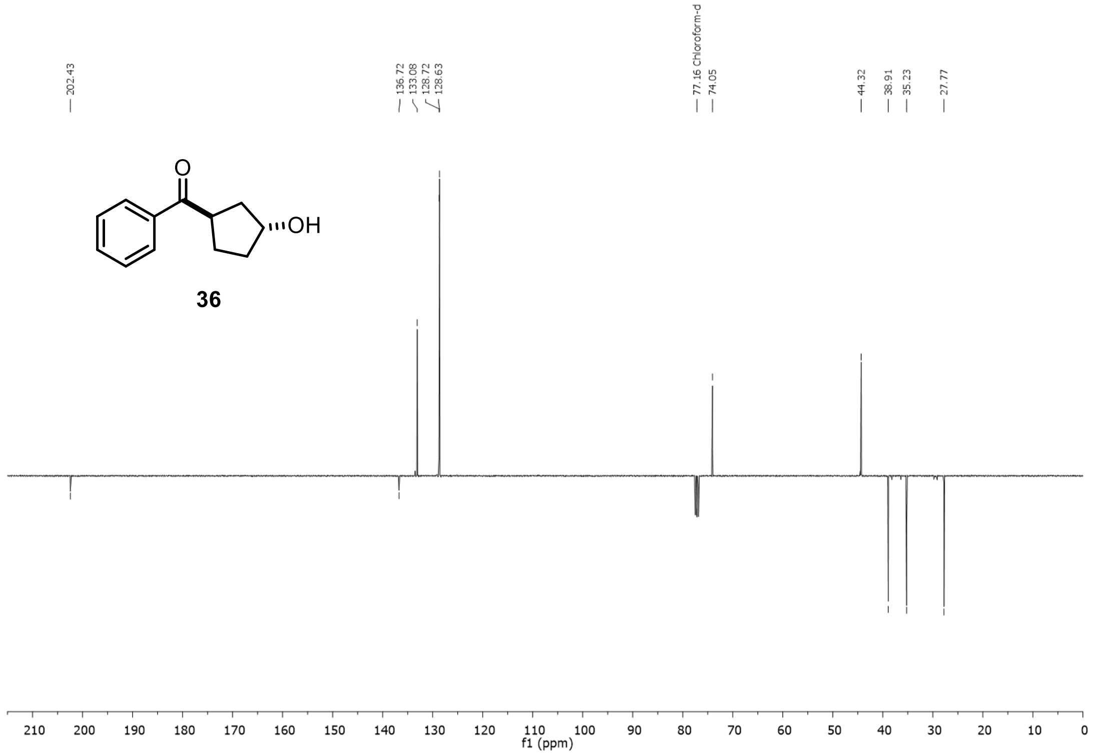

37: *anti*-(3-hydroxycyclohexyl)(4-(trifluoromethoxy)phenyl)methanone

^{1}H NMR (400 MHz, $CDCl_3$)

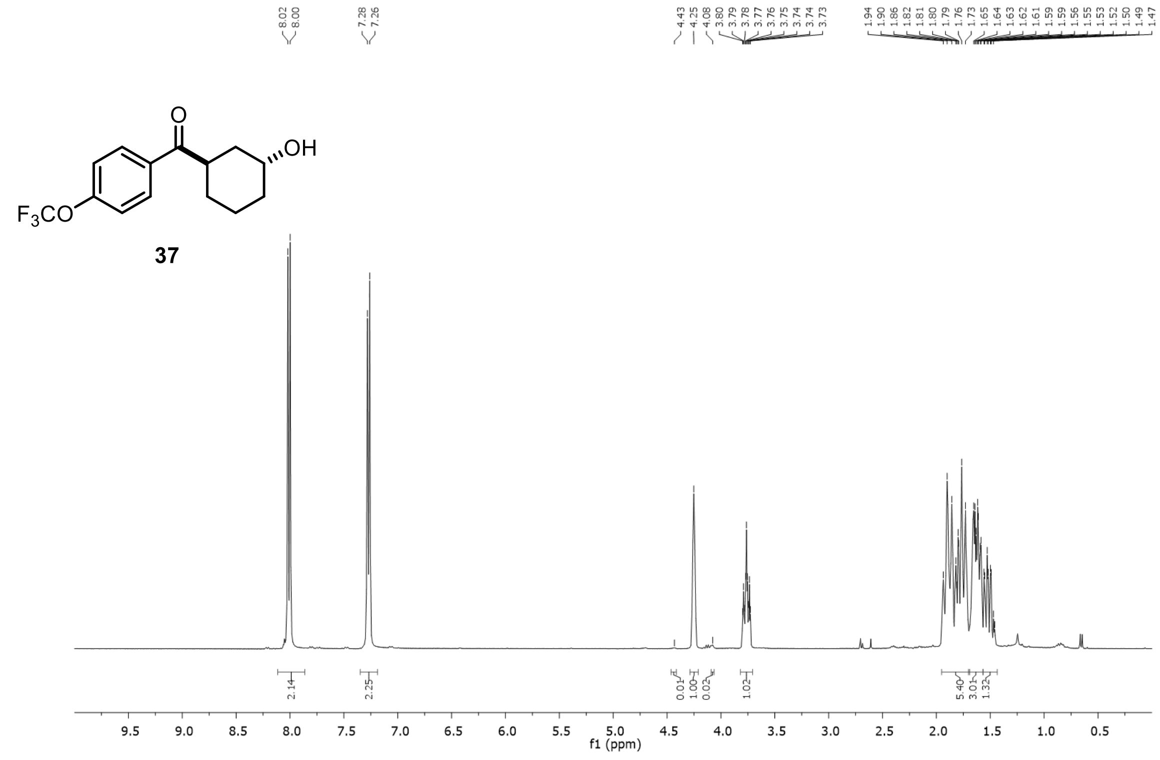

^{13}C NMR (101 MHz, $CDCl_3$)

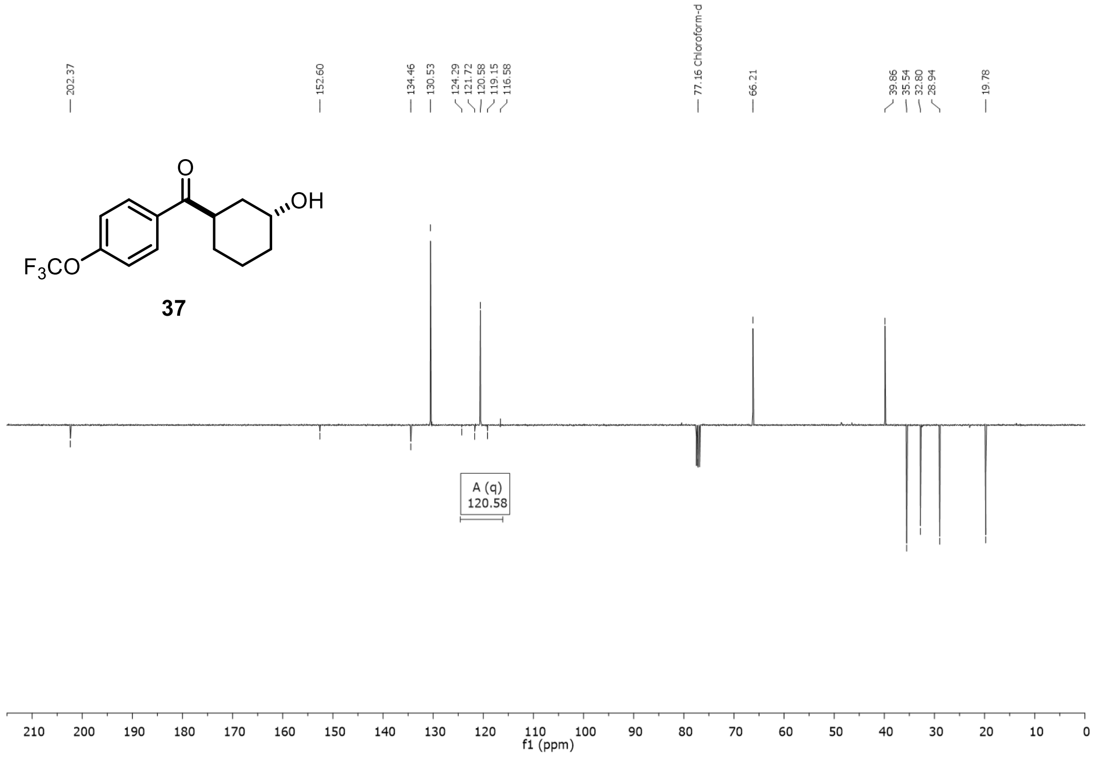

^{19}F NMR (377 MHz, $CDCl_3$)

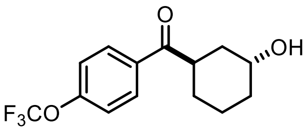

37

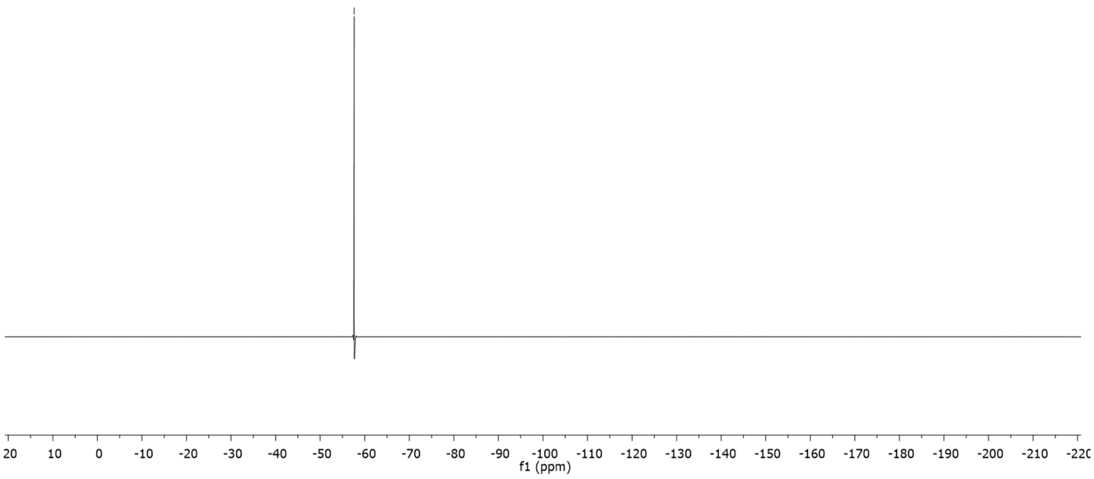

83: *anti*-(3-hydroxycyclohexyl)(phenyl)methanone

^{1}H NMR (400 MHz, $CDCl_3$)

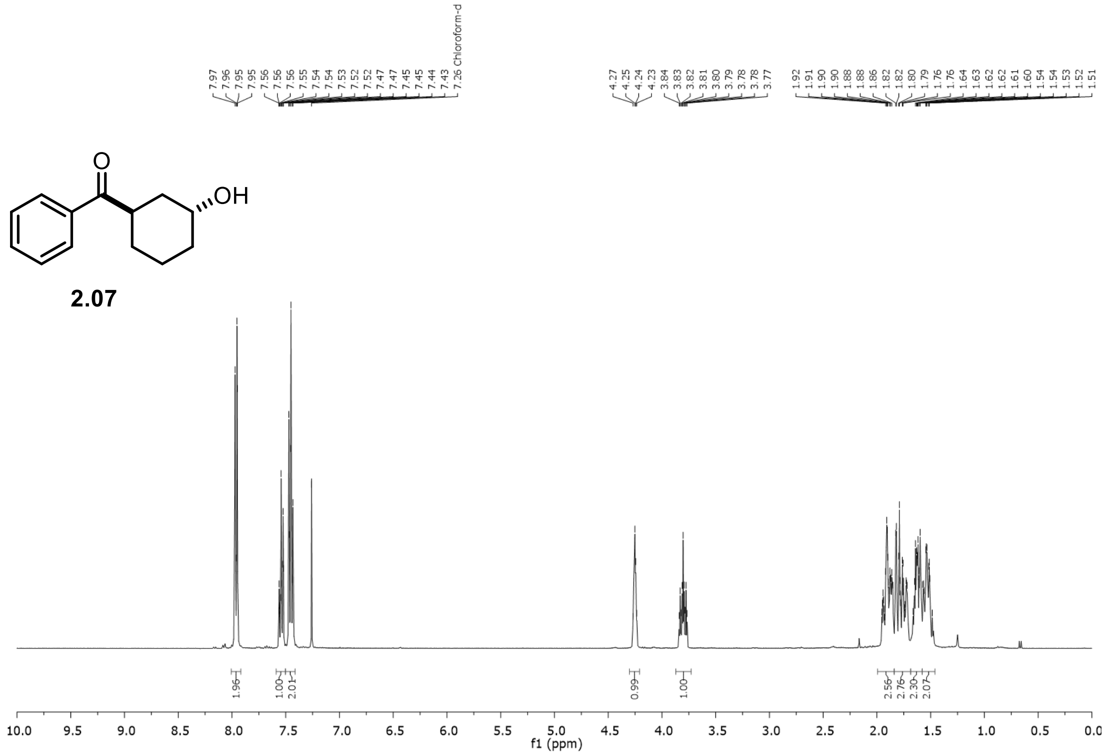

^{13}C NMR (151 MHz, $CDCl_3$)

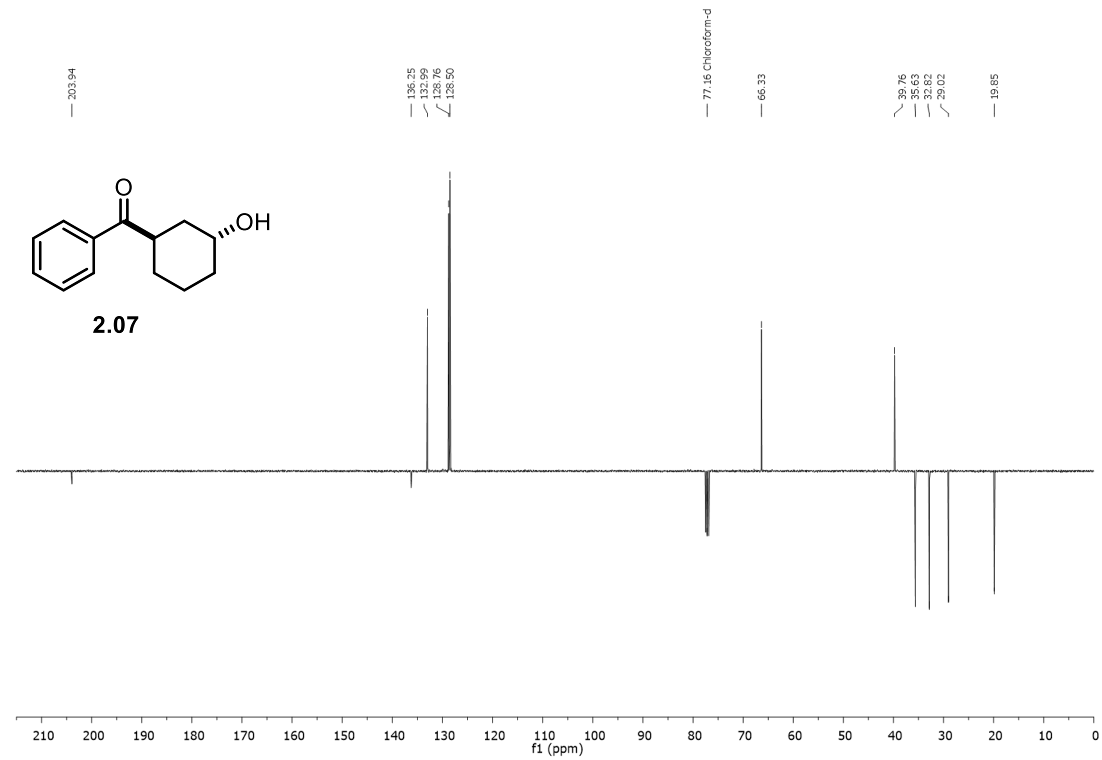

38: *anti*-(3-chlorocyclohexyl)(phenyl)methanone

^{1}H NMR (600 MHz, $CDCl_3$)

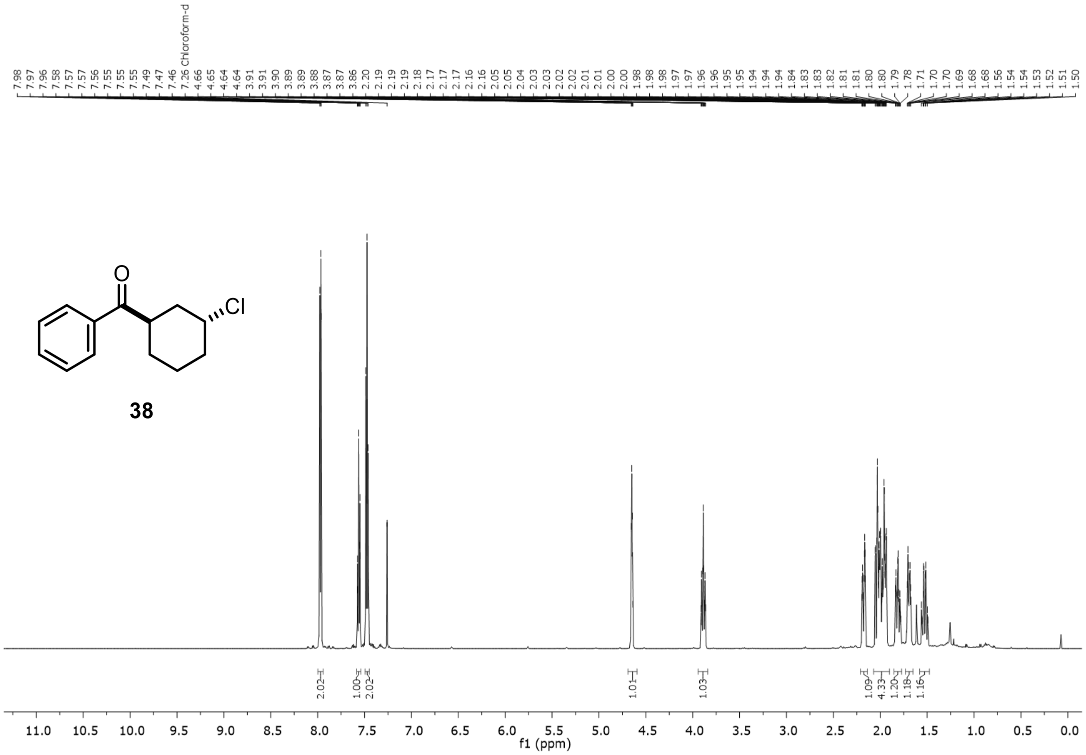

^{13}C NMR (151 MHz, $CDCl_3$)

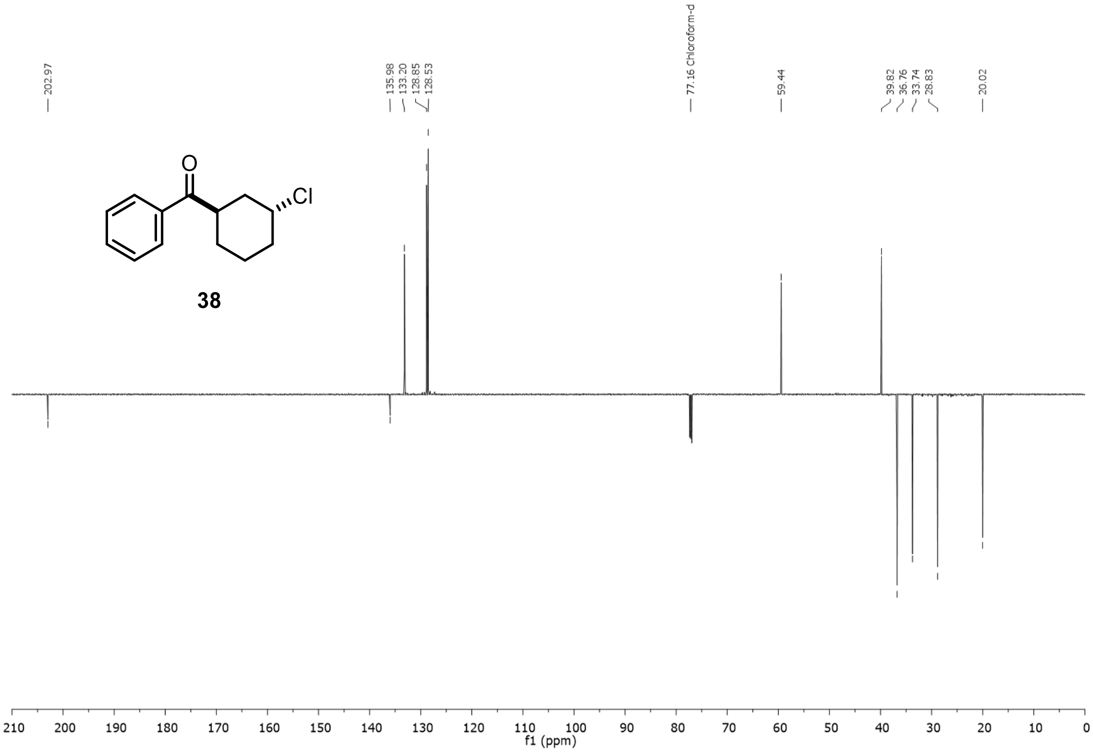

39: *anti*–((3-bromocyclohexyl)(phenyl)methanone

^{1}H NMR (600 MHz, $CDCl_3$)

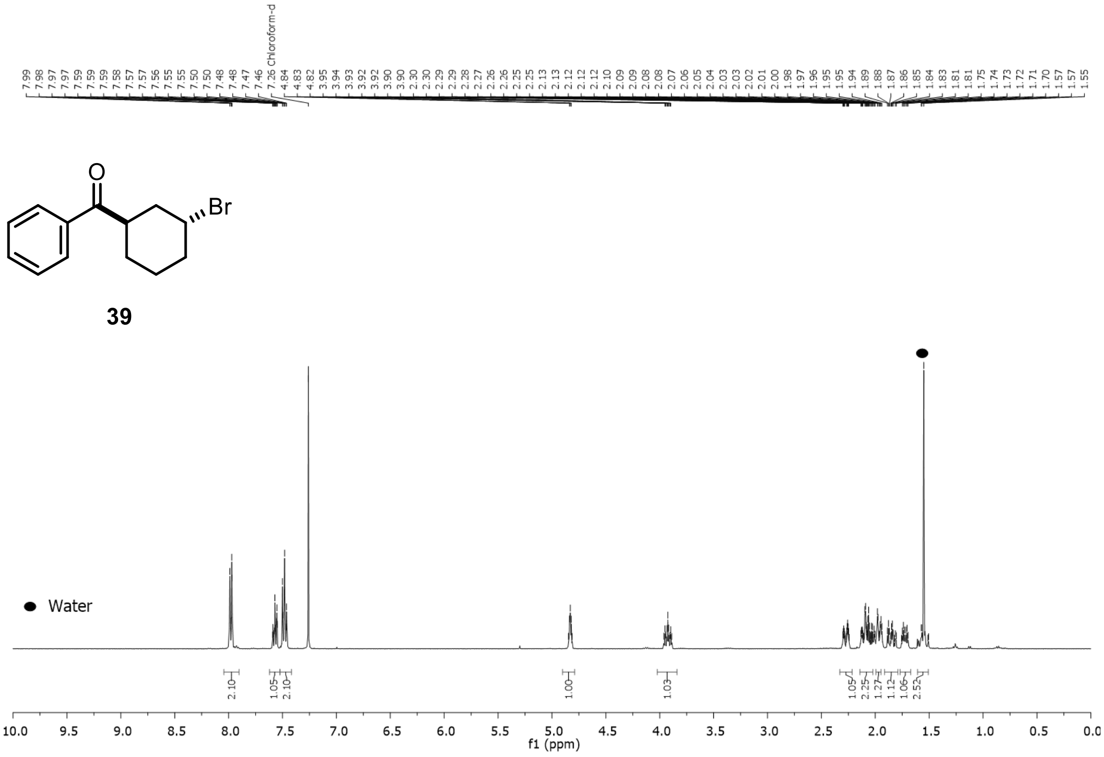

^{13}C NMR (151 MHz, $CDCl_3$)

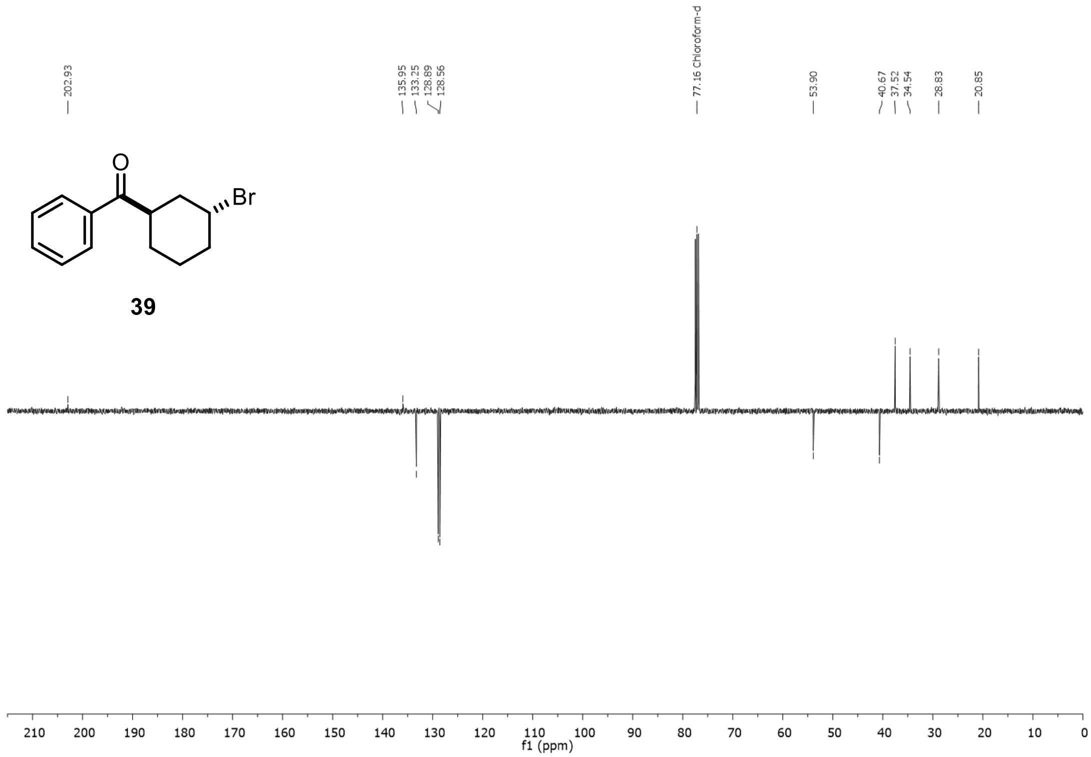

40: *anti*-(3-chlorocyclohexyl)(phenyl)methanone

^{1}H NMR (600 MHz, $CDCl_3$)

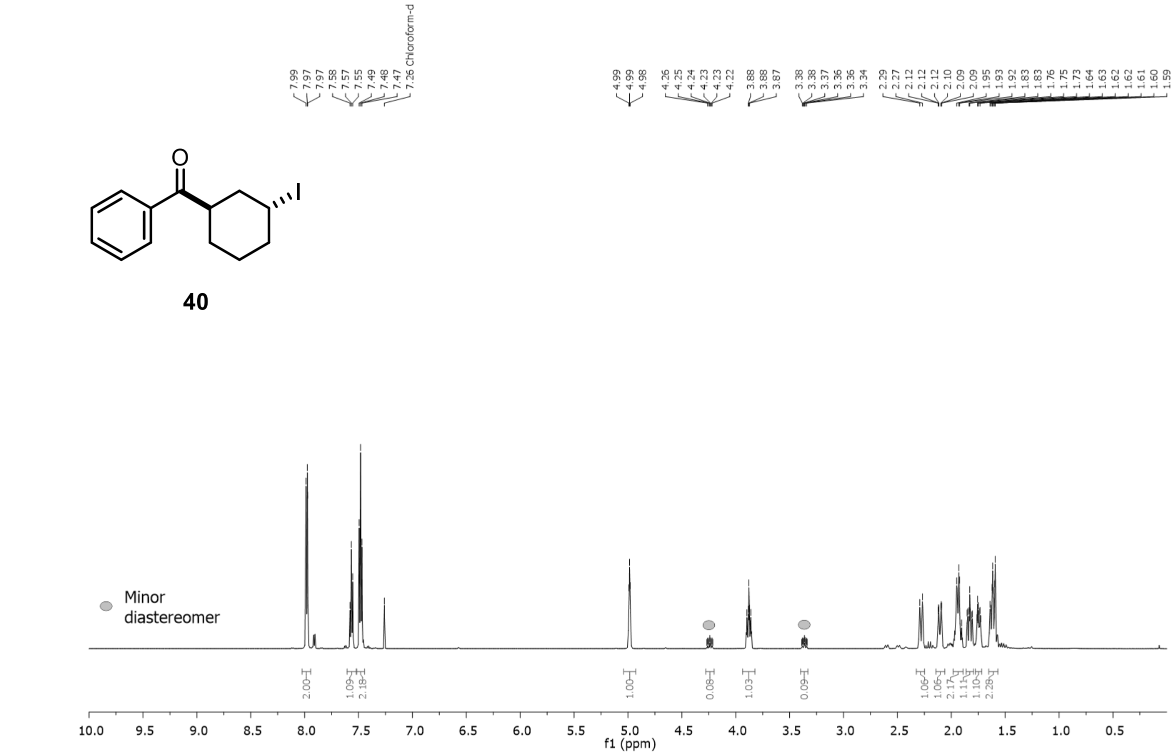

^{13}C NMR (151 MHz, $CDCl_3$)

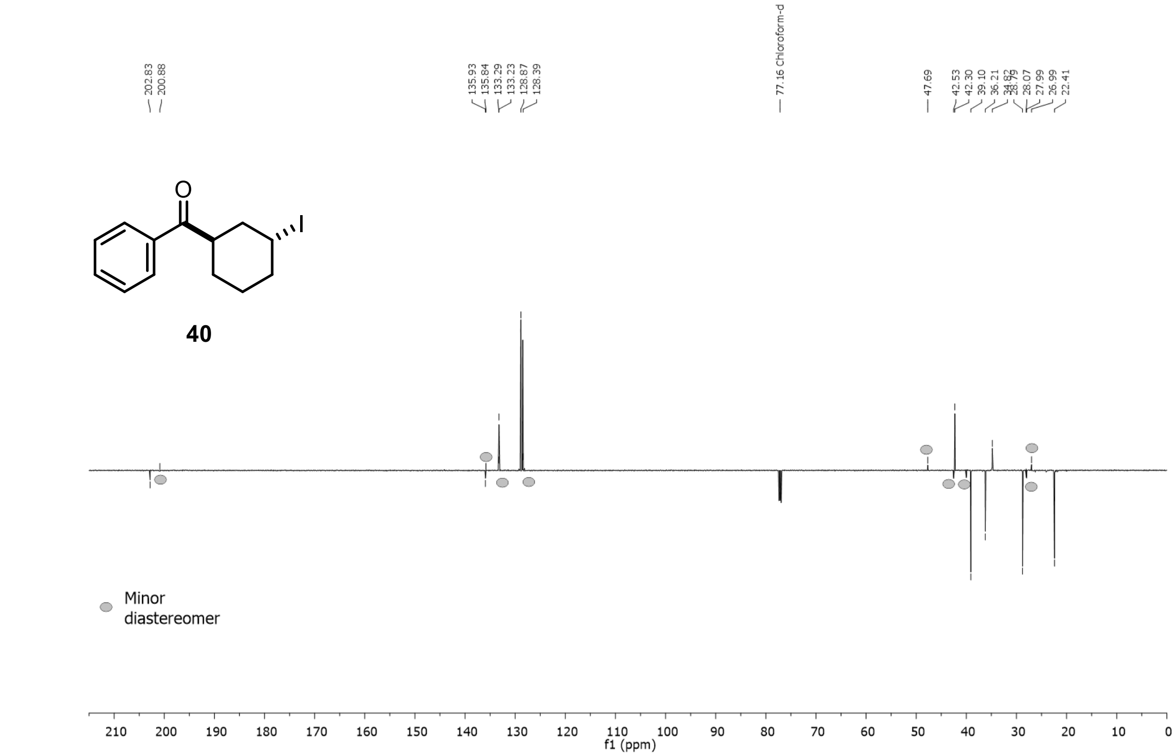

41: *anti*-3-benzoylcyclohexyl acetate

^{1}H NMR (600 MHz, $CDCl_3$)

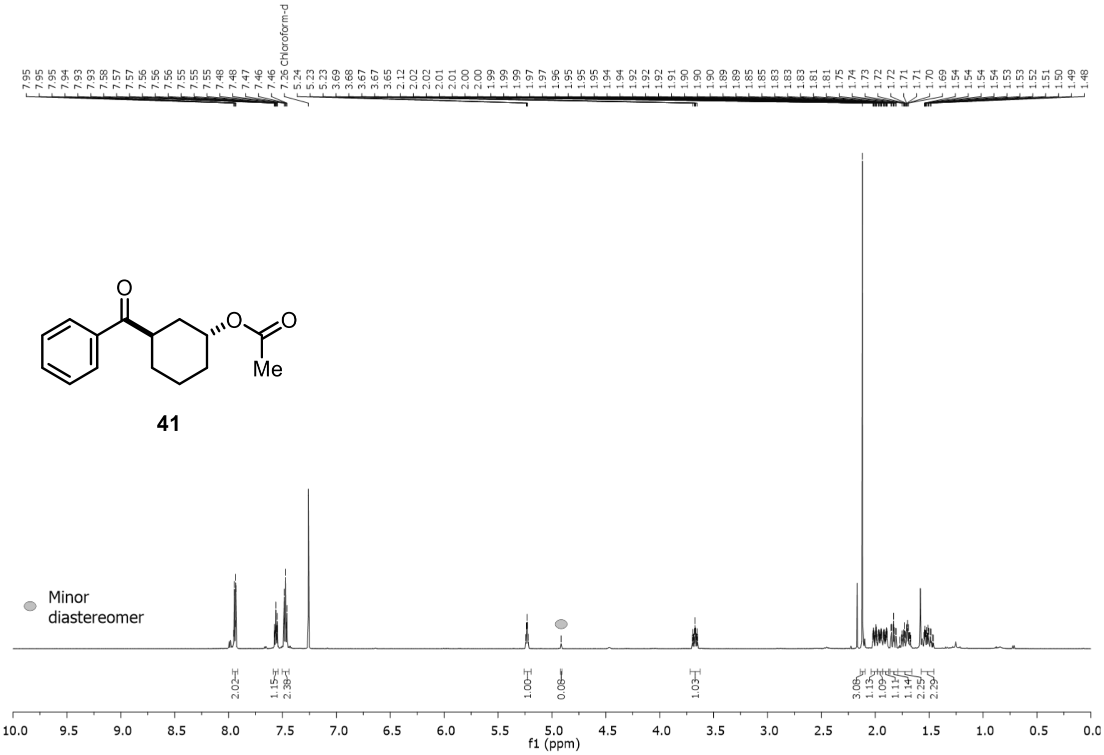

^{13}C NMR (151 MHz, $CDCl_3$)

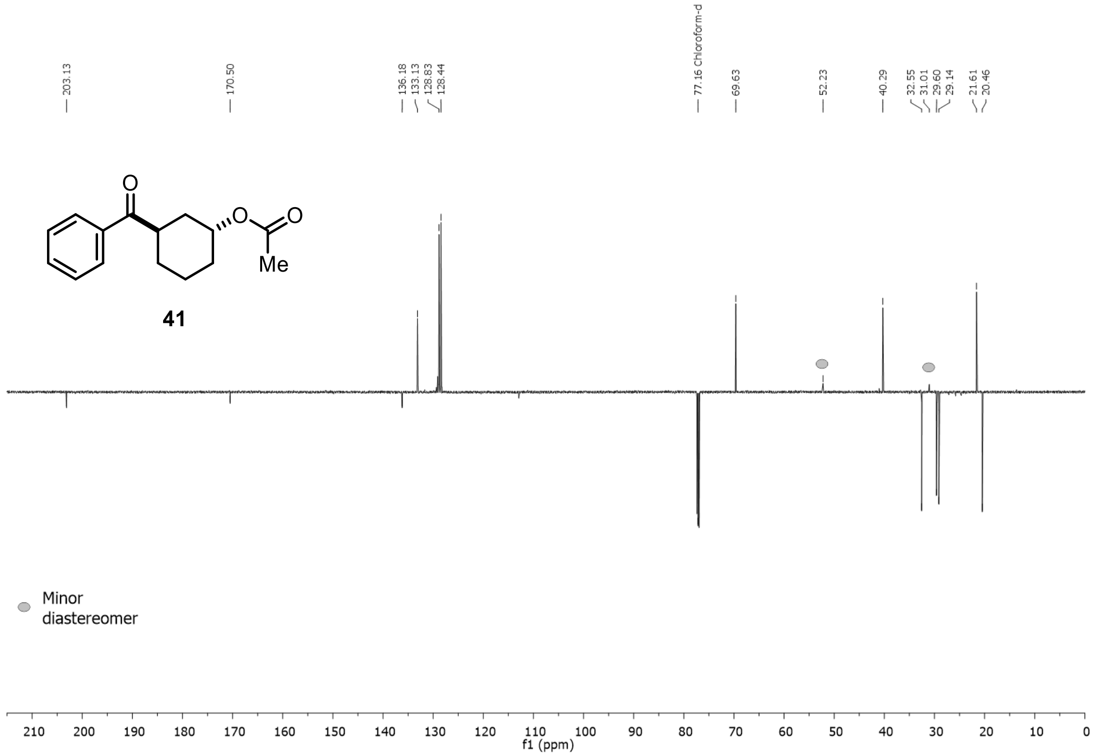

42: *anti*-3-benzoylcyclohexyl formate

^{1}H NMR (700 MHz, $CDCl_3$)

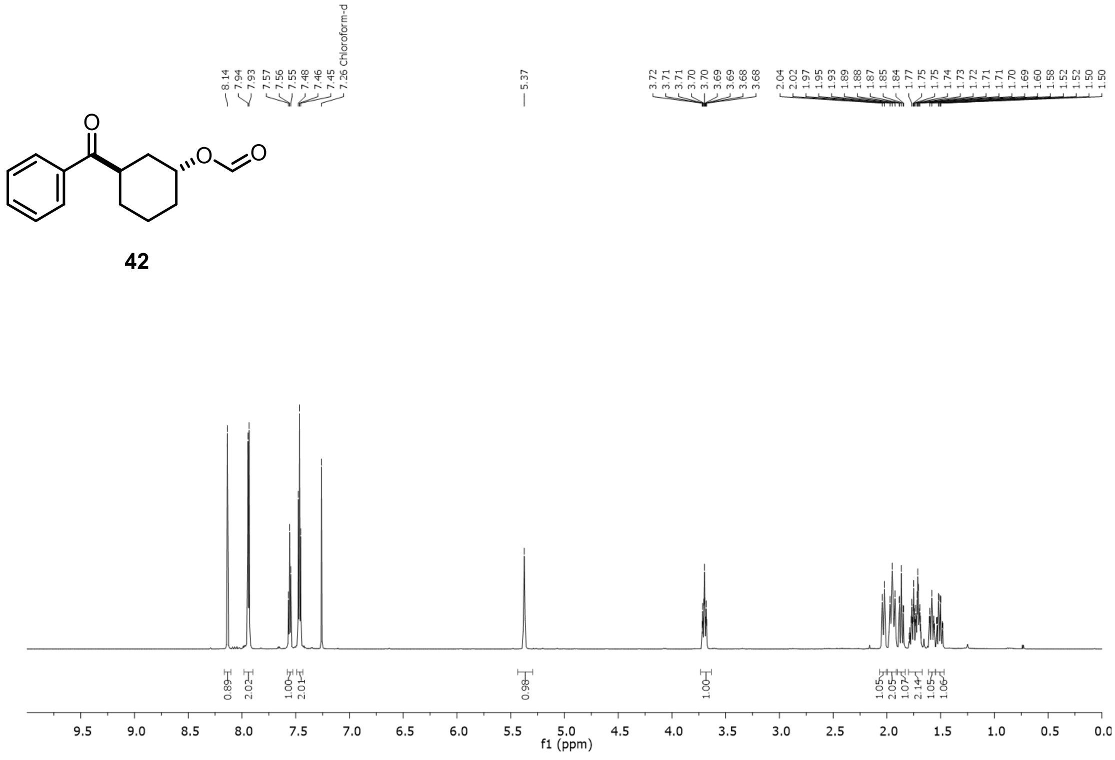

^{13}C NMR (176 MHz, $CDCl_3$)

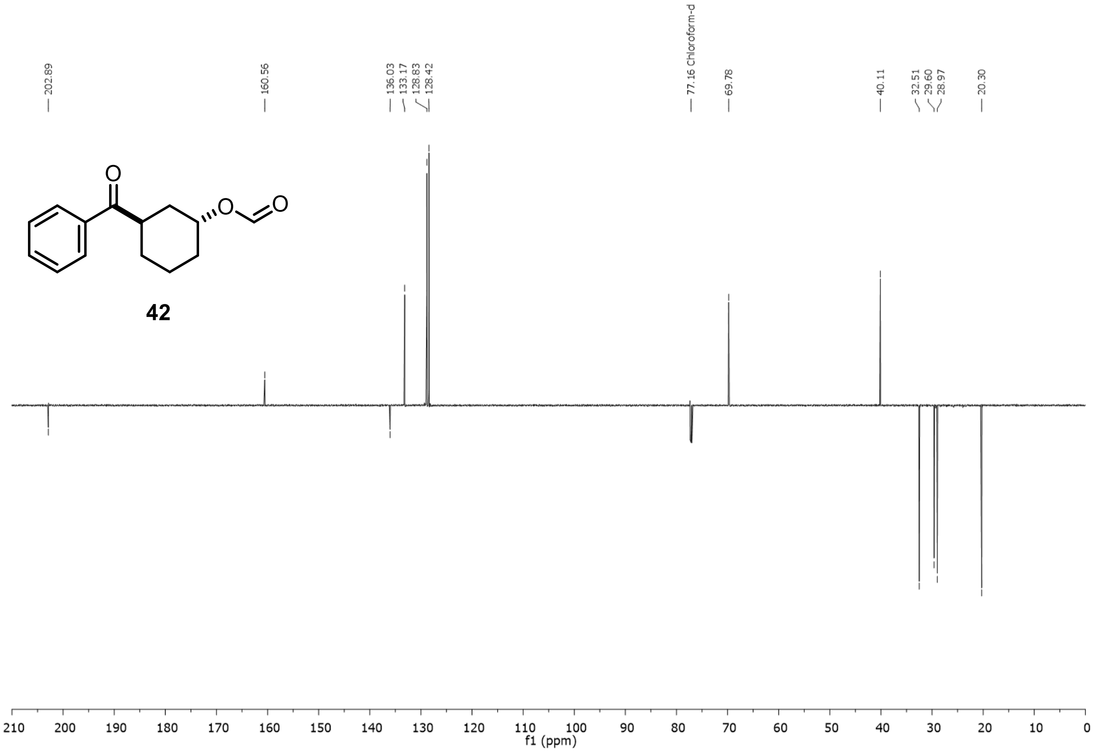

43: *anti*-phenyl(3-((2,2,6,6-tetramethylpiperidin-1-yl)oxy)cyclohexyl)methanone

^{1}H NMR (400 MHz, $CDCl_3$)

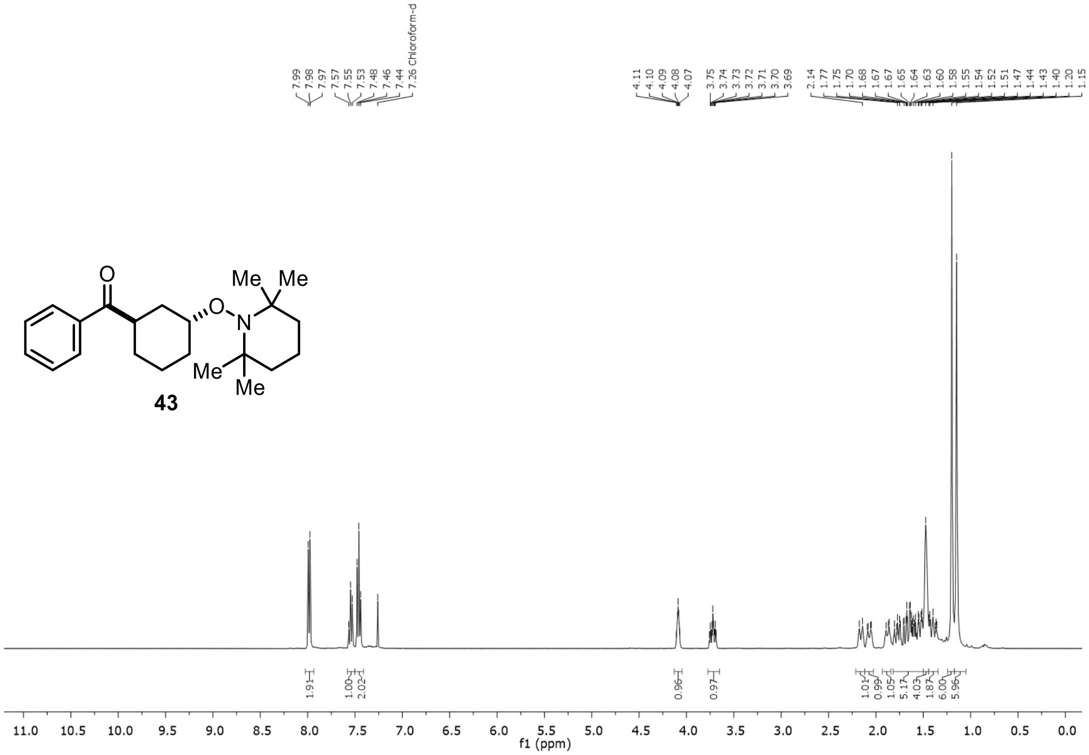

^{13}C NMR (101 MHz, $CDCl_3$)

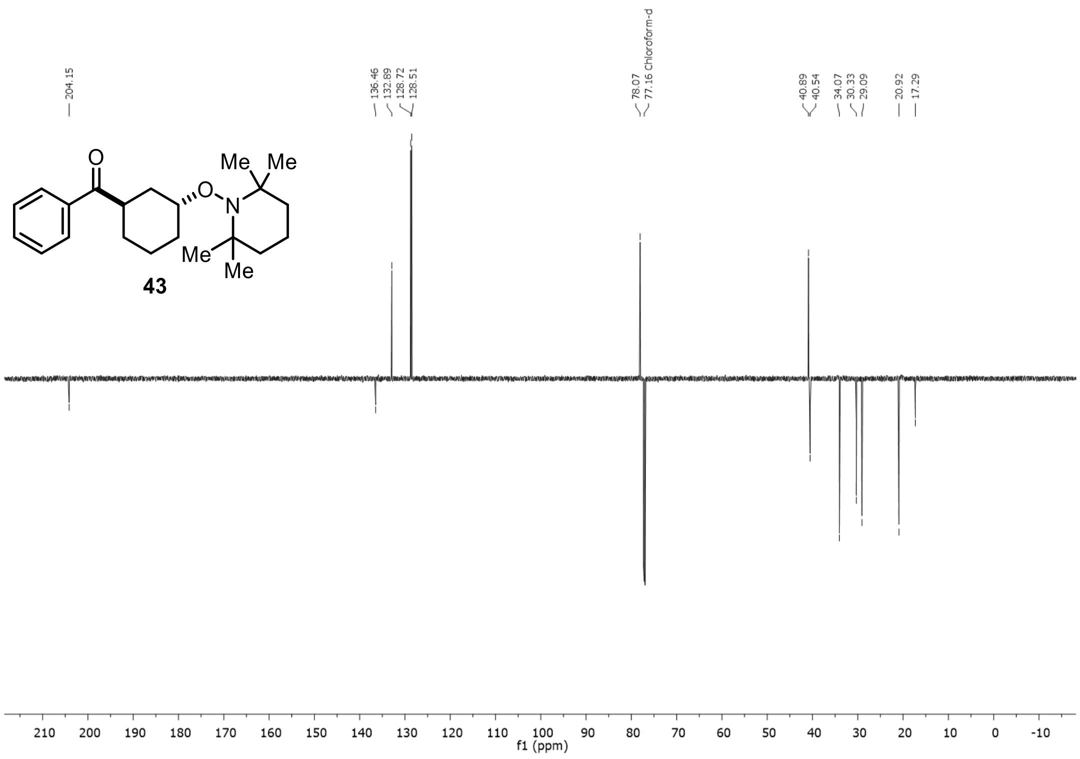

HSQC (^{1}H NMR (700 MHz, $CDCl_3$) ^{13}C NMR (176 MHz, $CDCl_3$))

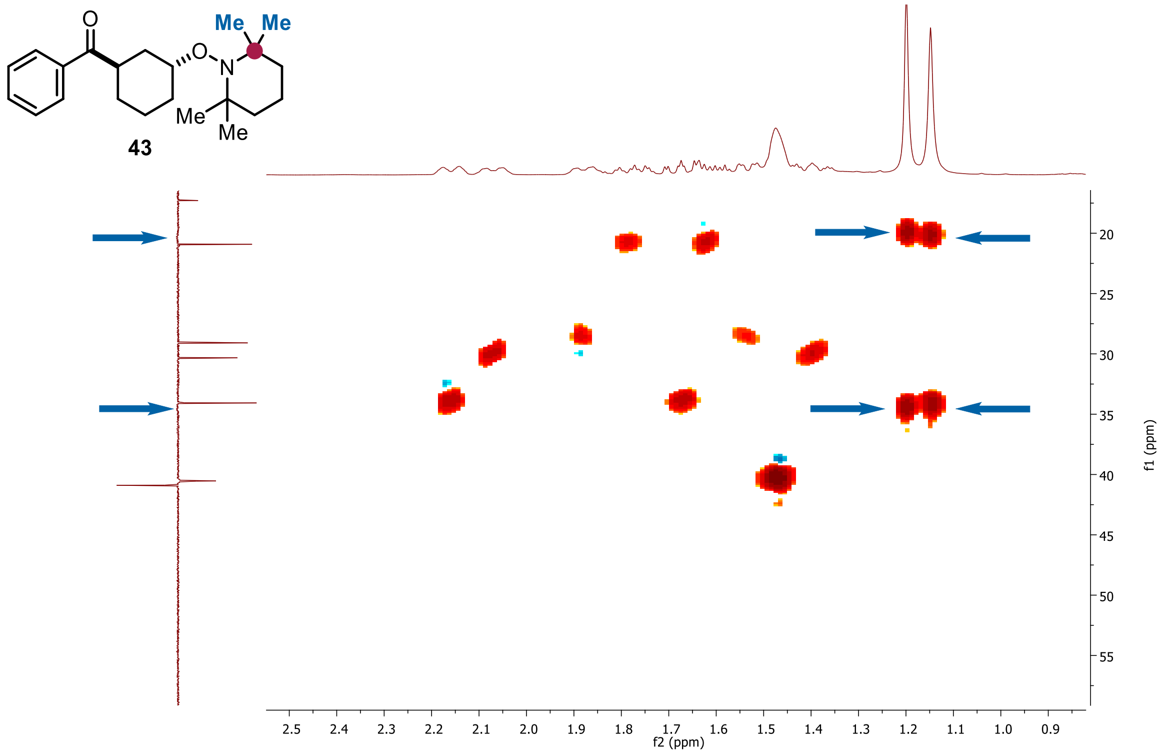

HMBC (^{1}H NMR (700 MHz, $CDCl_3$) ^{13}C NMR (176 MHz, $CDCl_3$))

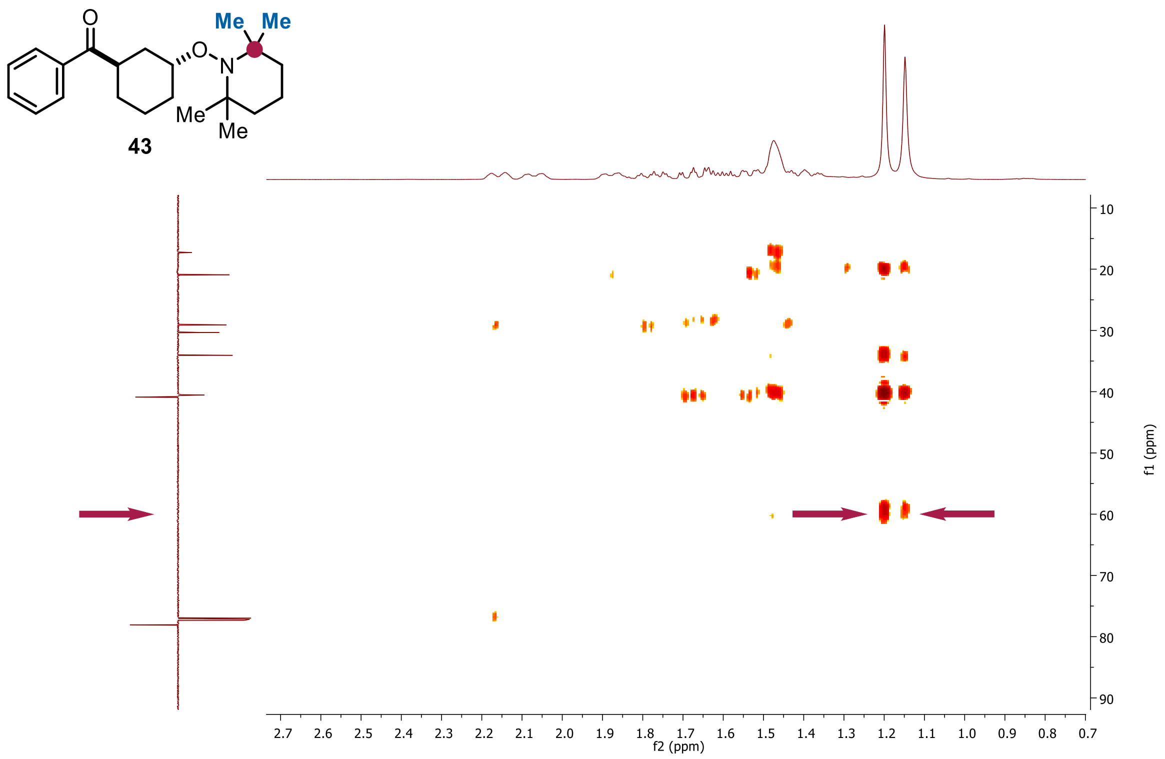

44: 3-benzoylcyclohexan-1-one

^{1}H NMR (400 MHz, $CDCl_3$)

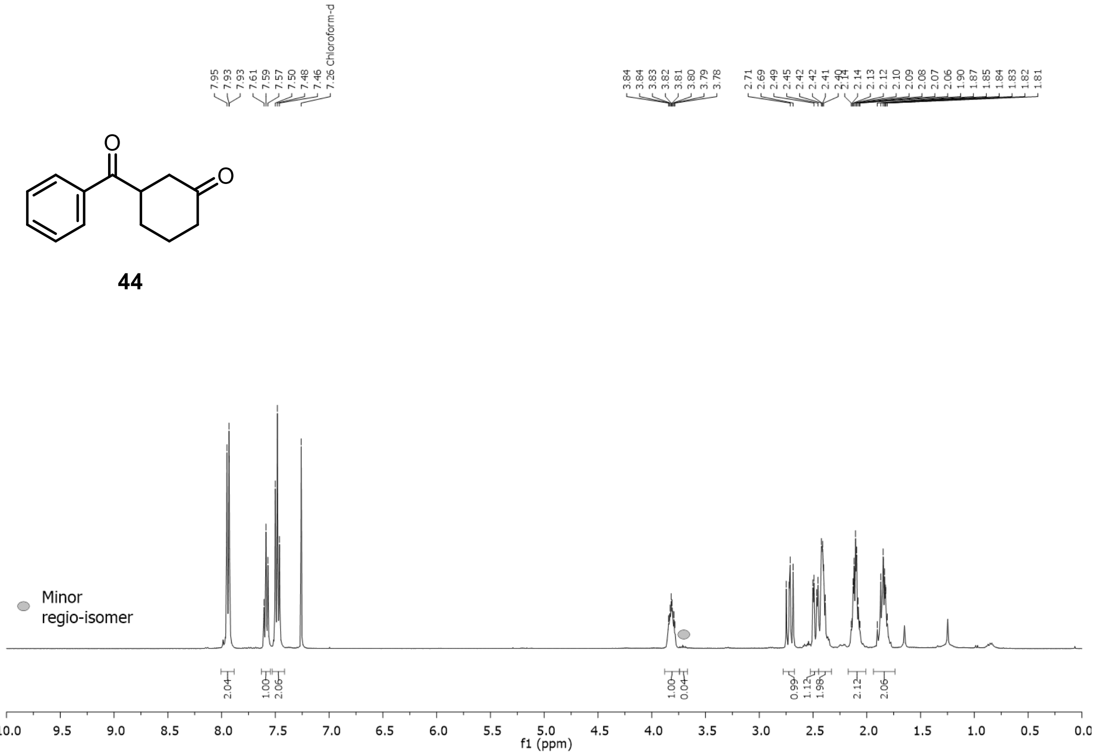

^{13}C NMR (101 MHz, $CDCl_3$)

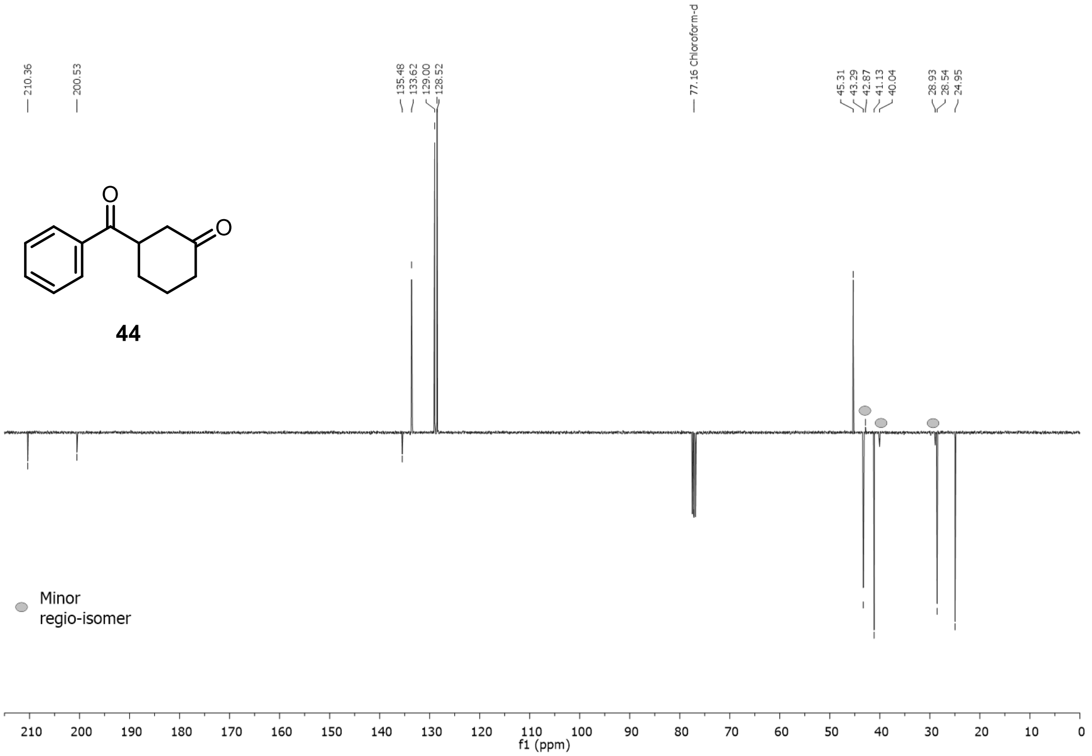

45: 3-(4-(trifluoromethyl)benzoyl)cyclohexan-1-one

^{1}H NMR (400 MHz, $CDCl_3$)

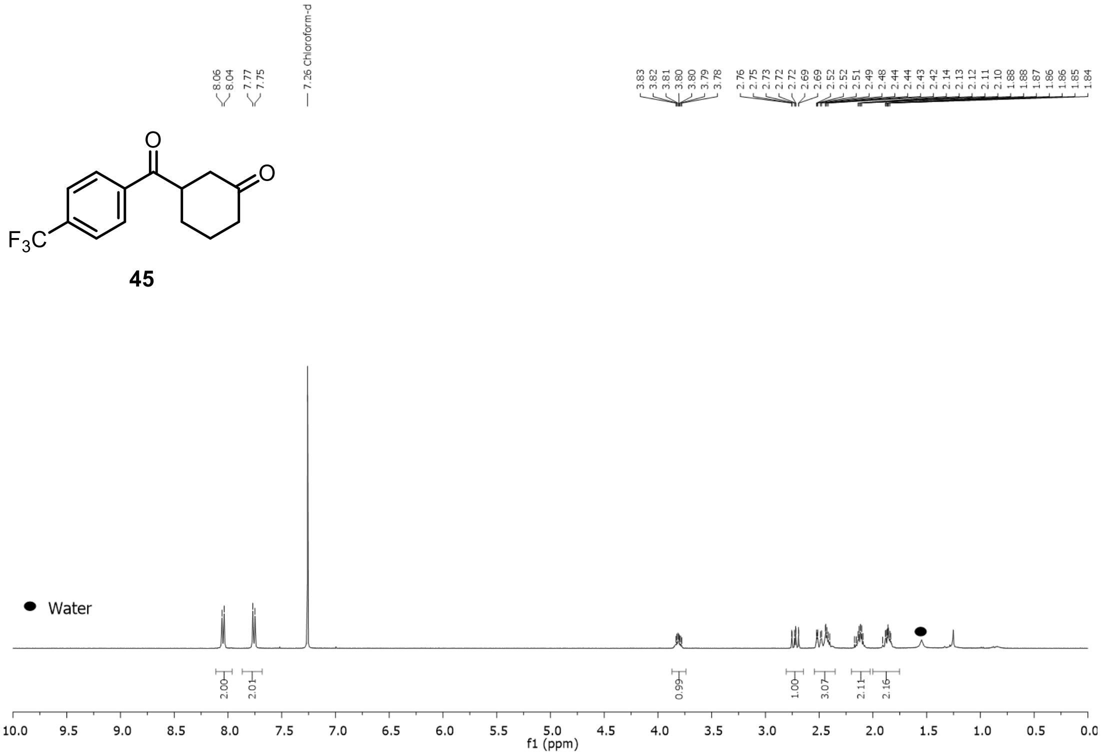

^{13}C NMR (101 MHz, $CDCl_3$)

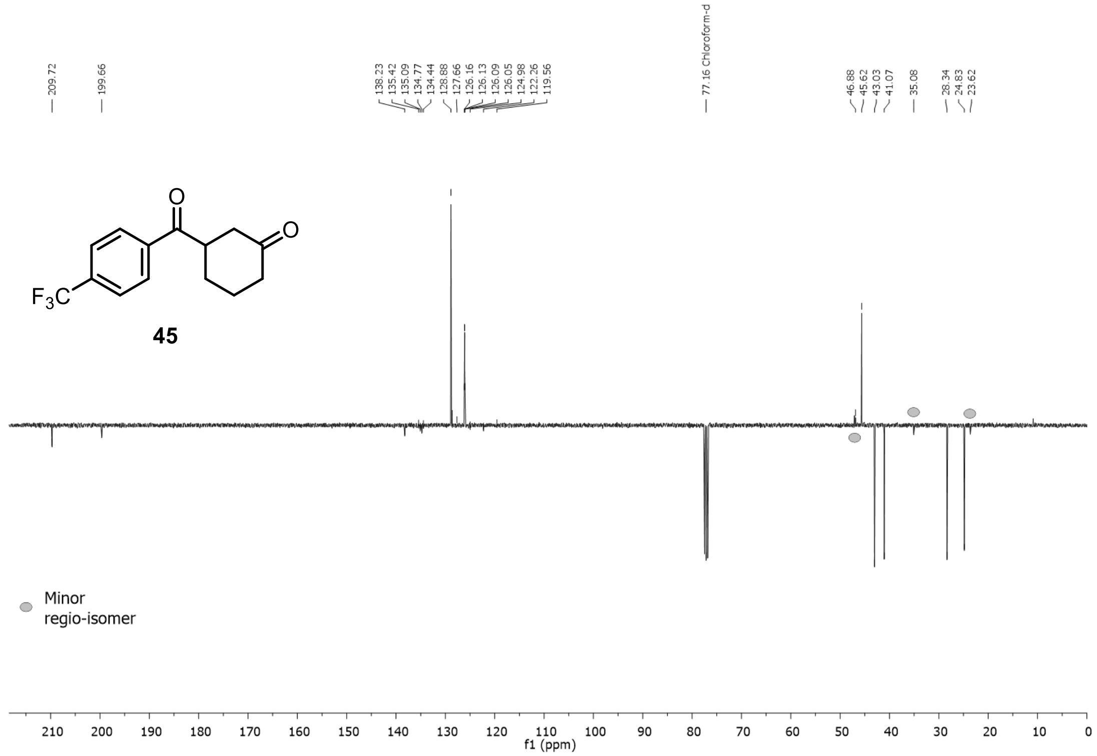

^{19}F NMR (377 MHz, $CDCl_3$)

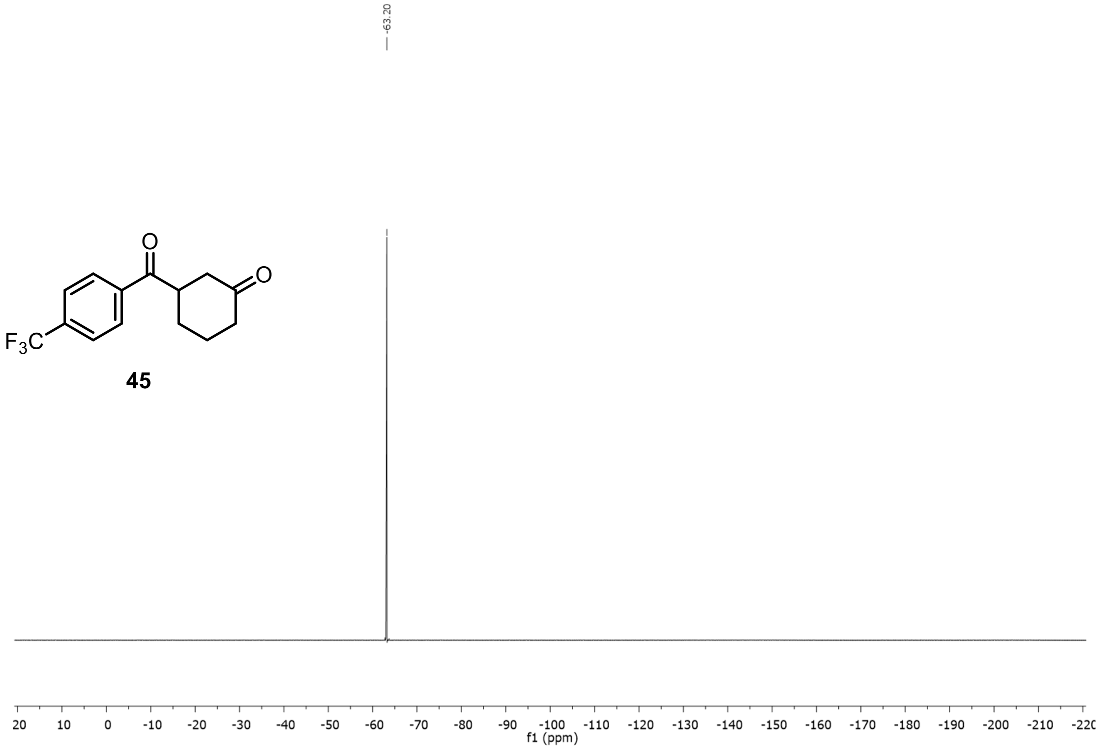

46: 3-benzoylcyclopentan-1-one

^{1}H NMR (400 MHz, $CDCl_3$)

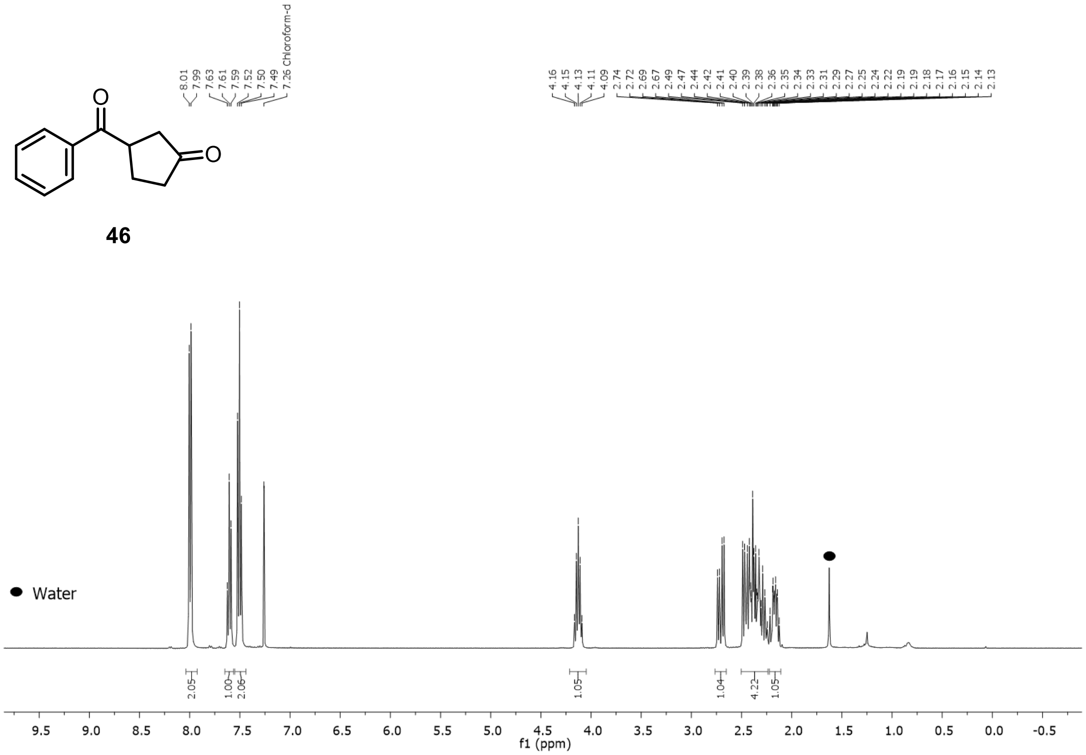

^{13}C NMR (101 MHz, $CDCl_3$)

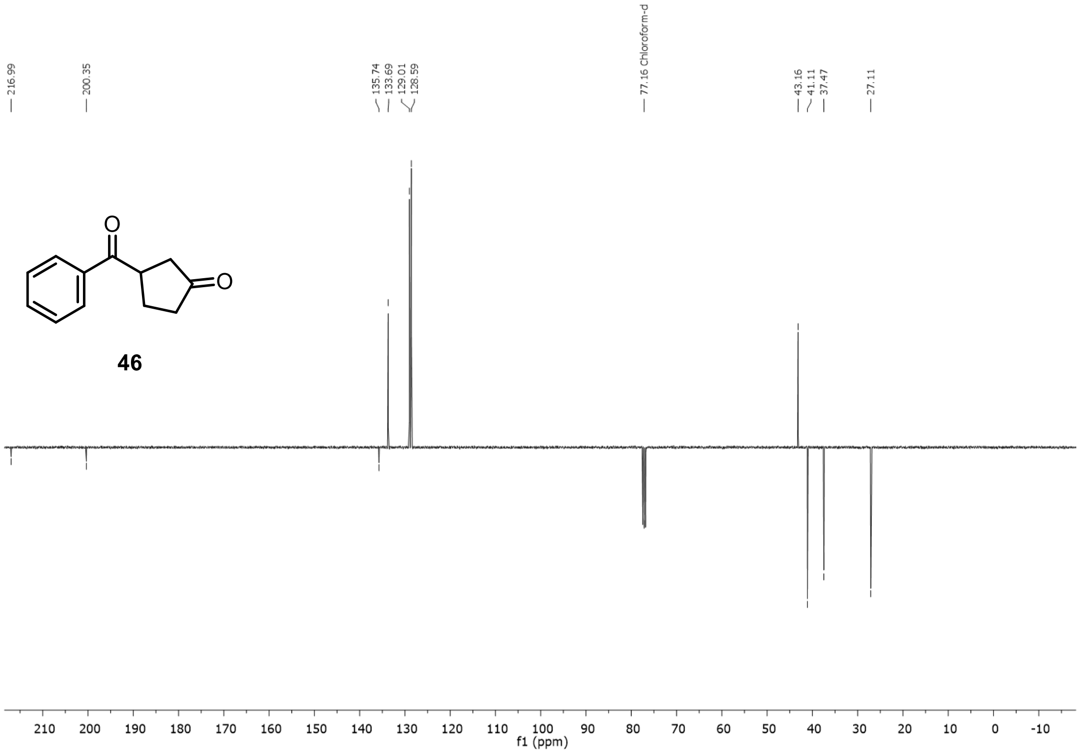

47: 3-(thiophene-2-carbonyl)cyclohexan-1-one

^{1}H NMR (400 MHz, $CDCl_3$)

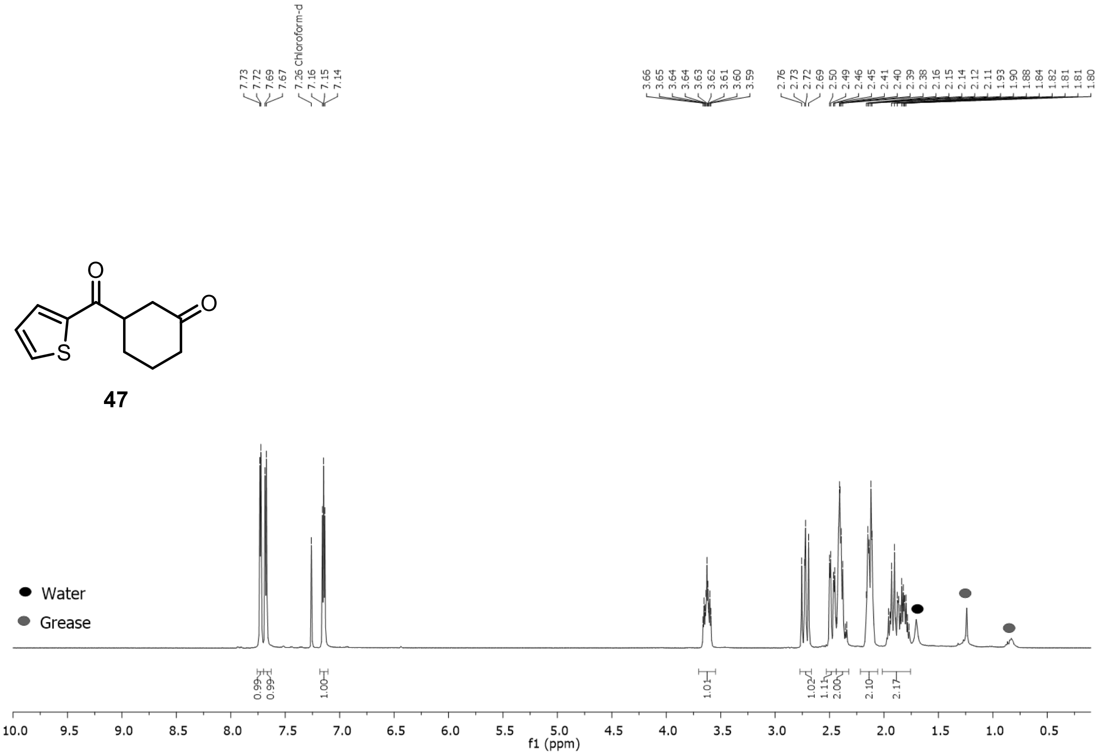

^{13}C NMR (126 MHz, $CDCl_3$)

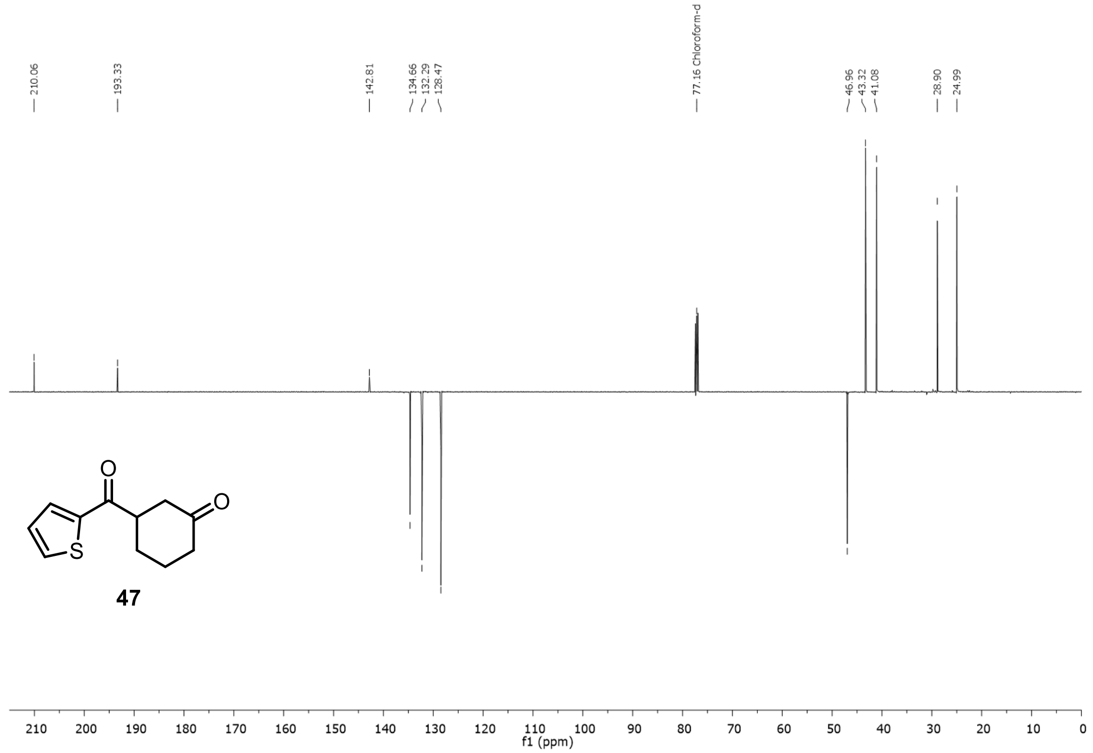

48: 3-pivaloylcyclohexan-1-one

^{1}H NMR (400 MHz, $CDCl_3$)

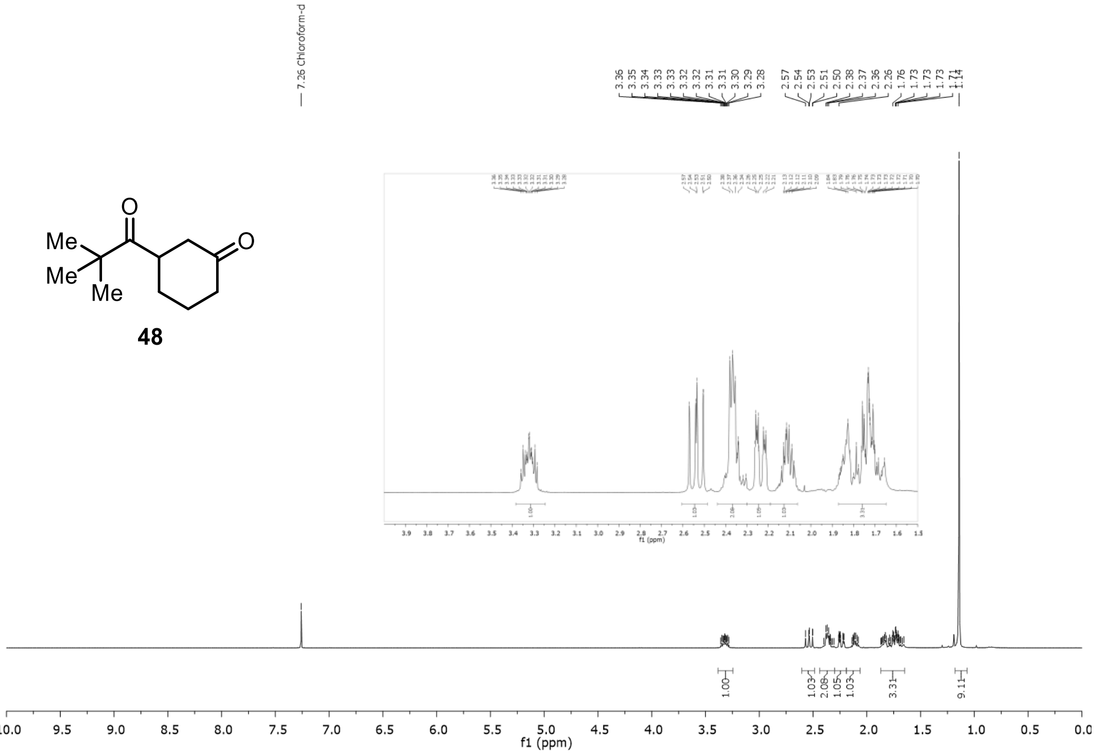

^{13}C NMR (101 MHz, $CDCl_3$)

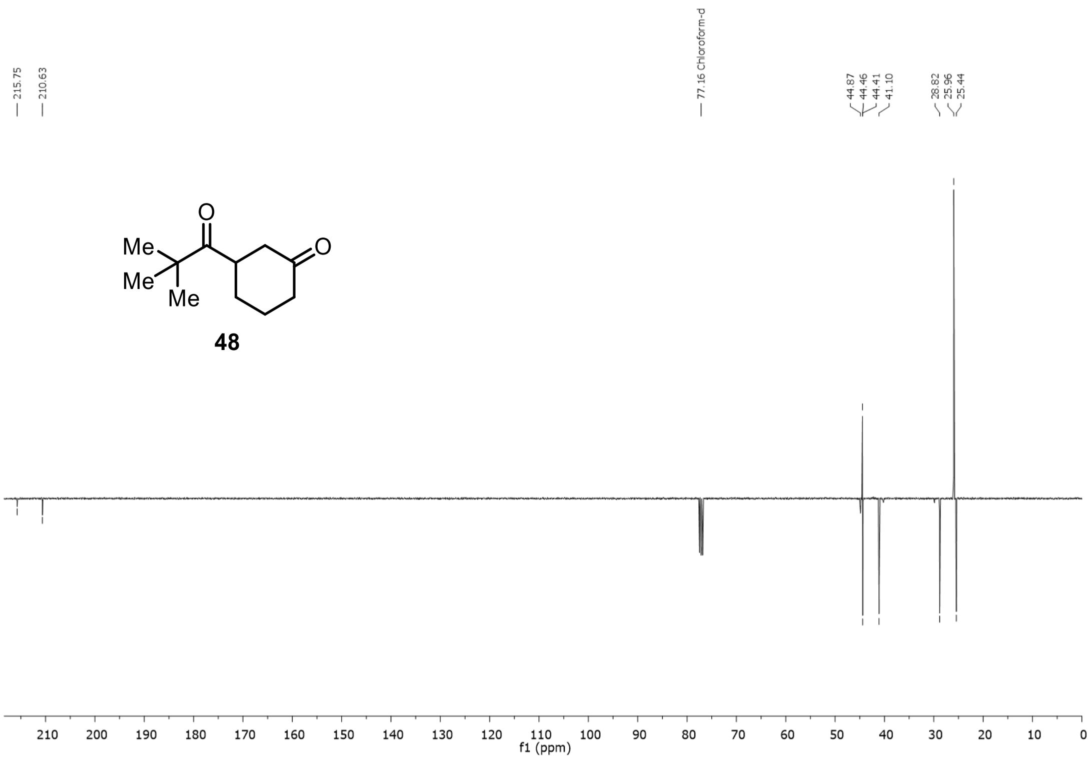

49: 3-(3-methylbenzoyl)cyclopentan-1-one

^{1}H NMR (400 MHz, $CDCl_3$)

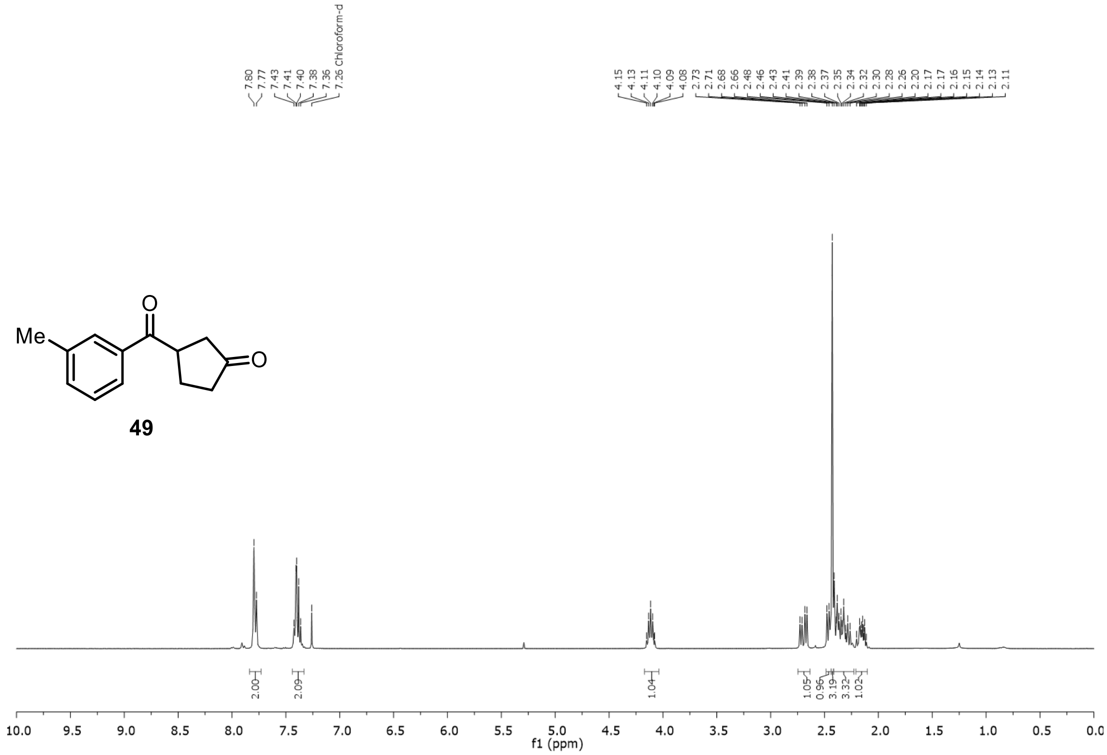

^{13}C NMR (101 MHz, $CDCl_3$)

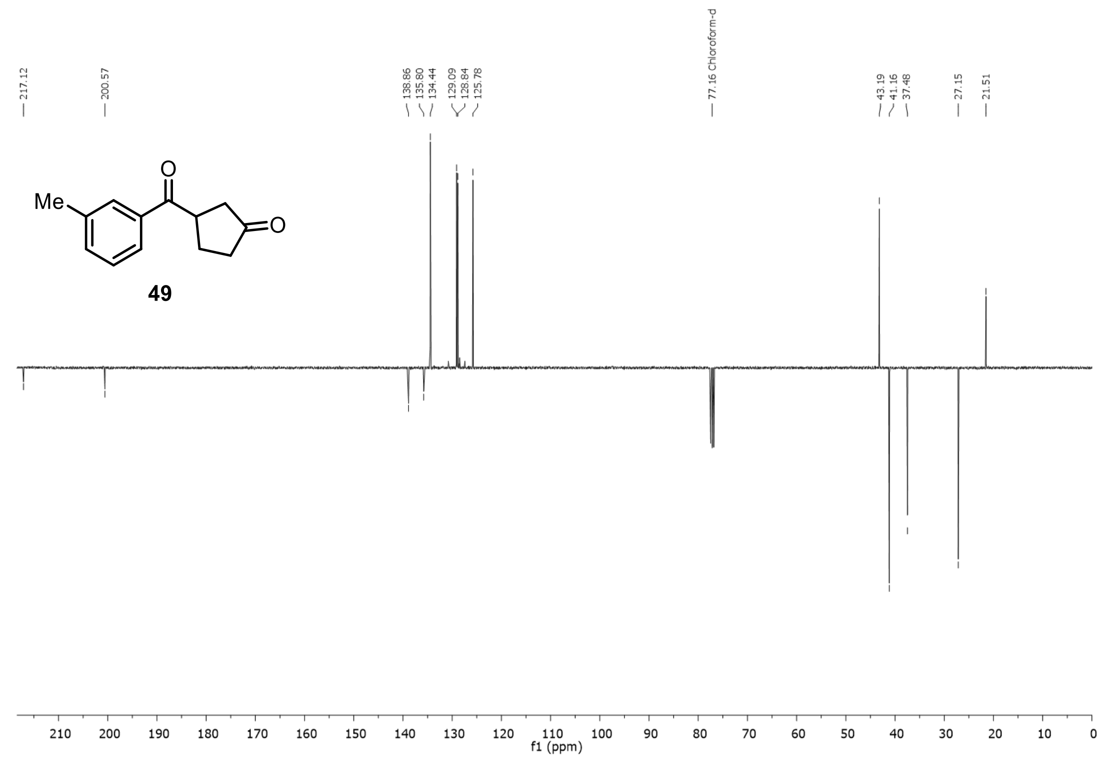

50: 4-Ipomeanol

^{1}H NMR (400 MHz, CDCl$_3$)

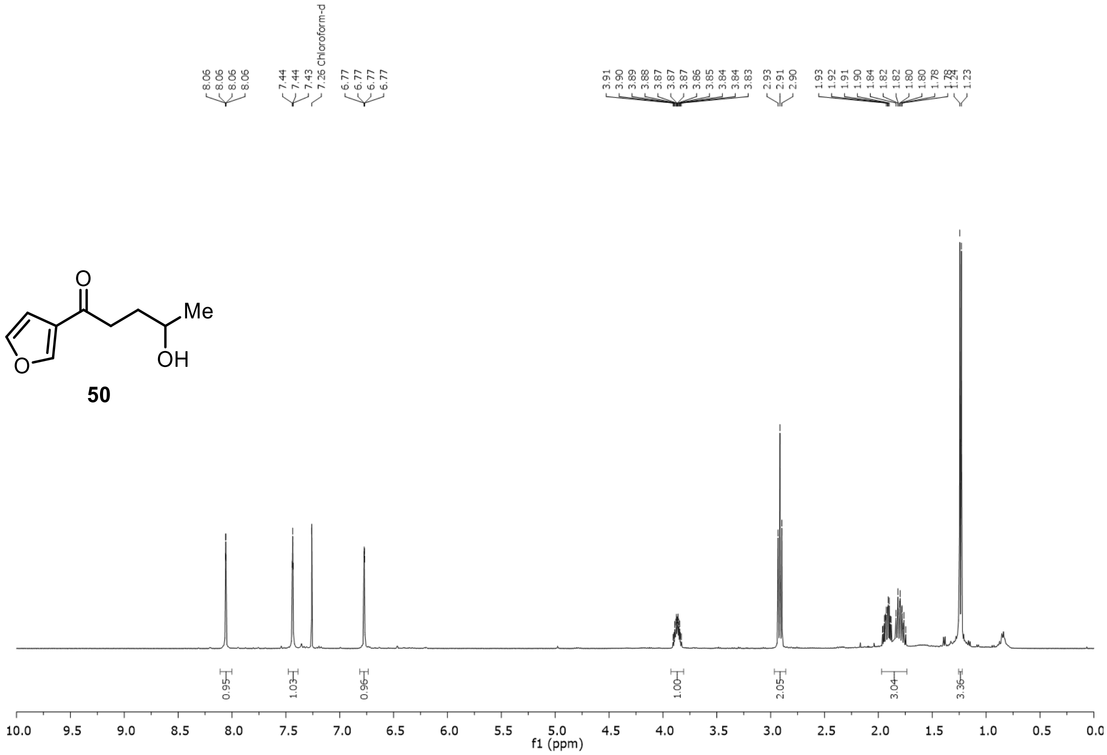

^{13}C NMR (101 MHz, CDCl$_3$)

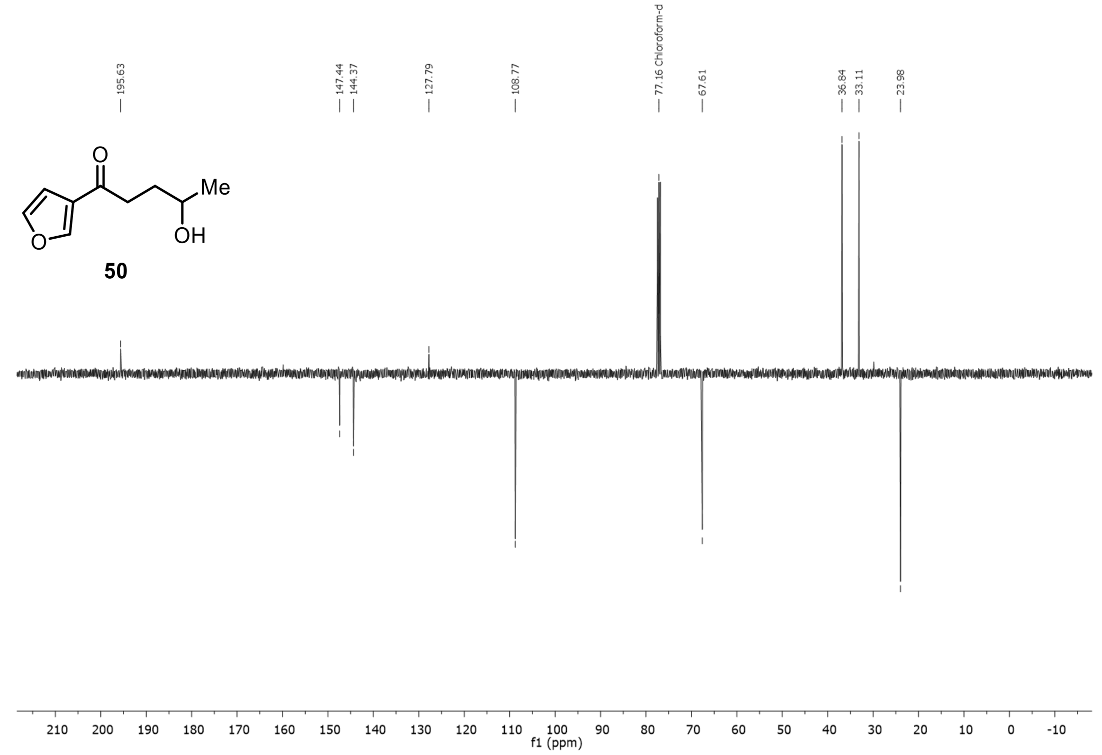

51: 4-hydroxy-1-phenylpentan-1-one

^{1}H NMR (400 MHz, $CDCl_3$)

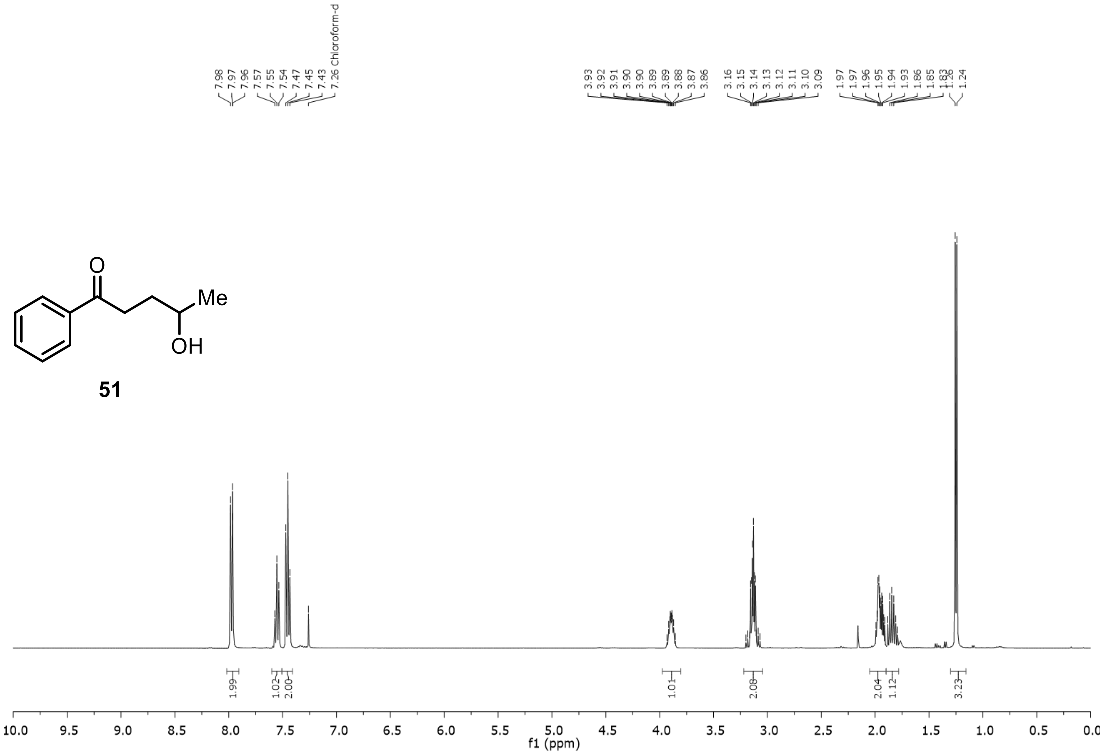

^{13}C NMR (101 MHz, $CDCl_3$)

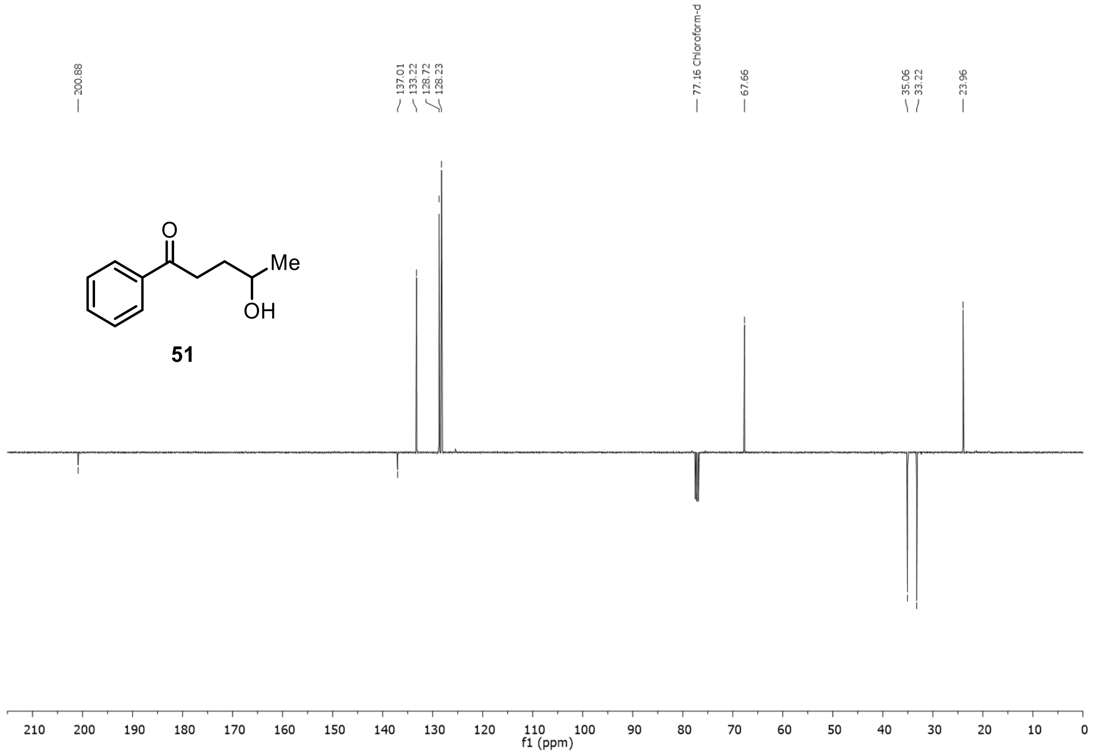

52: 7-hydroxy-2-methyloctan-4-one

^{1}H NMR (400 MHz, $CDCl_3$)

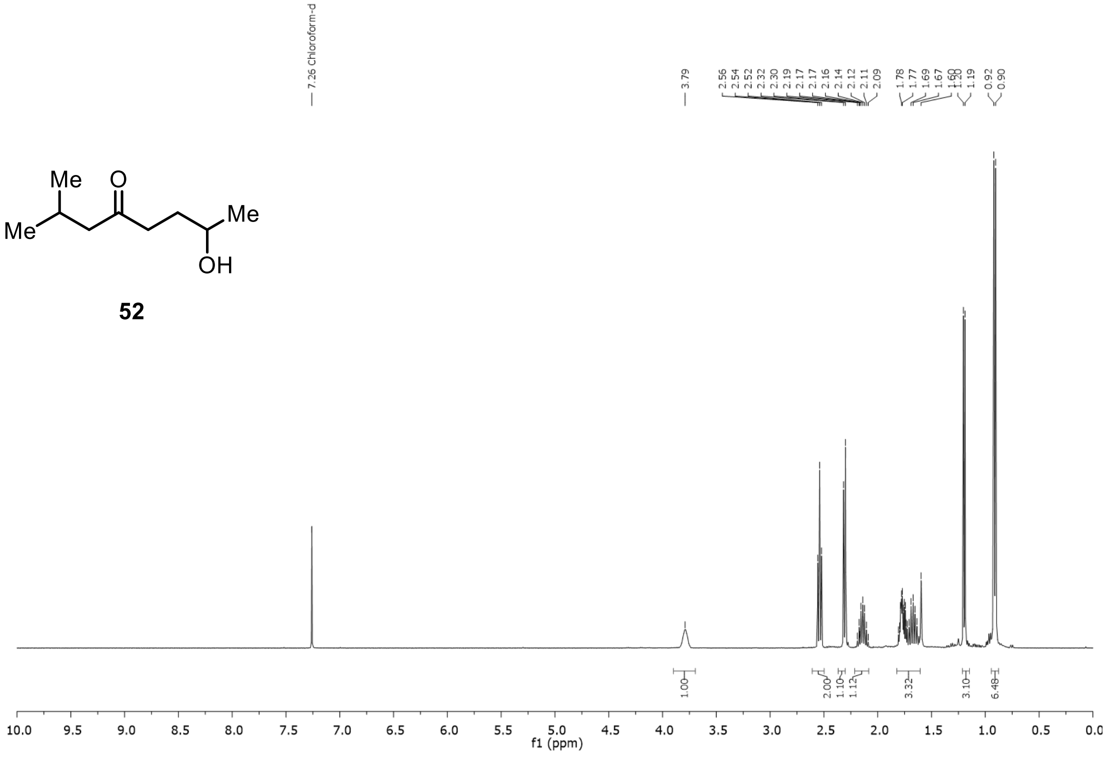

^{13}C NMR (101 MHz, $CDCl_3$)

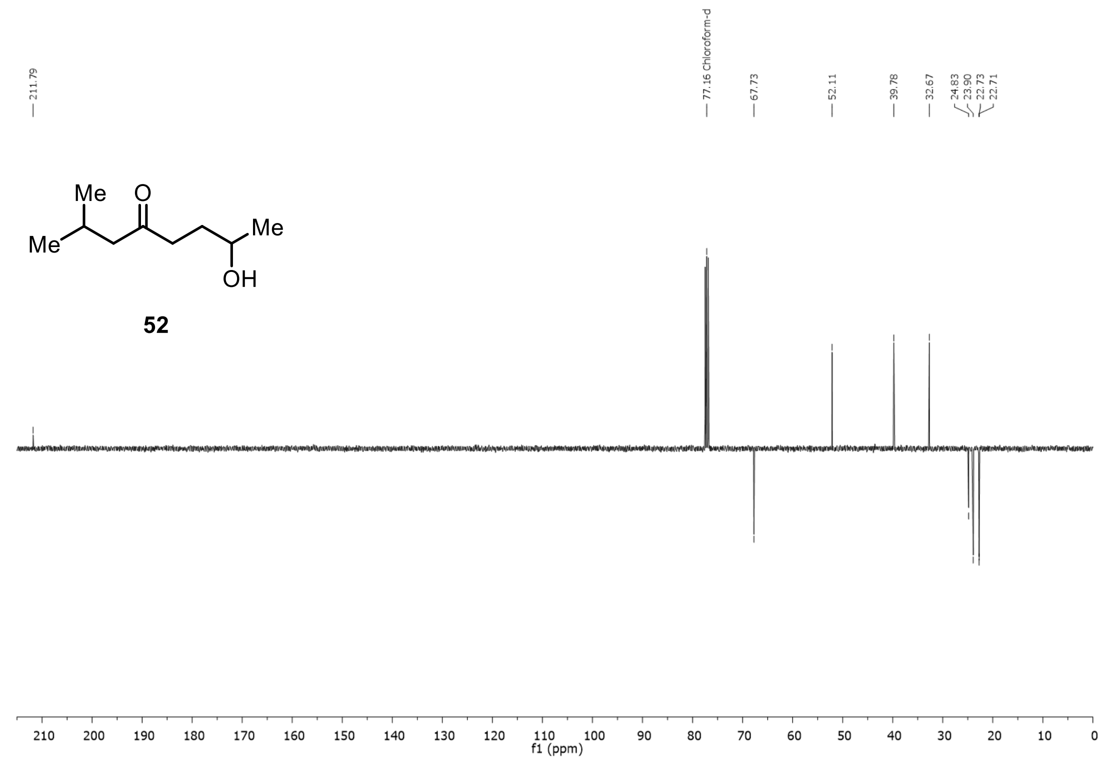

53: 4-hydroxy-1-(thiophen-2-yl)pentan-1-one

^{1}H NMR (400 MHz, $CDCl_3$)

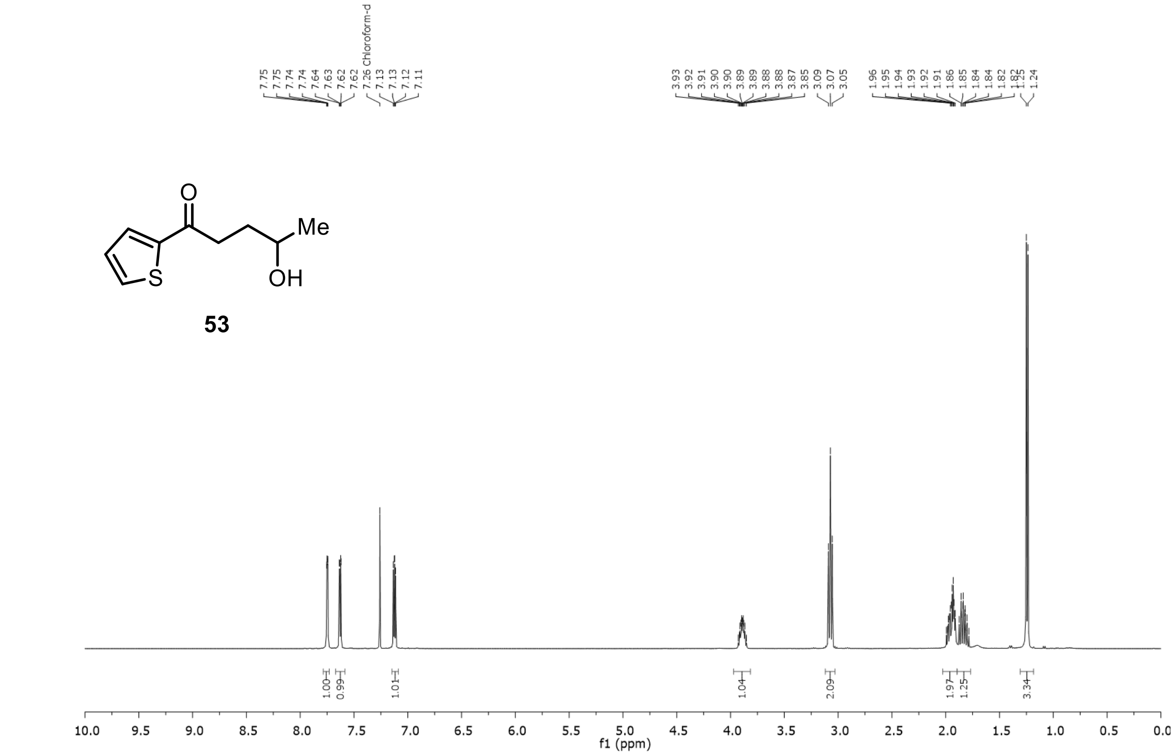

^{13}C NMR (101 MHz, $CDCl_3$)

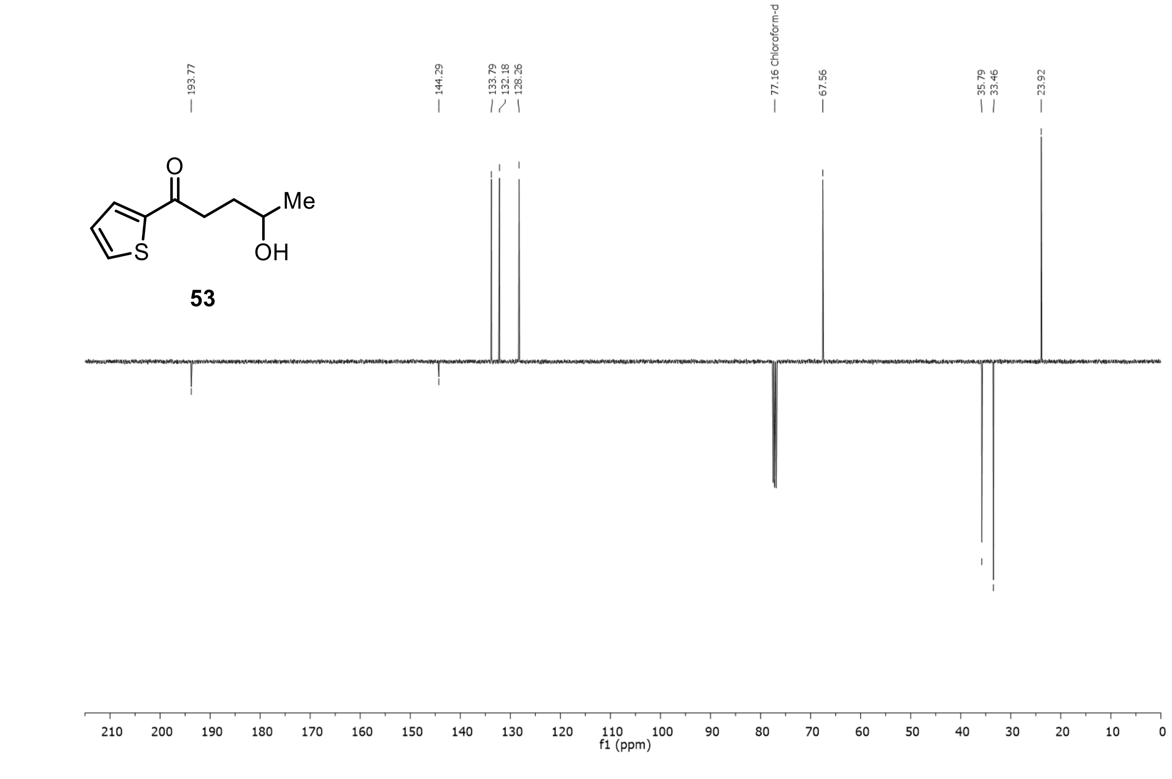

54: ((1*R**,2*R**,3*S**)-3-hydroxy-2-methylcyclohexyl)(phenyl)methanone

^{1}H NMR (400 MHz, $CDCl_3$)

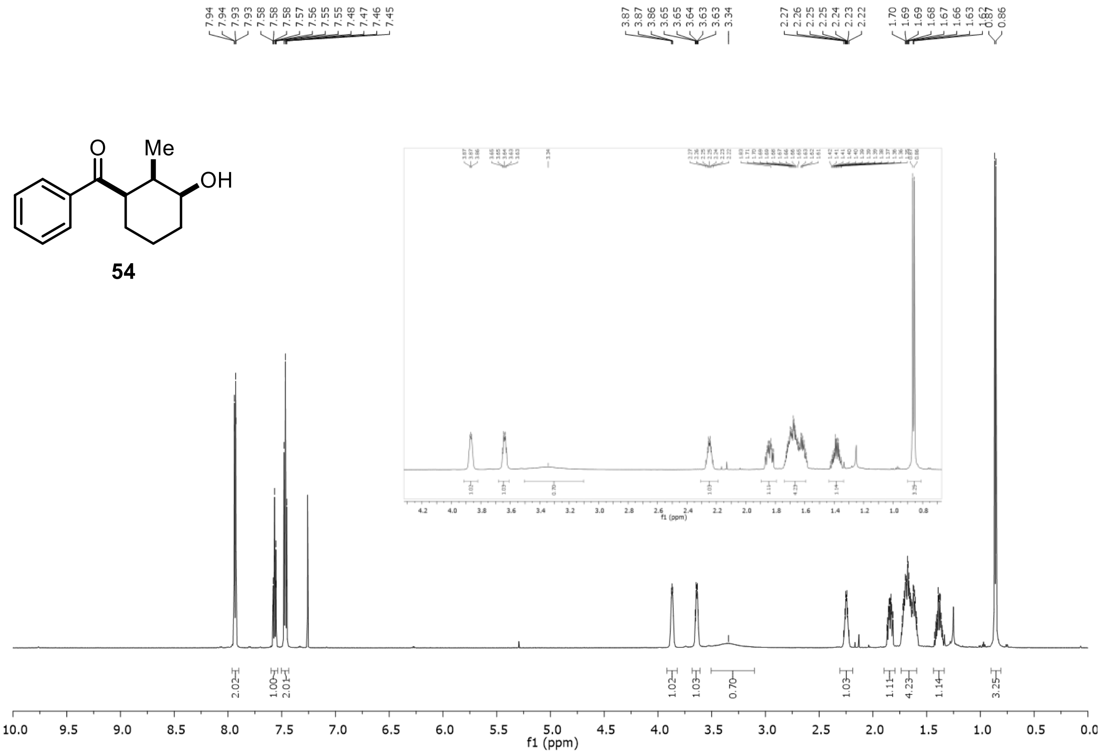

^{13}C NMR (101 MHz, $CDCl_3$)

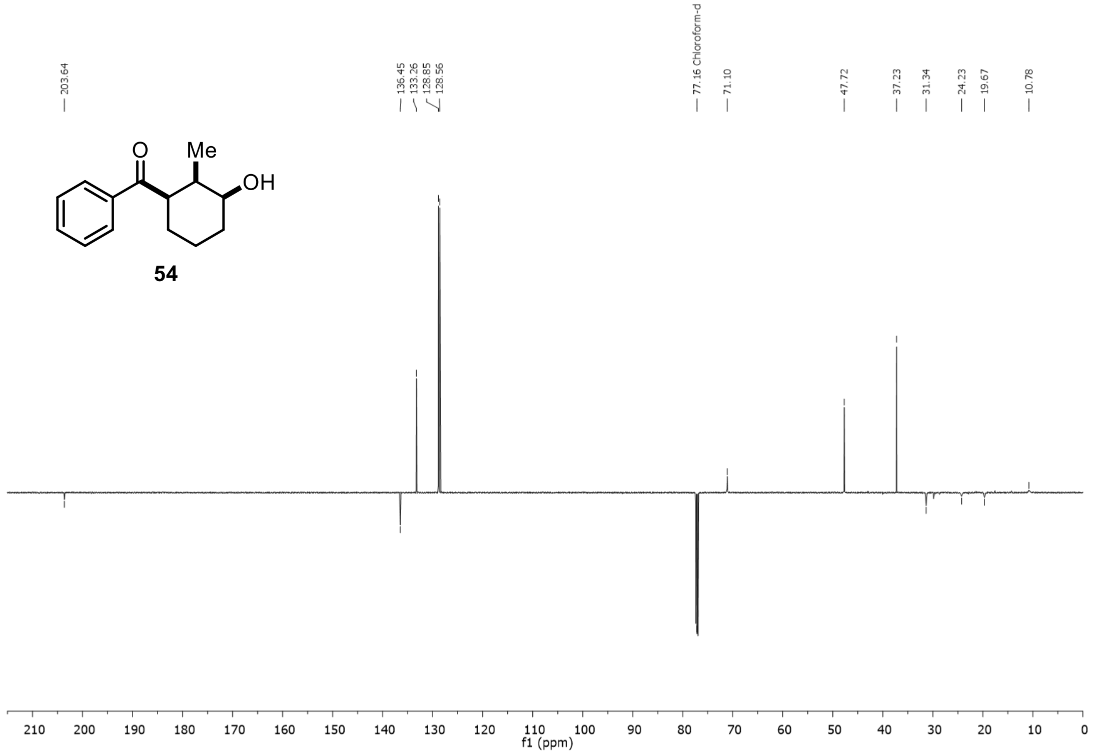

S4: hex-5-en-1-yl 2,2,2-trifluoroacetate

^{1}H NMR (400 MHz, $CDCl_3$)

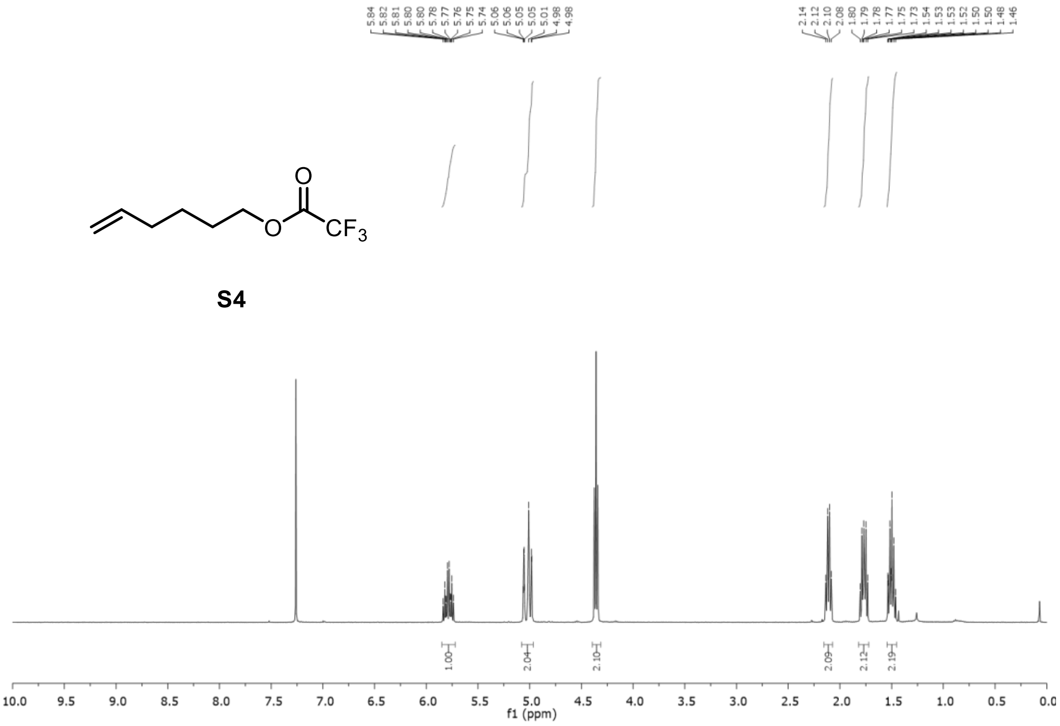

^{13}C NMR (101 MHz, $CDCl_3$)

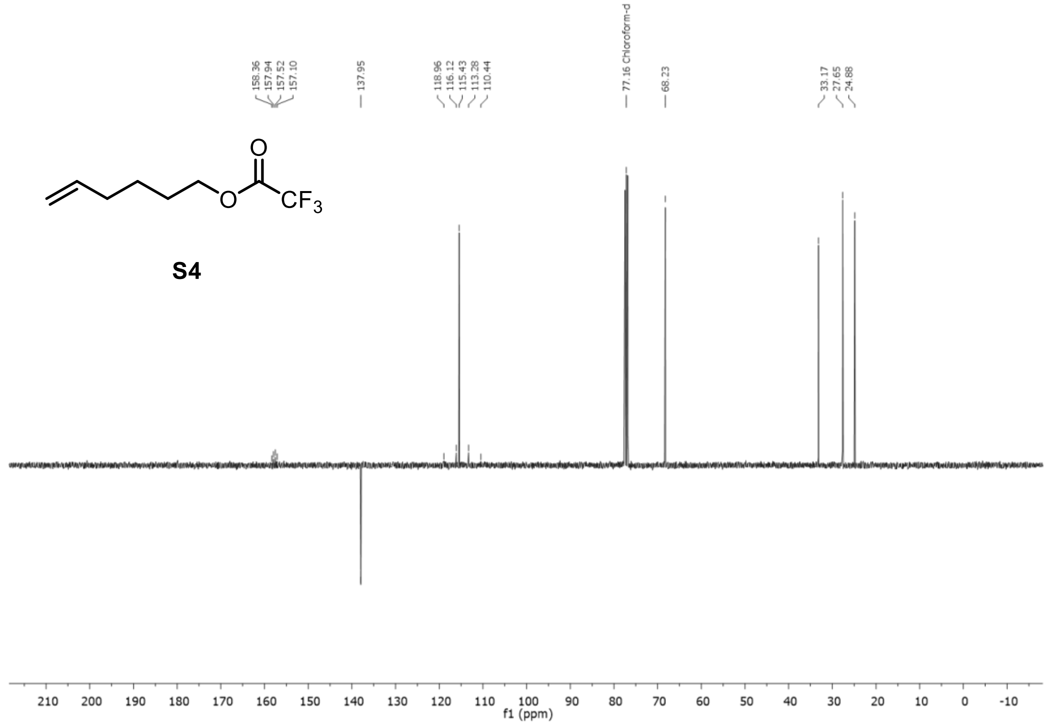

^{19}F NMR (377 MHz, CDCl$_3$)

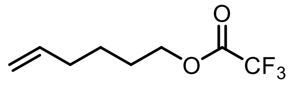

S4

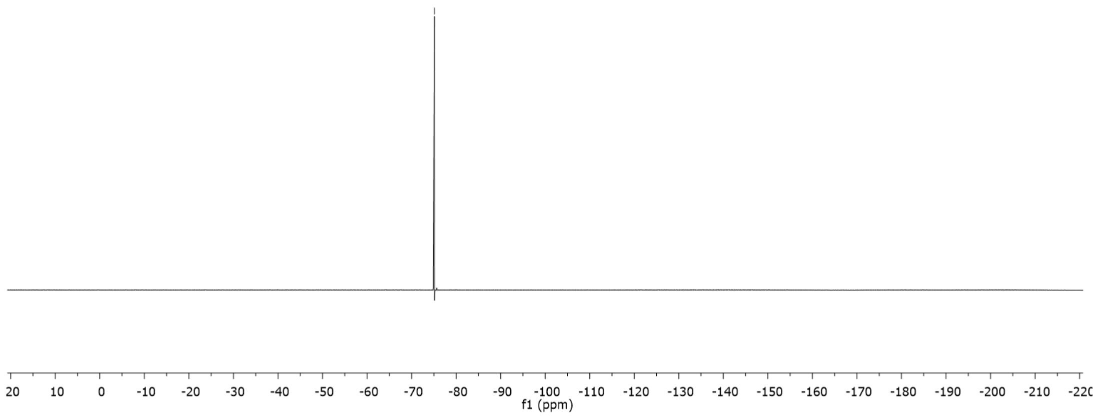

S5: 4,4,5,5-tetramethyl-2-(3-phenyl-3-(4-(trifluoromethyl)phenyl)propyl)-1,3,2-dioxaborolane

^{1}H NMR (400 MHz, $CDCl_3$)

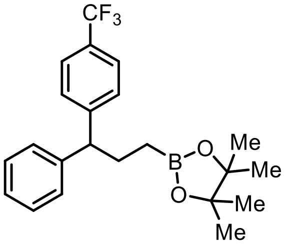

S5

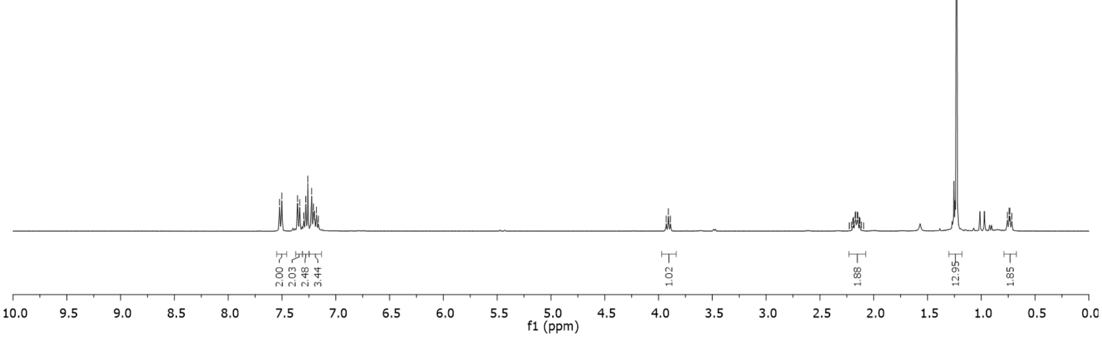

^{13}C NMR (101 MHz, $CDCl_3$)

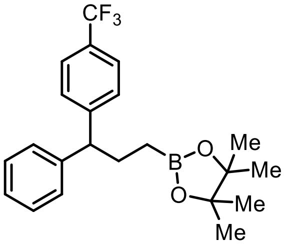

S5

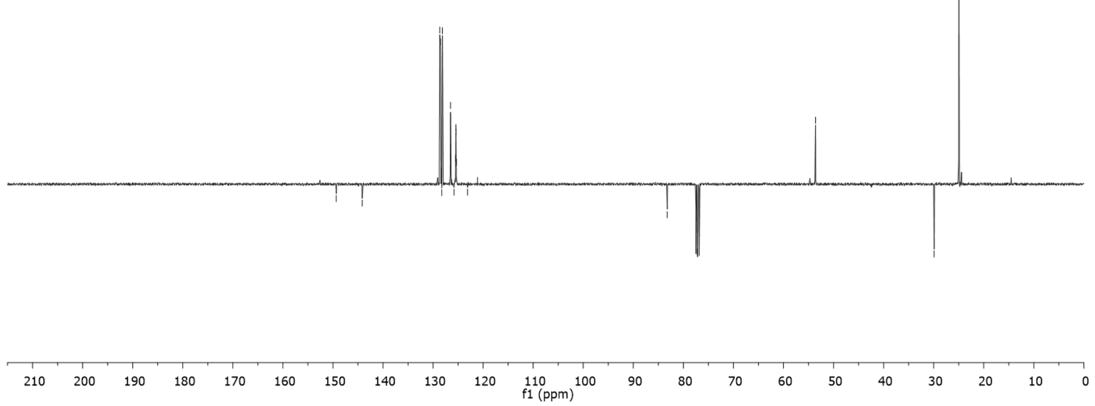

S6: (*E*)-4,4,5,5-tetramethyl-2-(non-1-en-1-yl)-1,3,2-dioxaborolane

^{1}H NMR (400 MHz, $CDCl_3$)

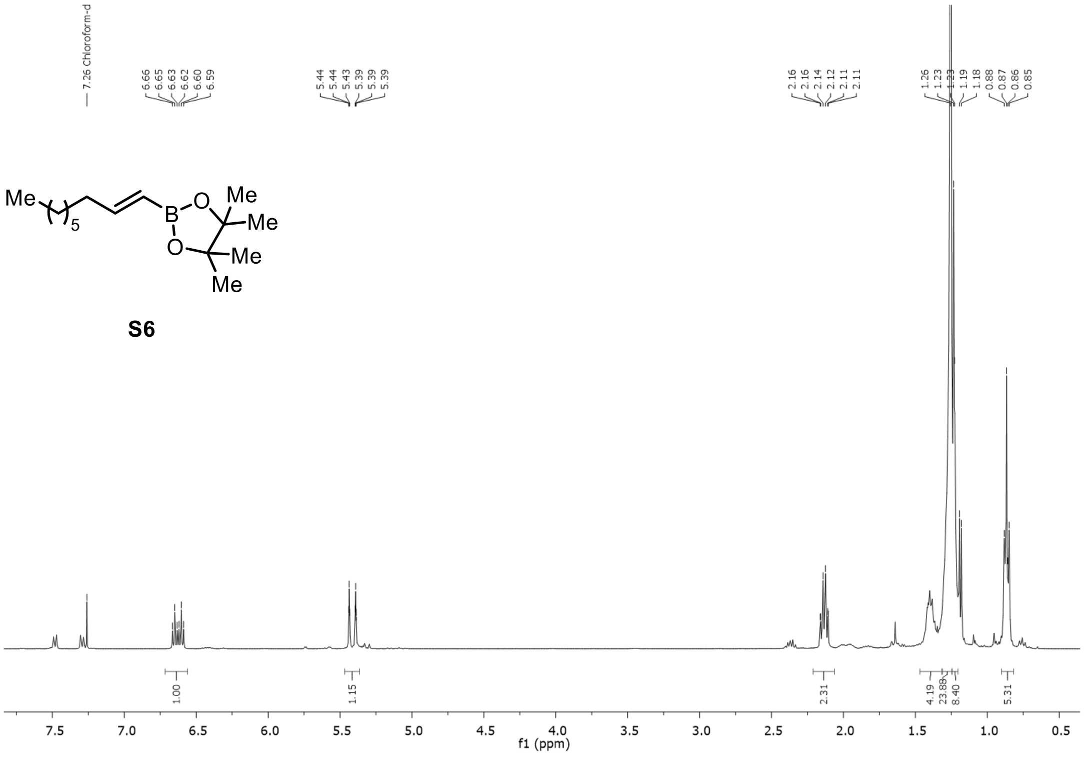

S7: 2-(3-(2,6-dimethylphenyl)-1-(indolin-1-yl)propyl)-6-methoxyphenol

^{1}H NMR (400 MHz, $CDCl_3$)

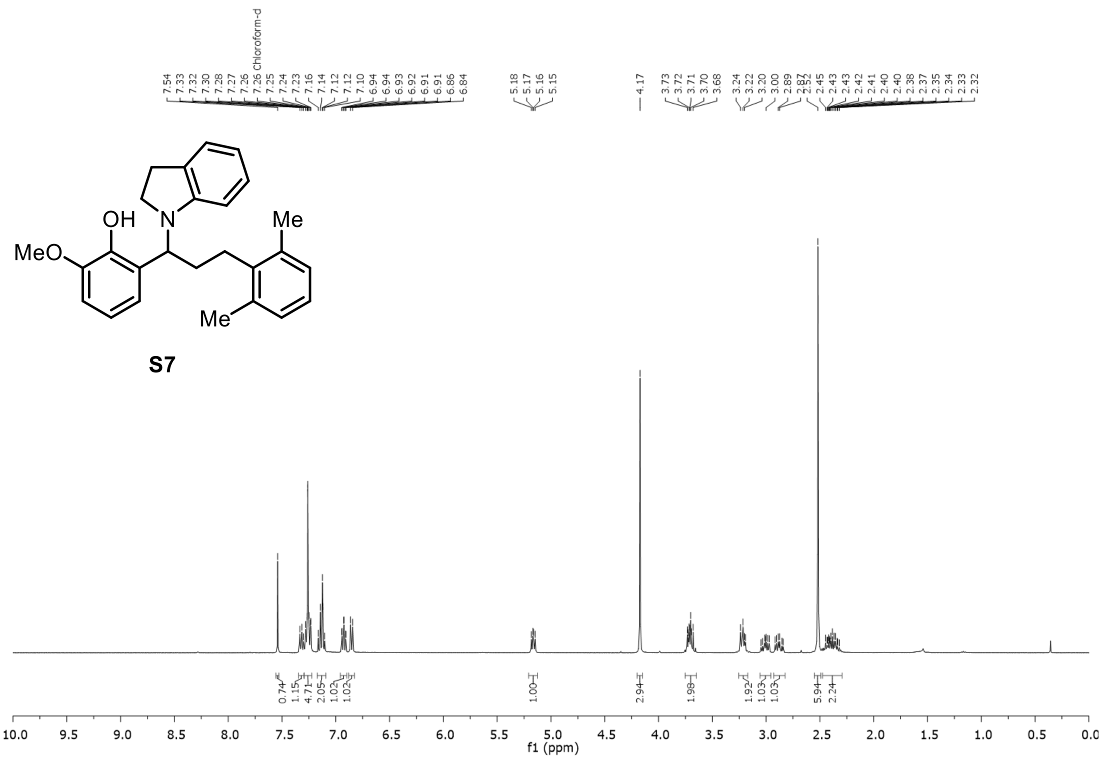

^{13}C NMR (101 MHz, $CDCl_3$)

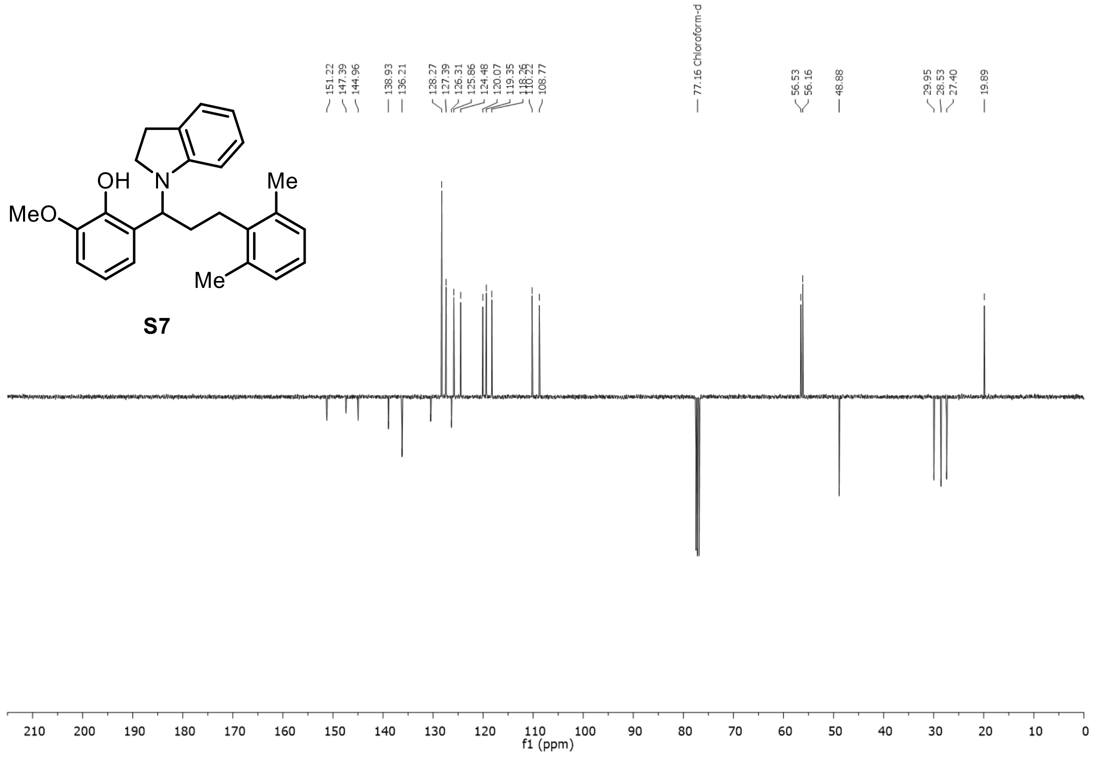

S8: (*E*)-but-1-ene-1,4-diyldibenzene

^{1}H NMR (400 MHz, $CDCl_3$)

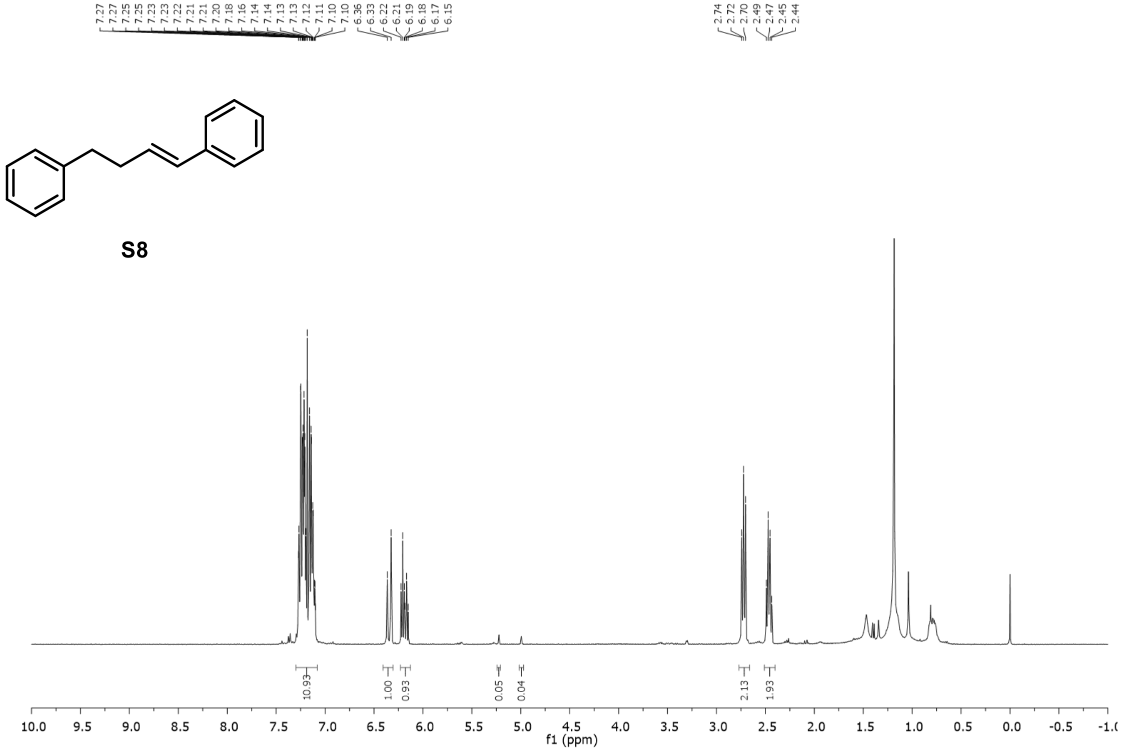

^{13}C NMR (101 MHz, $CDCl_3$)

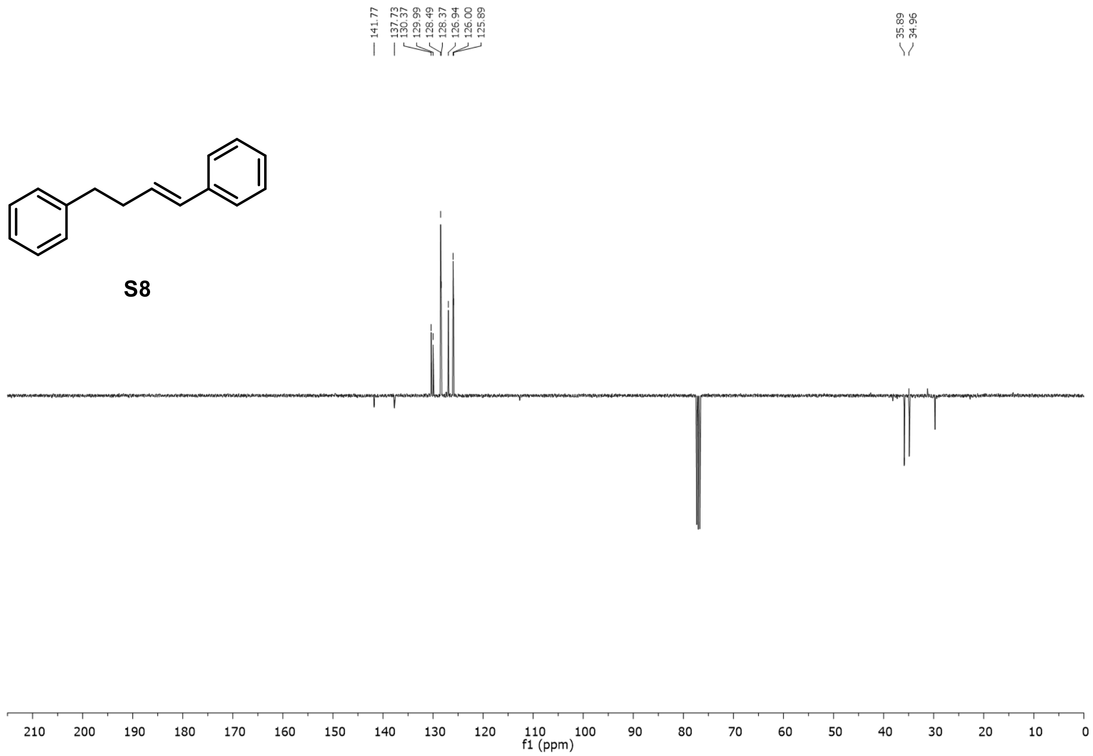

S9: (*E*)-undec-1-en-1-ylbenzene

^{1}H NMR (400 MHz, $CDCl_3$)

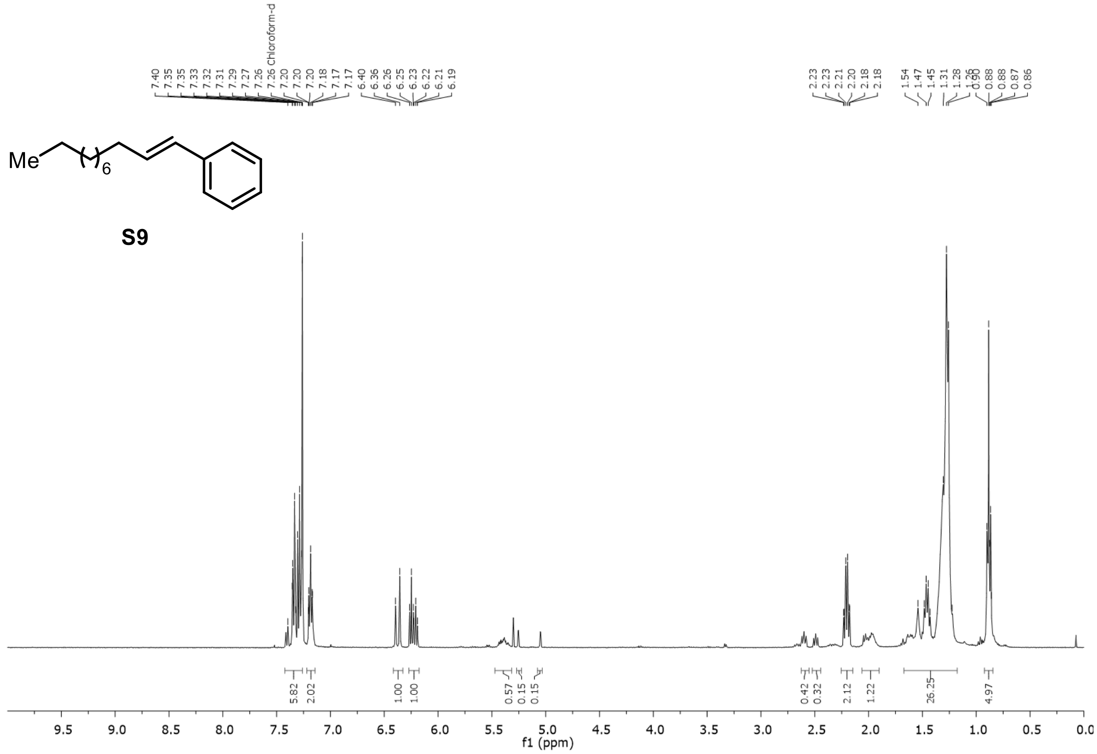

^{13}C NMR (101 MHz, $CDCl_3$)

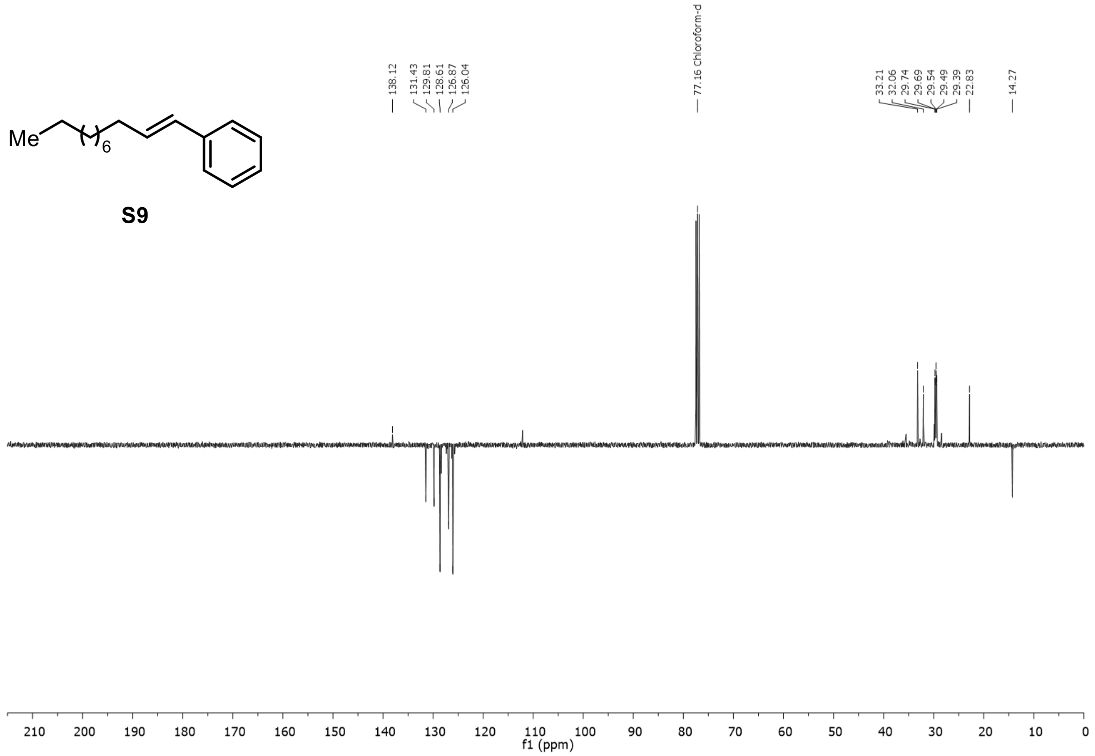

S10: ***syn*****-2-benzoylcyclohexane-1-carboxylic acid**

^{1}H NMR (400 MHz, $CDCl_3$)

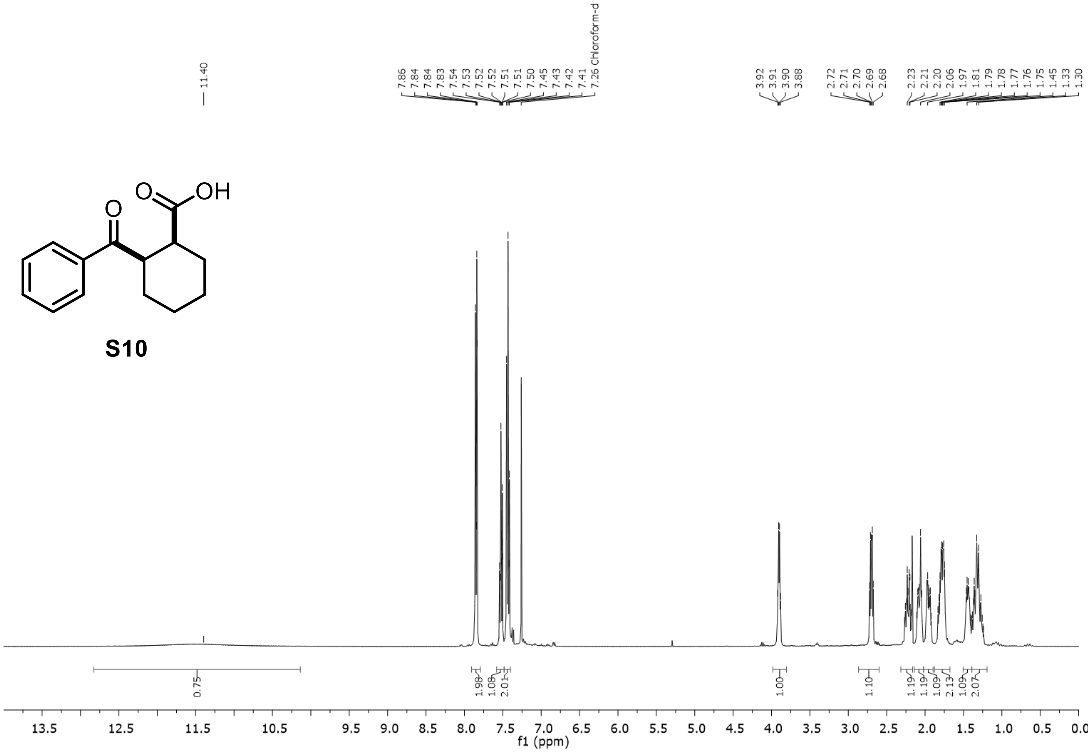

^{13}C NMR (101 MHz, $CDCl_3$)

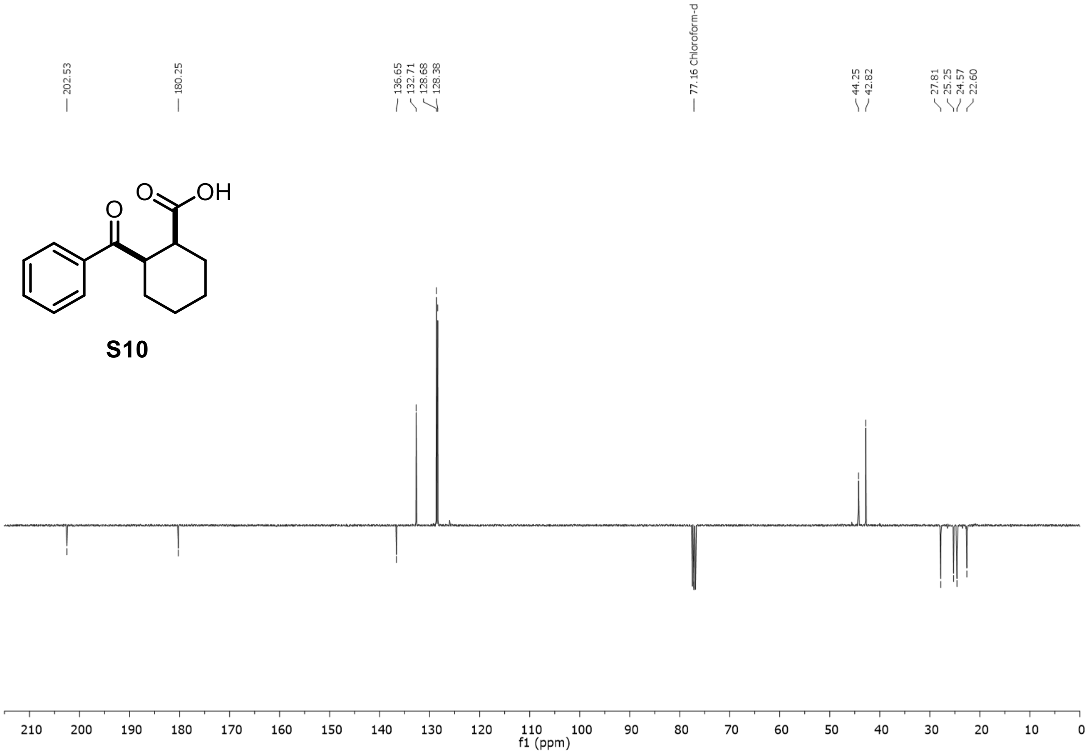

S11: *anti*-(2-bromocyclohexyl)(phenyl)methanone

^{1}H NMR (400 MHz, $CDCl_3$)

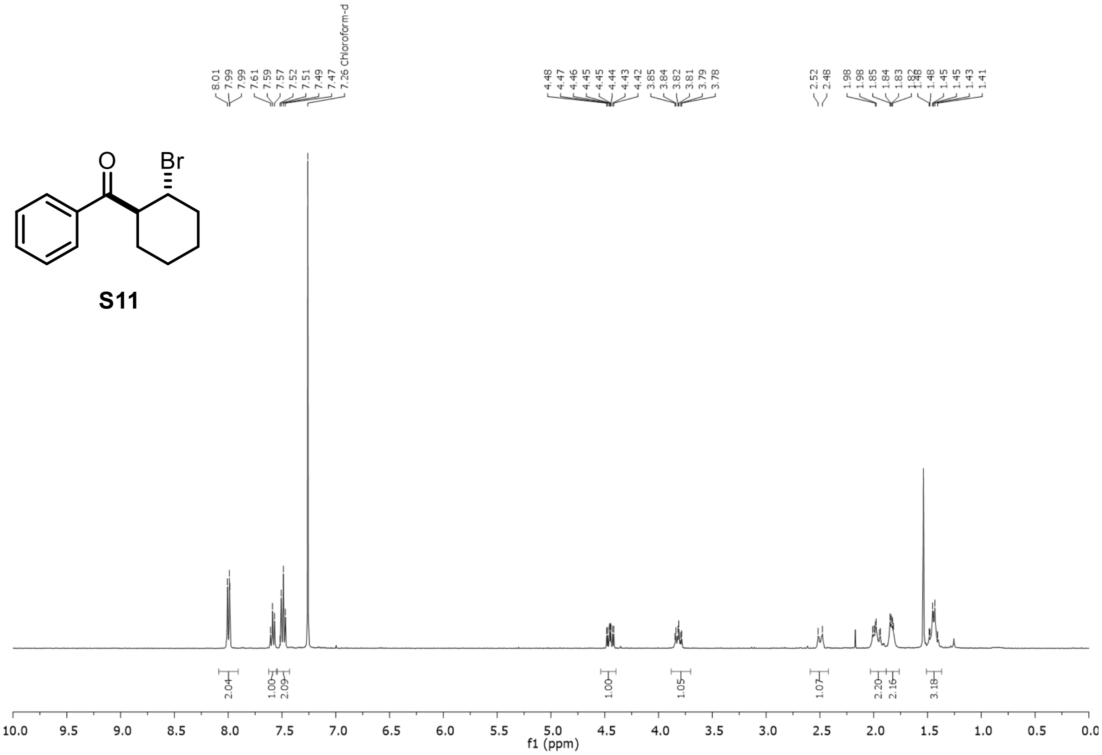

^{13}C NMR (101 MHz, $CDCl_3$)

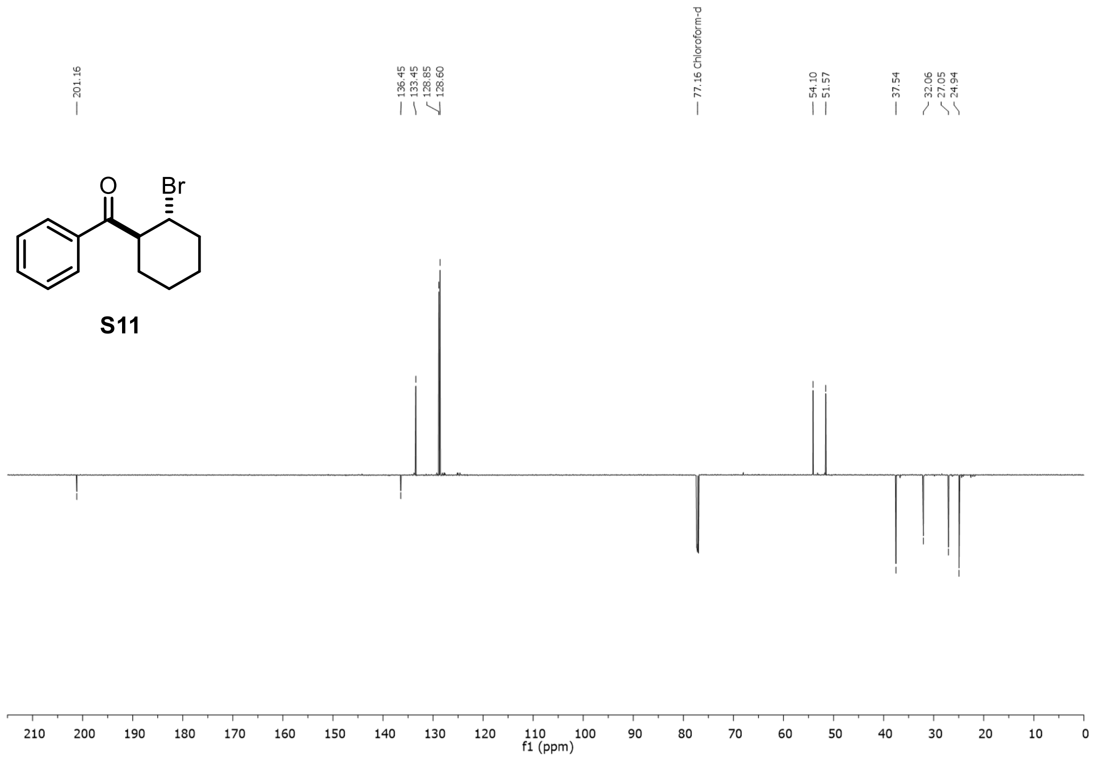

S12: (1-bromocyclohexyl)(phenyl)methanone

^{1}H NMR (400 MHz, $CDCl_3$)

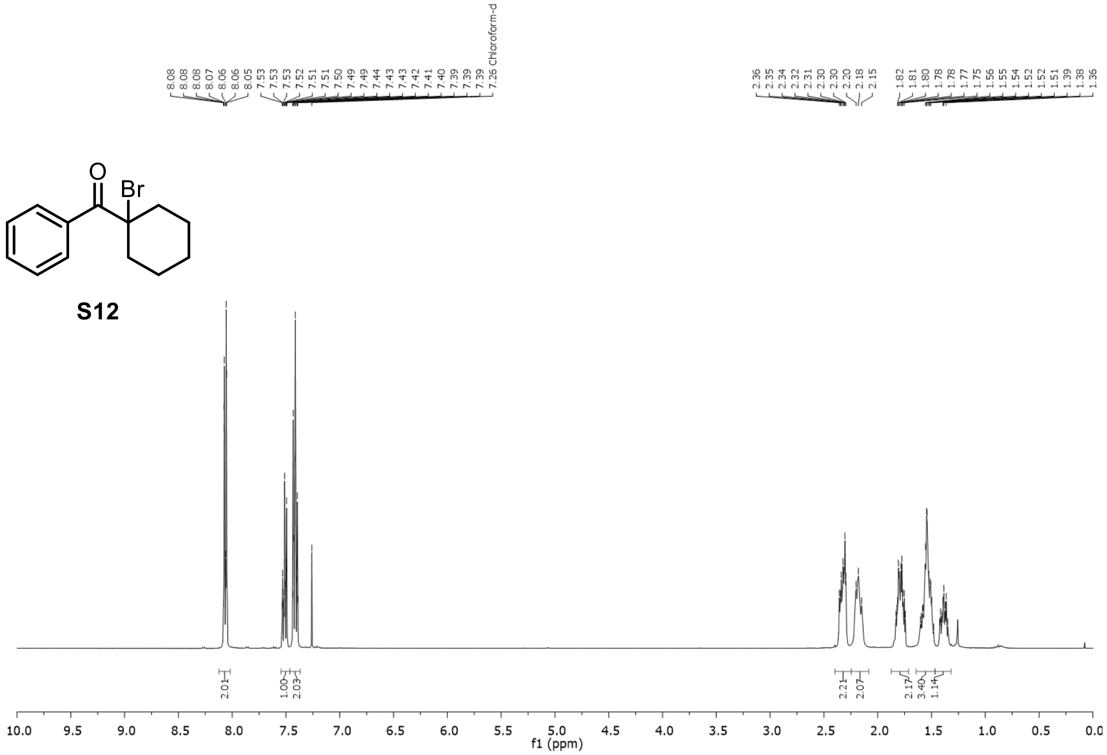

^{13}C NMR (101 MHz, $CDCl_3$)

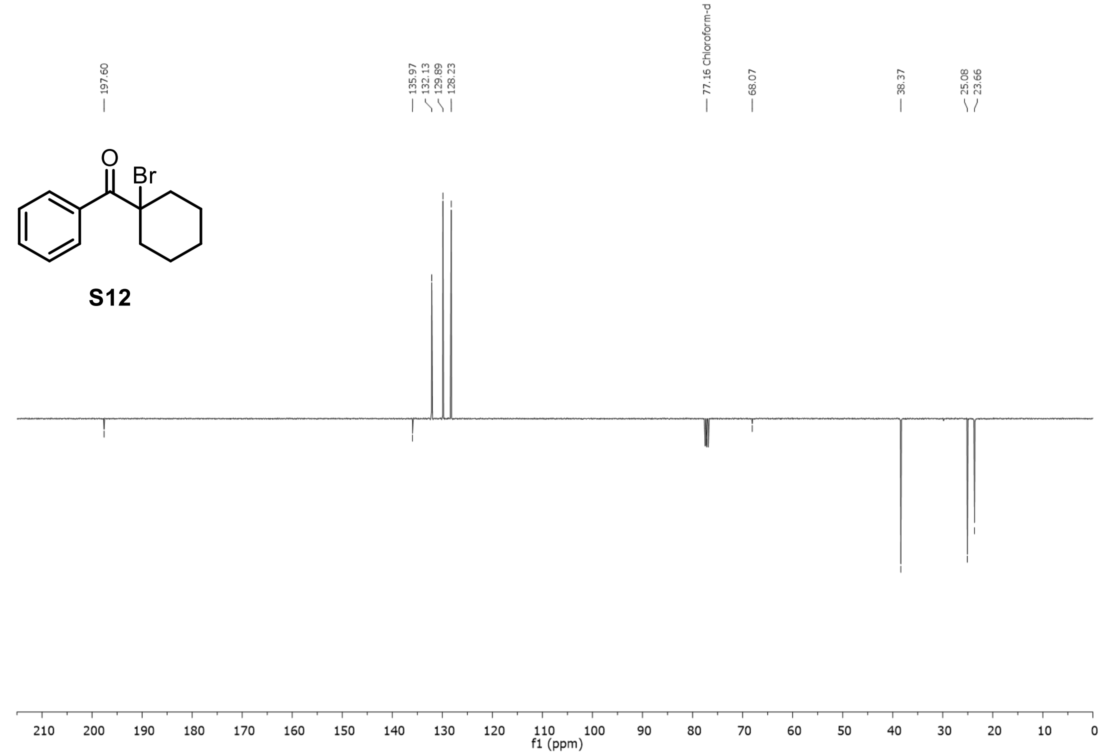

5. X-rays

3.10.1. Crystal Data for 10

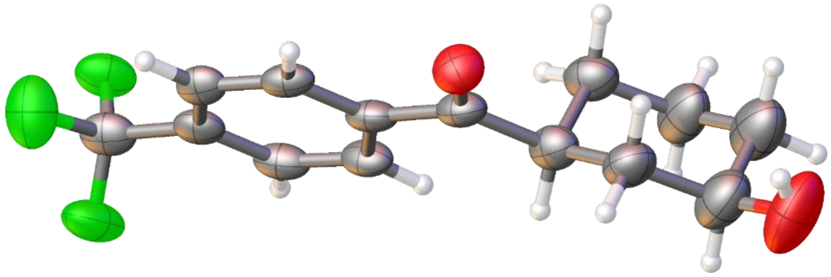

Table 1. Crystal data and structure refinement for 2244406.

Identification code	mo_daka1863_pbcn	
Empirical formula	C14 H15 F3 O2	
Formula weight	272.26	
Temperature	100.0 K	
Wavelength	0.71073 Å	
Crystal system	Orthorhombic	
Space group	Pbcn	
Unit cell dimensions	a = 34.170(6) Å	a= 90°.
	b = 15.018(3) Å	b= 90°.
	c = 5.6952(11) Å	g = 90°.
Volume	2922.5(10) $Å^3$	
Z	8	
Density (calculated)	1.238 mg/m^3	
Absorption coefficient	0.107 mm^{-1}	
F(000)	1136	
Crystal size	0.2 x 0.08 x 0.02 mm^3	
Theta range for data collection	2.244 to 25.347°.	
Index ranges	-41<=h<=34, -17<=k<=18, -6<=l<=6	
Reflections collected	9500	
Independent reflections	2675 [R(int) = 0.1329]	
Completeness to theta = 25.242°	99.6 %	
Absorption correction	Semi-empirical from equivalents	
Max. and min. transmission	0.7457 and 0.4670	
Refinement method	Full-matrix least-squares on F^2	
Data / restraints / parameters	2675 / 0 / 174	

Goodness-of-fit on F^2	1.034
Final R indices [I>2sigma(I)]	R1 = 0.1063, wR2 = 0.2785
R indices (all data)	R1 = 0.2068, wR2 = 0.3306
Extinction coefficient	0.025(5)
Largest diff. peak and hole	0.814 and -0.369 e.Å^{-3}

Table 2. Atomic coordinates (x 10^4) and equivalent isotropic displacement parameters (Å^{2}x 10^3) for 2244406. U(eq) is defined as one third of the trace of the orthogonalized U^{ij} tensor.

	x	y	z	U(eq)
F(1)	8269(1)	2809(2)	5534(6)	54(1)
O(2)	6405(1)	4254(3)	9580(8)	52(1)
F(3)	8230(1)	3973(2)	3409(7)	67(1)
F(4)	8370(1)	4087(2)	7062(7)	75(1)
C(5)	7717(2)	3731(3)	6093(10)	40(2)
C(6)	7195(2)	4145(3)	8668(10)	39(2)
C(7)	7446(2)	3435(3)	4471(10)	44(2)
C(8)	6916(2)	3861(3)	7037(9)	40(2)
C(9)	7593(2)	4090(3)	8225(9)	39(2)
C(10)	6490(2)	3962(3)	7644(10)	42(2)
C(11)	6180(2)	3664(4)	5913(11)	48(2)
C(12)	8142(2)	3660(4)	5537(11)	48(2)
O(13)	5093(2)	4105(4)	5239(12)	109(2)
C(14)	5781(2)	4071(4)	6400(12)	57(2)
C(15)	7050(2)	3512(3)	4901(9)	42(2)
C(16)	6156(2)	2637(4)	5928(11)	54(2)
C(17)	5454(2)	2715(5)	4772(15)	81(3)
C(18)	5471(2)	3736(5)	4713(14)	76(2)
C(19)	5849(2)	2318(4)	4255(13)	72(2)

Table 3. Bond lengths [Å] and angles [°] for 2244406.

F(1)-C(12)	1.351(6)
O(2)-C(10)	1.221(6)
F(3)-C(12)	1.335(7)
F(4)-C(12)	1.332(7)
C(5)-C(7)	1.382(8)
C(5)-C(9)	1.395(8)
C(5)-C(12)	1.489(9)
C(6)-H(6)	0.9500
C(6)-C(8)	1.397(8)
C(6)-C(9)	1.385(8)
C(7)-H(7)	0.9500
C(7)-C(15)	1.380(8)
C(8)-C(10)	1.504(9)
C(8)-C(15)	1.401(8)
C(9)-H(9)	0.9500
C(10)-C(11)	1.514(8)
C(11)-H(11)	1.0000
C(11)-C(14)	1.522(9)
C(11)-C(16)	1.546(8)
O(13)-H(13)	0.8400
O(13)-C(18)	1.437(10)
C(14)-H(14A)	0.9900
C(14)-H(14B)	0.9900
C(14)-C(18)	1.515(9)
C(15)-H(15)	0.9500
C(16)-H(16A)	0.9900
C(16)-H(16B)	0.9900
C(16)-C(19)	1.496(8)
C(17)-H(17A)	0.9900
C(17)-H(17B)	0.9900
C(17)-C(18)	1.536(10)
C(17)-C(19)	1.503(10)
C(18)-H(18)	1.0000
C(19)-H(19A)	0.9900

C(19)-H(19B)	0.9900
C(7)-C(5)-C(9)	120.1(6)
C(7)-C(5)-C(12)	119.3(5)
C(9)-C(5)-C(12)	120.7(5)
C(8)-C(6)-H(6)	119.0
C(9)-C(6)-H(6)	119.0
C(9)-C(6)-C(8)	122.0(5)
C(5)-C(7)-H(7)	119.6
C(15)-C(7)-C(5)	120.8(6)
C(15)-C(7)-H(7)	119.6
C(6)-C(8)-C(10)	118.4(5)
C(6)-C(8)-C(15)	118.0(6)
C(15)-C(8)-C(10)	123.6(6)
C(5)-C(9)-H(9)	120.6
C(6)-C(9)-C(5)	118.8(5)
C(6)-C(9)-H(9)	120.6
O(2)-C(10)-C(8)	118.3(5)
O(2)-C(10)-C(11)	121.9(6)
C(8)-C(10)-C(11)	119.8(5)
C(10)-C(11)-H(11)	108.0
C(10)-C(11)-C(14)	113.0(5)
C(10)-C(11)-C(16)	109.1(5)
C(14)-C(11)-H(11)	108.0
C(14)-C(11)-C(16)	110.6(5)
C(16)-C(11)-H(11)	108.0
F(1)-C(12)-C(5)	112.4(5)
F(3)-C(12)-F(1)	105.1(5)
F(3)-C(12)-C(5)	112.8(5)
F(4)-C(12)-F(1)	105.6(5)
F(4)-C(12)-F(3)	106.9(5)
F(4)-C(12)-C(5)	113.4(5)
C(18)-O(13)-H(13)	109.5
C(11)-C(14)-H(14A)	109.2
C(11)-C(14)-H(14B)	109.2
H(14A)-C(14)-H(14B)	107.9

C(18)-C(14)-C(11)	112.2(6)
C(18)-C(14)-H(14A)	109.2
C(18)-C(14)-H(14B)	109.2
C(7)-C(15)-C(8)	120.4(6)
C(7)-C(15)-H(15)	119.8
C(8)-C(15)-H(15)	119.8
C(11)-C(16)-H(16A)	109.5
C(11)-C(16)-H(16B)	109.5
H(16A)-C(16)-H(16B)	108.1
C(19)-C(16)-C(11)	110.6(5)
C(19)-C(16)-H(16A)	109.5
C(19)-C(16)-H(16B)	109.5
H(17A)-C(17)-H(17B)	108.0
C(18)-C(17)-H(17A)	109.4
C(18)-C(17)-H(17B)	109.4
C(19)-C(17)-H(17A)	109.4
C(19)-C(17)-H(17B)	109.4
C(19)-C(17)-C(18)	111.0(6)
O(13)-C(18)-C(14)	111.6(6)
O(13)-C(18)-C(17)	110.3(7)
O(13)-C(18)-H(18)	108.3
C(14)-C(18)-C(17)	110.0(6)
C(14)-C(18)-H(18)	108.3
C(17)-C(18)-H(18)	108.3
C(16)-C(19)-C(17)	112.2(6)
C(16)-C(19)-H(19A)	109.2
C(16)-C(19)-H(19B)	109.2
C(17)-C(19)-H(19A)	109.2
C(17)-C(19)-H(19B)	109.2
H(19A)-C(19)-H(19B)	107.9

Symmetry transformations used to generate equivalent atoms:

Table 4. Anisotropic displacement parameters ($Å^2$x 10^3) for 2244406. The anisotropic displacement factor exponent takes the form: $-2p^2[h^2 a^{*2}U^{11} + ... + 2 h k a^* b^* U^{12}]$

	U^{11}	U^{22}	U^{33}	U^{23}	U^{13}	U^{12}
F(1)	74(3)	33(2)	56(2)	5(2)	11(2)	11(2)
O(2)	58(3)	53(3)	43(3)	-6(2)	-1(2)	1(2)
F(3)	73(3)	60(2)	68(3)	28(2)	17(2)	10(2)
F(4)	61(3)	74(3)	89(3)	-33(2)	1(2)	-2(2)
C(5)	62(4)	22(3)	35(3)	3(2)	1(3)	0(3)
C(6)	66(5)	23(3)	27(3)	0(2)	-3(3)	5(3)
C(7)	76(5)	26(3)	28(3)	-1(3)	3(3)	-2(3)
C(8)	65(4)	27(3)	26(3)	4(2)	-3(3)	-1(3)
C(9)	59(4)	20(3)	38(4)	-3(3)	-5(3)	1(2)
C(10)	61(5)	35(3)	30(4)	4(3)	-6(3)	-2(3)
C(11)	62(5)	36(3)	47(4)	-2(3)	-8(3)	-5(3)
C(12)	75(5)	27(3)	43(4)	-4(3)	-1(4)	0(3)
O(13)	73(4)	120(5)	134(6)	-21(4)	-43(4)	26(3)
C(14)	66(5)	47(4)	58(4)	7(3)	-13(4)	4(3)
C(15)	67(5)	34(3)	24(3)	-1(2)	-4(3)	-7(3)
C(16)	72(5)	39(3)	52(4)	2(3)	-9(4)	-2(3)
C(17)	85(6)	62(5)	97(6)	-4(4)	-35(5)	-7(4)
C(18)	77(6)	69(5)	81(6)	-3(4)	-31(5)	2(4)
C(19)	77(6)	48(4)	91(6)	-7(4)	-35(5)	-8(4)

Table 5. Hydrogen coordinates (x 10^4) and isotropic displacement parameters (Å^{2}x 10 ^{3}) for 2244406.

	x	y	z	U(eq)
H(6)	7109	4383	10124	46
H(7)	7533	3175	3043	52
H(9)	7777	4292	9352	47
H(11)	6265	3856	4309	58
H(13)	5086	4264	6652	164
H(14A)	5701	3924	8026	68
H(14B)	5799	4727	6273	68
H(15)	6867	3328	3742	50
H(16A)	6413	2384	5477	65
H(16B)	6094	2427	7533	65
H(17A)	5366	2516	6341	97
H(17B)	5263	2501	3597	97
H(18)	5546	3925	3090	91
H(19A)	5831	1661	4348	87
H(19B)	5927	2477	2634	87

Table 6. Torsion angles [°] for 2244406.

O(2)-C(10)-C(11)-C(14)	21.4(7)
O(2)-C(10)-C(11)-C(16)	-102.1(6)
C(5)-C(7)-C(15)-C(8)	-2.6(8)
C(6)-C(8)-C(10)-O(2)	-3.0(7)
C(6)-C(8)-C(10)-C(11)	179.7(4)
C(6)-C(8)-C(15)-C(7)	1.7(7)
C(7)-C(5)-C(9)-C(6)	-0.3(7)
C(7)-C(5)-C(12)-F(1)	-70.0(7)
C(7)-C(5)-C(12)-F(3)	48.6(7)
C(7)-C(5)-C(12)-F(4)	170.3(5)
C(8)-C(6)-C(9)-C(5)	-0.6(7)
C(8)-C(10)-C(11)-C(14)	-161.5(5)
C(8)-C(10)-C(11)-C(16)	75.1(6)
C(9)-C(5)-C(7)-C(15)	1.9(7)
C(9)-C(5)-C(12)-F(1)	109.9(6)
C(9)-C(5)-C(12)-F(3)	-131.5(5)
C(9)-C(5)-C(12)-F(4)	-9.8(7)
C(9)-C(6)-C(8)-C(10)	-178.8(4)
C(9)-C(6)-C(8)-C(15)	-0.1(7)
C(10)-C(8)-C(15)-C(7)	-179.7(5)
C(10)-C(11)-C(14)-C(18)	-177.6(5)
C(10)-C(11)-C(16)-C(19)	179.3(6)
C(11)-C(14)-C(18)-O(13)	178.3(6)
C(11)-C(14)-C(18)-C(17)	55.4(8)
C(11)-C(16)-C(19)-C(17)	-56.3(8)
C(12)-C(5)-C(7)-C(15)	-178.3(5)
C(12)-C(5)-C(9)-C(6)	179.9(5)
C(14)-C(11)-C(16)-C(19)	54.4(7)
C(15)-C(8)-C(10)-O(2)	178.3(5)
C(15)-C(8)-C(10)-C(11)	1.1(7)
C(16)-C(11)-C(14)-C(18)	-55.0(7)
C(18)-C(17)-C(19)-C(16)	57.3(8)
C(19)-C(17)-C(18)-O(13)	-179.3(6)
C(19)-C(17)-C(18)-C(14)	-55.7(9)

Symmetry transformations used to generate equivalent atoms:

Table 7. Hydrogen bonds for 2244406 [Å and °].

D-H...A	d(D-H)	d(H...A)	d(D...A)	<(DHA)

3.10.2. Crystal Data for 28

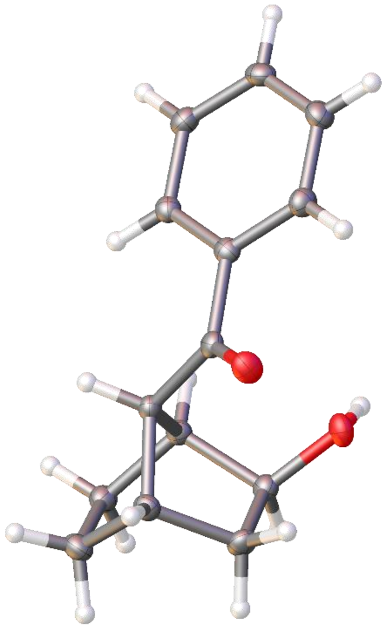

Table 1. Crystal data and structure refinement for 2244407.

Identification code	mo_daka1871_p21c	
Empirical formula	C14 H16 O2	
Formula weight	216.27	
Temperature	100.0 K	
Wavelength	0.71073 Å	
Crystal system	Monoclinic	
Space group	P 1 21/c 1	
Unit cell dimensions	a = 9.5206(12) Å	a= 90°.
	b = 10.4221(18) Å	b= 103.814(6)°.
	c = 11.8315(15) Å	g = 90°.
Volume	1140.0(3) Å^3	
Z	4	
Density (calculated)	1.260 mg/m^3	
Absorption coefficient	0.083 mm^{-1}	
F(000)	464	
Crystal size	0.05 x 0.04 x 0.02 mm^3	
Theta range for data collection	2.203 to 28.281°.	
Index ranges	-12<=h<=12, -13<=k<=13, -15<=l<=15	
Reflections collected	36477	
Independent reflections	2822 [R(int) = 0.0773]	

Completeness to theta = 25.242°	100.0 %
Absorption correction	Semi-empirical from equivalents
Max. and min. transmission	0.7460 and 0.7100
Refinement method	Full-matrix least-squares on F^2
Data / restraints / parameters	2822 / 0 / 146
Goodness-of-fit on F^2	1.094
Final R indices [I>2sigma(I)]	R1 = 0.0404, wR2 = 0.0991
R indices (all data)	R1 = 0.0623, wR2 = 0.1061
Extinction coefficient	n/a
Largest diff. peak and hole	0.288 and -0.235 e.Å^{-3}

Table 2. Atomic coordinates (x 10^4) and equivalent isotropic displacement parameters (Å^{2}x 10^3) for 2244408. U(eq) is defined as one third of the trace of the orthogonalized U^{ij} tensor.

	x	y	z	U(eq)
O(1)	3738(1)	4341(1)	2749(1)	21(1)
O(2)	5558(1)	1930(1)	2227(1)	21(1)
C(3)	4104(1)	3521(1)	3510(1)	16(1)
C(4)	6527(1)	2300(1)	4311(1)	16(1)
C(5)	5593(1)	3521(1)	4320(1)	15(1)
C(6)	3028(1)	2537(1)	3674(1)	16(1)
C(7)	3164(1)	1868(1)	4721(1)	17(1)
C(8)	7005(2)	3806(1)	2903(1)	20(1)
C(9)	1834(1)	2295(1)	2749(1)	19(1)
C(10)	8035(1)	4150(1)	5018(1)	22(1)
C(11)	6792(1)	2357(1)	3085(1)	18(1)
C(12)	951(1)	734(1)	3903(1)	20(1)
C(13)	2120(1)	975(1)	4832(1)	19(1)
C(14)	6720(1)	4432(1)	4009(1)	18(1)
C(15)	7943(1)	2675(1)	5185(1)	19(1)
C(16)	808(1)	1396(1)	2863(1)	21(1)

Table 3. Bond lengths [Å] and angles [°] for 2244408.

O(1)-C(3)	1.2305(15)
O(2)-H(2)	0.8400
O(2)-C(11)	1.4280(15)
C(3)-C(5)	1.5099(16)
C(3)-C(6)	1.4943(17)
C(4)-H(4)	1.0000
C(4)-C(5)	1.5543(17)
C(4)-C(11)	1.5316(18)
C(4)-C(15)	1.5408(16)
C(5)-H(5)	1.0000
C(5)-C(14)	1.5419(17)
C(6)-C(7)	1.3996(17)
C(6)-C(9)	1.3997(17)
C(7)-H(7)	0.9500
C(7)-C(13)	1.3908(18)
C(8)-H(8A)	0.9900
C(8)-H(8B)	0.9900
C(8)-C(11)	1.5459(18)
C(8)-C(14)	1.5433(18)
C(9)-H(9)	0.9500
C(9)-C(16)	1.3830(19)
C(10)-H(10A)	0.9900
C(10)-H(10B)	0.9900
C(10)-C(14)	1.5382(18)
C(10)-C(15)	1.5554(18)
C(11)-H(11)	1.0000
C(12)-H(12)	0.9500
C(12)-C(13)	1.3873(18)
C(12)-C(16)	1.3889(19)
C(13)-H(13)	0.9500
C(14)-H(14)	1.0000
C(15)-H(15A)	0.9900
C(15)-H(15B)	0.9900
C(16)-H(16)	0.9500

C(11)-O(2)-H(2)	109.5
O(1)-C(3)-C(5)	121.53(11)
O(1)-C(3)-C(6)	119.29(11)
C(6)-C(3)-C(5)	119.12(11)
C(5)-C(4)-H(4)	114.8
C(11)-C(4)-H(4)	114.8
C(11)-C(4)-C(5)	101.47(10)
C(11)-C(4)-C(15)	108.08(10)
C(15)-C(4)-H(4)	114.8
C(15)-C(4)-C(5)	101.42(10)
C(3)-C(5)-C(4)	116.57(10)
C(3)-C(5)-H(5)	109.5
C(3)-C(5)-C(14)	116.76(10)
C(4)-C(5)-H(5)	109.5
C(14)-C(5)-C(4)	94.04(9)
C(14)-C(5)-H(5)	109.5
C(7)-C(6)-C(3)	122.35(11)
C(7)-C(6)-C(9)	119.25(12)
C(9)-C(6)-C(3)	118.40(11)
C(6)-C(7)-H(7)	120.0
C(13)-C(7)-C(6)	119.97(11)
C(13)-C(7)-H(7)	120.0
H(8A)-C(8)-H(8B)	109.0
C(11)-C(8)-H(8A)	111.0
C(11)-C(8)-H(8B)	111.0
C(14)-C(8)-H(8A)	111.0
C(14)-C(8)-H(8B)	111.0
C(14)-C(8)-C(11)	103.84(10)
C(6)-C(9)-H(9)	119.8
C(16)-C(9)-C(6)	120.39(12)
C(16)-C(9)-H(9)	119.8
H(10A)-C(10)-H(10B)	109.1
C(14)-C(10)-H(10A)	111.1
C(14)-C(10)-H(10B)	111.1
C(14)-C(10)-C(15)	103.14(10)
C(15)-C(10)-H(10A)	111.1

C(15)-C(10)-H(10B)	111.1
O(2)-C(11)-C(4)	111.56(10)
O(2)-C(11)-C(8)	108.57(10)
O(2)-C(11)-H(11)	111.1
C(4)-C(11)-C(8)	103.11(10)
C(4)-C(11)-H(11)	111.1
C(8)-C(11)-H(11)	111.1
C(13)-C(12)-H(12)	119.9
C(13)-C(12)-C(16)	120.12(12)
C(16)-C(12)-H(12)	119.9
C(7)-C(13)-H(13)	119.9
C(12)-C(13)-C(7)	120.17(12)
C(12)-C(13)-H(13)	119.9
C(5)-C(14)-C(8)	102.45(10)
C(5)-C(14)-H(14)	114.9
C(8)-C(14)-H(14)	114.9
C(10)-C(14)-C(5)	100.42(10)
C(10)-C(14)-C(8)	107.61(11)
C(10)-C(14)-H(14)	114.9
C(4)-C(15)-C(10)	103.43(10)
C(4)-C(15)-H(15A)	111.1
C(4)-C(15)-H(15B)	111.1
C(10)-C(15)-H(15A)	111.1
C(10)-C(15)-H(15B)	111.1
H(15A)-C(15)-H(15B)	109.0
C(9)-C(16)-C(12)	120.09(12)
C(9)-C(16)-H(16)	120.0
C(12)-C(16)-H(16)	120.0

Symmetry transformations used to generate equivalent atoms:

Table 4. Anisotropic displacement parameters ($Å^2$x 10^3) for 2244408. The anisotropic displacement factor exponent takes the form: $-2p^2[h^2 a^{*2}U^{11} + ... + 2 h k a^* b^* U^{12}]$

	U^{11}	U^{22}	U^{33}	U^{23}	U^{13}	U^{12}
O(1)	24(1)	17(1)	21(1)	4(1)	1(1)	0(1)
O(2)	25(1)	17(1)	18(1)	-4(1)	2(1)	-1(1)
C(3)	18(1)	16(1)	14(1)	-3(1)	5(1)	1(1)
C(4)	16(1)	15(1)	16(1)	0(1)	4(1)	0(1)
C(5)	16(1)	16(1)	14(1)	-1(1)	3(1)	-1(1)
C(6)	15(1)	15(1)	18(1)	-1(1)	4(1)	2(1)
C(7)	15(1)	18(1)	17(1)	-1(1)	3(1)	2(1)
C(8)	23(1)	19(1)	20(1)	2(1)	9(1)	-3(1)
C(9)	19(1)	22(1)	17(1)	2(1)	3(1)	2(1)
C(10)	17(1)	23(1)	24(1)	-3(1)	3(1)	-4(1)
C(11)	17(1)	19(1)	17(1)	-2(1)	5(1)	0(1)
C(12)	16(1)	21(1)	25(1)	-2(1)	8(1)	-2(1)
C(13)	21(1)	20(1)	18(1)	2(1)	8(1)	1(1)
C(14)	18(1)	15(1)	20(1)	-1(1)	5(1)	-2(1)
C(15)	16(1)	22(1)	18(1)	1(1)	3(1)	0(1)
C(16)	16(1)	25(1)	21(1)	-1(1)	2(1)	-1(1)

Table 5. Hydrogen coordinates (x 10^4) and isotropic displacement parameters ($Å^2$x 10^3) for 2244408.

	x	y	z	U(eq)
H(2)	5512	1125	2244	31
H(4)	6078	1484	4498	19
H(5)	5512	3699	5133	18
H(7)	3969	2025	5355	20
H(8A)	8001	3987	2831	24
H(8B)	6310	4121	2197	24
H(9)	1728	2751	2038	23
H(10A)	7966	4617	5732	26
H(10B)	8949	4390	4812	26
H(11)	7675	1859	3041	21
H(12)	247	114	3979	24
H(13)	2208	528	5545	23
H(14)	6424	5354	3922	21
H(15A)	8783	2232	5005	23
H(15B)	7900	2463	5992	23
H(16)	5	1232	2230	25

Table 6. Torsion angles [°] for 2244408.

O(1)-C(3)-C(5)-C(4)	-120.98(13)
O(1)-C(3)-C(5)-C(14)	-11.08(17)
O(1)-C(3)-C(6)-C(7)	-157.76(12)
O(1)-C(3)-C(6)-C(9)	21.27(18)
C(3)-C(5)-C(14)-C(8)	-69.37(13)
C(3)-C(5)-C(14)-C(10)	179.80(10)
C(3)-C(6)-C(7)-C(13)	179.11(11)
C(3)-C(6)-C(9)-C(16)	-179.73(12)
C(4)-C(5)-C(14)-C(8)	53.16(11)
C(4)-C(5)-C(14)-C(10)	-57.67(10)
C(5)-C(3)-C(6)-C(7)	19.40(18)
C(5)-C(3)-C(6)-C(9)	-161.57(11)
C(5)-C(4)-C(11)-O(2)	-78.13(12)
C(5)-C(4)-C(11)-C(8)	38.21(12)
C(5)-C(4)-C(15)-C(10)	-32.42(12)
C(6)-C(3)-C(5)-C(4)	61.93(15)
C(6)-C(3)-C(5)-C(14)	171.82(11)
C(6)-C(7)-C(13)-C(12)	0.66(19)
C(6)-C(9)-C(16)-C(12)	0.5(2)
C(7)-C(6)-C(9)-C(16)	-0.67(19)
C(9)-C(6)-C(7)-C(13)	0.09(19)
C(11)-C(4)-C(5)-C(3)	66.61(13)
C(11)-C(4)-C(5)-C(14)	-56.07(10)
C(11)-C(4)-C(15)-C(10)	73.80(12)
C(11)-C(8)-C(14)-C(5)	-31.52(12)
C(11)-C(8)-C(14)-C(10)	73.82(12)
C(13)-C(12)-C(16)-C(9)	0.3(2)
C(14)-C(8)-C(11)-O(2)	114.21(11)
C(14)-C(8)-C(11)-C(4)	-4.24(12)
C(14)-C(10)-C(15)-C(4)	-3.84(13)
C(15)-C(4)-C(5)-C(3)	177.96(10)
C(15)-C(4)-C(5)-C(14)	55.28(11)
C(15)-C(4)-C(11)-O(2)	175.69(10)
C(15)-C(4)-C(11)-C(8)	-67.98(12)

C(15)-C(10)-C(14)-C(5)	39.05(12)
C(15)-C(10)-C(14)-C(8)	-67.70(12)
C(16)-C(12)-C(13)-C(7)	-0.8(2)

Symmetry transformations used to generate equivalent atoms:

Table 7. Hydrogen bonds for 2244408 [Å and °].

D-H...A	d(D-H)	d(H...A)	d(D...A)	<(DHA)

3.10.3. Crystal Data for 54

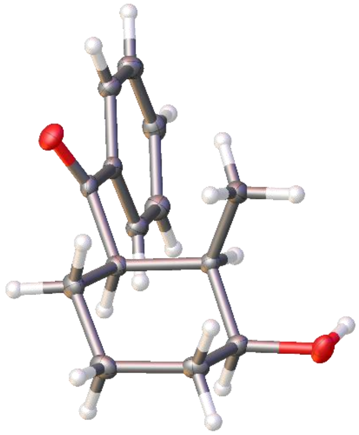

Table 1. Crystal data and structure refinement for 2244408.

Identification code	daka1872_p21c	
Empirical formula	C14 H18 O2	
Formula weight	218.28	
Temperature	100.0 K	
Wavelength	0.71073 Å	
Crystal system	Monoclinic	
Space group	P 1 21/c 1	
Unit cell dimensions	a = 6.55120(10) Å	a= 90°.
	b = 10.8561(3) Å	b= 94.2465(11)°.
	c = 16.2370(4) Å	g = 90°.
Volume	1151.61(5) $Å^3$	
Z	4	
Density (calculated)	1.259 mg/m^3	
Absorption coefficient	0.082 mm^{-1}	
F(000)	472	
Crystal size	0.4 x 0.3 x 0.2 mm^3	
Theta range for data collection	2.259 to 30.518°.	
Index ranges	-9<=h<=7, -12<=k<=14, -23<=l<=19	
Reflections collected	7048	
Independent reflections	3203 [R(int) = 0.0425]	
Completeness to theta = 25.242°	99.2 %	
Absorption correction	Semi-empirical from equivalents	
Max. and min. transmission	0.6478 and 0.6211	

Refinement method	Full-matrix least-squares on F^2
Data / restraints / parameters	3203 / 0 / 147
Goodness-of-fit on F^2	1.092
Final R indices [I>2sigma(I)]	R1 = 0.0425, wR2 = 0.0971
R indices (all data)	R1 = 0.0729, wR2 = 0.1111
Extinction coefficient	n/a
Largest diff. peak and hole	0.357 and -0.244 e.Å^{-3}

Table 2. Atomic coordinates (x 10^4) and equivalent isotropic displacement parameters (Å^{2}x 10^3) for DAKA1872. U(eq) is defined as one third of the trace of the orthogonalized U^{ij} tensor.

	x	y	z	U(eq)
O(1)	3930(1)	6672(1)	3909(1)	22(1)
O(2)	-1140(1)	3544(1)	1741(1)	20(1)
C(3)	1843(2)	5577(1)	2820(1)	14(1)
C(4)	-618(2)	5443(1)	1076(1)	14(1)
C(5)	2016(2)	4526(1)	2180(1)	14(1)
C(6)	5(2)	4421(1)	1661(1)	14(1)
C(7)	3936(2)	5719(1)	3302(1)	16(1)
C(8)	-2682(2)	5546(1)	791(1)	18(1)
C(9)	4652(2)	4534(1)	3727(1)	18(1)
C(10)	780(2)	6295(1)	802(1)	16(1)
C(11)	4746(2)	3492(1)	3099(1)	19(1)
C(12)	2703(2)	3317(1)	2593(1)	18(1)
C(13)	-1909(2)	7311(1)	-28(1)	19(1)
C(14)	142(2)	7217(1)	250(1)	19(1)
C(15)	91(2)	5368(1)	3376(1)	19(1)
C(16)	-3315(2)	6474(1)	246(1)	20(1)

Table 3. Bond lengths [Å] and angles [°] for DAKA1872.

O(1)-H(1)	0.8400
O(1)-C(7)	1.4284(15)
O(2)-C(6)	1.2248(15)
C(3)-H(3)	1.0000
C(3)-C(5)	1.5526(17)
C(3)-C(7)	1.5358(16)
C(3)-C(15)	1.5293(17)
C(4)-C(6)	1.4968(17)
C(4)-C(8)	1.4009(16)
C(4)-C(10)	1.3980(17)
C(5)-H(5)	1.0000
C(5)-C(6)	1.5155(16)
C(5)-C(12)	1.5258(17)
C(7)-H(7)	1.0000
C(7)-C(9)	1.5176(18)
C(8)-H(8)	0.9500
C(8)-C(16)	1.3837(19)
C(9)-H(9A)	0.9900
C(9)-H(9B)	0.9900
C(9)-C(11)	1.5272(18)
C(10)-H(10)	0.9500
C(10)-C(14)	1.3866(18)
C(11)-H(11A)	0.9900
C(11)-H(11B)	0.9900
C(11)-C(12)	1.5303(18)
C(12)-H(12A)	0.9900
C(12)-H(12B)	0.9900
C(13)-H(13)	0.9500
C(13)-C(14)	1.3888(18)
C(13)-C(16)	1.3905(19)
C(14)-H(14)	0.9500
C(15)-H(15A)	0.9800
C(15)-H(15B)	0.9800
C(15)-H(15C)	0.9800

C(16)-H(16)	0.9500
C(7)-O(1)-H(1)	109.5
C(5)-C(3)-H(3)	107.7
C(7)-C(3)-H(3)	107.7
C(7)-C(3)-C(5)	107.95(10)
C(15)-C(3)-H(3)	107.7
C(15)-C(3)-C(5)	112.41(10)
C(15)-C(3)-C(7)	113.20(10)
C(8)-C(4)-C(6)	118.67(11)
C(10)-C(4)-C(6)	122.58(11)
C(10)-C(4)-C(8)	118.74(12)
C(3)-C(5)-H(5)	107.5
C(6)-C(5)-C(3)	108.88(9)
C(6)-C(5)-H(5)	107.5
C(6)-C(5)-C(12)	113.31(10)
C(12)-C(5)-C(3)	111.88(10)
C(12)-C(5)-H(5)	107.5
O(2)-C(6)-C(4)	120.24(11)
O(2)-C(6)-C(5)	120.85(11)
C(4)-C(6)-C(5)	118.84(10)
O(1)-C(7)-C(3)	112.10(10)
O(1)-C(7)-H(7)	107.9
O(1)-C(7)-C(9)	108.48(10)
C(3)-C(7)-H(7)	107.9
C(9)-C(7)-C(3)	112.42(10)
C(9)-C(7)-H(7)	107.9
C(4)-C(8)-H(8)	119.8
C(16)-C(8)-C(4)	120.37(12)
C(16)-C(8)-H(8)	119.8
C(7)-C(9)-H(9A)	109.5
C(7)-C(9)-H(9B)	109.5
C(7)-C(9)-C(11)	110.53(10)
H(9A)-C(9)-H(9B)	108.1
C(11)-C(9)-H(9A)	109.5
C(11)-C(9)-H(9B)	109.5

C(4)-C(10)-H(10)	119.6
C(14)-C(10)-C(4)	120.70(11)
C(14)-C(10)-H(10)	119.6
C(9)-C(11)-H(11A)	109.2
C(9)-C(11)-H(11B)	109.2
C(9)-C(11)-C(12)	111.96(10)
H(11A)-C(11)-H(11B)	107.9
C(12)-C(11)-H(11A)	109.2
C(12)-C(11)-H(11B)	109.2
C(5)-C(12)-C(11)	110.29(11)
C(5)-C(12)-H(12A)	109.6
C(5)-C(12)-H(12B)	109.6
C(11)-C(12)-H(12A)	109.6
C(11)-C(12)-H(12B)	109.6
H(12A)-C(12)-H(12B)	108.1
C(14)-C(13)-H(13)	120.1
C(14)-C(13)-C(16)	119.76(12)
C(16)-C(13)-H(13)	120.1
C(10)-C(14)-C(13)	120.02(12)
C(10)-C(14)-H(14)	120.0
C(13)-C(14)-H(14)	120.0
C(3)-C(15)-H(15A)	109.5
C(3)-C(15)-H(15B)	109.5
C(3)-C(15)-H(15C)	109.5
H(15A)-C(15)-H(15B)	109.5
H(15A)-C(15)-H(15C)	109.5
H(15B)-C(15)-H(15C)	109.5
C(8)-C(16)-C(13)	120.39(12)
C(8)-C(16)-H(16)	119.8
C(13)-C(16)-H(16)	119.8

Symmetry transformations used to generate equivalent atoms:

Table 4. Anisotropic displacement parameters (Å^{2}x 10^3) for DAKA1872. The anisotropic displacement factor exponent takes the form: -2p^2[h^2 a*^{2}U^{11} + ... + 2 h k a* b* U^{12}]

	U^{11}	U^{22}	U^{33}	U^{23}	U^{13}	U^{12}
O(1)	23(1)	17(1)	23(1)	-4(1)	-8(1)	2(1)
O(2)	22(1)	16(1)	23(1)	-1(1)	1(1)	-6(1)
C(3)	14(1)	12(1)	16(1)	0(1)	-1(1)	0(1)
C(4)	16(1)	15(1)	13(1)	-3(1)	1(1)	0(1)
C(5)	14(1)	14(1)	16(1)	0(1)	2(1)	0(1)
C(6)	16(1)	14(1)	14(1)	-4(1)	3(1)	-1(1)
C(7)	14(1)	15(1)	18(1)	0(1)	-1(1)	-1(1)
C(8)	16(1)	20(1)	18(1)	-3(1)	1(1)	-2(1)
C(9)	14(1)	19(1)	20(1)	4(1)	-1(1)	2(1)
C(10)	15(1)	19(1)	15(1)	-2(1)	0(1)	-1(1)
C(11)	17(1)	17(1)	24(1)	4(1)	2(1)	4(1)
C(12)	19(1)	14(1)	22(1)	0(1)	1(1)	2(1)
C(13)	22(1)	19(1)	14(1)	0(1)	0(1)	4(1)
C(14)	20(1)	19(1)	17(1)	1(1)	2(1)	-2(1)
C(15)	15(1)	21(1)	21(1)	-5(1)	2(1)	0(1)
C(16)	16(1)	26(1)	18(1)	-2(1)	0(1)	2(1)

Table 5. Hydrogen coordinates (x 10^4) and isotropic displacement parameters (Å^{2}x 10 ^{3}) for DAKA1872.

	x	y	z	U(eq)
H(1)	3245	7273	3715	33
H(3)	1553	6358	2506	16
H(5)	3087	4772	1804	17
H(7)	4958	5947	2900	19
H(8)	-3653	4976	973	21
H(9A)	3698	4307	4147	22
H(9B)	6025	4660	4012	22
H(10)	2181	6243	995	19
H(11A)	5120	2716	3393	23
H(11B)	5826	3676	2722	23
H(12A)	2852	2679	2166	22
H(12B)	1653	3033	2958	22
H(13)	-2349	7945	-403	22
H(14)	1109	7784	62	22
H(15A)	-1214	5363	3040	28
H(15B)	83	6032	3785	28
H(15C)	283	4576	3661	28
H(16)	-4719	6539	58	24

Table 6. Torsion angles [°] for DAKA1872.

O(1)-C(7)-C(9)-C(11)	-178.34(10)
C(3)-C(5)-C(6)-O(2)	-108.54(13)
C(3)-C(5)-C(6)-C(4)	68.22(13)
C(3)-C(5)-C(12)-C(11)	-56.88(13)
C(3)-C(7)-C(9)-C(11)	57.12(13)
C(4)-C(8)-C(16)-C(13)	0.31(19)
C(4)-C(10)-C(14)-C(13)	0.85(19)
C(5)-C(3)-C(7)-O(1)	179.94(10)
C(5)-C(3)-C(7)-C(9)	-57.54(13)
C(6)-C(4)-C(8)-C(16)	-179.56(11)
C(6)-C(4)-C(10)-C(14)	178.97(11)
C(6)-C(5)-C(12)-C(11)	179.56(10)
C(7)-C(3)-C(5)-C(6)	-176.57(10)
C(7)-C(3)-C(5)-C(12)	57.41(12)
C(7)-C(9)-C(11)-C(12)	-54.93(14)
C(8)-C(4)-C(6)-O(2)	16.19(17)
C(8)-C(4)-C(6)-C(5)	-160.60(11)
C(8)-C(4)-C(10)-C(14)	-0.74(18)
C(9)-C(11)-C(12)-C(5)	55.00(13)
C(10)-C(4)-C(6)-O(2)	-163.52(12)
C(10)-C(4)-C(6)-C(5)	19.70(17)
C(10)-C(4)-C(8)-C(16)	0.16(18)
C(12)-C(5)-C(6)-O(2)	16.64(16)
C(12)-C(5)-C(6)-C(4)	-166.59(10)
C(14)-C(13)-C(16)-C(8)	-0.21(19)
C(15)-C(3)-C(5)-C(6)	57.87(13)
C(15)-C(3)-C(5)-C(12)	-68.15(13)
C(15)-C(3)-C(7)-O(1)	-54.97(14)
C(15)-C(3)-C(7)-C(9)	67.55(13)
C(16)-C(13)-C(14)-C(10)	-0.36(19)

Symmetry transformations used to generate equivalent atoms:

Table 7. Hydrogen bonds for DAKA1872 [Å and °].

D-H...A	d(D-H)	d(H...A)	d(D...A)	<(DHA)

6. References

[1] Fulmer, G. R. et al. NMR chemical shifts of trace impurities: common laboratory solvents, organics, and gases in deuterated solvents relevant to the organometallic chemist. *Organometallics* **29**, 2176–2179 (2010).

[2] Yu, Z., Eno, M. S., Annis, A. H. & Morken, J. P. Enantioselective Hydroformylation of 1-Alkenes with Commercial Ph-BPE Ligand. *Org. Lett.* **17**, 3264–3267 (2015).

[3] Wang, W. et al. Migratory arylboration of unactivated alkenes enabled by nickel catalysis. *Angew. Chem., Int. Ed.* ***58***, 4612–4616 (2019).

[4] Higashibayashi, S., Mori, T., Shinko, K., Hashimoto, K. & Nakata, M. Synthetic studies on thiostrepton family of peptide antibiotics: synthesis of the tetrasubstituted dihydroquinoline portion of siomycin D_1. *Heterocycles* **57**, 111–122 (2002).

[5] For the synthesis of ligand **L5**, see: Su, B. et al. A Chiral Nitrogen Ligand for Enantioselective, Iridium-Catalyzed Silylation of Aromatic C−H Bonds. *Angew. Chem. Int. Ed.* **129**, 1112–1116 (2017).

[6] Han, C. et al. Palladium-catalyzed remote 1,n-arylamination of unactivated terminal alkenes. *ACS Catal.* **9**, 4196–4202 (2019).

[7] Mandal, S., Verma, P. K. & Geetharani, K. Lewis acid catalysis: regioselective hydroboration of alkynes and alkenes promoted by scandium triflate. *Chem. Commun.* **54**, 13690–13693 (2018).

[8] Chen, K. et al. Dinuclear Cobalt Complex-Catalyzed Stereodivergent Semireduction of Alkynes: Switchable Selectivities Controlled by H_2O. *ACS Catal.* **11**, 13696–13705 (2011).

[9] Seto, C., Otsuka, T., Takeuchi, Y., Tabuchi, D. & Nagano, T. Iron-catalyzed Grignard cross-couplings with allylic methyl ethers or allylic trimethylsilyl ethers. *Synlett* **29**, 1211–1214 (2018).

[10] Delcamp, J. H. & White, M. C. Sequential Hydrocarbon Functionalization: Allylic C−H Oxidation/Vinylic C−H Arylation. *J. Am. Chem. Soc.* **128**, 15076–15077 (2006).

[11] Delcamp, J. H., Brucks, A. P. & White, M. C. *J. Am. Chem. Soc.* **130**, 11270–11271 (20086).

[12] Wang, Z. et al. Silver-Catalyzed decarboxylative chlorination of aliphatic carboxylic acids. *J. Am. Chem. Soc.* **134**, 4258–4263 (2012).

[13] Tan, X. et al. Silver-catalyzed decarboxylative bromination of aliphatic carboxylic acids. *Org. Lett.* **19**, 1634–1637 (2017).

[14] Yang, T., Fan, X., Zhao, X. & Yu, W. Iron-catalyzed acyl migration of tertiary α-azidyl ketones: synthetic approach toward enamides and isoquinolones. *Org. Lett.* **20**, 1875–1879 (2018).

[15] Ren, X. et al. Access to α-cyano carbonyls bearing a quaternary carbon center by reductive cyanation. *Org. Lett.* **23**, 2527–2532 (2021).

[16] Bégué, J. P., Bonnet, D., Charpentier-Morize, M. & Pardo, C. Ions α-cetocarbenium. Influence de la structure sur l'evolution des ions α-cetocyclohexylcarbenium. *Tetrahedron* **31**, 2505–2511 (1975).
